# Pegcetacoplan for Adolescents with C3 Glomerulopathy or Primary Immune Complex Membranoproliferative GN: Phase 3 VALIANT Subgroup Analysis

**DOI:** 10.2215/CJN.0000001077

**Published:** 2026-05-14

**Authors:** Marina Vivarelli, Gema Ariceta, Yael Borovitz, Bradley P. Dixon, Larry A. Greenbaum, Christoph Licht, Antonio Mastrangelo, Nabil Melhem, Naoya Fujita, Nicole C.A.J. van de Kar, Dean Wallace, Eli Khankin, Zhongshen Wang, Luis López-Lázaro, Johan Szamosi, Carla M. Nester

**Affiliations:** 1 Bambino Gesù Children's Hospital, Istituto di Ricovero e Cura a Carattere Scientifico, Rome, Italy; 2 Vall d'Hebron University Hospital, Barcelona, Spain; 3 Schneider Children's Medical Center of Israel, Petah Tikva, Israel; 4 University of Colorado School of Medicine, Aurora, Colorado; 5 Emory School of Medicine, Atlanta, Georgia; 6 The Hospital for Sick Children, Toronto, Ontario, Canada; 7 Fondazione IRCCS Ca' Granda Ospedale Maggiore Policlinico, Milan, Italy; 8 Evelina London Children's Hospital, Guy's and St Thomas' NHS Foundation Trust, London, United Kingdom; 9 Aichi Children's Health and Medical Center, Aichi, Japan; 10 Amalia Children's Hospital, Radboud University Medical Center, Nijmegen, The Netherlands; 11 Royal Manchester Children's Hospital, Manchester, United Kingdom; 12 Apellis Pharmaceuticals, Inc., Waltham, Massachusetts; 13 Swedish Orphan Biovitrum AB, Stockholm, Sweden; 14 University of Iowa Stead Family Children's Hospital, Iowa City, Iowa

**Keywords:** complement, GFR, glomerulopathy, MPGN (membranoproliferative GN), proteinuria, randomized controlled trials

## Abstract

**Key Points:**

- Adolescents with C3 glomerulopathy/primary immune complex membranoproliferative GN received pegcetacoplan.
- Proteinuria reduction and stable eGFR in adolescents were consistent with the overall VALIANT population.
- Pegcetacoplan may resolve pathophysiology underlying C3 glomerulopathy/primary immune complex membranoproliferative GN and prevent long-term kidney damage in a key patient population.

**Background:**

In VALIANT (phase 3; NCT05067127), pegcetacoplan (C3/C3b inhibitor) led to significant proteinuria reduction (>68% versus placebo) and eGFR stabilization in native kidney and posttransplant recurrent C3 glomerulopathy and primary immune complex membranoproliferative GN. Here, we describe results for adolescents (12–17 years) in VALIANT.

**Methods:**

Patients (randomized 1:1) received pegcetacoplan twice weekly or placebo for 26 weeks. For overall population, the primary end point was baseline-to-week 26 change in log-transformed urine protein-to-creatinine ratio (UPCR) in pegcetacoplan versus placebo arms. Key secondary end points were patients achieving composite renal end point (≤15% eGFR reduction and ≥50% UPCR reduction), patients achieving ≥50% UPCR reduction, and eGFR change.

**Results:**

Twenty-eight adolescents received pegcetacoplan; 27 received placebo. Consistent with overall population, pegcetacoplan-treated adolescents achieved clinically meaningful UPCR reduction (relative reduction, 75% [95% confidence interval], 59 to 84; nominal *P* < 0.001). More adolescents in pegcetacoplan versus placebo arms achieved the composite end point (57% versus 4%; nominal *P* = 0.002) and ≥50% UPCR reduction (71% versus 4%; nominal *P* < 0.001). eGFR was stable for pegcetacoplan-treated adolescents (adjusted least squares mean difference [95% confidence interval] versus placebo, +9.7 [−0.026 to 19.382] ml/min per 1.73 m^2^; nominal *P* = 0.05). Three adolescents in each group experienced serious treatment-emergent adverse events (pyrexia in one pegcetacoplan-treated patient was considered treatment related). None experienced infection caused by encapsulated bacteria.

**Conclusions:**

Pegcetacoplan induced clinically meaningful proteinuria reduction and eGFR stabilization compared with placebo in adolescents with C3 glomerulopathy or primary immune complex membranoproliferative GN and was well tolerated.

**Clinical Trial registry name and registration number:**

ClincialTrials.gov, NCT05067127.

## Visual Abstract

**Figure:**
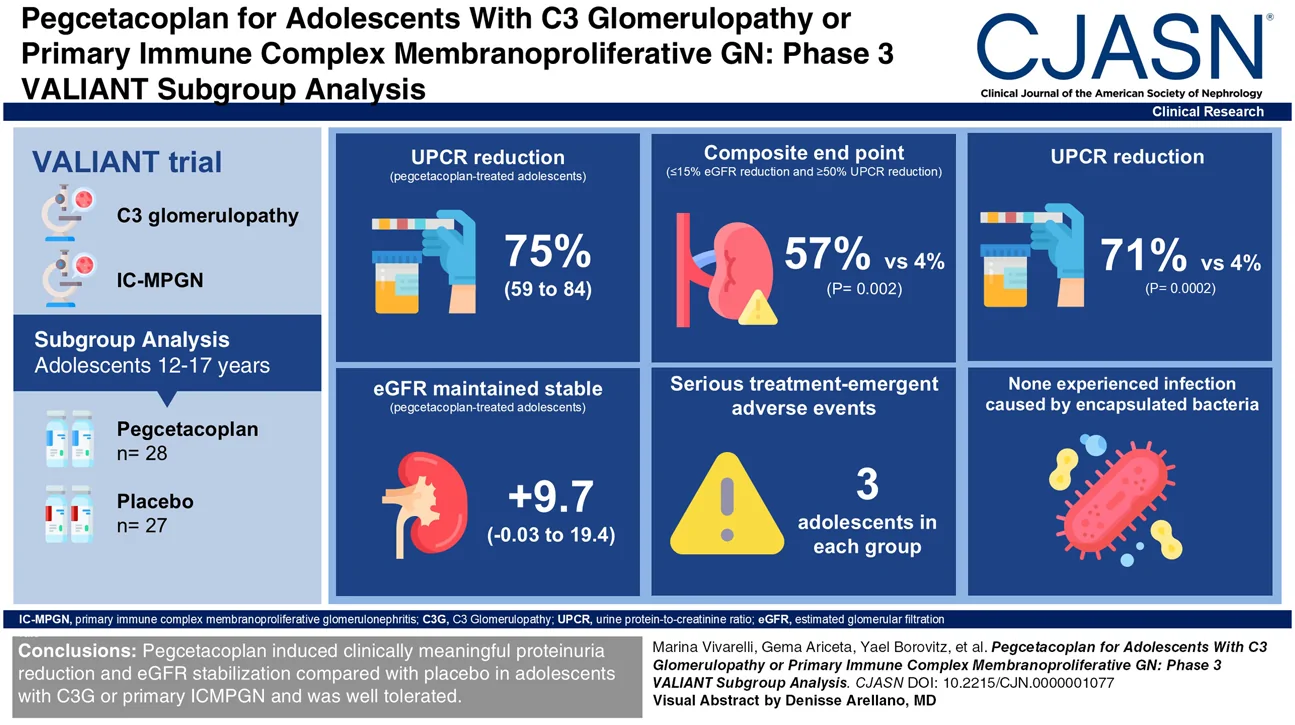

## Introduction

C3 glomerulopathy (C3G) and primary (idiopathic) immune complex membranoproliferative GN (IC-MPGN) are rare, chronic, heterogeneous, complement-mediated diseases with a high unmet need in pediatric patients. These glomerulopathies often manifest after an infectious or autoimmune trigger that activates the alternative complement pathway^1–3^ and are driven by complement dysregulation. Overactivity of the complement system results in the accumulation of C3 effectors in the glomeruli, inflammation, and progressive kidney damage—often leading to permanent loss of kidney function.^2,4^

Both C3G and primary IC-MPGN are frequently diagnosed during adolescence and early adulthood. Approximately one half of patients with C3G are diagnosed when they are younger than 18 years, and the median age at diagnosis of primary IC-MPGN is approximately 21 years.^5^ Children and adolescents with C3G or primary IC-MPGN present with varying degrees of proteinuria (ranging from mild to nephrotic range) and hematuria (ranging from microscopic to macroscopic), and most have low serum C3 levels.^3,6–9^ A kidney biopsy is required for diagnosis, with isolated or dominant C3 deposits on immunofluorescence being the hallmark of C3G; IC-MPGN shows significant deposits of both C3 and Ig deposits.^2,4,10^

Frequently, autoantibodies known as nephritic factors and, less frequently, pathogenic variants in genes of the alternative pathway of the complement system are identified in patients with C3G and primary IC-MPGN. In pediatric patients, IC-MPGN is typically idiopathic; in adults, secondary causes of the disease may lead to worse prognoses.^7^ Although preserved eGFR has been observed in some children and adolescents with C3G or primary IC-MPGN, the diseases can have an aggressive presentation in many young patients, who, alongside their families, face considerable burden of disease.^6,7,9^ Approximately 20% of children will progress to kidney failure within 10–15 years of C3G or primary IC-MPGN diagnosis despite standard-of-care treatment,^9^ requiring dialysis or kidney transplantation. Unfortunately, disease recurrence and graft loss after transplantation are frequent,^9,11,12^ with C3 deposits detectable in up to 89% of patients within a median of 1 month after transplantation,^12^ and children may have a higher rate of recurrence than adults.^13^ Published data describing outcomes in pediatric patients treated for complement-mediated disease recurrence are limited.^11,14^ Until the approval of pegcetacoplan in the United States in July 2025 and in Europe in January 2026,^15,16^ there were no approved treatments for C3G or primary IC-MPGN in adolescents, with standard of care for pediatric patients focusing on symptom management and not the underlying disease mechanism.^17^

Pegcetacoplan, a targeted C3 and C3b inhibitor, acts centrally in the complement cascade: it blocks C3 cleavage by both the alternative and classical/lectin convertases and subsequent generation of downstream complement activation effectors (C3b, C3 alternative convertase, and C5 convertases) together with the amplification loop. In this way, pegcetacoplan displays comprehensive control over C3 dysregulation.^15–22^ Pegcetacoplan inhibition of C3 and C5 convertases^22^ is predicted to stop glomerular C3 and C5 activation in C3G and primary IC-MPGN, preventing associated inflammation, structural damage, and, ultimately, the consequent kidney failure.^18^

VALIANT (NCT05067127) was a double-blind, randomized, placebo-controlled, phase 3 trial evaluating the safety and efficacy of pegcetacoplan for adolescents (age 12–17 years) and adults (age ≥18 years) with C3G or primary IC-MPGN occurring as native kidney disease or posttransplant disease recurrence. The primary results from the overall population demonstrated a clinically and statistically significant reduction in proteinuria, decreased C3 staining on kidney biopsy, and stable kidney function with 26 weeks of pegcetacoplan treatment, as well as an acceptable safety profile.^23^ Here, we describe the results during the 26-week randomized controlled period (RCP) from the adolescent subgroup of the VALIANT population.

## Methods

The full study methodology of VALIANT has been reported elsewhere.^23^ Adolescents followed the same protocol as adults, with only a few minor differences, the most notable of which was the lack of requirement for serial kidney biopsies for adolescent patients during the study period. In addition, adolescents were not required to have a biopsy within 28 weeks of randomization if historical biopsy results were available (Supplemental Methods).

### Ethics Considerations

All patients (or their caregivers) provided written informed consent and assent before study participation. The study was conducted in accordance with the International Council for Harmonisation of Technical Requirements for Pharmaceuticals for Human Use E6 Guidelines for Good Clinical Practice, the Declaration of Helsinki, all applicable regulatory requirements, and Institutional Review Board or independent ethics committee requirements at each site. An independent data monitoring committee reviewed the safety and efficacy data throughout the trial.

### Adolescent Population

VALIANT was a multicenter, randomized, double-blind, placebo-controlled, phase 3 trial to evaluate the efficacy and safety of pegcetacoplan for patients with native or posttransplant recurrent C3G or primary IC-MPGN. Patients with clinical and pathologic evidence of C3G or primary IC-MPGN were randomized 1:1 to pegcetacoplan or placebo treatment groups. Randomized adolescents (age 12–17 years) were stratified by transplant status (no transplant versus posttransplant recurrence).^23^

Patients were required to be receiving stable (≥12 weeks before randomization), optimized (per investigator's discretion) supportive care regimens for C3G or primary IC-MPGN (Supplemental Table 1). Vaccinations against *Streptococcus pneumoniae*, *Neisseria meningitidis* (types A, C, W, Y, and B), and *Hemophilus influenzae* (type B) were required to be initiated at least 14 days before receiving the first dose of study drug. Vaccination practices aligned with current recommendations of the Advisory Committee on Immunization Practices. Patients with C3G or primary IC-MPGN secondary to other causes (*e.g*., infection, systemic autoimmune diseases, or monoclonal gammopathies) or with evidence of kidney transplant rejection or improving kidney disease were excluded from the study.

### Study Design

The study comprised a screening period, an RCP, an open-label period (OLP), and a follow-up period (Supplemental Figure 1). The 10-week screening period included two study visits to determine eligibility and establish a baseline urine protein-to-creatinine ratio (UPCR). Randomized patients received pegcetacoplan or placebo in addition to stable and optimized supportive care during the 26-week, double-blind, RCP. Following the RCP, patients who completed all assessments for weeks 24 through 26 advanced to the 26-week OLP, during which all patients received pegcetacoplan plus stable and optimized supportive care. Study visits occurred every 4 weeks during the RCP, with an additional visit at week 26, and every 4 weeks during the OLP. Patients who completed the VALIANT RCP and OLP were allowed to continue to the 8-week follow-up period or enroll in the phase 3 long-term extension study (VALE; NCT05809531).^24^ The VALE study is ongoing and will generate long-term data on the safety and efficacy of pegcetacoplan.

### Medication Administration

Pegcetacoplan or placebo was administered twice weekly by subcutaneous infusion. Age- and weight-based pegcetacoplan dosages were based on modeling data expected to replicate exposures at weeks 1, 2, and 16 (steady state) that were similar to those of the adult dosage of 1080 mg twice weekly. The pegcetacoplan dosage for adolescents weighing 50 kg or more was 1080 mg (20 ml); for adolescents weighing 30–34 kg, pegcetacoplan was started with two doses of 540 mg (10 ml) followed by maintenance doses of 648 mg (12 ml); and for adolescents weighing 35–49 kg, pegcetacoplan was started with an initial dose of 648 mg and a second dose of 810 mg (15 ml), followed by maintenance doses of 810 mg. Patients and/or their caregivers were trained to administer subcutaneous infusions by a research nurse or other qualified study personnel. Rescue treatment, including high-dose corticosteroids and/or C5 inhibitors, was permitted in the event of a serum creatinine increase of two or more times the baseline concentration that the investigator attributed to the underlying disease.

### Study Assessments

Glomerular disease burden was assessed based on proteinuria measured from 24-hour urine samples, triplicate first-morning urine (FMU) samples, and random in-clinic urine samples. The bedside Schwartz equation (eGFR=0.413×[height (cm)]/serum creatinine [mg/dl]) was used to calculate eGFR for adolescents throughout the study.^25^ Protocol-directed kidney biopsies for assessment of secondary end points were not required for adolescent patients; therefore, histological end points are not described in this publication.

### Study Objectives and End Points

The primary objective of VALIANT was to assess the efficacy of pegcetacoplan compared with placebo based on proteinuria reduction in patients with C3G or primary IC-MPGN. The primary end point was the log-transformed ratio of UPCR sampled from FMU collections at week 26 relative to baseline.

The effect of pegcetacoplan treatment on eGFR and additional C3G or IC-MPGN disease-related measures, as well as safety, were assessed as secondary objectives. Key secondary efficacy end points for adolescent participants included the following: the proportion of patients who met a composite renal end point (a stable or improved eGFR [≤15% eGFR reduction] and ≥50% UPCR reduction); the proportion of patients who achieved ≥50% UPCR reduction; and the change in eGFR from baseline.

Safety was evaluated with end points including the incidence and severity of treatment-emergent adverse events (TEAEs) and death. Signs of infection (*i.e*., body temperature, vital signs, and relevant blood parameters) were monitored throughout the study. For adolescents, body height (cm) was measured at screening and every 12 weeks throughout the study; weight (kg) was measured throughout study, during brief and full physical examinations. For patients with prior kidney transplant, rejection episodes and graft loss were assessed.

### Statistical Considerations

The primary analysis, including all efficacy and safety analyses, was completed when all patients completed the RCP or discontinued early. For this prespecified adolescent subgroup analysis, efficacy end points were analyzed for all randomized adolescent patients according to the treatment group assigned at randomization. Safety end points were assessed for all adolescent patients who received at least one dose of study treatment.

Continuous outcomes were analyzed using a mixed-effect model for repeated measures, including fixed categorical effects for treatment group, study visit, disease type (C3G versus primary IC-MPGN), use of immunosuppressants at baseline (for primary end point only), stratification factors, visit-by-treatment group interaction, and fixed covariate of the baseline value of the end point. Multiple imputation was used to handle missing data and intercurrent events. A logistic regression model was used to analyze binary outcomes. This model included treatment group as an independent variable and adjusted for baseline value of the variable(s) used to define the end point, disease type, and stratification factors. For all end points described herein for adolescent patients, comparisons were not corrected for multiplicity, and, therefore, nominal *P* values are reported.

## Results

### Patient Population

VALIANT included a broad patient population with a large proportion of adolescents. In the overall population, 63 patients were randomized to the pegcetacoplan treatment group and 61 were randomized to the placebo group. Of them, 28 (44%) adolescents were included in the pegcetacoplan group and 27 (44%) adolescents were included in the placebo group (Table 1). Except for age, the baseline demographics and clinical characteristics of the adolescent population were similar to those of the overall population, and the characteristics of adolescents in the two treatment groups were similar. The mean (SD) age of the adolescent patients was 14.6 (1.7) years in the pegcetacoplan group and 14.8 (1.8) years in the placebo group. More than half of the adolescent patients were female (pegcetacoplan, 18 [64%]; placebo, 14 [52%]). Baseline proteinuria was slightly greater in the adolescent population who received pegcetacoplan (mean [SD] FMU UPCR, 3.54 [3.13] g/g) compared with the adolescent placebo group (mean [SD] FMU UPCR, 2.58 [2.33] g/g) and with the overall population (mean [SD] FMU UPCR, 2.84 [2.23] g/g). Baseline eGFR was similar between adolescents who received pegcetacoplan and those who received placebo (mean [SD] eGFR, 92.8 [32.4] versus 94.0 [34.3] ml/min per 1.73 m^2^) but was greater in adolescents compared with the overall population (mean [SD] eGFR, 93.4 [33.1] versus 82.8 [35.8] ml/min per 1.73 m^2^). One (4%) adolescent patient in the pegcetacoplan group had received a prior kidney transplant; no adolescents in the placebo group had received transplants. Most adolescents (43 [78%]) were receiving immunosuppression therapy (immunosuppressants and/or systemic corticosteroids) at baseline, consistent with the overall population.

**Table 1:** Demographic and baseline clinical characteristics

Characteristic	Adolescents		Overall Population	
	Pegcetacoplan	Placebo	Pegcetacoplan	Placebo
Patients, *No.* (%)	28 (44)	27 (44)	63 (100)	61 (100)
Age, yr, mean (SD)	14.6 (1.7)	14.8 (1.8)	28.2 (17.1)	23.6 (14.3)
Height, cm, median (IQR)	163.05 (155.25–173.55)	165.00 (160.40–175.80)	163.10 (158.00–172.00)	166.30 (161.10–178.00)
Weight, kg, median (IQR)	56.20 (47.70–62.80)	54.40 (50.50–66.20)	62.20 (53.80–70.00)	60.00 (53.30–72.00)
**Sex, *No.* (%)**				
Female	18 (64)	14 (52)	37 (59)	33 (54)
Male	10 (36)	13 (48)	26 (41)	28 (46)
**Race, *No.* (%)**				
American Indian or Alaska Native	1 (4)	0	1 (2)	0
Asian	3 (11)	4 (15)	9 (14)	9 (15)
Black	1 (4)	0	1 (2)	0
Other	3 (11)	4 (15)	7 (11)	6 (10)
White	20 (71)	19 (70)	45 (71)	46 (75)
Baseline 24-h UPCR, g/g, mean (SD)	4.6 (3.8)	4.0 (3.4)	4.0 (2.9)	3.3 (2.4)
Baseline triplicate FMU UPCR, g/g, mean (SD)	3.5 (3.1)	2.6 (2.3)	3.1 (2.4)	2.5 (2.0)
Baseline eGFR^a^, ml/min per 1.73 m^2^, mean (SD)	92.8 (32.4)	94.0 (34.3)	78.5 (34.1)	87.2 (37.2)
**Underlying disease based on screening biopsy, *No.* (%)**				
C3G^b^	21 (75)	17 (63)	51 (81)	45 (74)
C3GN	19 (68)	15 (56)	45 (71)	41 (67)
DDD	2 (7)	2 (7)	4 (6)	4 (7)
Primary IC-MPGN	7 (25)	10 (37)	12 (19)	16 (26)
Time since diagnosis, yr, mean (SD)	3.3 (2.5)	3.4 (3.5)	3.6 (3.5)	3.8 (3.6)
Posttransplant disease recurrence, *No.* (%)	1 (4)	0	5 (8)	4 (7)
Immunosuppressant use at baseline, *No.* (%)	22 (79)	21 (78)	47 (75)	42 (69)

C3G, C3 glomerulopathy; DDD, dense deposit disease; FMU, first-morning urine; IC-MPGN, immune complex membranoproliferative GN; IQR, interquartile range; UPCR, urine protein-to-creatinine ratio.

a Calculated by the bedside Schwartz equation for adolescents and the CKD Epidemiology Collaboration equation for adults.

b Two adults (3%) in the pegcetacoplan treatment arm had an undetermined type of underlying C3 glomerulopathy based on screening biopsy.

### Primary End Point

**Figure 1:**
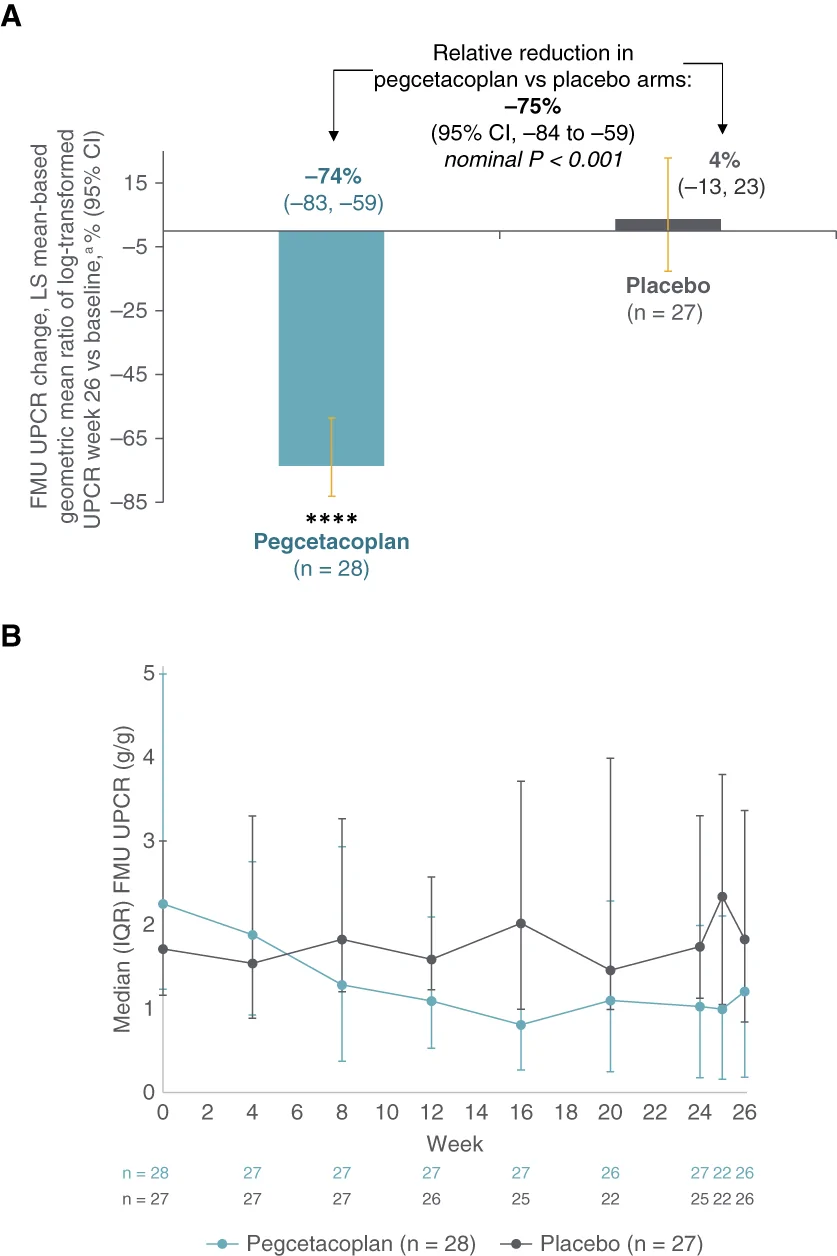
**Change in proteinuria among adolescent patients (week 26 versus baseline).** (A) Reduction in proteinuria compared with baseline. (B) Proteinuria over time. ^a^Percentages calculated by converting the ratio of geometric means to percentages. *P < 0.05, **P < 0.01, ***P < 0.001, ****P < 0.0001. CI, confidence interval; C3G, C3 glomerulopathy; DDD, dense deposit disease; FMU, first-morning urine; IC-MPGN, immune complex membranoproliferative GN; LS, least squares; UPCR, urine protein-to-creatinine ratio.

A clinically meaningful relative proteinuria reduction of 75% (95% confidence interval [CI], 59 to 84; nominal *P* < 0.001) was achieved among adolescents receiving pegcetacoplan versus placebo at week 26 (Figure 1A). Proteinuria reduction with pegcetacoplan was observed as early as week 4 and continued through week 26 (Figure 1B). The relative reduction achieved by adolescents with pegcetacoplan compared with placebo was consistent with what was observed in the overall population (relative reduction of geometric mean, 68%; 95% CI, 57 to 76; *P* < 0.001).^23^

### Secondary End Points

**Figure 2:**
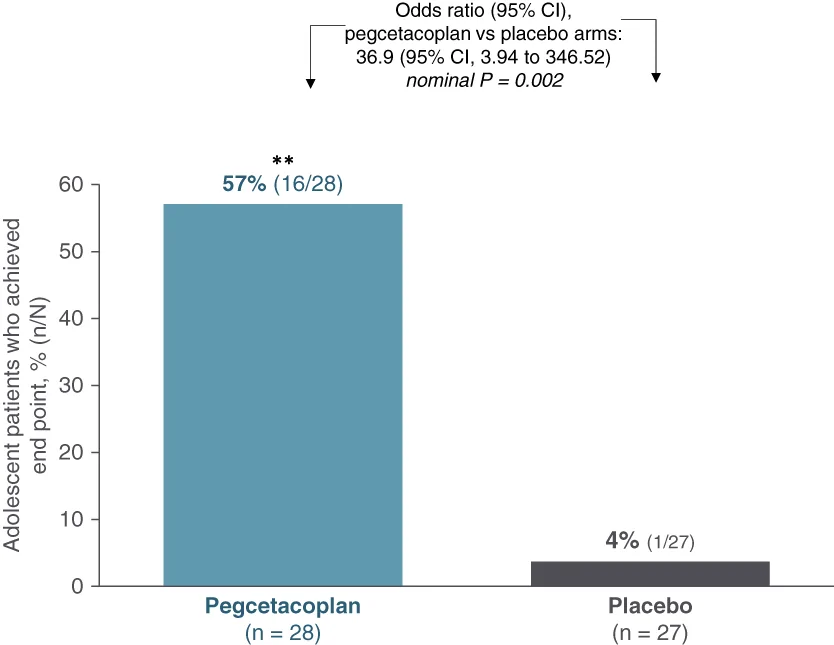
**Proportion of adolescent patients who achieved the composite renal end point**^**a,b**^ **at week 26.** ^a^Composite renal end point: a stable or improved eGFR (≤15% reduction) and a ≥50% UPCR ratio reduction. ^b^Proteinuria calculated using FMU samples. *P < 0.05, **P < 0.01, ***P < 0.001, ****P < 0.0001.

Substantially more adolescents achieved the composite renal end point with pegcetacoplan compared with placebo at week 26 (Figure 2); adolescents receiving pegcetacoplan had 37 times higher odds of achieving the composite renal end point compared with those receiving placebo (57% versus 4%; nominal *P* = 0.002). In the overall population, pegcetacoplan-treated patients had 27 times higher odds of achieving the composite renal end point (49% versus 3%; *P* < 0.001).^23^

**Figure 3:**
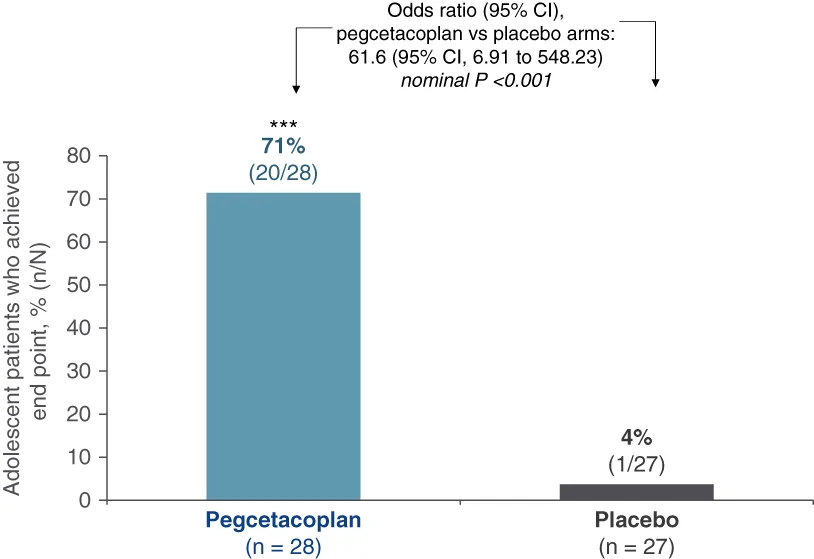
**Proportion of adolescent patients who achieved ≥50% proteinuria reduction**^**a**^ **at week 26.** ^a^Calculated using FMU samples. *P < 0.05, **P < 0.01, ***P < 0.001, ****P < 0.0001.

Twenty (71%) adolescents who received pegcetacoplan achieved ≥50% proteinuria reduction compared with only 1 (4%) adolescent patient who received placebo (Figure 3). Adolescents receiving pegcetacoplan had 62 times higher odds of achieving ≥50% proteinuria reduction compared with placebo (nominal *P* < 0.001). In the overall population, pegcetacoplan-treated patients had 31 times higher odds of reaching this end point (60% versus 5%; *P* < 0.001).^23^

**Figure 4:**
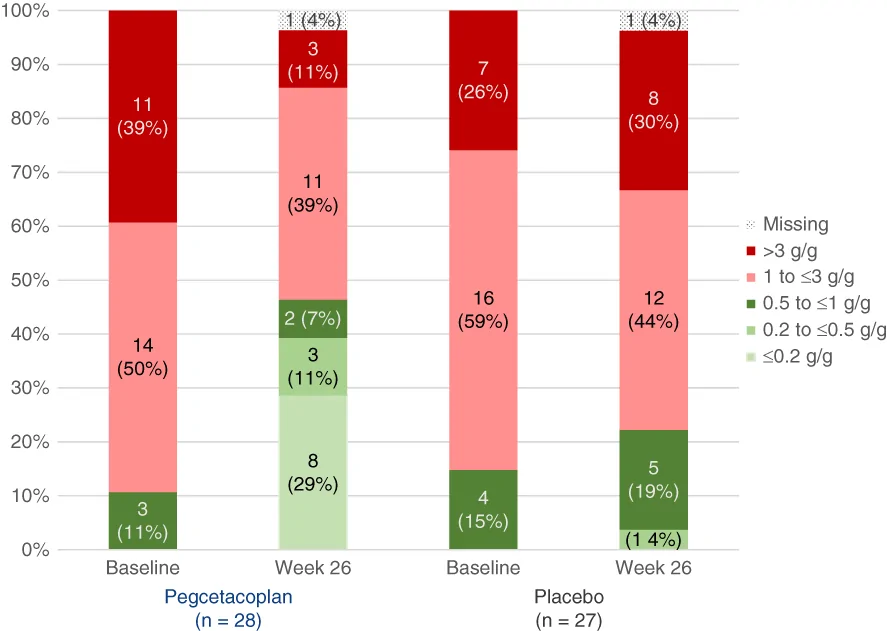
**Shift in proteinuria**^**a**^ **among adolescents (baseline to week 26).** ^a^Calculated using FMU samples.

Proteinuria reduction with pegcetacoplan resulted in a substantial increase in the proportion of adolescents with proteinuria ≤1 g/g after 26 weeks (11% at baseline versus 46% at week 26). Of these patients, 11 (39%) achieved proteinuria ≤0.5 g/g and 8 (29%) achieved ≤0.2 g/g. A corresponding decrease in the proportion of adolescents in the nephrotic range of >3 g/g was observed (39% at baseline versus 11% at week 26; Figure 4).

**Figure 5:**
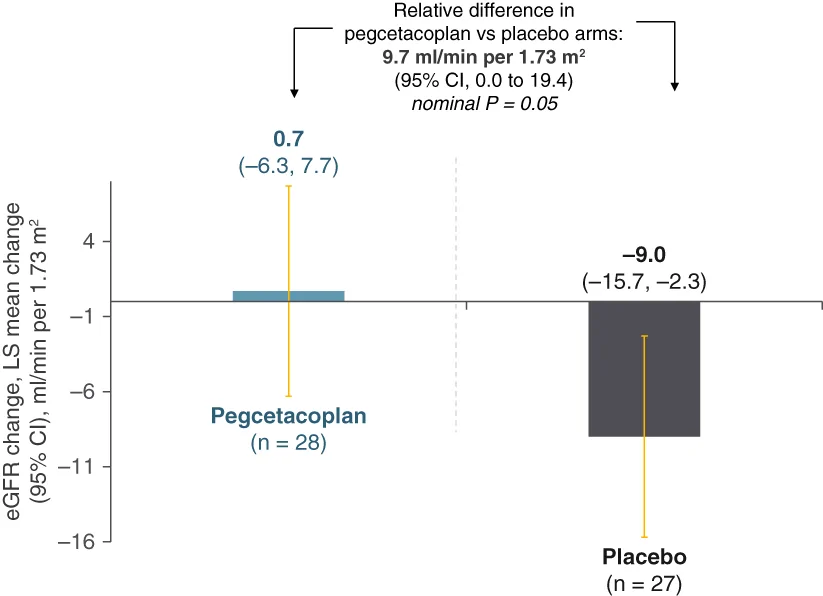
**Change in eGFR**^**a**^ **among adolescent patients (baseline to week 26).** ^a^Calculated by the bedside Schwartz equation for adolescents.

Adolescent patients receiving pegcetacoplan achieved stable eGFR during the RCP (Figure 5). The difference (least squares mean [95% CI]) in the pegcetacoplan versus placebo arms was +9.7 (−0.026 to 19.382) ml/min per 1.73 m^2^ (nominal *P* = 0.05), in favor of pegcetacoplan. The difference in the overall population was +6.3 ml/min per 1.73 m^2^ (nominal *P* = 0.03).^23^

### Safety

Pegcetacoplan demonstrated an acceptable safety profile among adolescents. TEAE frequency and severity were similar between treatment groups for the adolescent population (Table 2) and between adolescents and the overall population.^23^ In all, 23 adolescents (82%) in the pegcetacoplan group and 26 (96%) in the placebo group experienced TEAEs. Most TEAEs in the pegcetacoplan group were considered mild.

**Table 2:** Treatment-emergent adverse events among adolescent patients^a^

Patients, *No.* (%)	Pegcetacoplan (*n*=28)	Placebo (*n*=27)
**TEAEs**	23 (82)	26 (96)
Mild	12 (43)	11 (41)
Moderate	9 (32)	14 (52)
Severe	2 (7)	1 (4)
Treatment-related TEAEs	13 (46)	11 (41)
Serious TEAEs	3 (11)	3 (11)
TEAEs leading to study discontinuation	0	1 (4)
Deaths	0	0

TEAE, treatment-emergent adverse event.

a Adolescent subgroup of safety set defined as all adolescent patients who received ≥1 dose of study treatment.

Among adolescents, the most frequent classes of TEAEs in both pegcetacoplan and placebo groups were general disorders and administration site conditions (pegcetacoplan, 15 [54%]; placebo, 15 [56%]; Supplemental Table 2); most of these TEAEs were considered to be of mild severity (11 [39%] and 13 [48%], respectively). In the overall population, general disorders and administration site conditions were the second-most frequent class of TEAEs (pegcetacoplan, 29 [46%]; placebo, 29 [48%]), and infections and infestations were the most frequent (pegcetacoplan, 35 [56%]; placebo, 27 [44%]. Infections occurred among 13 adolescents in each group (pegcetacoplan, 46%; placebo, 48%). Pyrexia (pegcetacoplan, 7 [25%]; placebo, 6 [22%]) and nasopharyngitis (pegcetacoplan, 7 [25%]; placebo, 4 [15%]) were the most common TEAEs.

Serious TEAEs occurred in three (11%) adolescents in each treatment group. In the pegcetacoplan group, one adolescent reported worsening of hypertension, one reported an influenza A infection and AKI, and one experienced pyrexia of unknown etiology. The case of pyrexia was considered related to treatment by the investigator. The fever occurred an hour after the infusion and resolved shortly after; the fever recurred with the next administration and was therefore deemed a reaction to the infusion and not to the study medication, and no infection was identified as the cause of the fever. The dose of pegcetacoplan was not changed, and the patient continued the study. In the placebo group, one adolescent reported worsening of AKI; one reported a viral infection, increased creatinine, and AKI; and one reported pregnancy and miscarriage. No serious infections caused by encapsulated bacteria were reported among adolescents.

Among adolescents, one (4%) TEAE in the placebo led to study discontinuation, and none led to death. No graft loss or rejection was reported in the single posttransplant adolescent patient.

Height, weight (Supplemental Figure 2), and BP evolution (Supplemental Figure 3) did not differ between pegcetacoplan- and placebo-treated patients.

## Discussion

The phase 3 VALIANT trial was the largest trial of C3G and primary IC-MPGN to date and the first randomized controlled trial to include a large proportion of adolescents. In VALIANT, pegcetacoplan demonstrated efficacy and an acceptable safety profile for adolescent patients with C3G or primary IC-MPGN, achieving a clinically meaningful proteinuria reduction of 75% compared with placebo, as well as eGFR stabilization. Proteinuria reduction and eGFR stabilization results among adolescents were consistent with those of the full VALIANT population,^23^ confirming pegcetacoplan's safety and efficacy in a key patient population. In addition, more than 70% of pegcetacoplan-treated adults with biopsies at baseline and at week 26 achieved clearance in glomerular C3 as seen by reduction to zero in C3 staining intensity; protocol-directed biopsies were not required for adolescents. Pegcetacoplan was well tolerated among adolescents, consistent with findings of previous trials in adults and adolescents and with postmarketing experience totaling more than 3000 patient-years of pegcetacoplan exposure.

Early treatment initiation is important for patients with C3G and primary IC-MPGN, especially for adolescents, as slowing disease progression and prolonging time to kidney damage leads to improved outcomes.^26^ Specifically, proteinuria reduction has been associated with improved kidney function and slower eGFR decline in C3G and primary IC-MPGN,^27,28^ and treating toward targets such as ≥50% proteinuria reduction represents a disease-modifying treatment paradigm for complement-mediated glomerulopathies. Kidney Disease Improving Global Outcomes guidelines suggest targeting a proteinuria level of <1 g/d^29^ (equivalent to a UPCR of 0.88 g/g).^30^ In VALIANT, pegcetacoplan showed a marked improvement in proteinuria as early as 4 weeks after treatment initiation, and effects were sustained through the 26-week RCP, with 71% of pegcetacoplan-treated adolescents achieving ≥50% proteinuria reduction and 46% achieving proteinuria ≤1 g/g, with 29% achieving the lowest threshold of ≤0.2 g/g, or proteinuria normalization. The results from the full VALIANT population demonstrated the clinically important proof-of-efficacy triad described by the Kidney Health Initiative workgroup of reduced proteinuria, stable eGFR, and decreased C3 staining.^31^ This supports that pegcetacoplan targets the complement dysregulation underlying C3G and primary IC-MPGN and improves disease progression.^23^ Despite the absence of biopsy data for adolescents, there is no reason to believe the disease mechanism is different between adolescent and adult patients, so similar histology outcomes would be expected, and the proteinuria and eGFR outcomes were similar to the adult cohort, supporting pegcetacoplan's efficacy in this population. In fact, 26-week proteinuria reduction is a common primary end point in trials of glomerular diseases and serves as a predictor of improved long-term outcomes.^28,32^

To date, few adolescents have been included in clinical trials of complement-directed therapies,^33,34^ and no data have been reported to show clinical success in this group. A notable strength of VALIANT is that adolescents comprised nearly half of patients, offering much-needed information on the treatment of C3G and primary IC-MPGN in this population.

This analysis is limited to the first 6 months of treatment with pegcetacoplan; long-term safety and efficacy data are being further evaluated in the OLP of VALIANT and the VALE long-term extension study.^24^ Information regarding C3 nephritic factor and complement gene variants was not systematically collected, which could limit further analysis of the findings. In addition, serial biopsies were not required for adolescent patients, so changes in C3 staining among adolescents cannot be compared with those among the overall population, and we cannot confirm the treatment triad of decreased proteinuria, stable eGFR, and decreased C3 staining among adolescents. Finally, only one adolescent patient had received a previous kidney transplant. Posttransplant disease recurrence is common, and more data may be needed to understand treatment outcomes for posttransplant adolescents.

In conclusion, the phase 3 VALIANT trial was the largest trial in these disease indications and the first trial to complete an assessment of a large proportion of adolescents with C3G or primary IC-MPGN. Pegcetacoplan-treated adolescents achieved reduced proteinuria and stable eGFR with 6 months of pegcetacoplan treatment, confirming its efficacy in a broad patient population.

## Supplementary Material

## Data Availability

Original data generated for the study will be made available upon reasonable request to the corresponding author. Data Type: Clinical Trial Data and Statistical Analysis Plan. Reason for Restricted Access: The data reported in this article will be made available by the sponsor and/or corresponding author upon reasonable request. Individual participant data will not be made available to protect the anonymity of patients included in the trial. All proposals requesting data access will need to specify how the data will be used, and all proposals will need the approval of the trial investigator team before data release following a research proposal and execution of a data-sharing agreement.

## Declarative Statements

This study includes clinical experimentation and received Institutional Review Board or Ethics Committee approval. All patients provided written informed consent. This study includes clinical experimentation and complies with the Declaration of Helsinki.

## Supplemental Material

Supplemental Methods

Supplemental Table 1. VALIANT key inclusion and exclusion criteria.

Supplemental Table 2. TEAEs according to system organ class and preferred term among adolescent patients.

Supplemental Figure 1. VALIANT study design.

Supplemental Figure 2. Growth during the VALIANT RCP.

Supplemental Figure 3. BP during VALIANT RCP.

Statistical Analysis Plan
