# Supplementary material for: Pegcetacoplan for Adolescents with C3 Glomerulopathy or Primary Immune Complex Membranoproliferative GN: Phase 3 VALIANT Subgroup Analysis
Source: Clin J Am Soc Nephrol. 2026 May 14;21(8):1404–13. doi: 10.2215/CJN.0000001077 (PMC13493029; doi:10.2215/CJN.0000001077)
Supplement: Supplementary file 1 [file cjasn-21-1404-s001.pdf]

ASN American Society of Nephrology
JASN Journal of the American Society of Nephrology
KIDNEY360 Accessing Our World From Every Angle
CJASN Clinical Journal of the American Society of Nephrology

ASN Journal Disclosure Form

As per ASN journal policy, I have disclosed any financial relationships or commitments I have held in the past 36 months as included below. I have listed my Current Employer below to indicate there is a relationship requiring disclosure. If no relationship exists, my Current Employer is not listed.

M. Ariceta Iraola reports the following:
Advisory or Leadership Role: Alexion. Astra-Zeneca Rare Diseases; Dicerna; Alnylam; Chiesi, Sobi, Kyowa Kirin

I understand that the information above will be published within the journal article, if accepted, and that failure to comply and/or to accurately and completely report the potential financial conflicts of interest could lead to the following: 1) Prior to publication, article rejection, or 2) Post-publication, sanctions ranging from, but not limited to, issuing a correction, reporting the inaccurate information to the authors' institution, banning authors from submitting work to ASN journals for varying lengths of time, and/or retraction of the published work.

Name: María Gema Ariceta Iraola
Manuscript ID: CJASN-2025-001913R3
Manuscript Title: Pegcetacoplan for Adolescents With C3 Glomerulopathy or Primary Immune Complex Membranoproliferative Glomerulonephritis: Phase 3 VALIANT Subgroup Analysis
Date of Completion: April 12, 2026
Disclosure Updated Date: April 12, 2026

ASN
American Society of Nephrology
JASN
Journal of the American Society of Nephrology
KIDNEY360
Accessing Our World From Every Angle
CJASN
Clinical Journal of the American Society of Nephrology

ASN Journal Disclosure Form

As per ASN journal policy, I have disclosed any financial relationships or commitments I have held in the past 36 months as included below. I have listed my Current Employer below to indicate there is a relationship requiring disclosure. If no relationship exists, my Current Employer is not listed.

Y. Borovitz reports the following:
Employer: schneider childrens medical center; Consultancy: Novartis, Sobi; Research Funding: Cystinosis research foundation; and Honoraria: Novartis, Neopharm, Medison.

I understand that the information above will be published within the journal article, if accepted, and that failure to comply and/or to accurately and completely report the potential financial conflicts of interest could lead to the following: 1) Prior to publication, article rejection, or 2) Post-publication, sanctions ranging from, but not limited to, issuing a correction, reporting the inaccurate information to the authors' institution, banning authors from submitting work to ASN journals for varying lengths of time, and/or retraction of the published work.

Name: Yael Borovitz
Manuscript ID: CJASN-2025-001913R1
Manuscript Title: Pegcetacoplan for Adolescents With C3 Glomerulopathy or Primary Immune Complex Membranoproliferative Glomerulonephritis: Phase 3 VALIANT Subgroup Analysis
Date of Completion: February 27, 2026
Disclosure Updated Date: February 27, 2026

ASN
American Society of Nephrology
JASN
Journal of the American Society of Nephrology
KIDNEY360
Accessing Our World From Every Angle
CJASN
Clinical Journal of the American Society of Nephrology

ASN Journal Disclosure Form

As per ASN journal policy, I have disclosed any financial relationships or commitments I have held in the past 36 months as included below. I have listed my Current Employer below to indicate there is a relationship requiring disclosure. If no relationship exists, my Current Employer is not listed.

B. Dixon reports the following:
Employer: University of Colorado School of Medicine; Consultancy: Alexion AstraZeneca Rare Disease, Apellis Pharmaceuticals, Novartis Pharmaceuticals, Arrowhead Pharmaceuticals, Calliditas Therapeutics, Roche Genentech; Research Funding: Alexion AstraZeneca Rare Disease, Apellis Pharmaceuticals, Novartis Pharmaceuticals, Roche Genentech, Ultragenyx Pharmaceuticals; Honoraria: Alexion AstraZeneca Rare Disease, Apellis Pharmaceuticals, Novartis Pharmaceuticals, Calliditas Therapeutics, Roche Genentech; and Advisory or Leadership Role: aHUS Action Network (unpaid).

I understand that the information above will be published within the journal article, if accepted, and that failure to comply and/or to accurately and completely report the potential financial conflicts of interest could lead to the following: 1) Prior to publication, article rejection, or 2) Post-publication, sanctions ranging from, but not limited to, issuing a correction, reporting the inaccurate information to the authors' institution, banning authors from submitting work to ASN journals for varying lengths of time, and/or retraction of the published work.

Name: Bradley P. Dixon
Manuscript ID: CJASN-2025-001913R1
Manuscript Title: Pegcetacoplan for Adolescents With C3 Glomerulopathy or Primary Immune Complex Membranoproliferative Glomerulonephritis: Phase 3 VALIANT Subgroup Analysis
Date of Completion: February 20, 2026
Disclosure Updated Date: January 16, 2026

ASN American Society of Nephrology
JASN Journal of the American Society of Nephrology
KIDNEY360 Accessing Our World From Every Angle
CJASN Clinical Journal of the American Society of Nephrology

ASN Journal Disclosure Form

As per ASN journal policy, I have disclosed any financial relationships or commitments I have held in the past 36 months as included below. I have listed my Current Employer below to indicate there is a relationship requiring disclosure. If no relationship exists, my Current Employer is not listed.

N. Fujita reports the following:
Advisory or Leadership Role: Swedish Orphan Biovitrum Japan Co.,Ltd

I understand that the information above will be published within the journal article, if accepted, and that failure to comply and/or to accurately and completely report the potential financial conflicts of interest could lead to the following: 1) Prior to publication, article rejection, or 2) Post-publication, sanctions ranging from, but not limited to, issuing a correction, reporting the inaccurate information to the authors' institution, banning authors from submitting work to ASN journals for varying lengths of time, and/or retraction of the published work.

Name: Naoya Fujita
Manuscript ID: CJASN-2025-001913R1
Manuscript Title: Pegcetacoplan for Adolescents With C3 Glomerulopathy or Primary Immune Complex Membranoproliferative Glomerulonephritis: Phase 3 VALIANT Subgroup Analysis
Date of Completion: February 20, 2026
Disclosure Updated Date: February 20, 2026

ASN American Society of Nephrology | JASN Journal of the American Society of Nephrology | KIDNEY360 Accessing Our World From Every Angle | CJASN Clinical Journal of the American Society of Nephrology

ASN Journal Disclosure Form

As per ASN journal policy, I have disclosed any financial relationships or commitments I have held in the past 36 months as included below. I have listed my Current Employer below to indicate there is a relationship requiring disclosure. If no relationship exists, my Current Employer is not listed.

L. Greenbaum reports the following:
Employer: Emory University and Children's Healthcare of Atlanta (hospital); Consultancy: Novartis, Advicenne, Alexion, Genentech, Apellis, and Otsuka; Research Funding: Novartis, Alexion, Apellis, Aurinia, Vertex, and Genentech; Honoraria: Alexion, Apellis; and Other Interests or Relationships: DSMB payments: Travere, NS Pharma, Vifor.

I understand that the information above will be published within the journal article, if accepted, and that failure to comply and/or to accurately and completely report the potential financial conflicts of interest could lead to the following: 1) Prior to publication, article rejection, or 2) Post-publication, sanctions ranging from, but not limited to, issuing a correction, reporting the inaccurate information to the authors' institution, banning authors from submitting work to ASN journals for varying lengths of time, and/or retraction of the published work.

Name: Larry A. Greenbaum
Manuscript ID: CJASN-2025-001913R1
Manuscript Title: Pegcetacoplan for Adolescents With C3 Glomerulopathy or Primary Immune Complex Membranoproliferative Glomerulonephritis: Phase 3 VALIANT Subgroup Analysis
Date of Completion: February 20, 2026
Disclosure Updated Date: February 20, 2026

ASN American Society of Nephrology | JASN Journal of the American Society of Nephrology | KIDNEY360 Accessing Our World From Every Angle | CJASN Clinical Journal of the American Society of Nephrology

ASN Journal Disclosure Form

As per ASN journal policy, I have disclosed any financial relationships or commitments I have held in the past 36 months as included below. I have listed my Current Employer below to indicate there is a relationship requiring disclosure. If no relationship exists, my Current Employer is not listed.

E. Khankin reports the following:
Employer: Apellis Inc.; Ownership Interest: Apellis Inc. (stocks and shares); and Other Interests or Relationships: ASN member, FASN; AST member, FAST.

I understand that the information above will be published within the journal article, if accepted, and that failure to comply and/or to accurately and completely report the potential financial conflicts of interest could lead to the following: 1) Prior to publication, article rejection, or 2) Post-publication, sanctions ranging from, but not limited to, issuing a correction, reporting the inaccurate information to the authors' institution, banning authors from submitting work to ASN journals for varying lengths of time, and/or retraction of the published work.

Name: Eliyahu V. Khankin
Manuscript ID: CJASN-2025-001913R3
Manuscript Title: "Pegcetacoplan for Adolescents With C3 Glomerulopathy or Primary Immune Complex Membranoproliferative Glomerulonephritis: Phase 3 VALIANT Subgroup Analysis."
Date of Completion: April 14, 2026
Disclosure Updated Date: April 14, 2026

ASN American Society of Nephrology
JASN Journal of the American Society of Nephrology
KIDNEY360 Accessing Our World From Every Angle
CJASN Clinical Journal of the American Society of Nephrology

ASN Journal Disclosure Form

As per ASN journal policy, I have disclosed any financial relationships or commitments I have held in the past 36 months as included below. I have listed my Current Employer below to indicate there is a relationship requiring disclosure. If no relationship exists, my Current Employer is not listed.

C. Licht reports the following:
Employer: The Hospital for Sick Children; Consultancy: Alexion, AstraZeneca Rare Disease; Biogen / Apellis Pharmaceuticals, Inc.; Novartis; Oak Bay Biosciences; Sobi; Honoraria: Alexion, AstraZeneca Rare Disease; Biogen / Apellis Pharmaceuticals, Inc.; Novartis; Oak Bay Biosciences; Sobi; Patents or Royalties: [International #: WO 2007/038995 A1]; [U.S. #: 11/992,194]; [CSL Behring #: 2005_M006_A105]; [Finnegan #: 06478.1518-00000]; Title: Factor H for the treatment of chronic nephropathies and production thereof.; Advisory or Leadership Role: Editorial Boards: Kidney International; Nephrology Dialysis Transplantation; Pediatric Nephrology; Advisory Committees: Alexion, AstraZeneca Rare Disease (SAB, Global aHUS Registry); DOUBLE PRO-TECT Alport and EMPA Alport (DSMB); Argenx-Axio Research (DSMB); OPKO Health, Inc. (DSMB, CTAP101-CL-3007); Rocket Pharma (Clinical Monitoring Team, RP-A501-0123); and Speakers Bureau: Alexion, AstraZeneca Rare Disease; Sobi.

I understand that the information above will be published within the journal article, if accepted, and that failure to comply and/or to accurately and completely report the potential financial conflicts of interest could lead to the following: 1) Prior to publication, article rejection, or 2) Post-publication, sanctions ranging from, but not limited to, issuing a correction, reporting the inaccurate information to the authors' institution, banning authors from submitting work to ASN journals for varying lengths of time, and/or retraction of the published work.

Name: Christoph Licht
Manuscript ID: CJASN-2025-001913R3
Manuscript Title: Pegcetacoplan for Adolescents With C3 Glomerulopathy or Primary Immune Complex Membranoproliferative Glomerulonephritis: Phase 3 VALIANT Subgroup Analysis
Date of Completion: April 22, 2026
Disclosure Updated Date: April 22, 2026

ASN American Society of Nephrology | JASN Journal of the American Society of Nephrology | KIDNEY360 Accessing Our World From Every Angle | CJASN Clinical Journal of the American Society of Nephrology

ASN Journal Disclosure Form

As per ASN journal policy, I have disclosed any financial relationships or commitments I have held in the past 36 months as included below. I have listed my Current Employer below to indicate there is a relationship requiring disclosure. If no relationship exists, my Current Employer is not listed.

L. López Lázaro reports the following:
Employer: Sobi AG; Ownership Interest: Stock: Dr Reddy's Laboratories ; Stock Options: Sobi AG; and Patents or Royalties: Pharmamar SAU (authorship in Patent applications with no economic rights).

I understand that the information above will be published within the journal article, if accepted, and that failure to comply and/or to accurately and completely report the potential financial conflicts of interest could lead to the following: 1) Prior to publication, article rejection, or 2) Post-publication, sanctions ranging from, but not limited to, issuing a correction, reporting the inaccurate information to the authors' institution, banning authors from submitting work to ASN journals for varying lengths of time, and/or retraction of the published work.

Name: Luis López Lázaro
Manuscript ID: CJASN-2025-001913R1
Manuscript Title: Pegcetacoplan for Adolescents With C3 Glomerulopathy or Primary Immune Complex Membranoproliferative Glomerulonephritis: Phase 3 VALIANT Subgroup Analysis
Date of Completion: February 25, 2026
Disclosure Updated Date: February 25, 2026

ASN
American Society of Nephrology
JASN
Journal of the American Society of Nephrology
KIDNEY360
Accessing Our World From Every Angle
CJASN
Clinical Journal of the American Society of Nephrology

ASN Journal Disclosure Form

As per ASN journal policy, I have disclosed any financial relationships or commitments I have held in the past 36 months as included below. I have listed my Current Employer below to indicate there is a relationship requiring disclosure. If no relationship exists, my Current Employer is not listed.

A. Mastrangelo reports the following:
Employer: Fondazione IRCCS Ca' Granda Ospedale Maggiore Policlinico Milano; and Consultancy: Sweedish Orphan BIovitrium (SOBI); Novartis.

I understand that the information above will be published within the journal article, if accepted, and that failure to comply and/or to accurately and completely report the potential financial conflicts of interest could lead to the following: 1) Prior to publication, article rejection, or 2) Post-publication, sanctions ranging from, but not limited to, issuing a correction, reporting the inaccurate information to the authors' institution, banning authors from submitting work to ASN journals for varying lengths of time, and/or retraction of the published work.

Name: Antonio Mastrangelo
Manuscript ID: CJASN-2025-001913R1
Manuscript Title: Pegcetacoplan for Adolescents With C3 Glomerulopathy or Primary Immune Complex Membranoproliferative Glomerulonephritis: Phase 3 VALIANT Subgroup Analysis
Date of Completion: March 7, 2026
Disclosure Updated Date: March 7, 2026

ASN American Society of Nephrology | JASN Journal of the American Society of Nephrology | KIDNEY360 Accessing Our World From Every Angle | CJASN Clinical Journal of the American Society of Nephrology

ASN Journal Disclosure Form

As per ASN journal policy, I have disclosed any financial relationships or commitments I have held in the past 36 months as included below. I have listed my Current Employer below to indicate there is a relationship requiring disclosure. If no relationship exists, my Current Employer is not listed.

N. Melhem reports the following:
Employer: Evelina London Children's hospital; Consultancy: SOBI; SERB pharmaceutical ; Recordati; and Honoraria: SOBI.

I understand that the information above will be published within the journal article, if accepted, and that failure to comply and/or to accurately and completely report the potential financial conflicts of interest could lead to the following: 1) Prior to publication, article rejection, or 2) Post-publication, sanctions ranging from, but not limited to, issuing a correction, reporting the inaccurate information to the authors' institution, banning authors from submitting work to ASN journals for varying lengths of time, and/or retraction of the published work.

Name: Nabil Melhem
Manuscript ID: CJASN-2025-001913R1
Manuscript Title: Pegcetacoplan for Adolescents With C3 Glomerulopathy or Primary Immune Complex Membranoproliferative Glomerulonephritis: Phase 3 VALIANT Subgroup Analysis
Date of Completion: February 24, 2026
Disclosure Updated Date: February 24, 2026

ASN American Society of Nephrology | JASN Journal of the American Society of Nephrology | KIDNEY360 Accessing Our World From Every Angle | CJASN Clinical Journal of the American Society of Nephrology

ASN Journal Disclosure Form

As per ASN journal policy, I have disclosed any financial relationships or commitments I have held in the past 36 months as included below. I have listed my Current Employer below to indicate there is a relationship requiring disclosure. If no relationship exists, my Current Employer is not listed.

C. Nester reports the following:
Consultancy: Novartis (Relationship has not ended); Apellis (Relationship has not ended); Vertex (Relationship has not ended); AstraZeneca (Relationship has not ended); Biocryst (Relationship has not ended); and Research Funding: Novartis (Relationship has not ended); Apellis (Relationship has not ended); Biocryst (Relationship has not ended); Retrophin (Relationship has not ended).

I understand that the information above will be published within the journal article, if accepted, and that failure to comply and/or to accurately and completely report the potential financial conflicts of interest could lead to the following: 1) Prior to publication, article rejection, or 2) Post-publication, sanctions ranging from, but not limited to, issuing a correction, reporting the inaccurate information to the authors' institution, banning authors from submitting work to ASN journals for varying lengths of time, and/or retraction of the published work.

Name: Carla M. Nester
Manuscript ID: CJASN-2025-001913R2.
Manuscript Title: Pegcetacoplan for Adolescents With C3 Glomerulopathy or Primary Immune Complex Membranoproliferative Glomerulonephritis: Phase 3 VALIANT Subgroup Analysis
Date of Completion: March 25, 2026
Disclosure Updated Date: March 25, 2026

ASN American Society of Nephrology | JASN Journal of the American Society of Nephrology | KIDNEY360 Accessing Our World From Every Angle | CJASN Clinical Journal of the American Society of Nephrology

ASN Journal Disclosure Form

As per ASN journal policy, I have disclosed any financial relationships or commitments I have held in the past 36 months as included below. I have listed my Current Employer below to indicate there is a relationship requiring disclosure. If no relationship exists, my Current Employer is not listed.

J. Szamosi reports the following:
Employer: Sobi; and Ownership Interest: Sobi.

I understand that the information above will be published within the journal article, if accepted, and that failure to comply and/or to accurately and completely report the potential financial conflicts of interest could lead to the following: 1) Prior to publication, article rejection, or 2) Post-publication, sanctions ranging from, but not limited to, issuing a correction, reporting the inaccurate information to the authors' institution, banning authors from submitting work to ASN journals for varying lengths of time, and/or retraction of the published work.

Name: Johan Szamosi
Manuscript ID: CJASN-2025-001913R2
Manuscript Title: Pegcetacoplan for Adolescents With C3 Glomerulopathy or Primary Immune Complex Membranoproliferative Glomerulonephritis: Phase 3 VALIANT Subgroup Analysis
Date of Completion: March 25, 2026
Disclosure Updated Date: March 25, 2026

ASN
American Society of Nephrology
JASN
Journal of the American Society of Nephrology
KIDNEY360
Accessing Our World From Every Angle
CJASN
Clinical Journal of the American Society of Nephrology

ASN Journal Disclosure Form

As per ASN journal policy, I have disclosed any financial relationships or commitments I have held in the past 36 months as included below. I have listed my Current Employer below to indicate there is a relationship requiring disclosure. If no relationship exists, my Current Employer is not listed.

N. Van De Kar reports the following:
Employer: Radboudumc, Nijmegen, the Netherlands; Consultancy: Roche , Novartis, Alexion, Samsung, Sobi consultancy fees; and Speakers Bureau: Novartis, Sobi.

I understand that the information above will be published within the journal article, if accepted, and that failure to comply and/or to accurately and completely report the potential financial conflicts of interest could lead to the following: 1) Prior to publication, article rejection, or 2) Post-publication, sanctions ranging from, but not limited to, issuing a correction, reporting the inaccurate information to the authors' institution, banning authors from submitting work to ASN journals for varying lengths of time, and/or retraction of the published work.

Name: Nicole Van De Kar
Manuscript ID: CJASN-2025-001913R1
Manuscript Title: Pegcetacoplan for Adolescents With C3 Glomerulopathy or Primary Immune Complex Membranoproliferative Glomerulonephritis: Phase 3 VALIANT Subgroup Analysis
Date of Completion: February 20, 2026
Disclosure Updated Date: February 20, 2026

ASN American Society of Nephrology | JASN Journal of the American Society of Nephrology | KIDNEY360 Accessing Our World From Every Angle | CJASN Clinical Journal of the American Society of Nephrology

ASN Journal Disclosure Form

As per ASN journal policy, I have disclosed any financial relationships or commitments I have held in the past 36 months as included below. I have listed my Current Employer below to indicate there is a relationship requiring disclosure. If no relationship exists, my Current Employer is not listed.

M. Vivarelli reports the following:
Employer: Bambino Gesù Children Hospital IRCCS; Consultancy: Novartis Pharmaceuticals; SOBI Pharmaceuticals; Travere Pharmaceuticals; Roche Pharmaceuticals; Apellis Pharmaceuticals, Alexion Pharmaceuticals, BioCryst Pharmaceuticals, PureSpring Pharmaceuticals, Santhera, Takeda Pharmaceuticals, Vertex Pharmaceuticals.; Research Funding: Alexion, Bayer, Novartis, Roche, Chinook, Apellis, Travere for participation as local PI in clinical studies; Advisory or Leadership Role: SOBI Pharmaceuticals, Novartis Pharmaceuticals; Speakers Bureau: Novartis, SOBI, Alexion, Roche, Travere, WebMD, Cambridge Medical; and Other Interests or Relationships: Co-Chair, European Rare Kidney diseases Network (ERKNet) Working Group on Thrombotic Microangiopathies; Chair, KDIGO Controversy Meeting on Complement in kidney diseases; Member, International Society of Glomerular Disease Executive committee; Member, European Rare Kidney diseases Network (ERKNet) Registry Executive Committee. Editorial Board, Kidney International, JASN, Pediatric Nephrology; Member, Italian Ministry of Health Superior Council on Health and Italian Pediatric Ethics Committee.

I understand that the information above will be published within the journal article, if accepted, and that failure to comply and/or to accurately and completely report the potential financial conflicts of interest could lead to the following: 1) Prior to publication, article rejection, or 2) Post-publication, sanctions ranging from, but not limited to, issuing a correction, reporting the inaccurate information to the authors' institution, banning authors from submitting work to ASN journals for varying lengths of time, and/or retraction of the published work.

Name: Marina Vivarelli
Manuscript ID: CJASN-2025-001913R1
Manuscript Title: Pegcetacoplan for Adolescents With C3 Glomerulopathy or Primary Immune Complex Membranoproliferative Glomerulonephritis: Phase 3 VALIANT Subgroup Analysis
Date of Completion: February 24, 2026
Disclosure Updated Date: December 23, 2025

ASN American Society of Nephrology | JASN Journal of the American Society of Nephrology | KIDNEY360 Accessing Our World From Every Angle | CJASN Clinical Journal of the American Society of Nephrology

ASN Journal Disclosure Form

As per ASN journal policy, I have disclosed any financial relationships or commitments I have held in the past 36 months as included below. I have listed my Current Employer below to indicate there is a relationship requiring disclosure. If no relationship exists, my Current Employer is not listed.

D. Wallace reports the following:
Employer: Royal Manchester Children's Hospital; Consultancy: Sobi/Appellis: Received consultancy fees for producing video material and interviews on C3GN/IC-MPGN and novel complement therapies.; Honoraria: As above - for production of educational materials.; and Advisory or Leadership Role: National KQuip leader for BAPN (unpaid); Served on medical experts advisory board for Pegcetacoplan in C3GN/IC-MPGN during 4th NICE TA appraisal applications in the UK.; Served on UKKA expert group for improving access to home dialysis therapies - paid position.

I understand that the information above will be published within the journal article, if accepted, and that failure to comply and/or to accurately and completely report the potential financial conflicts of interest could lead to the following: 1) Prior to publication, article rejection, or 2) Post-publication, sanctions ranging from, but not limited to, issuing a correction, reporting the inaccurate information to the authors' institution, banning authors from submitting work to ASN journals for varying lengths of time, and/or retraction of the published work.

Name: Dean Wallace
Manuscript ID: CJASN-2025-001913R3
Manuscript Title: Pegcetacoplan for Adolescents With C3 Glomerulopathy or Primary Immune Complex Membranoproliferative Glomerulonephritis: Phase 3 VALIANT Subgroup Analysis.
Date of Completion: May 3, 2026
Disclosure Updated Date: May 3, 2026

ASN American Society of Nephrology
JASN Journal of the American Society of Nephrology
KIDNEY360 Accessing Our World From Every Angle
CJASN Clinical Journal of the American Society of Nephrology

ASN Journal Disclosure Form

As per ASN journal policy, I have disclosed any financial relationships or commitments I have held in the past 36 months as included below. I have listed my Current Employer below to indicate there is a relationship requiring disclosure. If no relationship exists, my Current Employer is not listed.

Z. Wang reports the following:
Employer: Apellis Pharmaceuticals, Inc.; and Ownership Interest: Apellis Pharmaceuticals, Inc.

I understand that the information above will be published within the journal article, if accepted, and that failure to comply and/or to accurately and completely report the potential financial conflicts of interest could lead to the following: 1) Prior to publication, article rejection, or 2) Post-publication, sanctions ranging from, but not limited to, issuing a correction, reporting the inaccurate information to the authors' institution, banning authors from submitting work to ASN journals for varying lengths of time, and/or retraction of the published work.

Name: Zhongshen Wang
Manuscript ID: CJASN-2025-001913R3
Manuscript Title: Pegcetacoplan for Adolescents With C3 Glomerulopathy or Primary Immune Complex Membranoproliferative Glomerulonephritis: Phase 3 VALIANT Subgroup Analysis.
Date of Completion: April 26, 2026
Disclosure Updated Date: April 26, 2026
