# Supplementary material for: Pegcetacoplan for Adolescents with C3 Glomerulopathy or Primary Immune Complex Membranoproliferative GN: Phase 3 VALIANT Subgroup Analysis
Source: Clin J Am Soc Nephrol. 2026 May 14;21(8):1404–13. doi: 10.2215/CJN.0000001077 (PMC13493029; doi:10.2215/CJN.0000001077)
Supplement: Supplementary file 2 [file cjasn-21-1404-s002.pdf]

**Supplemental Material**

**Supplemental Material**

This article contains the following supplemental material online.

**Supplemental Methods**

**Supplemental Table 1.** VALIANT key inclusion and exclusion criteria

**Supplemental Table 2.** Treatment-emergent adverse events according to system organ class and preferred term among adolescent patients

**Supplemental Figure 1.** VALIANT study design

**Supplemental Figure 2.** Growth during the VALIANT randomized controlled period

**Supplemental Figure 3.** Blood pressure during VALIANT randomized controlled period

## Supplemental Methods

Biopsies were required during screening or within 28 weeks of randomization for the assessment of inclusion criteria and end points for adult patients in VALIANT. Due to the invasive nature of kidney biopsies and the risks of repeat biopsies,[1] biopsies within 28 weeks of randomization were requested, if permitted by the patients' country and site, but were not required for adolescents. For adolescents, if a biopsy was not available within 28 weeks, study eligibility was assessed with a historical biopsy substantiating the diagnosis of C3G or primary IC-MPGN (even if completed >28 weeks before randomization) with alternative clinical evidence of active kidney disease at baseline based on clinical criteria (**Table S1**). Participants initially screened as adolescents followed the procedures for adolescents throughout the duration of the study, even if they turned 18 years of age during the trial.

## Reference



Confidential - Not for Public Consumption or Distribution

1. Kidney Disease: Improving Global Outcomes (KDIGO) Glomerular Diseases Work Group. KDIGO 2021 clinical practice guideline for the management of glomerular diseases. *Kidney Int.* 2021;100(suppl 4):S1–S276. doi:10.1016/j.kint.2021.05.021



Confidential - Not for Public Consumption or Distribution

**Supplemental Table 1. VALIANT key inclusion and exclusion criteria**

| **Inclusion criteria** |
|---|
| Adolescents (12-17 years) weighing ≥30 kg (where approved) |
| Adults (≥18 years) |
| Primary, biopsy-diagnosed C3G or primary IC-MPGN (with or without prior kidney transplant) |
| Evidence of active kidney disease based on ≥1 of the following at screening: |
| • Adults or adolescents with baseline (≤28 weeks before randomization) kidney biopsy |
| ○ C3 staining ≥2+ on baseline kidney biopsy |
| • Adolescents diagnosed using a kidney biopsy collected >28 weeks before randomization |
| ○ Plasma C5b-9 concentration >ULN |
| ○ Serum C3 <LLN |
| ○ Active urine sediment (hematuria with ≥5 RBCs/HPF and/or RBC casts based on local or central microscopic analysis) |
| ○ C3 nephritic factors within 6 months based on central laboratory analysis or medical history |
| Global glomerulosclerosis or interstitial fibrosis <50% on baseline kidney biopsy (if available) |
| Proteinuria ≥1 g/day and UPCR ≥1 g/g in ≥2 FMU samples |
| eGFR[a] ≥30 mL/min/1.73 $m^2$ |
| Stable (≥12 weeks prior to randomization) and optimized[b] treatment including the following: |
| • ACE inhibitors, ARBs, and/or SGLT2 inhibitors |
| • MMF (optional) |
| • Prednisone ≤20 mg/day (or equivalent dosage of other corticosteroids; optional) |
| *Streptococcus pneumoniae*, *Neisseria meningitidis* (types A, C, W, Y, and B), and *Haemophilus influenzae* (type B) vaccination ≥14 days prior to randomization[c] |
| **Exclusion criteria** |
| Weight >100 kg or < 30 kg |
| Secondary C3G or IC-MPGN[b] |
| Evidence of kidney transplant rejection requiring treatment based on baseline kidney biopsy |



Confidential - Not for Public Consumption or Distribution

| |
|---|
| Significant other kidney disease that would confound study results[b] |
| Evidence of improving kidney disease (>30% eGFR increase or >50% proteinuria decrease) within 8 weeks of or during screening |
| ANC <1000 cells/mm$^3$ |
| Previous exposure to pegcetacoplan, rituximab, belimumab, or any FDA-approved or investigational anticomplement therapy within 5 half-lives prior to screening |
| Current or prior HIV, hepatitis B, or hepatitis C infection |
| Malignancy, except for the following:<br>• Cured basal or squamous cell skin cancer<br>• Curatively treated in situ disease<br>• Malignancy free and off treatment for ≥5 years |
| History of meningococcal disease or severe infection within 14 days prior to the first dose of study drug |

ACE, angiotensin-converting enzyme; ACIP, Advisory Committee on Immunization Practices; ARB, angiotensin receptor blocker; ANC, absolute neutrophil count;; C3G, C3 glomerulopathy; CKD-EPI, Chronic Kidney Disease–Epidemiology Collaboration; eGFR, estimated glomerular filtration rate; FDA, US Food and Drug Administration; FMU, first-morning urine; HIV, human immunodeficiency virus; HPF, high-power field; IC-MPGN, immune complex membranoproliferative glomerulonephritis; LLN, lower limit of normal; MMF, mycophenolate mofetil; RBC, red blood cell; sC5b-9, soluble C5b-9; SGLT2, sodium-glucose cotransporter 2; ULN, upper limit of normal; UPCR, urine protein-to-creatinine ratio.

[a] Calculated by the bedside Schwartz equation for adolescents and the Chronic Kidney Disease Epidemiology Collaboration equation for adults.

[b] According to the investigators.

[c] Per ACIP recommendations for adults or children with complement deficiencies, unless documented evidence exists that the participant is a nonresponder to vaccination.



Confidential - Not for Public Consumption or Distribution

**Supplemental Table 2. Treatment-emergent adverse events according to system organ class and preferred term among adolescent patients[a]**

| Patients, n (%) | Pegcetacoplan (*n*=28) | Placebo (*n*=27) |
|---|---|---|
| General disorders and administration site conditions | 15 (54) | 15 (56) |
| Pyrexia | 7 (25) | 6 (22) |
| Fatigue | 2 (7) | 0 |
| Infusion site erythema | 2 (7) | 0 |
| Injection site pain | 2 (7) | 3 (11) |
| Injection site swelling | 2 (7) | 3 (11) |
| Malaise | 2 (7) | 1 (4) |
| Edema | 2 (7) | 1 (4) |
| Peripheral edema | 2 (7) | 3 (11) |
| Vaccination site pain | 2 (7) | 0 |
| Application site pain | 1 (4) | 0 |
| Infusion site bruising | 1 (4) | 0 |
| Infusion site pruritus | 1 (4) | 0 |
| Infusion site reaction | 1 (4) | 0 |
| Injection site bruising | 1 (4) | 0 |
| Injection site erythema | 1 (4) | 0 |
| Injection site induration | 1 (4) | 1 (4) |
| Injection site pruritus | 1 (4) | 0 |
| Injection site reaction | 1 (4) | 0 |
| Thirst | 1 (4) | 0 |
| Administration site pain | 0 | 1 (4) |
| Asthenia | 0 | 1 (4) |



Confidential - Not for Public Consumption or Distribution

| | | |
|---|---|---|
| Chills | 0 | 1 (4) |
| Face edema | 0 | 1 (4) |
| Infusion site discomfort | 0 | 1 (4) |
| Infusion site hemorrhage | 0 | 1 (4) |
| Infusion site pain | 0 | 1 (4) |
| Infusion site swelling | 0 | 1 (4) |
| Peripheral swelling | 0 | 1 (4) |
| Puncture site pain | 0 | 1 (4) |
| Vaccination site reaction | 0 | 1 (4) |
| Infections and infestations | 13 (46) | 13 (48) |
| Nasopharyngitis | 7 (25) | 4 (15) |
| Influenza | 3 (11) | 1 (4) |
| Body tinea | 1 (4) | 0 |
| COVID-19 | 1 (4) | 1 (4) |
| Ear infection | 1 (4) | 0 |
| Impetigo | 1 (4) | 0 |
| Myringitis | 1 (4) | 0 |
| Oral candidiasis | 1 (4) | 0 |
| Pharyngitis | 1 (4) | 2 (7) |
| Respiratory tract infection | 1 (4) | 0 |
| Viral diarrhea | 1 (4) | 0 |
| Bronchitis | 0 | 1 (4) |
| Gastroenteritis | 0 | 2 (7) |
| Herpes zoster | 0 | 2 (7) |
| Oral herpes | 0 | 1 (4) |
| Pharyngotonsillitis | 0 | 1 (4) |
| Pneumonia | 0 | 1 (4) |
| Sinusitis | 0 | 1 (4) |



Confidential - Not for Public Consumption or Distribution

| | | |
|---|---|---|
| Sinusitis bacterial | 0 | 1 (4) |
| Upper respiratory tract infection | 0 | 3 (11) |
| Viral infection | 0 | 2 (7) |
| GI disorders | 12 (43) | 6 (22) |
| Abdominal pain | 4 (14) | 1 (4) |
| Nausea | 4 (14) | 2 (7) |
| Vomiting | 4 (14) | 3 (11) |
| Upper abdominal pain | 2 (7) | 2 (7) |
| Abdominal distension | 1 (4) | 0 |
| Diarrhea | 1 (4) | 2 (7) |
| Dyspepsia | 1 (4) | 0 |
| Hematochezia | 1 (4) | 0 |
| Impaired gastric emptying | 1 (4) | 0 |
| Oral pain | 0 | 1 (4) |
| Respiratory, thoracic and mediastinal disorders | 7 (25) | 4 (15) |
| Cough | 5 (18) | 1 (4) |
| Oropharyngeal pain | 3 (11) | 0 |
| Rhinorrhea | 2 (7) | 0 |
| Sneezing | 1 (4) | 0 |
| Nasal congestion | 0 | 1 (4) |
| Allergic rhinitis | 0 | 1 (4) |
| Upper respiratory tract irritation | 0 | 1 (4) |
| Skin and subcutaneous tissue disorders | 7 (25) | 5 (19) |
| Alopecia | 1 (4) | 0 |
| Dermatitis | 1 (4) | 0 |
| Erythema | 1 (4) | 0 |
| Night sweats | 1 (4) | 1 (4) |
| Pruritus | 1 (4) | 0 |



Confidential - Not for Public Consumption or Distribution

| | | |
|---|---|---|
| Maculopapular rash | 1 (4) | 0 |
| Seborrheic dermatitis | 1 (4) | 1 (4) |
| Skin discoloration | 1 (4) | 0 |
| Skin striae | 1 (4) | 0 |
| Acne | 0 | 1 (4) |
| Dry skin | 0 | 1 (4) |
| Vitiligo | 0 | 1 (4) |
| Eye disorders | 5 (18) | 5 (19) |
| Abnormal sensation in eye | 1 (4) | 0 |
| Eye irritation | 1 (4) | 0 |
| Eye edema | 1 (4) | 1 (4) |
| Eye pain | 1 (4) | 0 |
| Periorbital dermatitis | 1 (4) | 0 |
| Visual impairment | 1 (4) | 1 (4) |
| Dry eye | 0 | 1 (4) |
| Eye swelling | 0 | 1 (4) |
| Papilledema | 0 | 1 (4) |
| Nervous system disorders | 5 (18) | 7 (26) |
| Headache | 5 (18) | 6 (22) |
| Migraine | 1 (4) | 1 (4) |
| Paraesthesia | 1 (4) | 0 |
| Presyncope | 1 (4) | 0 |
| Ageusia | 0 | 1 (4) |
| Anosmia | 0 | 1 (4) |
| Dizziness | 0 | 3 (11) |
| Injury, poisoning and procedural complications | 4 (14) | 3 (11) |
| Contusion | 2 (7) | 1 (4) |
| Immunization reaction | 1 (4) | 0 |



Confidential - Not for Public Consumption or Distribution

| | | |
|---|---|---|
| Road traffic accident | 1 (4) | 0 |
| Infusion-related reaction | 0 | 1 (4) |
| Joint dislocation | 0 | 1 (4) |
| Thermal burn | 0 | 1 (4) |
| Metabolism and nutrition disorders | 4 (14) | 1 (4) |
| Hypokalemia | 2 (7) | 0 |
| Vitamin D deficiency | 2 (7) | 0 |
| Hypoglycemia | 1 (4) | 0 |
| Impaired fasting glucose | 1 (4) | 0 |
| Decreased appetite | 0 | 1 (4) |
| Hypoalbuminemia | 0 | 1 (4) |
| Musculoskeletal and connective tissue disorders | 4 (14) | 3 (11) |
| Pain in extremity | 2 (7) | 0 |
| Arthralgia | 1 (4) | 1 (4) |
| Limb mass | 1 (4) | 0 |
| Myalgia | 1 (4) | 0 |
| Groin pain | 0 | 1 (4) |
| Muscle spasms | 0 | 1 (4) |
| Muscle twitching | 0 | 1 (4) |
| Ear and labyrinth disorders | 3 (11) | 1 (4) |
| Ear pain | 2 (7) | 0 |
| External ear inflammation | 1 (4) | 0 |
| Tympanic membrane perforation | 1 (4) | 0 |
| Middle ear effusion | 0 | 1 (4) |
| Investigations | 3 (11) | 7 (26) |
| Blood CPK increased | 1 (4) | 2 (7) |
| GFR decreased | 1 (4) | 0 |
| Intraocular pressure increased | 1 (4) | 0 |



Confidential - Not for Public Consumption or Distribution

| | | |
|---|---|---|
| SARS-CoV-2 test positive | 1 (4) | 1 (4) |
| Blood bicarbonate decreased | 0 | 1 (4) |
| Blood cholesterol increased | 0 | 1 (4) |
| Blood creatinine increased | 0 | 1 (4) |
| Blood LDH increased | 0 | 1 (4) |
| Blood phosphorus increased | 0 | 1 (4) |
| Blood potassium increased | 0 | 1 (4) |
| Liver function test increased | 0 | 1 (4) |
| Neutrophil count increased | 0 | 2 (7) |
| Weight decreased | 0 | 1 (4) |
| Renal and urinary disorders | 3 (11) | 5 (19) |
| Acute kidney injury | 2 (7) | 2 (7) |
| Urinary incontinence | 1 (4) | 0 |
| Nephrotic syndrome | 0 | 1 (4) |
| Proteinuria | 0 | 2 (7) |
| Vascular disorders | 3 (11) | 2 (7) |
| Hypertension | 3 (11) | 2 (7) |
| Blood and lymphatic system disorders | 2 (7) | 2 (7) |
| Lymphadenopathy | 1 (4) | 1 (4) |
| Neutropenia | 1 (4) | 0 |
| Anemia | 0 | 1 (4) |
| Cardiac disorders | 2 (7) | 0 |
| Left ventricular failure | 1 (4) | 0 |
| Tricuspid valve incompetence | 1 (4) | 0 |
| Pregnancy, puerperium and perinatal conditions | 0 | 1 (4) |
| Spontaneous abortion | 0 | 1 (4) |
| Pregnancy | 0 | 1 (4) |
| Psychiatric disorders | 0 | 4 (15) |



Confidential - Not for Public Consumption or Distribution

| | | |
|---|---|---|
| Anxiety | 0 | 1 (4) |
| Depression | 0 | 1 (4) |
| Irritability | 0 | 1 (4) |
| Sleep disorder | 0 | 1 (4) |

COVID-19, coronavirus disease 2019; CPK, creatine phosphokinase; GFR, glomerular filtration rate; GI, gastrointestinal; LDH, lactate dehydrogenase; SARS-CoV-2, severe acute respiratory syndrome coronavirus 2.

[a] Adolescent subgroup of safety set defined as all adolescent patients who received ≥1 dose of study treatment.



Confidential - Not for Public Consumption or Distribution

**Supplemental Figure 1. VALIANT study design**

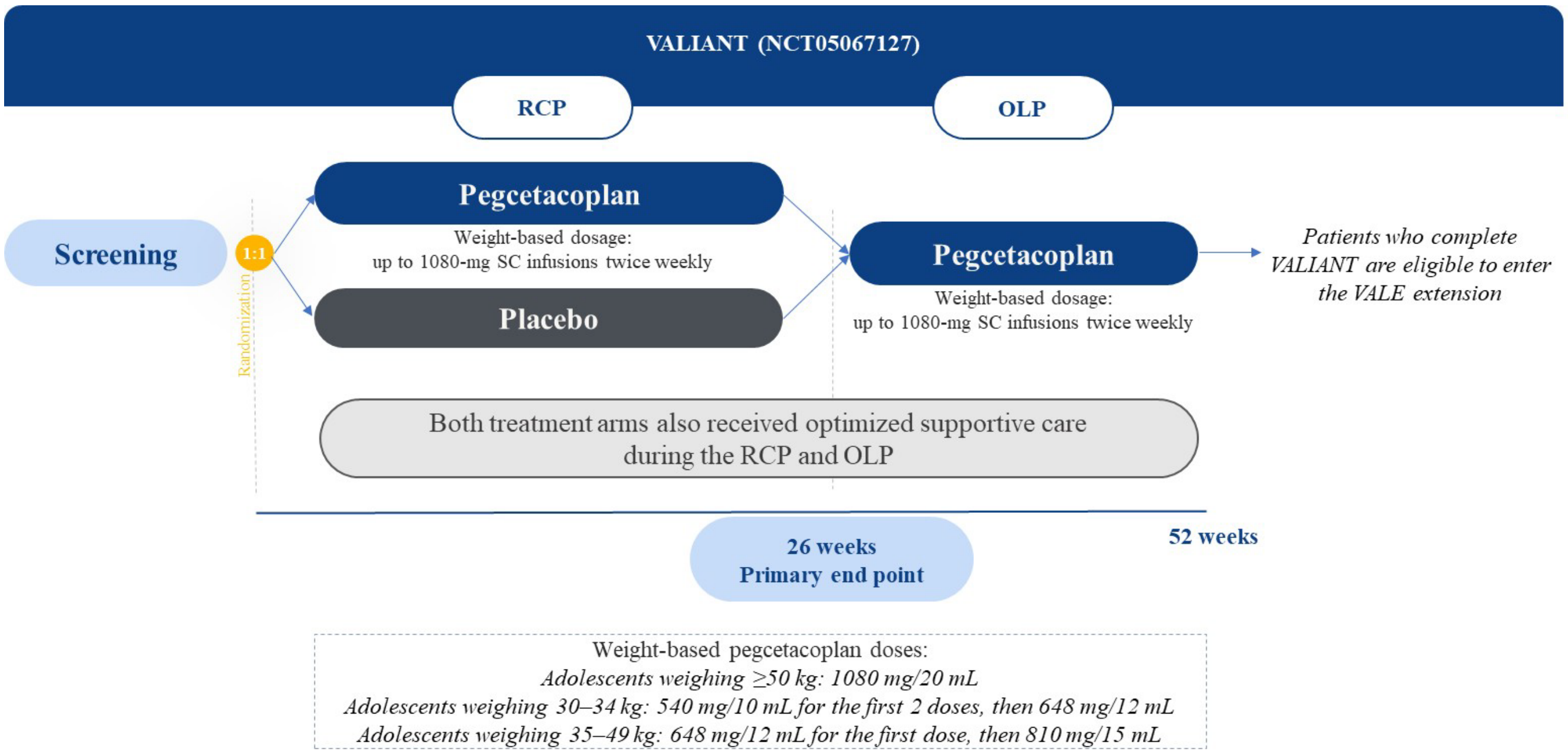


OLP, open-label period; RCP, randomized controlled period; SC, subcutaneous.



Confidential - Not for Public Consumption or Distribution

**Supplemental Figure 2. Growth during the VALIANT randomized controlled period**

**a) Height of adolescent patients according to treatment group**

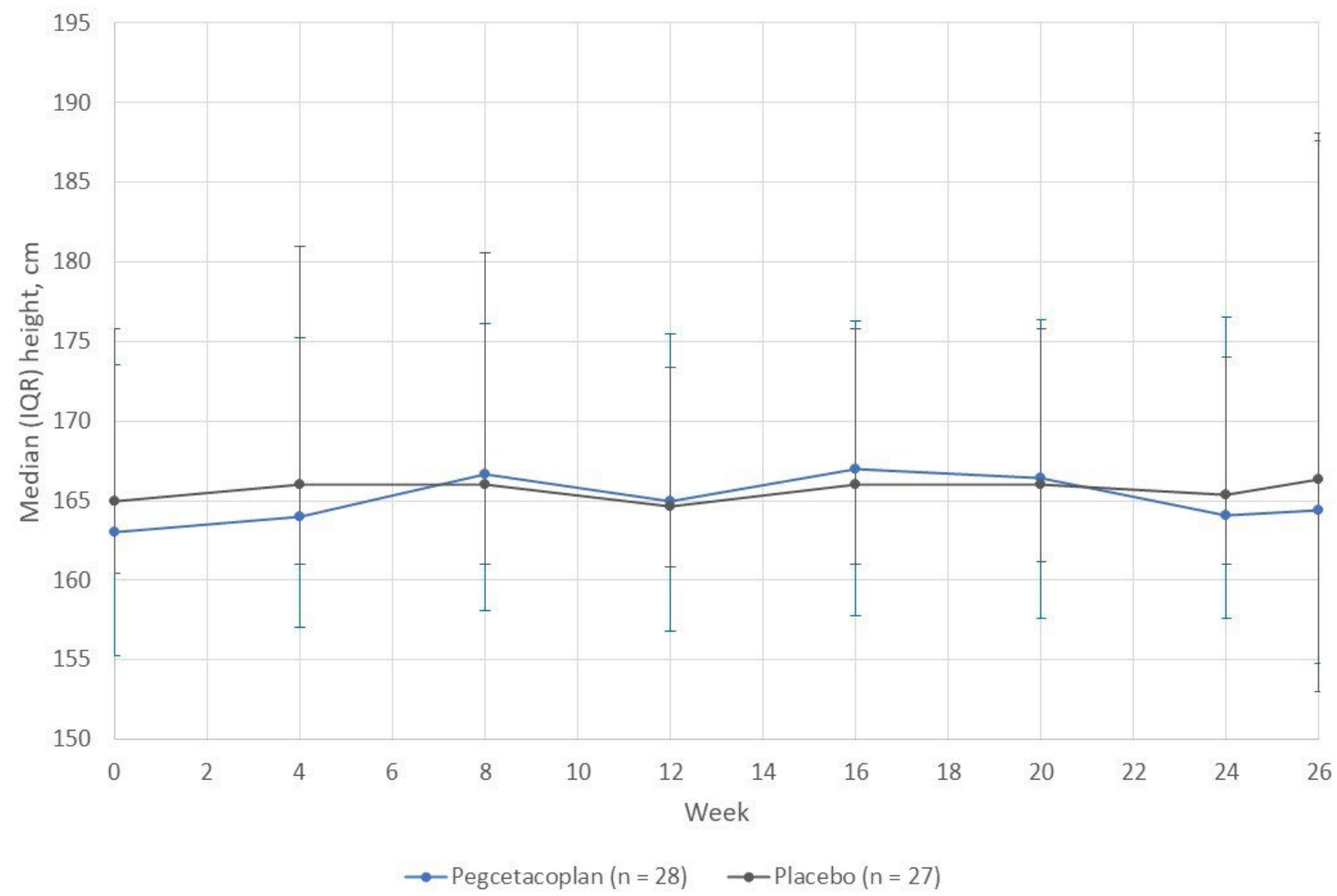


| | | | | | | | |
|---|---|---|---|---|---|---|---|
| n = 28 | 19 | 20 | 26 | 21 | 21 | 26 | 19 |
| n = 27 | 23 | 23 | 26 | 22 | 23 | 26 | 23 |

**b) Weight of adolescent patients according to treatment group**

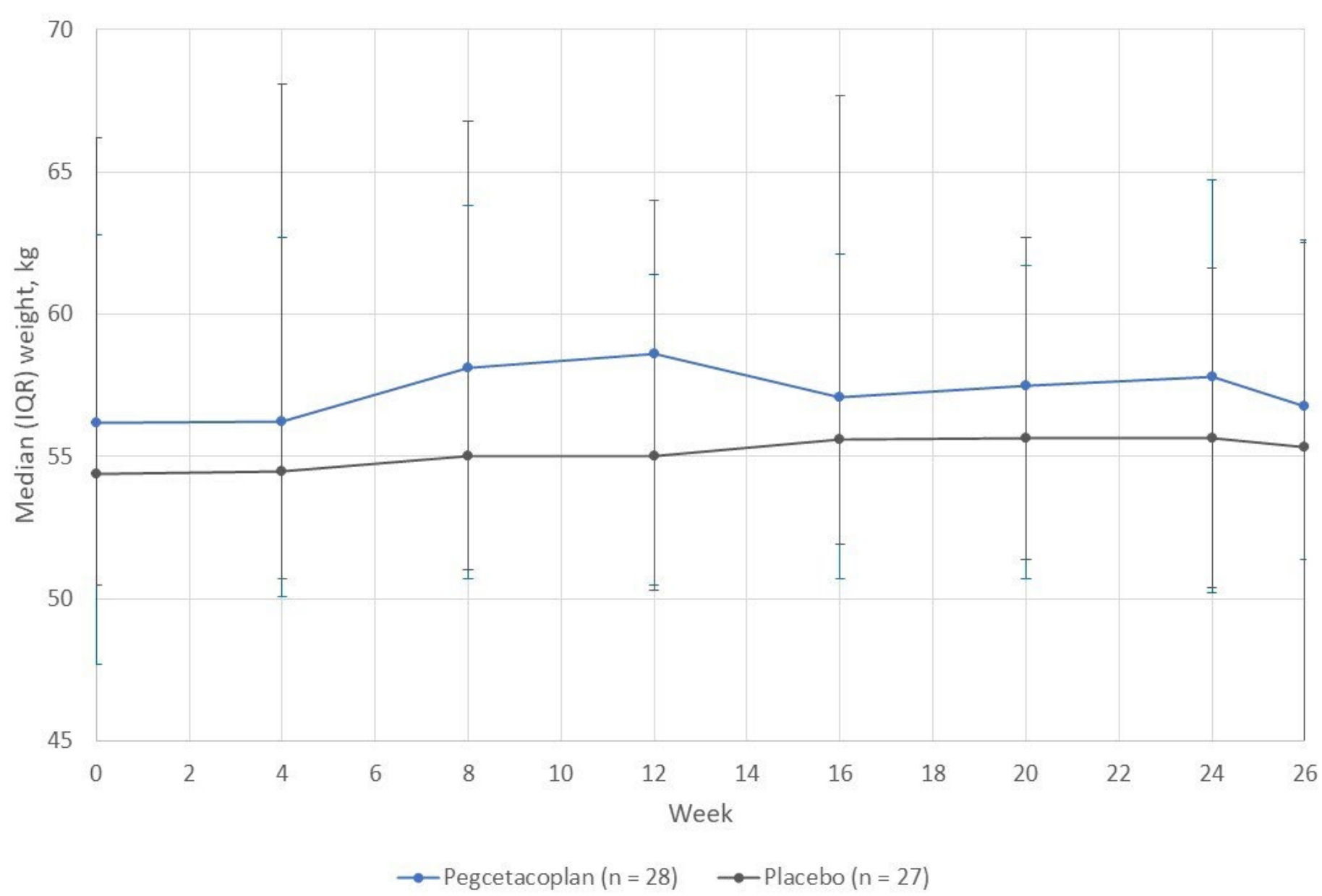


| | | | | | | | |
|---|---|---|---|---|---|---|---|
| n = 28 | 26 | 27 | 27 | 27 | 27 | 27 | 26 |
| n = 27 | 27 | 27 | 26 | 27 | 26 | 26 | 26 |



Confidential - Not for Public Consumption or Distribution

**Supplemental Figure 3. Blood pressure during VALIANT randomized controlled period**

a) **Systolic blood pressure of adolescent patients according to treatment group**

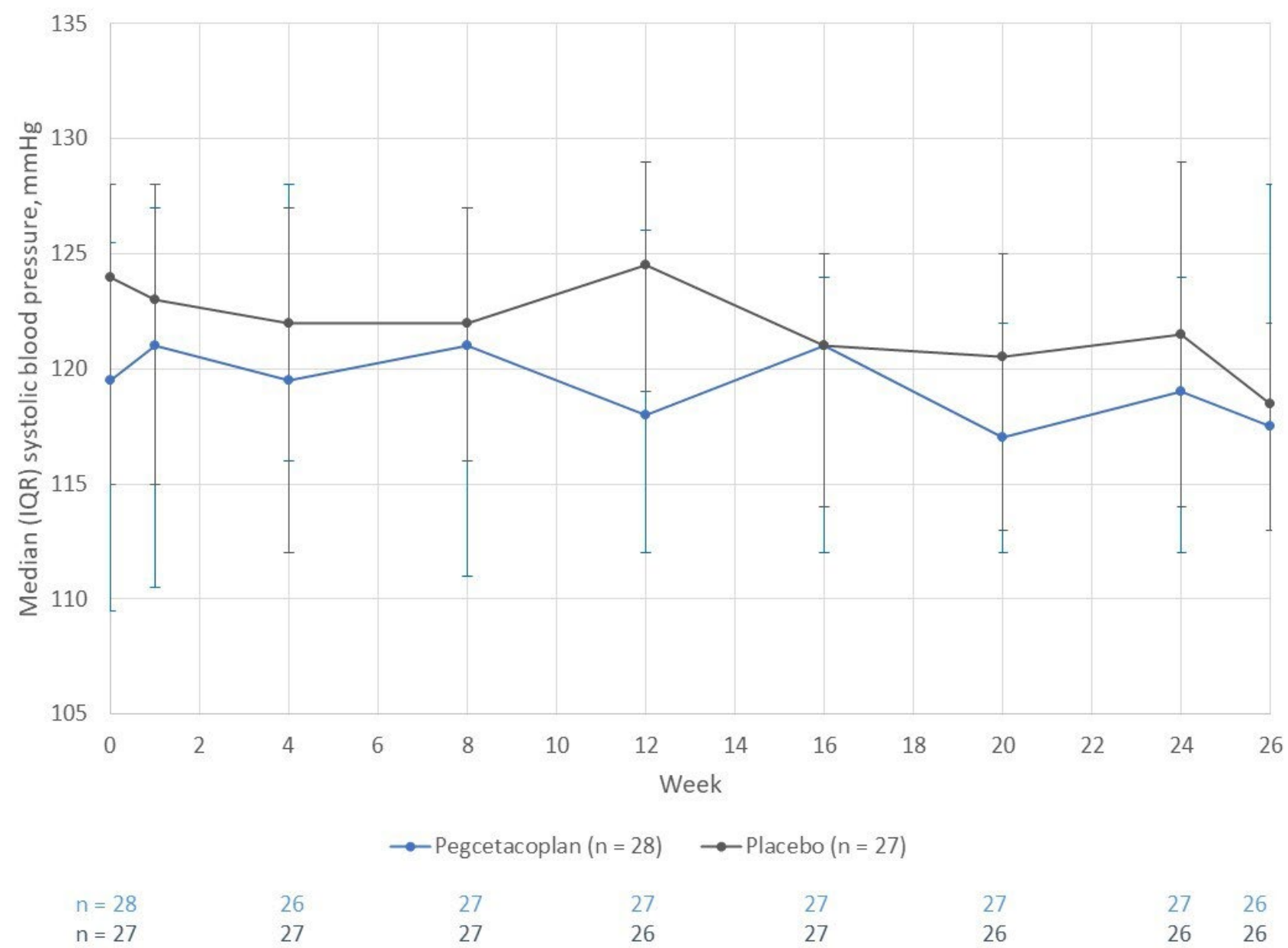


b) **Diastolic blood pressure of adolescent patients according to treatment group**

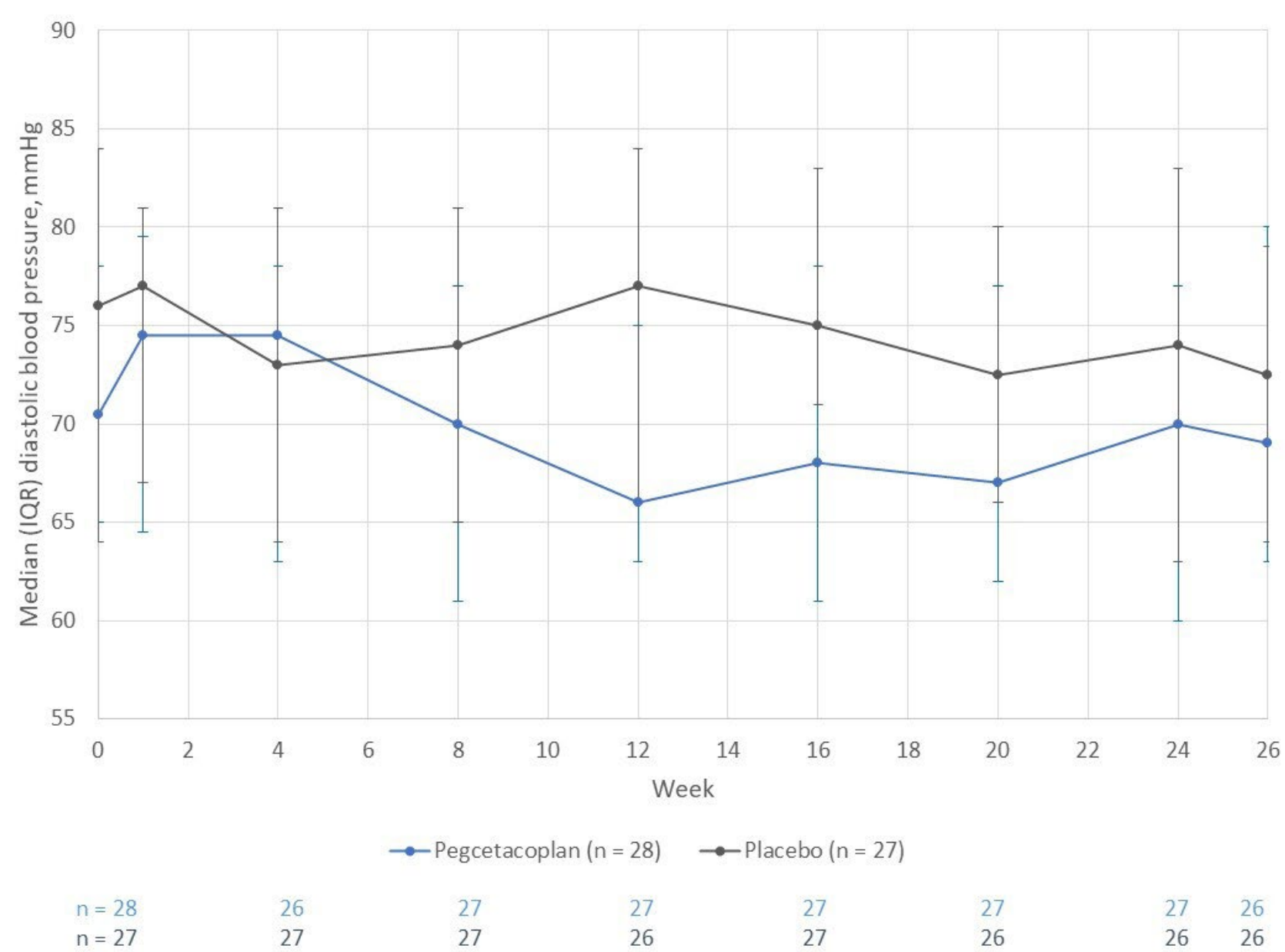




Confidential - Not for Public Consumption or Distribution

This supplement contains the following items:

1. Original protocol, final protocol, summary of changes.

2. Original statistical analysis plan, final statistical analysis plan, summary of changes

# PEGCETACOPLAN (APL-2)

# APL2-C3G-310

# A PHASE 3, RANDOMIZED, MULTICENTER, OPEN-LABEL, CONTROLLED STUDY TO EVALUATE THE EFFICACY AND SAFETY OF APL-2 IN SUBJECTS WITH C3G OR IC-MPGN

**Regulatory Agency Identifier Number(s)**

IND: 136409

EudraCT:

## SIGNATURE PAGE

**Sponsor's Approval**

The protocol has been approved by Apellis Pharmaceuticals, Inc..

**Responsible Medical Director:**

| | |
|---|---|
| Company/Sponsor signatory | Date |

# INVESTIGATOR'S AGREEMENT

I have received and read the Investigator's Brochure for Pegcetacoplan (APL-2). I have read the APL2-C3G-310 Protocol and agree to conduct the study as outlined. I agree to maintain the confidentiality of all information received or developed in connection with this protocol.

______________________________________

Printed Name of Investigator

______________________________________

Signature of Investigator

___________________

Date

# 2. SYNOPSIS

**Name of Sponsor/Company:**
Apellis Pharmaceuticals, Inc.

**Name of Investigational Product:**
Pegcetacoplan (APL-2)

**Name of Active Ingredient:**
Pegcetacoplan (APL-2)

**Protocol Number:** APL2-C3G-310

**Title of Study:**
A Phase 3, Randomized, Multicenter, Open-Label, Controlled Study to Evaluate the Efficacy and Safety of APL-2 in Subjects with C3G or IC-MPGN

**Objectives:**

**Primary:**
To assess the efficacy of twice weekly SC doses of APL-2 1080 mg compared to no APL-2 treatment in subjects with C3G/IC-MPGN, using proteinuria, as measured by uPCR

**Secondary:**

- To assess the effect of APL-2 on C3G/IC-MPGN disease parameters
- To assess the safety of APL-2 through incidence of treatment-emergent adverse events (TEAEs)

**Endpoints:**

**Primary:**
Change from baseline in uPCR at Week 26 in subjects treated with APL-2 relative to subjects receiving no APL2 treatment.

**Secondary Efficacy Endpoints:**

- The proportion of patients who achieve at least a 30% reduction in uPCR from baseline at Week 26.
- The number and proportion of subjects with normalization of serum albumin above the lower limit of normal at Week 26, for those in whom the parameter was below the lower limit of normal at baseline
- The proportion of patients who achieve at least a 50% reduction in uPCR from baseline at Week 26
- The change from baseline in uPCR at Week 52
- The durability from Week 26 in uPCR reduction at Week 52 for patients randomized to the APL-2 arm
- The change from historical eGFR (up to 3 years prior to screening) at baseline relative to the change from baseline in eGFR at Week 52
- The proportion of patients showing a change in C3c staining on renal biopsy from baseline to Week 26 in those patients with renal biopsies at baseline and Week 26
- The change from Week 26 in uPCR at Week 52

**Safety Endpoints:**

- Incidence and severity of TEAEs
- Change from baseline in vital signs and clinical laboratory tests
- Long term safety and tolerability of APL-2
- Number and incidence of rejection episodes (post-transplant patients only)

**Study Design:**

**Methodology:**
This is a Phase 3, randomized, open-label, multicenter, controlled study to evaluate the safety and efficacy of

subcutaneous (SC) APL-2 (1080 mg twice weekly) and will be conducted in approximately 90 subjects diagnosed -with C3G or IC-MPGN.

The planned duration of participation in the study for each subject is up to approximately 84 weeks. The study will consist of 4 parts:

- Part 1: 8-week Screening Period
- Part 2: 26-week Randomized Treatment Period
- Part 3: 26-week Continuation Period
- Part 4: 24-week Follow-up Period (only for those patients who do not roll into the planned long-term extension study)

**Part 1**

<u>Screening Period (approximately 8 weeks)</u>

- Informed consent will be obtained at screening prior to the conduct of any study-related procedures
- Subjects will be screened to confirm subject selection criteria are met
- A renal biopsy will be performed to confirm diagnosis and eligibility, and to serve as the baseline renal biopsy. A prior renal biopsy done within 6 months of randomization may be used for eligibility confirmation, and serve as the baseline biopsy, if confirmed as adequate per central pathology review. Subjects under the age of 18 may opt out of the baseline biopsy provided they have an adequate prior renal biopsy to establish the diagnosis, even if the prior biopsy is more than 6 months prior to randomization.
- Data to calculate historical eGFR (using CKD-EPI) will be collected for 3 years prior to study entry, or since initial biopsy diagnosis, whichever is longer
- A 24-hour urine sample will be collected to establish eligibility
- Three sets of triplicate first-morning spot urines will be collected (first-morning void collected on 3 consecutive days) to establish baseline proteinuria for the primary endpoint analysis; the 3 sets must be collected over no more than 8 weeks, and one set must be within 2 weeks of Day 1.
- Vaccinations against *Streptococcus pneumoniae*, *Neisseria meningitidis* (types A, C, W, Y, and B), and *Haemophilus influenzae* (type B) are mandatory unless documented evidence exists that subjects are non-responders to vaccination. If required, vaccinations should occur at least 14 days prior to randomization.

**Part 2**

<u>Randomized Treatment Period (26 weeks)</u>

Approximately 90 subjects who meet all of the inclusion criteria and none of the exclusion criteria will be randomized 1:1 to either the "APL-2 Arm" or the "No APL-2 Arm." Safety and efficacy will be assessed. To achieve balance between the 2 arms, there will be 2 stratification factors that will be assigned a 1:1 ratio. The first stratification factor includes subjects with post-transplant recurrence followed by the second stratification factor that includes adolescent subjects (ages 12–17) who opt out of biopsies.

- **APL-2 Arm**: Subjects randomized to this arm will receive SC APL-2 at a dose of 1080 mg twice-weekly for 26 weeks.
- **No APL-2 Arm**: Subjects randomized to this arm will not receive APL-2 treatment
- Other than study drug administration, subjects in the 2 arms will undergo the same schedule, procedures and assessments during Part 2
- Every effort will be made to minimize changes to medications related to C3G/IC-MPGN disease
- At the end of Part 2, there will be a renal biopsy, which is required to advance to Part 3. Subjects under 18 years of age who opt out of the baseline biopsy will not have a Week 26 biopsy

**Part 3**

<u>Continuation Period (26 weeks)</u>

- **APL-2 Arm**: After completion of Part 2, subjects treated with APL-2 1080 mg twice weekly will be able to continue APL-2 treatment in Part 3 up to Week 52

- **No APL-2 Arm**: After completion of Part 2, subjects randomized to the No APL-2 Arm may enter Part 3 and be treated with APL-2 up to Week 52
- During Part 3, all subjects will be treated with APL-2 1080 mg twice weekly to assess durability of response and long-term safety and efficacy up to Week 52
- Every effort should be made to minimize changes to medications related to C3G/IC-MPGN disease

**Part 4**

Follow-up Period (24 weeks)

- At Week 52, if subjects would benefit from continuing to receive APL-2, as per the Investigator's opinion, they may roll over into a separate long-term extension study
- Subjects who do not enter the planned long-term extension study will enter the 24-week Follow-up Period.

**Number of Patients (planned) and Sample Size Justification:**

Approximately 90 subjects (1:1 allocation ratio to APL-2 Arm or No APL-2 Arm) will be randomized into the study.

Assuming a difference in the change from baseline of -1.30 mg/mg in uPCR at Week 26 between treatment groups with a standard deviation of 1.67 mg/mg, a total of 36 subjects per treatment group is required for 90% power at the significance level of 0.025 (one-sided) using a two-sample t-test with equal allocation to treatment groups. These assumptions regarding the difference in the change from baseline and standard deviation are based on 11 patients from the APL2-201 Phase 2 study.

A total of 90 subjects will be randomized to account for an anticipated 20% of randomized subjects prematurely discontinuing the study without providing a post-baseline uPCR measurement.

A sample size re-estimation based on the conditional power approach without adjustment of Type I error rate may be considered. The sole purpose of the planned sample size re-estimation (which may take place when approximately 50% of all patients have completed the Week 26 visit or withdrew prior to the Week 26 visit) is to adjust the sample size, which will be changed only under certain conditions (details will be provided in the interim analysis SAP). This assessment will not lead to a reduction in the sample size. The sample size of the study may be increased to a maximum of 120. An increase beyond this maximum would require a protocol amendment.

**Diagnosis:**

Patients with a diagnosis of C3G or IC-MPGN, including those with post-transplant recurrence

**Main Criteria for Inclusion:**

Inclusion Criteria

1. At least 18 years of age; where approved, adolescents (ages 12–17) weighing at least 42 kg may also be enrolled
2. A diagnosis of primary C3G or IC-MPGN with evidence of ongoing complement activation, based on central pathology and medical review of the case
3. At least 2+ staining for C3c on the baseline renal biopsy collected during screening, per the central pathology laboratory
4. No more than 50% global glomerulosclerosis or tubulointerstitial scarring on the baseline biopsy collected during screening
5. uPCR ≥ 1000 mg/g, and at least 1 g/day of proteinuria, on a screening 24-hour urine collection
6. eGFR ≥ 30 ml/min/1.73 $m^2$ calculated by the Chronic Kidney Disease -Epidemiology Collaboration (CKD-EPI) creatinine equation for adults, or the Bedside Schwartz equation for adolescents
7. Body mass index (BMI) ≤ 35 $kg/m^2$
8. Stable regimen for C3G/IC-MPGN treatment for at least 4 weeks prior to the baseline renal biopsy collected during screening, and at least 8 weeks (or 5 half-lives of medications, whichever is greater) prior to randomization
9. Have received vaccinations against *Streptococcus pneumoniae*, *Neisseria meningitidis* (types A, C, W,

Y, and B), and *Haemophilus influenzae* (type B) within 5 years prior to randomization, or agree to receive vaccinations during screening. Vaccinations should occur at least 14 days prior to randomization. Vaccination is mandatory, unless documented evidence exists that subjects are non-responders to vaccination (as evidenced by titers or display titer levels within acceptable local limits).

10. Female subjects of child-bearing potential must have a negative blood pregnancy test at screening (and negative urine pregnancy test on Day 1) and must agree to use protocol-defined methods of contraception from screening through at least 90 days after receiving the last dose of APL-2.
11. Male subjects must agree to use protocol-defined methods of contraception and agree to refrain from donating semen from screening through at least 90 days after receiving the last dose of APL-2
12. Willing and able to provide informed consent

Exclusion Criteria

1. Previous treatment with APL-2
2. Improving renal disease in the 8 weeks prior to screening, or during the Screening Period; improving renal disease is defined as > 30% increase in eGFR, or > 50% decrease in proteinuria
3. Evidence of rejection on the baseline renal biopsy collected during screening, that requires treatment, in a renal transplant patient
4. C3G/IC-MPGN secondary to another condition (eg, infection, malignancy, monoclonal gammopathy, autoimmunity, chronic antibody-mediated rejection, or a medication)
5. Current or prior diagnosis of human immunodeficiency virus (HIV), hepatitis B (HBV), or hepatitis C (HCV) infection, or positive serology during screening that is indicative of infection
6. Malignancy, except for the following:
   a. Cured basal or squamous cell skin cancer
   b. Curatively treated in situ disease
   c. Malignancy-free and off treatment for ≥ 5 years
7. Significant other renal disease that would confound interpretation of study results
8. Participation in any other investigational drug trial or exposure to other investigational agent, device or procedure within 30 days or 5 half-lives from the last dose of investigational agent (whichever is longer) prior to Screening Period
9. Use of rituximab, belimumab, or any approved or investigational anti-complement therapy other than APL 2 within 5 half-lives of that product prior to the Screening Period
10. Female subjects who are currently breastfeeding and are unwilling to discontinue for the duration of the study and for at least 90 days after the final dose of study drug
11. Inability to cooperate or any condition that, in the opinion of the Investigator, creates an undue risk for the patient by participating in the study, or is likely to confound interpretation of the study results
12. Evidence of, in the opinion of the Investigator, drug or alcohol abuse or dependence
13. Presence or suspicion of severe recurrent or chronic infections that, in the opinion of the Investigator, may place the subject at unacceptable risk by study participation
14. Presence or suspicion of liver dysfunction as indicated by elevated alanine aminotransferase (ALT), aspartate aminotransferase (AST), or bilirubin levels > 2× upper limit of normal (ULN).

**Investigational Product, Dosage and Mode of Administration:**

APL-2, 1080 mg, twice weekly, SC

**Duration of Participation:**

Total participation will be approximately 84 weeks (Screening: approximately 8 weeks; Randomized Treatment: 26 weeks; Continuation Period: 26 weeks; Follow-up: 24 weeks). Depending on randomization, treatment with APL-2 will be for a maximum of 52 weeks (Randomized Treatment: 26 weeks; Continuation Period: 26 weeks).

**Statistical Methodology:**

**Efficacy:**

Primary Efficacy:

The change from baseline in uPCR will be calculated using the following steps:

1. Baseline uPCR value will be calculated as the average of up to 9 uPCR measurements preceding and including Day 1.
2. Week 26 uPCR value will be calculated as the average of up to 9 uPCR measurements preceding and including Week 26.

The between-treatment group comparison for the primary efficacy endpoint will be performed using a mixed effect model for repeated measures (MMRM) (Mallinckrodt, 2008). The model will include fixed categorical effects for treatment group, study visit, stratification factors and the study visit-by-treatment group interaction, as well as the continuous, fixed covariate of baseline uPCR level. An unstructured covariance matrix will be used. The difference between treatment groups will be estimated, along with its 97.5% one-sided CI and corresponding p-value.

To account for missing data, sensitivity analyses will be performed and provided in the full protocol.

Secondary Efficacy:

To preserve the Type 1 error a fixed sequence testing strategy will be used. The ordering of the secondary endpoints in this testing strategy will match the order in which they are presented in Section 6.2.2.

- The proportion of patients who achieve at least a 30% reduction in uPCR will be calculated using the following steps:
    1. Baseline uPCR value will be calculated as the average of up to 9 uPCR measurements preceding and including Day 1.
    2. Week 26 uPCR value will be calculated as the average of up to 9 uPCR measurements preceding and including Week 26.
    3. Patients will be determined as either success or failures based on whether the change from baseline in uPCR calculated from steps 1 and 2 achieves at least a 30% reduction.

  The proportion of patients who achieve at least a 30% reduction in uPCR from baseline at Week 26 will be analyzed using mixed-model for repeated measures (MMRM) with the effects of treatment; visit, visit-by treatment interaction; the relevant baseline level; and the baseline level-by-visit interaction using an unstructured covariance matrix. The odds ratio of achieving at least a 30% reduction in uPCR from baseline at Week 26 for the APL-2 Arm versus the No APL-2 Arm and associated 97.5% one-sided CI will be provided.

- The number and proportion of patients with serum albumin above the lower limit of normal, for those in whom the parameter was below the lower limit of normal at baseline, will be calculated using the following steps:
    1. Baseline serum albumin value will be calculated as the average of up to 2 serum albumin measurements preceding and including Day 1. Patients will only be included in the denominator of this calculation if the baseline albumin value is below the lower limit of normal.
    2. Week 26 serum albumin value will be calculated as the average of up to 2 serum albumin measurements preceding and including Week 26.
    3. Patients will be determined as either success or failures based on whether the Week 26 serum albumin value as calculated in Step 2 is above the lower limit or normal.

  The number and proportion of patients with serum albumin above the lower limit of normal at Week 26, for those in whom the parameter was below the lower limit of normal at Baseline, will be tabulated by treatment group and compared between treatment groups using Fisher's exact test. The odds ratio of showing serum albumin above the lower limit of normal at Week 26 for the APL-2 Arm versus the No APL-2 Arm and associated 97.5% one-sided CI will be provided.

- The proportion of patients who achieve at least a 50% reduction in uPCR will be calculated using the following steps:

1. Baseline uPCR value will be calculated as the average of up to 9 uPCR measurements preceding and including Day 1.
2. Week 26 uPCR value will be calculated as the average of up to 9 uPCR measurements preceding and including Week 26.
3. Patients will be determined as either success or failures based on whether the change from baseline in uPCR calculated from steps 1 and 2 achieves at least a 50% reduction.

The proportion of patients who achieve at least a 50% reduction in uPCR from baseline at Week 26 will be analyzed using mixed-model for repeated measures (MMRM) with the effects of treatment; visit, visit-by treatment interaction; the relevant baseline level; and the baseline level-by-visit interaction using an unstructured covariance matrix. The odds ratio of showing at least a 50% reduction in uPCR from baseline at Week 26 for the APL-2 Arm versus the No APL-2 Arm and associated 97.5% one-sided CI will be provided.

- The change from baseline in uPCR at Week 52 will be calculated using the following steps:
  1. Baseline uPCR value will be calculated as the average of up to 9 uPCR measurements preceding and including Day 1.
  2. Week 52 uPCR value will be calculated as the average of up to 9 uPCR measurements preceding and including Week 52.

  The change from baseline in uPCR at Week 52 will be analyzed using a one-sample t-test (APL-2 arm). This difference will be estimated, along with its 97.5% one-sided CI and corresponding p-value.
- A matched pair t-test will be conducted to compare the durability of treatment between the uPCR reduction from baseline to Week 26 and uPCR reduction from Week 26 to Week 52. The difference between uPCR reduction at the end of the Continuation Period and uPCR reduction at the end of Randomized Treatment Period will be estimated, along with its 97.5% one-sided CI and p-value.
- A matched paired t-test will be conducted to compare the change in eGFR from historical eGFR (up to 3 years prior to screening) to baseline and change in eGFR from baseline to Week 52. The difference will be estimated, along with its 97.5% one-sided CI and p-value.
- The number and proportion of patients who show a reduction in C3c staining on renal biopsy from baseline to Week 26 will be tabulated by treatment group and compared between treatment groups using Fisher's exact test. The odds ratio of showing a reduction from baseline in C3c staining on renal biopsy at Week 26 for the APL-2 Arm versus the No APL-2 Arm and associated 97.5% one-sided CI will be provided.
- The change from Week 26 in uPCR at Week 52 will be calculated using the following steps:
  1. Week 26 uPCR value will be calculated as the average of up to 9 uPCR measurements preceding and including Week 26.
  2. Week 52 uPCR value will be calculated as the average of up to 9 uPCR measurements preceding and including Week 52.

  The change from Week 26 in uPCR at Week 52 will be analyzed using a one-sample t-test. This difference will be estimated, along with its 97.5% one-sided CI and corresponding p-value.

**Safety:**

Adverse events will be coded using the Medical Dictionary for Drug Regulatory Activities (MedDRA). All AEs, including TEAEs, will be summarized by System Organ Class, Preferred Term, treatment group for number of subjects and proportion reporting the event. A similar summary will be produced for SAEs, AEs leading to termination, severe AEs, and AEs related to the investigational product. The intensity of AEs and the relationship to investigational product will be summarized for each System Organ Class and Preferred Term by treatment group.

Withdrawals due to AEs will be summarized for each body system and Preferred Term by treatment group.

Laboratory assessments, anti-APL-2/PEG antibodies, and ECGs will be summarized by treatment group using appropriate descriptive statistics.

# 3. TABLE OF CONTENTS, LIST OF TABLES, AND LIST OF FIGURES

## TABLE OF CONTENTS

1. TITLE PAGE ....1
SIGNATURE PAGE ....1
INVESTIGATOR'S AGREEMENT ....2
2. SYNOPSIS ....3
3. TABLE OF CONTENTS, LIST OF TABLES, AND LIST OF FIGURES ....9
4. LIST OF ABBREVIATIONS AND DEFINITIONS OF TERMS ....16
5. INTRODUCTION ....19
5.1. C3G and IC-MPGN and Unmet Medical Need ....19
5.2. Study Rationale ....19
5.2.1. Justification of Study Population ....20
5.2.2. Justification of Dose ....20
5.3. Summary of Clinical Experience with APL-2 ....22
5.4. Clinical Risks/Benefits of APL-2 ....22
6. STUDY OBJECTIVES AND ENDPOINTS ....24
6.1. Study Objectives ....24
6.1.1. Primary Objective ....24
6.1.2. Secondary Objectives ....24
6.1.3. Exploratory Objectives ....24
6.2. Study Endpoints ....24
6.2.1. Primary Efficacy Endpoint ....24
6.2.2. Secondary Efficacy Endpoints ....24
6.2.3. Exploratory Endpoints ....25
6.2.4. Safety Endpoints: ....25
6.2.5. Pharmacokinetic Endpoints: ....26
6.2.6. Pharmacodynamic Endpoints: ....26
7. INVESTIGATIONAL PLAN ....27
7.1. Overall Study Design ....27
7.1.1. Part 1: Screening Period (up to 8 weeks) ....29
7.1.1.1. Rescreening ....29

7.1.2. Part 2: Randomized Treatment Period (26 weeks) ....30
7.1.3. Part 3: Continuation Period (26 weeks) ....30
7.1.4. Part 4: Follow-up Period (24 weeks) ....30
7.2. Number of Subjects ....36
7.3. Treatment Assignment ....36
7.4. Criteria for Study Termination ....36
8. SELECTION AND WITHDRAWAL OF SUBJECTS ....37
8.1. Subject Inclusion Criteria ....37
8.2. Subject Exclusion Criteria ....37
8.3. Subject Withdrawal Criteria ....38
8.3.1. Reasons for Discontinuation ....39
8.3.2. Subjects Lost to Follow-up Prior to Last Scheduled Visit ....39
9. TREATMENT OF SUBJECTS ....40
9.1. Description of Study Drug ....40
9.2. Vaccinations ....40
9.2.1. Empiric Antibiotic Treatment for Possible Infection ....40
9.3. Concomitant Medications ....40
9.3.1. Post-Transplant Immunosuppression ....41
9.3.2. Restricted and Prohibited Medications ....41
9.4. Treatment Compliance ....41
9.5. Measures to Minimize Bias: Study Treatment Assignment ....41
9.5.1. Randomization ....41
9.5.2. Blinding ....42
10. STUDY DRUG MATERIALS AND MANAGEMENT ....43
10.1. Study Drug ....43
10.2. Study Drug Packaging and Labeling ....43
10.2.1. Packaging ....43
10.2.2. Labeling ....43
10.3. Study Drug Storage ....43
10.4. Injection/Infusion Supplies ....44
10.5. APL-2 Administration ....44
10.6. Study Drug Accountability ....45

10.7. Study Drug Handling and Disposal ....45
11. ASSESSMENT OF EFFICACY ....46
11.1. Retrospective Data Collection for eGFR and Measured GFR....46
11.2. 24-Hour Urine Collection ....46
11.3. Triplicate First-Morning Spot Urine Collections ....47
11.4. Random Spot Urine Collection....47
11.5. Renal Biopsy....48
11.6. Ophthalmologic Evaluation ....48
11.7. Measured Glomerular Filtration Rate ....48
11.8. Health-Related Quality of Life Assessments....49
11.8.1. Functional Assessment of Illness Therapy Fatigue Scale....49
11.8.2. Short Form (36) Health Survey ....49
11.8.3. 5-Level EuroQol-5 Dimension ....49
11.8.4. Kidney Disease Quality of Life ....49
12. ASSESSMENT OF SAFETY....50
12.1. Safety Parameters ....50
12.1.1. Demographic/Medical History ....50
12.1.2. Central Medical Review ....50
12.1.3. Vital Signs ....50
12.1.4. Weight and Height....51
12.1.5. Physical Examination ....51
12.1.6. Menses ....51
12.1.7. Electrocardiogram (ECG)....51
12.1.8. Injection/Infusion Site/Pump Safety Assessment....52
12.1.9. Laboratory Assessments ....52
12.1.9.1. Pregnancy Screen....54
12.1.10. Pharmacokinetic Assessment....54
12.1.11. Anti-APL-2 and Anti-PEG Antibody Assessment ....54
12.2. Adverse and Serious Adverse Events ....55
12.2.1. Definition of Adverse Events ....55
12.2.1.1. Adverse Event (AE)....55
12.2.1.2. Serious Adverse Event (SAE) ....55

12.2.2. Relationship to Study Drug ....................56
12.2.3. Recording Adverse Events ....................57
12.2.4. Reporting Adverse Events ....................57
12.3. Pregnancy ....................58
12.3.1. Acceptable Methods of Contraception ....................58
13. STATISTICS ....................60
13.1. Determination of Sample Size ....................60
13.2. Analysis Set ....................60
13.2.1. Screened Set ....................60
13.2.2. Safety Set ....................60
13.2.3. Intent-to-Treat Set ....................60
13.2.4. Modified Intent-to-Treat Set ....................61
13.2.5. Per Protocol Set ....................61
13.2.6. Pharmacokinetic Set ....................61
13.2.7. Pharmacodynamic Set ....................61
13.2.8. Data Review for Analysis Set ....................61
13.3. Efficacy Analysis ....................61
13.3.1. Analysis of Primary Efficacy Endpoint ....................61
13.3.2. Analysis of Secondary Efficacy Endpoint ....................62
13.4. Safety Analysis ....................64
13.5. Pharmacokinetic Analysis ....................65
13.6. Pharmacodynamic Analysis ....................65
13.7. Other Analyses ....................65
13.8. Interim Analysis ....................65
13.9. Data Monitoring Committee ....................66
14. DIRECT ACCESS TO SOURCE DATA/DOCUMENTS ....................67
14.1. Study Monitoring ....................67
14.2. Audits and Inspections ....................67
14.3. Institutional Review Board ....................68
15. QUALITY CONTROL AND QUALITY ASSURANCE ....................69
16. ETHICS ....................70
16.1. Ethics Review ....................70

16.2. Ethical Conduct of the Study ..... 70
16.3. Written Informed Consent ..... 70
17. DATA HANDLING AND RECORDKEEPING ..... 71
17.1. Inspection of Records ..... 71
17.2. Retention of Records ..... 71
18. PUBLICATION POLICY ..... 72
19. LIST OF REFERENCES ..... 73

# LIST OF TABLES

Table 1: Schedule of Assessments ........ 31
Table 2: Urine Collection Schedule: Randomized Treatment Period ........ 35
Table 3: Laboratory Assessments ........ 53
Table 4: Definitions of Adverse Event Relatedness ........ 56
Table 5: Severity of Events ........ 56

# LIST OF FIGURES

Figure 1: Predicted APL-2 PK Concentration for Adolescent Subjects Receiving APL-2 at the Proposed Pediatric Dose Regimens ....................................................21

Figure 2: APL2-C3G-310 Study Design ........................................................................28

# 4. LIST OF ABBREVIATIONS AND DEFINITIONS OF TERMS

The following abbreviations and specialist terms are used in this study protocol.

| Abbreviation | Definition |
|---|---|
| Ab | antibodies |
| ACR | albumin-to-creatinine ratio |
| ADA | antidrug antibodies |
| ADR | adverse drug reaction |
| AE | adverse event |
| AH50 | 50% alternative hemolytic complement pathway activity |
| AIHA | autoimmune hemolytic anemia |
| ALP | alkaline phosphatase |
| ALT | alanine aminotransferase |
| ANA | anti-nuclear antibodies |
| ANCA | antineutrophil cytoplasmic antibodies |
| ANCOVA | analysis of covariance |
| AP | alternative pathway |
| ARC | absolute reticulocyte count |
| AST | aspartate aminotransferase |
| BCR | B-2 microglobulin-to-creatinine ratio |
| β-HCG | beta human chorionic gonadotropin |
| BMI | body mass index |
| BUN | blood urea nitrogen |
| C3G | complement 3 glomerulopathy |
| C3GN | C3 glomerulonephritis |
| CDC | Centers for Disease Control and Prevention |
| CDN | complement-dependent nephropathy |
| CH50 | 50% classical hemolytic complement pathway activity |
| CKD-EPI | Chronic Kidney Disease-Epidemiology Collaboration |
| $C_{max}$ | maximum drug plasma concentration |
| DDD | dense deposit disease |
| DMC | data monitoring committee |
| DNA | deoxyribonucleic acid |
| ECG | electrocardiogram |
| eCRF | electronic case report form |
| EDC | electronic data capture |
| eGFR | estimated glomerular filtration rate |

| Abbreviation | Definition |
|---|---|
| ESRD | end-stage renal disease |
| ET | early termination |
| FACIT-F | Functional Assessment of Chronic Illness Therapy- Fatigue Scale score |
| FDA | Food and Drug Administration |
| FSH | follicle stimulating hormone |
| GCP | good clinical practice |
| GFR | glomerular filtration rate |
| GGT | gamma-glutamyltransferase |
| Hb | Hemoglobin |
| HBsAg | hepatitis B surface antibody |
| HBV | hepatitis B virus |
| HCV | hepatitis C virus |
| HDL | high-density lipoproteins |
| HIV | human immunodeficiency virus |
| HRQoL | health-related quality of life |
| IB | Investigator's Brochure |
| IC-MPGN | immune complex membranoproliferative glomerulonephritis |
| ICF | informed consent form |
| ICH | International Conference on Harmonisation |
| IEC | independent ethics committee |
| Ig | Immunoglobulin |
| IND | Investigational New Drug |
| INR | international normalized ratio |
| IRB | institutional review board |
| IRT | interactive response technology |
| ITT | intent-to-treat |
| IUD | intrauterine device |
| IUS | intrauterine hormone-releasing system |
| KDQOL | Kidney Disease Quality of Life |
| LDH | lactate dehydrogenase |
| LDL | low-density lipoproteins |
| MAR | missing at random |
| MedDRA | Medical Dictionary for Regulatory Activities |
| MMRM | mixed-model for repeated measures |
| MPO | Myeloperoxidase |

| Abbreviation | Definition |
|---|---|
| NOAEL | no-observed-adverse-effect level |
| PCR | protein-to-creatinine ratio |
| PD | Pharmacodynamic |
| PEG | polyethylene glycol |
| PK | Pharmacokinetic |
| PNH | paroxysmal nocturnal hemoglobinuria |
| RBC | red blood cell |
| RNA | ribonucleic acid |
| SAE | serious adverse event |
| SAP | statistical analysis plan |
| SC | subcutaneous |
| SD-OCT | spectral domain optical coherence tomography |
| SPEP | serum protein electrophoresis |
| TEAE | treatment-emergent adverse event |
| TMDD | target-mediated drug disposition |
| uACR | urine albumin-to-creatinine ratio |
| ULN | upper limit of normal |
| uPCR | urine protein-to-creatinine ratio |
| US | United States |
| VAS | visual analog scale |
| WBC | white blood cell |
| WHO | World Health Organization |
| WOCBP | women of childbearing potential |

# 5. INTRODUCTION

Two similar diseases, complement component 3 glomerulopathy (C3G) and immune complex membranoproliferative glomerulonephritis (IC-MPGN), will be studied in this protocol. This study will include patients with native kidney disease or with post-transplant disease recurrence, and will exclude patients whose C3G or IC-MPGN is secondary to an underlying condition (eg, infection, monoclonal gammopathy, malignancy, or medication).

## 5.1. C3G and IC-MPGN and Unmet Medical Need

Component 3 glomerulopathy is a disease in which the complement system, specifically the alternative pathway (AP), is indiscriminately overactive, leading to excessive deposition of C3 breakdown products in the glomeruli of the kidney, and damage to the renal parenchyma. The underlying AP hyperactivity can be due to various factors, most commonly, an acquired autoantibody which stabilizes the AP C3 or C5 convertase (C3 or C5 nephritic factor). AP hyperactivity leads to uncontrolled C3 activation, resulting in an excess of C3 breakdown products that are deposited in the glomerular basement membrane and mesangium. This stimulates a brisk inflammatory response, disrupting the normal glomerular architecture.

There are 2 subtypes of C3G: dense deposit disease (DDD) and C3 glomerulonephritis (C3GN), which are distinguishable only by renal biopsy. The clinical presentation, features, and disease course are similar between the 2 subtypes, but with some distinctions. DDD tends to present earlier in life than C3GN (median ages of diagnosis for DDD and C3GN are 12 and 26 years old, respectively), and DDD is also associated with extra-renal manifestations, such as acquired partial lipodystrophy and macular degeneration. The incidence of C3GN is about 3-fold greater than that of DDD (Medjeral-Thomas, 2014; Servais, 2012; Lu, 2012).

Although IC-MPGN is typically considered a distinct pathologic entity from C3G, the underlying pathophysiology and clinical course of the 2 primary diseases are remarkably similar (Noris, 2018; Holle, 2018). As in C3G, patients with primary IC-MPGN often have evidence of AP hyperactivity, including the presence of genetic or acquired causes of AP dysregulation. In these patients, uncontrolled C3 activation and glomerular deposition of C3 breakdown products is also observed, similar to C3G. The only pathologic distinction from C3G is that the glomerular C3 deposition in IC-MPGN is accompanied by immunoglobulin deposition. The common clinical manifestations of primary IC-MPGN are the same as those of C3G and other glomerulonephritides – proteinuria, hematuria, hypertension, and reduced glomerular filtration rate.

## 5.2. Study Rationale

There are no therapies approved to prevent or reverse disease progression in C3G or IC-MPGN. Similar to other glomerular diseases, disease management includes non-specific measures to manage proteinuria, hypertension, hyperlipidemia, edema, and other facets of glomerular and chronic kidney disease. Despite these various measures, the prognosis of C3G and IC-MPGN is poor as patients steadily progress to end-stage renal disease (ESRD) (Smith, 2019; Regunathan-Shenk, 2019; Medjeral-Thomas, 2014; Zhang, 2014; Pickering, 2013; Lu, 2012; Okpechi, 2014).

Renal transplantation is an option for patients who reach ESRD, but the incidence of disease recurrence is high, with up to 50% of patients losing their renal allografts due to disease recurrence (Medjeral-Thomas, 2014; Zhang, 2014; Pickering, 2013; Lu, 2012). Therefore, a therapy that can protect the kidneys, native or transplanted, from ongoing damage due to complement hyperactivity is an unmet need.

As an inhibitor of C3 and its fragment, C3b, APL-2 has the potential to address the underlying disease pathophysiology of complement hyperactivity in C3G and IC-MPGN. Specifically, by inhibiting C3, it is expected that APL-2 will halt the inappropriate and excessive C3 activation that characterizes these diseases. Additionally, by inhibiting C3b, APL-2 inhibits the activity of the AP C3 convertase (whether it is stabilized by nephritic factors or not) through a second complementary mechanism of action in addition to the inhibition of C3 as a substrate in the complement cascade. These 2 complementary mechanisms will prevent further deposition of C3 breakdown products in the glomeruli, affording the opportunity for the kidney to recover by clearing existing deposits and resolving inflammation. This renal recovery would be expected, initially, to yield a reduction in proteinuria, and ultimately, to result in prolonged renal survival. Therefore, APL-2 is a potentially life-altering therapy for C3G and IC-MPGN.

### 5.2.1. Justification of Study Population

Based on the mechanism of action of APL-2, the Sponsor aims to study patients with a glomerular disease driven by complement over-activation and as such, will include patients with either C3G or IC-MPGN, as both are characterized by complement dysregulation leading to excessive deposition of C3 breakdown products in glomeruli (Iatropoulos, 2018; Cook, 2018; Servais, 2012). Since the same underlying pathophysiology of complement dysregulation that leads to C3G/IC-MPGN in the native kidney also leads to disease recurrence post-transplant, and the clinical course for both is characterized by significant proteinuria and risk of progression to ESRD, the trial includes patients with post-transplant disease recurrence, in addition to non-transplant patients. Finally, the trial includes adolescents (ages 12–17), as the disease features in adolescents are similar to those of adults.

### 5.2.2. Justification of Dose

In this study, APL-2 will be administered as a subcutaneous injection/infusion at a dose of 1080 mg twice weekly.

The relationship between dosing regimen and APL-2 serum concentration is well-predicted and well-understood using a target-mediated drug disposition (TMDD) pharmacokinetic/ pharmacodynamic (PK/PD) model (Mager and Jusko 2001) that has been developed from the cumulative nonclinical and clinical APL-2 SC dosing data. Modelling of exposure in adolescent patients weighing at least 42 kg predicts a PK profile similar to that observed in adults (Figure 1). Therefore, adolescents must weigh at least 42 kg to be enrolled and will receive the same dose of 1080 mg twice weekly.

**Figure 1: Predicted APL-2 PK Concentration for Adolescent Subjects Receiving APL-2 at the Proposed Pediatric Dose Regimens**

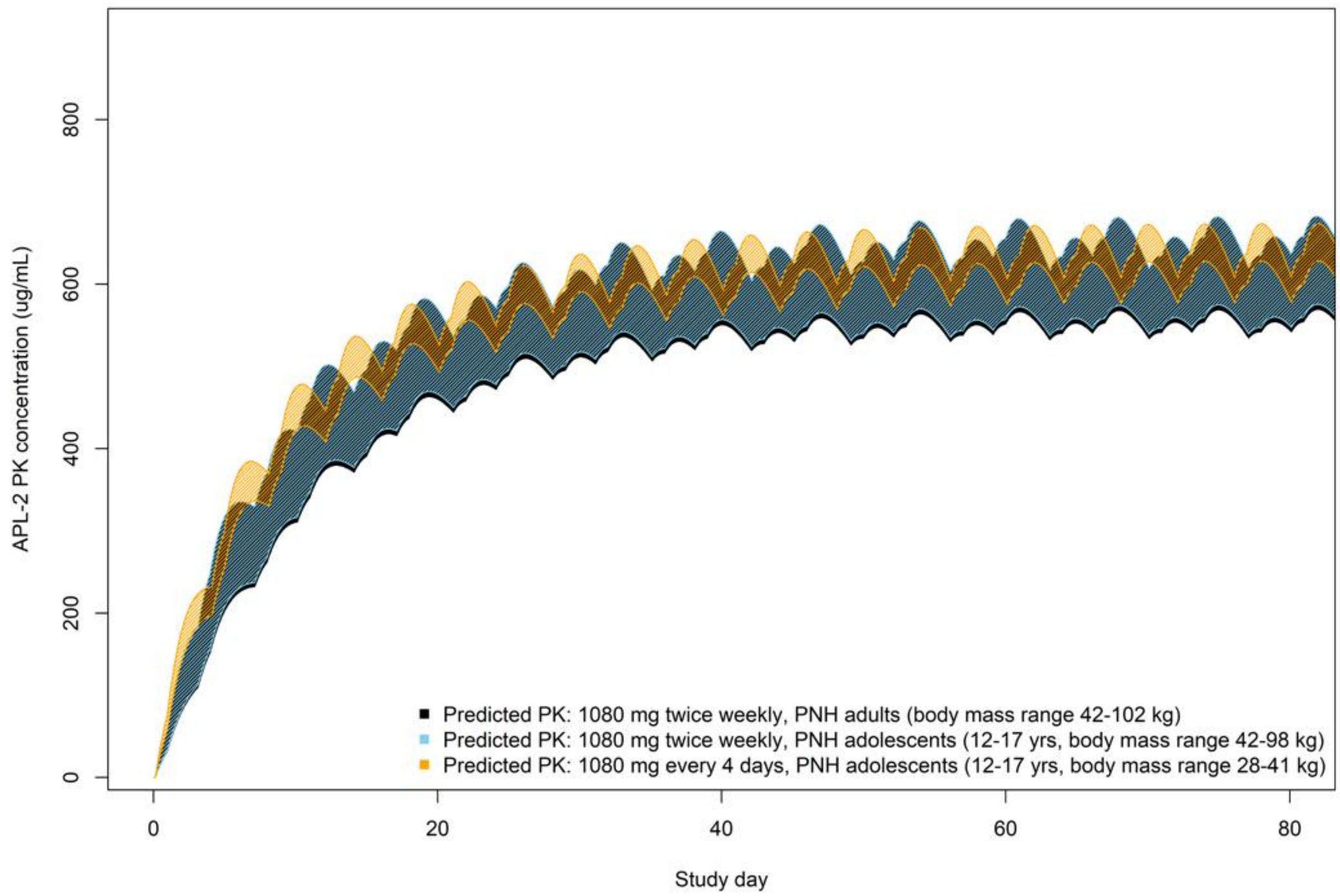


APL-2 SC dose of 1080 mg twice weekly (equivalent to approximately 310 mg/day) results in PK exposures (ie, maximum drug plasma concentration [$C_{max}$]) between 270 and 360 mg/day doses; doses well-tolerated in healthy volunteers and patients [AIHA, PNH and complement-dependent nephropathy (CDN)]. No serious adverse drug reactions (ADRs) were observed at any APL-2 dose tested. The exposures associated with doses of 1080 mg twice weekly is also below the $C_{max}$ observed at the no-observed-adverse-effect level (NOAEL) dose (7 mg/kg/d) in a 9-month chronic toxicity assessment in cynomolgus monkeys.

In addition to being well-tolerated in both healthy volunteers and patients, a dose of 1080 mg twice weekly was associated with substantial and sustained inhibition of the complement system, as measured by the AH50 assay. While higher doses might be associated with increased pharmacology, the clinical experience and safety profile is much more limited for doses above 1080 mg twice weekly. Therefore, a dose of 1080 mg twice weekly is planned as the most appropriate regimen for this clinical trial, maximizing potential pharmacology and benefit to patients while keeping within an exposure range that has been demonstrated to be well-tolerated in human subjects.

Please refer to the latest version of the APL-2 Investigator's Brochure (IB) for the overall risk/benefit assessment and the most accurate and current information regarding the drug metabolism, PK, efficacy, and safety of APL-2.

## 5.3. Summary of Clinical Experience with APL-2

APL-2 is being studied in APL2-201, an ongoing Phase 2 study that includes patients with 1 of 4 glomerular diseases: C3G, immunoglobulin (Ig) A nephropathy, primary membranous nephropathy, or lupus nephritis. Preliminary data from the 8 C3G patients in this study indicate that APL-2 is able to target the complement hyperactivity of C3G, as well as reduce proteinuria in patients with C3G.

Clinical data is also available from 2 ongoing Phase 1b studies with APL-2 in patients with paroxysmal nocturnal hemoglobinuria (PNH) (Studies APL-CP0514 and APL2-CP-PNH-204). Results from these studies indicate that APL-2 may be effective in providing broad control of intravascular and extravascular hemolysis, as evidenced by the following: increased and stable hemoglobin (Hb) levels, normalization of lactate dehydrogenase (LDH), normalization of absolute reticulocyte count (ARC), normalization of total bilirubin levels, reduction in C3 fragment opsonization, and increased numbers of PNH red blood cells (RBCs).

APL-2 at doses of ≤ 360 mg/day has been generally well-tolerated when administered via subcutaneous (SC) infusion, including to patients with C3G and other glomerular diseases.

Please refer to the latest version of the APL-2 IB for the overall risk/benefit assessment and the most accurate and current information regarding the drug metabolism, pharmacokinetic (PK), efficacy, and safety of APL-2.

## 5.4. Clinical Risks/Benefits of APL-2

APL-2 has the potential to address the underlying disease pathophysiology of complement hyperactivity in C3G and IC-MPGN, and, therefore, to provide benefit in these diseases with a high unmet medical need.

The safety of subcutaneous APL-2 administration has been studied in multiple Phase 2 and 3 studies for C3G and PNH, with an acceptable safety profile to date. Nonetheless, a number of safety monitoring practices are being employed by this protocol to ensure patient safety, including physical examination, vital signs monitoring, electrocardiograms (ECGs), hematology (including coagulation), serum chemistry, urinalysis at specified intervals, as well as prompt reporting of adverse events (AEs).

Infusion site/pump safety will be assessed during clinical visits, and any significant finding from the assessment will be reported as an AE (see Section 12.2.4). The volume of blood planned for collection from each patient over the course of the study will be minimized in order to limit the impact on the overall health of these anemic patients.

Systemic complement inhibition might predispose individuals to infections caused by encapsulated organisms, including *Streptococcus pneumoniae* (*S. pneumoniae*), *Neisseria meningitidis* (*N. meningitidis*), and *Haemophilus influenzae* (*H. influenzae*). Vaccinations against these organisms will be taken to minimize potential risk of infection. Use of prophylactic antibiotics is allowed, at the discretion of the Investigator, and should take into consideration the level of immunosuppression, complement levels, and timing of vaccination relative to APL-2 start, as well as local practices. Body temperature and vital signs will be monitored periodically, and relevant blood parameters monitored regularly throughout the study to assess for signs of infection. The patient will be counseled regarding this potential risk for infection and given a

patient safety wallet card in the event of an emergency. The Investigator should be contacted immediately in the event of a suspected infection for guidance on appropriate action to be taken.

# 6. STUDY OBJECTIVES AND ENDPOINTS

## 6.1. Study Objectives

### 6.1.1. Primary Objective

The primary objective of this study is to assess the efficacy of twice weekly SC doses of APL-2 1080 mg compared to no APL-2 treatment in subjects with C3G/IC-MPGN, using proteinuria as measured by uPCR.

### 6.1.2. Secondary Objectives

The secondary objectives of the study are as follows:

- To assess the effect of APL-2 on C3G/IC-MPGN disease parameters
- To assess the safety of APL-2 through incidence of treatment-emergent adverse events (TEAEs)

### 6.1.3. Exploratory Objectives

The exploratory objectives are to characterize the additional clinical, laboratory, and histologic findings of C3G/IC-MPGN in response to APL-2.

## 6.2. Study Endpoints

### 6.2.1. Primary Efficacy Endpoint

The primary efficacy endpoint is the change from baseline in uPCR at Week 26 in subjects treated with APL-2 relative to subjects receiving no APL-2 treatment.

### 6.2.2. Secondary Efficacy Endpoints

The secondary efficacy endpoints are as follows:

- The proportion of patients who achieve at least a 30% reduction in uPCR from baseline at Week 26
- The number and proportion of subjects with a serum albumin above the lower limit of normal at Week 26, for those in whom the parameter was below the lower limit of normal at baseline
- The proportion of patients who achieve at least a 50% reduction in uPCR from baseline at Week 26
- The change from baseline in uPCR at Week 52
- The durability from Week 26 in uPCR reduction at Week 52 for patients randomized to the APL-2 arm
- The change from historical eGFR (up to 3 years prior to screening) at baseline relative to the change from baseline in eGFR at Week 52

- The proportion of patients showing a change in C3c staining on renal biopsy from baseline to Week 26 in those patients with renal biopsies at baseline and Week 26
- The change from Week 26 in uPCR at Week 52

### 6.2.3. Exploratory Endpoints

The exploratory endpoints are as follows:

- The percentage of patients who achieve the following reductions in uPCR:
  - At least a 30% reduction at Week 52
  - At least a 50% reduction at Week 52
  - Normalization at Week 26
  - Normalization at Week 52
- The number and proportion of subjects with normalization of renal disease markers at Week 26 and Week 52, for those in whom the parameter is abnormal at baseline:
  - Serum albumin (Week 52 only)
  - Hematuria
  - Blood pressure
- The time to normalization from baseline for:
  - uPCR
  - Serum albumin
  - Hematuria
  - Blood pressure
- The change from baseline in FACIT-Fatigue, SF-36, EQ-5L-5D, and KDQOL scores at Week 26 and Week 52.
- The change from baseline in the C3G histologic index activity score at Week 26, in those patients with renal biopsies at baseline and Week 26 (Bomback, 2018)
- The change from baseline in uPCR using the 24-hour urine collections at Week 26 and Week 52
- Immunogenicity: presence of antibodies to APL-2 and PEG

### 6.2.4. Safety Endpoints:

The safety endpoints are as follows:

- The incidence and severity of TEAEs
- The change from baseline in vital signs and clinical laboratory tests
- Long term safety and tolerability of APL-2
- The number and incidence of rejection episodes (post-transplant patients only)

### 6.2.5. Pharmacokinetic Endpoints:

The pharmacokinetic endpoints are APL-2 concentrations at Week 26 and Week 52.

### 6.2.6. Pharmacodynamic Endpoints:

The pharmacodynamic endpoints are:

- Changes from baseline in complement levels at Week 26 and Week 52:
  - Serum C3
  - CH50
  - AH50
  - Plasma C5b-9

Additional complement components may be evaluated as exploratory endpoints, as described in Table 3.

# 7. INVESTIGATIONAL PLAN

This Phase 3 randomized, open-label, multicenter, controlled clinical study is designed to evaluate the safety and efficacy of SC APL-2 in patients with primary C3G or IC-MPGN. There will be up to 90 patients enrolled in this study.

The planned length of participation in the study for each subject is a maximum of approximately 84 weeks. This study will consist of 4 parts:

- Part 1: Up to 8-week Screening Period
- Part 2: 26-week Randomized Treatment Period
- Part 3: 26-week Continuation Period
- Part 4: 24-week Follow-up Period (only for those patients who do not roll into the planned long-term extension study)

See Figure 2 for the Study Schematic, and Table 1 for the Schedule of Assessments.

## 7.1. Overall Study Design

This is an open-label, randomized, controlled, Phase 3 study, consisting of a single cohort of up to 90 patients with clinical and pathologic evidence of primary C3G or IC-MPGN. Patients will be randomized 1:1 to either the APL-2 Arm (APL-2 treatment throughout the Randomized Treatment Period and Continuation Period) or the No APL-2 Arm (no APL-2 treatment for the Randomized Treatment Period, followed by APL-2 treatment in the Continuation Period), respectively. The visit schedule will be the same, regardless of randomization.

The study will require 2 renal biopsies, one during screening and another at the end of the Randomized Treatment Period (Week 26). Details around the biopsies are provided in Section 11.5.

After completion of the Randomized Treatment Period and Continuation Period, any patient who, in the opinion of the Investigator, is experiencing clinical benefit from APL-2 administration will be invited to participate in the planned long-term extension study in order to continue to receive treatment with APL-2 until it is commercially available for the disease under study. Those who do not continue on APL-2 in the long-term extension study will enter the Follow-up Period of this study, which is 24 weeks.

See Figure 2 for the study schematic.

**Figure 2: APL2-C3G-310 Study Design**

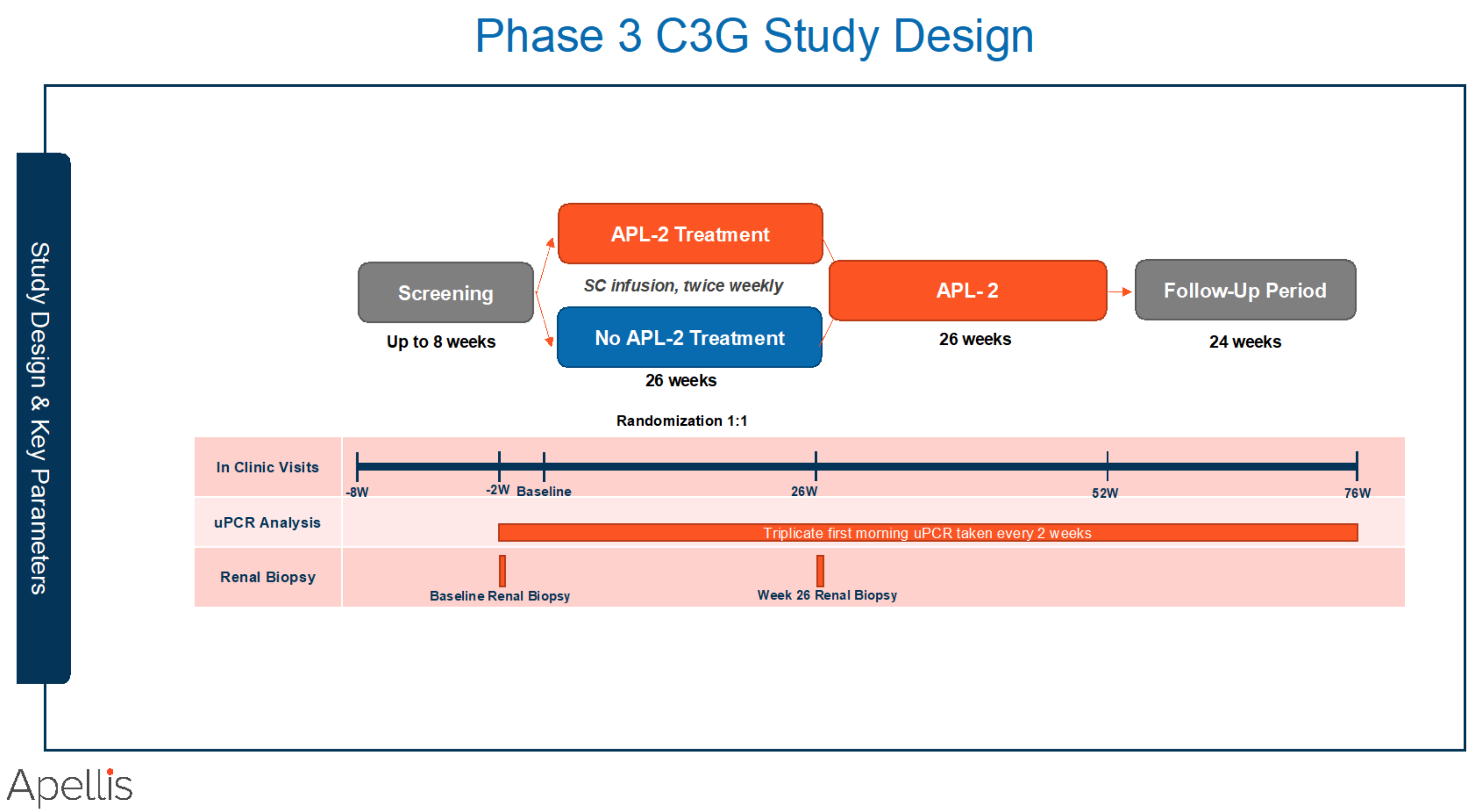

### 7.1.1. Part 1: Screening Period (up to 8 weeks)

Informed consent will be obtained at screening prior to any study related procedures being conducted. Subjects will be screened to confirm that the subject selection criteria for the study have been met. The Screening Period will occur in 2 stages, such that all assessments described at Screening Visit 1 must occur before those in Screening Visit 2. This ensures that the more invasive procedures (vaccinations and renal biopsy) do not occur unless the subject meets all other eligibility criteria.

- A 24-hour urine will be collected to establish eligibility
- A renal biopsy will be performed to confirm diagnosis and eligibility, and to serve as the baseline renal biopsy. All biopsies will be evaluated by the central pathology laboratory. Renal biopsies are discussed in more detail in Section 11.5.
    - A prior renal biopsy done within 6 months of randomization may be used for eligibility confirmation, and serve as the baseline biopsy, if confirmed as adequate by the central pathology laboratory based on review of the biopsy report as well as slides and/or images.
    - Subjects under the age of 18 may opt out of the baseline biopsy provided they have an adequate prior renal biopsy to establish the diagnosis, even if the prior biopsy is more than 6 months prior to randomization.
- Retrospective data will be collected to calculate historical eGFR (using CKD-EPI) for up to 3 years prior to study entry, depending on the date of diagnosis (for renal transplant recipients, this refers to diagnosis of recurrent disease, not diagnosis of the original disease in native kidneys). Retrospective data for eGFR are discussed in more detail in Section 11.1.
- Three sets of triplicate first-morning spot urines will be collected (first-morning void collected on 3 consecutive days) to establish baseline proteinuria for the primary endpoint analysis; the 3 sets must be collected over no more than 8 weeks, and one set must be within 2 weeks of Day 1. Collection of urine samples is discussed in more detail in Section 11.3.
- Central medical review of the case will confirm the diagnosis of primary C3G or IC-MPGN. The central medical reviewer will be a qualified medical expert who will review all available historical, laboratory and pathology data. For an adolescent in whom a renal biopsy within 6 months of Day 1 is not available, the central medical reviewer will also consider whether there is evidence for ongoing complement dysregulation.
- Vaccinations against *S. pneumoniae*, *N. meningitidis* (types A, C, W, Y, and B), and *H. influenzae* (type B) are mandatory unless documented evidence exists that subjects are non-responders to vaccination. If required, vaccinations should occur at least 14 days prior to randomization. Vaccinations are discussed in more detail in Section 9.2.

#### 7.1.1.1. Rescreening

Individuals who consent to participate in the study, but who do not initially meet all the requirements as outlined in the inclusion and exclusion criteria are not able to be enrolled;

however, these individuals may be held in the Screening Period until such time that they are able to be enrolled. In this case, additional screening visits and/or repeat screening assessments may be conducted, as needed, to establish eligibility.

In the event that rescreening occurs, and the patient has not remained in the Screening Period, the individual is required to re-consent and must be assigned a new identification number.

### 7.1.2. Part 2: Randomized Treatment Period (26 weeks)

Approximately 90 subjects who meet all of the inclusion criteria and none of the exclusion criteria will be randomized 1:1 to either the APL-2 Arm or the No APL-2 Arm. To achieve balance between the 2 arms, there will be 2 stratification factors that will be assigned a 1:1 ratio. The first stratification factor examines subjects with post-transplant recurrence versus non-transplant subjects. This is followed by the second stratification factor that examines adolescent subjects (ages 12-17) who opt out of biopsies versus all subjects who did not opt out of biopsies.

- **APL-2 Arm**: Subjects randomized to this arm will receive SC APL-2 at a dose of 1080 mg twice-weekly for 26 weeks.
- **No APL-2 Arm**: Subjects randomized to this arm will not receive APL-2 treatment.
- Other than study drug administration, subjects in the 2 arms will undergo the same schedule, procedures and assessments during Part 2.
- Every effort will be made to minimize changes to medications related to C3G/IC-MPGN disease, in both arms.
- At the end of Part 2, there will be a renal biopsy, which is required for subjects 18 years of age and older to advance to Part 3. Subjects under 18 years of age may opt out of the biopsies, and still advance to Part 3 upon completion of all other Week 24–Week 26 assessments.

Safety and efficacy will be assessed.

### 7.1.3. Part 3: Continuation Period (26 weeks)

At the end of Part 2, subjects from both arms will have the option to continue to Part 3. All subjects participating in Part 3 will be treated with APL-2 1080 mg twice weekly up to Week 52 Subjects who do not continue to Part 3 will continue to Part 4 (Follow-up Period). Subjects who complete Part 3 will enter Part 4, unless they enter the Sponsor-planned long-term extension protocol.

### 7.1.4. Part 4: Follow-up Period (24 weeks)

After completion of Part 3, subjects who would benefit from continuing to receive APL-2, as per Investigator's opinion, may roll over into a separate long-term extension study. Subjects who do not enter the Sponsor-planned long-term extension study will discontinue APL-2 treatment and complete the 24-week Follow-up Period.

## Table 1: Schedule of Assessments

| Study Period | Screening Period (up to 8 weeks) all patients[1] | | Randomization Treatment Period | | | | | | | | Continuation Period | | | | | | Follow-Up Period (24 Weeks) | | | | | |
|---|---|---|---|---|---|---|---|---|---|---|---|---|---|---|---|---|---|---|---|---|---|---|
| **Study Week** | -8 to -4 | -2 | 0 | 4 | 8 | 12 | 16 | 20 | 24 | 26 | 28 | 32 | 36 | 42 | 48 | 52 | 54 | 56 | 58 | 64 | 70 | 76 Exit |
| **Study Day** | -28 | -14 | 1 | 28 | 56 | 84 | 112 | 140 | 168 | 182 | 196 | 224 | 252 | 294 | 336 | 364 | 378 | 392 | 406 | 448 | 490 | 532 |
| **Study Visit** | 1 | 2 | 3 | 4 | 5 | 6 | 7 | 8 | 9 | 10 | 11 | 12 | 13 | 14 | 15 | 16 | 17 | 18 | 19 | 20 | 21 | 22 |
| **Visit Window (± days)** | 0 | [2] | 0 | 3 | 7 | 7 | 7 | 7 | 3 | 3 | 3 | 7 | 7 | 7 | 7 | 7 | 3 | 3 | 3 | 7 | 7 | 7 |
| **Assessments** | | | | | | | | | | | | | | | | | | | | | | |
| Informed Consent | X | | | | | | | | | | | | | | | | | | | | | |
| Demographics | X | | | | | | | | | | | | | | | | | | | | | |
| Medical History | X | | | | | | | | | | | | | | | | | | | | | |
| Inclusion/Exclusion | X | X | X | | | | | | | | | | | | | | | | | | | |
| Vaccination[3] | | X | | | | X | | | | | | | | | | | | | | | | |
| Concomitant Medications | X | X | X | X | X | X | X | X | X | X | X | X | X | X | X | X | X | X | X | X | X | X |
| Physical Examination (Full)[4] | X | | X | | | | | | | X | | | | | | X | | | | | | |
| Physical Examination (Brief)[4] | | X | | X | X | X | X | X | X | | X | X | X | X | X | | X | X | X | X | X | X |
| 12-Lead ECG | X | | X | X | X | X | X | X | X | X | X | X | X | X | X | X | X | X | X | X | X | X |
| Renal Biopsy[5] | | X[6] | | | | | | | | X[7] | | | | | | | | | | | | |
| Randomization | | | X | | | | | | | | | | | | | | | | | | | |
| APL-2 Administration[8] | | | X | X | X | X | X | X | X | X | X | X | X | X | X | X | | | | | | |
| Infusion Site/Pump Safety Assessment[9] | | | X | X | X | X | X | X | X | X | X | X | X | X | X | X | X | | | | | |
| Vital Sign Measurements[10] | X | X | X | X | X | X | X | X | X | X | X | X | X | X | X | X | X | X | X | X | X | X |

## Table 1: Schedule of Assessments

| Study Period | Screening Period (up to 8 weeks) all patients[1] | | Randomization Treatment Period | | | | | | | | Continuation Period | | | | | | Follow-Up Period (24 Weeks) | | | | | |
|---|---|---|---|---|---|---|---|---|---|---|---|---|---|---|---|---|---|---|---|---|---|---|
| **Study Week** | -8 to -4 | -2 | 0 | 4 | 8 | 12 | 16 | 20 | 24 | 26 | 28 | 32 | 36 | 42 | 48 | 52 | 54 | 56 | 58 | 64 | 70 | 76 Exit |
| **Study Day** | -28 | -14 | 1 | 28 | 56 | 84 | 112 | 140 | 168 | 182 | 196 | 224 | 252 | 294 | 336 | 364 | 378 | 392 | 406 | 448 | 490 | 532 |
| **Study Visit** | 1 | 2 | 3 | 4 | 5 | 6 | 7 | 8 | 9 | 10 | 11 | 12 | 13 | 14 | 15 | 16 | 17 | 18 | 19 | 20 | 21 | 22 |
| **Visit Window (± days)** | 0 | [2] | 0 | 3 | 7 | 7 | 7 | 7 | 3 | 3 | 3 | 7 | 7 | 7 | 7 | 7 | 3 | 3 | 3 | 7 | 7 | 7 |
| **<u>Urine</u>** | | | | | | | | | | | | | | | | | | | | | | |
| 24-Hour Urine Collection[11] | X[10] | | See Table 2 | | | | | | | | | | | | | | | | | | | |
| Triplicate First-Morning uPCR[12] | X | X | X[13] | See Table 2[12] | | | | | | | | | | | | | | | | | | |
| In-Clinic (Random) uPCR | X | X | X | X | X | X | X | X | X | X | X | X | X | X | X | X | X | X | X | X | X | X |
| Urinalysis (Dipstick & Microscopic)[14] | X | | | | | | | | | | | | | | | | | | | | | |
| Urine Pregnancy Test | | X | X | X | X | X | X | X | X | X | X | X | X | X | X | X | X | X | X | X | X | X |
| **<u>Blood</u>** | | | | | | | | | | | | | | | | | | | | | | |
| Hematology[14] & Chemistry[14] | X | X | X | X | X | X | X | X | X | X | X | X | X | X | X | X | X | X | X | X | X | X |
| PK Sample Collection | | | X | X | X | X | X | X | X | X | X | X | X | X | X | X | X | X | X | X | X | X |
| Anti-APL-2/PEG Ab assays[15] | | | X | X | | | X | | X | | | | X | | | X | X | | | | | X |
| Complement Biomarker Profile[14] | X | X | X | X | X | X | X | X | X | X | X | X | X | X | X | X | X | X | X | X | X | X |
| eGFR[16] | X | X | X | X | X | X | X | X | X | X | X | X | X | X | X | X | X | X | X | X | X | X |
| Pregnancy (β-HCG) | X | | | | | | | | | X | | | | | | | | | | | | |
| FSH, HIV, HCV, HbsAg[17] | X | | | | | | | | | | | | | | | | | | | | | |

## Table 1: Schedule of Assessments

| Study Period | Screening Period (up to 8 weeks) all patients[1] | | Randomization Treatment Period | | | | | | | | Continuation Period | | | | | | Follow-Up Period (24 Weeks) | | | | | |
|---|---|---|---|---|---|---|---|---|---|---|---|---|---|---|---|---|---|---|---|---|---|---|
| **Study Week** | -8 to -4 | -2 | 0 | 4 | 8 | 12 | 16 | 20 | 24 | 26 | 28 | 32 | 36 | 42 | 48 | 52 | 54 | 56 | 58 | 64 | 70 | 76 Exit |
| **Study Day** | -28 | -14 | 1 | 28 | 56 | 84 | 112 | 140 | 168 | 182 | 196 | 224 | 252 | 294 | 336 | 364 | 378 | 392 | 406 | 448 | 490 | 532 |
| **Study Visit** | 1 | 2 | 3 | 4 | 5 | 6 | 7 | 8 | 9 | 10 | 11 | 12 | 13 | 14 | 15 | 16 | 17 | 18 | 19 | 20 | 21 | 22 |
| **Visit Window (± days)** | 0 | [2] | 0 | 3 | 7 | 7 | 7 | 7 | 3 | 3 | 3 | 7 | 7 | 7 | 7 | 7 | 3 | 3 | 3 | 7 | 7 | 7 |
| **Other Assessments** | | | | | | | | | | | | | | | | | | | | | | |
| AEs | X | X | X | X | X | X | X | X | X | X | X | X | X | X | X | X | X | X | X | X | X | X |
| HRQoL[18] | | X | | | | | | | | X | | | | | | X | | | | | | |
| Post-Transplant Immunosuppression Plan Documentation[19] | | X | | | | | | | | | | | | | | | | | | | | |
| **Optional Assessments** | | | | | | | | | | | | | | | | | | | | | | |
| Ophthalmologic Evaluation, including SD-OCT[20] | | X[20] | | | | | | | | | | | | X[20] | | | | | | | | |
| Measured GFR[21] | | | X | | | | | | | | | | | X | | | | | | | | |

Abbreviations: ADA = antidrug antibodies; AE = adverse events; β-HCG = beta human chorionic gonadotropin; CDC = Centers for Disease Control and Prevention; CKD-EPI = Chronic Kidney Disease Epidemiology Collaboration; ECG = electrocardiogram; eGFR = estimated glomerular filtration rate; FSH = follicle stimulating hormone; GFR = glomerular filtration rate; HBsAg, hepatitis B surface antigen; HCV = hepatitis C virus; HIV = human immunodeficiency virus; PEG Ab = polyethylene glycol circulating antibodies; PK = pharmacokinetic(s); RBC = red blood cell; SC = subcutaneous; SD-OCT = spectral domain optical coherence tomography; uPCR = urine protein-to-creatinine ratio.

**NOTE:** When multiple assessments/procedures occur at the same time, order should be vital signs, ECGs, blood collection/sampling, APL-2 dosing.

[1] Patients who meet the eligibility requirements from Visit 1 will return for the assessments indicated in Visit 2; however, all Visit 2 assessments need not occur in a single visit; therefore, Visit 2 can be split into multiple visits (eg, 2a, 2b).

[2] Study Visit 2 must occur at least 7 days after Study Visit 1 and at least 14 days prior to the Day 1 dosing date; the vaccinations and biopsy can occur any time after confirmation of eligibility based on Study Visit 1 data.

[3] Vaccines should be administered at least 14 days prior to randomization; additional vaccines may be required at Visit 6. Please see Section 9.2 for more details on vaccination requirements.

[4] A full physical examination is required only at Study Weeks -4 to -8, 1 and 52. Brief physical examinations, including weight (kg) and assessment of edema, will be conducted at all other timepoints noted. A symptom-driven physical examination may be performed at other times, at the Investigator's discretion. Body height (cm) will be measured during

screening for adults and every 12 weeks throughout the study for adolescents; weight (kg) will be measured throughout study, during brief and full physical examinations. Both body weight and height will be assessed without shoes on. Edema should be assessed at every visit.

[5] Patients under the age of 18 may opt out of the renal biopsy provided they have an adequate prior renal biopsy to establish the diagnosis.

[6] The baseline renal biopsy should be conducted only if the patient remains eligible based on assessments from Visit 1.

[7] The Week 26 renal biopsy need not occur on the same day as all other Week 26 assessments. However, the Week 26 triplicate uPCR must occur prior to the renal biopsy, and for Control patients, the Week 26 biopsy must be performed prior to initiation of APL-2 treatment. Subjects under 18 years of age who opt out of the baseline biopsy will not have a Week 26 biopsy.

[8] Patients will self-administer SC APL-2, after receiving appropriate training and sign-off by a research nurse (or other qualified personnel) in their first treatment week. During site visits, patients will self-administer APL-2 at the site with the exception of the very first dose.

[9] Between site visits, patients will be instructed to report any infusion site reaction to the study staff. Pump use safety assessment will be performed by licensed healthcare professional (ie, Investigator or nurse) within 30 minutes following study drug administration at all clinic visits and during at-home qualification. See Manual of Procedures for more details.

[10] Vital signs should be measured a maximum of 2 hours before APL-2 infusion. On Day 1, vital signs should also be measured at 30 minutes (± 5 minutes) after the first infusion of APL-2 dosing., timed from the completion of the study drug administration. Blood pressure and heart rate should be evaluated after the patient has been resting in a seated, recumbent, or supine position for at least 5 minutes.

[11] The 24-hour urine collection should be done following Screening Visit 1.

[12] Triplicate First-Morning urinary PCR samples will be collected by the patient at home on 3 consecutive days, every 2 weeks, throughout the duration of the study. The first-morning uPCR should be the first urinary output of the day. An additional triplicate first-morning uPCR sample will be collected at Week 25. Each triplicate can either be brought to the clinic by the patient, or collected from the patient via courier. At every visit, enough uPCR collection containers need to be dispensed to the patient to enable all at-home uPCR collections until the next clinic visit.

[13] The Day 1 first-morning triplicate uPCR samples should be collected before the first dose of APL 2 (eg, Day -2, Day -1 and Day 1).

[14] See laboratory assessments (Table 3) for more details.

[15] The Day 1 samples should be collected before dosing with APL-2. Patients who discontinue dosing will need to have ADA samples collected at 6 and 12 weeks after the last treatment. Patients who are considered treatment-boosted for anti-APL-2/PEG Abs at any time will be followed with ADA samples being collected every 6 months until the antibody levels revert to baseline.

[16] eGFR will be calculated using the Chronic Kidney Disease -Epidemiology Collaboration (CKD-EPI) creatinine equation for adults, or the Bedside Schwartz equation for adolescents.

[17] Serum FSH to be performed on females only (see laboratory manual for more details).

[18] FACIT-Fatigue, SF-36, EQ-5L-5D, and KDQOL

[19] For transplant patients only. Must be documented prior to randomization.

[20] SD-OCT is optional and will be performed at selected sites; if done, it should occur once prior to randomization and once again between Weeks 44 and 52.

[21] Measured GFR is an optional assessment which, if done, should performed at 2 time points: once on Day 1, or within 8 weeks before Day 1, and again at 1 timepoint between Week 44 and Week 52, inclusive. Measured GFR should only be done at sites where it is routinely performed, as per the site’s standard protocol.

## Table 2: Urine Collection Schedule: Randomized Treatment Period

| Study Week | Week -2 | Week 0 | Week 2 | Week 4 | Week 6 | Week 8 | Week 10 | Week 12 | Week 14 | Week 16 | Week 18 | Week 20 | Week 22 | Week 24 | Week 25 | Week 26 |
|---|---|---|---|---|---|---|---|---|---|---|---|---|---|---|---|---|
| **Study Day** | -14 | 1 | 14 | 28 | 42 | 56 | 70 | 84 | 98 | 112 | 126 | 140 | 154 | 168 | 175 | 182 |
| **Study Visit** | 2 | 3 | | 4 | | 5 | | 6 | | 7 | | 8 | | 9 | | 10 |
| Triplicate first morning uPCR | X[1] | X[1] | X | X | X | X | X | X | X | X | X | X | X | X | X | X |
| 24 Hour urine collection | X | | | | | | | X | | | | | | X | | |

[1] Three sets of triplicate first morning uPCR need to be collected within the 8 weeks prior to Study Day 1 (Week 0). Two sets can be collected between Study Visits 1 and 2, with 1 set being during the 3 days prior to Study Visit 2. The last set can be collected during the 3 days prior to Study Visit 1.

## Urine Collection Schedule: Continuation Period

| Study Week | Week 28 | Week 30 | Week 32 | Week 34 | Week 36 | Week 40 | Week 42 | Week 44 | Week 46 | Week 48 | Week 50 | Week 52 |
|---|---|---|---|---|---|---|---|---|---|---|---|---|
| **Study Day** | 196 | 210 | 224 | 238 | 252 | 280 | 294 | 308 | 322 | 336 | 350 | 364 |
| **Study Visit** | 11 | | 12 | | 13 | | 14 | | | 15 | | 16 |
| Triplicate first morning uPCR | X | X | X | X | X | X | X | X | X | X | X | X |
| 24 Hour urine collection | | | | | | | | | | X | | |

## Urine Collection Schedule: Follow-up Period

| Study Week | Week 54 | Week 56 | Week 58 | Week 60 | Week 62 | Week 64 | Week 66 | Week 68 | Week 70 | Week 72 | Week 74 | Week 76 |
|---|---|---|---|---|---|---|---|---|---|---|---|---|
| **Study Day** | 378 | 392 | 406 | 420 | 432 | 448 | 464 | 476 | 490 | 504 | 518 | 532 |
| **Study Visit** | 17 | 18 | 19 | | | 20 | | | 21 | | | 22 |
| Triplicate first morning uPCR | X | X | X | X | X | X | X | X | X | X | X | X |
| 24 Hour urine collection | | | | | | | | | | | | X |

Shaded columns indicate samples to be collected at home and brought to the clinic during visit weeks; unshaded columns indicate samples to be collected at home and picked up by courier during non-visit weeks.

## 7.2. Number of Subjects

Approximately 90 patients with clinical and pathologic evidence of C3G or IC-MPGN will be randomized into this study.

## 7.3. Treatment Assignment

All subjects who meet all the inclusion criteria and none of the exclusion criteria will be enrolled in the study, until such time that the intended enrollment has been met. After confirmation of study eligibility, subjects will be randomized in a ratio of 1:1 to receive APL-2 or no APL-2 treatment as described in Section 9.5.1.

Following completion of Part 2, all subjects will receive APL-2 starting on the first visit of Part 3 (Continuation Period), after which they will enter Part 4 (the Follow-up Period), unless they enroll in the Sponsor-planned long-term extension study.

## 7.4. Criteria for Study Termination

The Sponsor reserves the right to suspend or discontinue this study for administrative and/or safety reasons, at any time. The Investigator reserves the right to discontinue dosing subjects, at any time, for safety reasons.

# 8. SELECTION AND WITHDRAWAL OF SUBJECTS

## 8.1. Subject Inclusion Criteria

1. At least 18 years of age; where approved, adolescents (ages 12–17) weighing at least 42 kg may also be enrolled
2. A diagnosis of primary C3G or IC-MPGN with evidence of ongoing complement activation, based on central pathology and central medical review of the case
3. At least 2+ staining for C3c on the baseline renal biopsy collected during screening, per the central pathology laboratory
4. No more than 50% global glomerulosclerosis or tubulointerstitial scarring on the baseline biopsy collected during screening
5. uPCR ≥ 1000 mg/g, and at least 1 g/day of proteinuria, on a screening 24-hour urine collection
6. eGFR ≥ 30 ml/min/1.73 $m^2$ calculated by the Chronic Kidney Disease -Epidemiology Collaboration (CKD-EPI) creatinine equation for adults, or the Bedside Schwartz equation for adolescents
7. Body mass index (BMI) ≤ 35 $kg/m^2$
8. Stable regimen for C3G/IC-MPGN treatment for at least 4 weeks prior to the baseline renal biopsy collected during screening, and at least 8 weeks (or 5 half-lives of medications, whichever is greater) prior to randomization
9. Have received vaccinations against *S. pneumoniae*, *N. meningitidis* (types A, C, W, Y, and B), and *H. influenzae* (type B) within 5 years prior to randomization, or agree to receive vaccinations during screening. Vaccinations should occur at least 14 days prior to randomization. Vaccination is mandatory, unless documented evidence exists that subjects are non-responders to vaccination (as evidenced by titers or display titer levels within acceptable local limits).
10. Female subjects of child-bearing potential must have a negative blood pregnancy test at screening (and negative urine pregnancy test on Day 1) and must agree to use protocol-defined methods of contraception from screening through at least 90 days after receiving the last dose of APL-2.
11. Male subjects must agree to use protocol-defined methods of contraception and agree to refrain from donating semen from screening through at least 90 days after receiving the last dose of APL-2.
12. Willing and able to provide informed consent

## 8.2. Subject Exclusion Criteria

1. Previous treatment with APL-2

2. Improving renal disease in the 8 weeks prior to screening, or during the Screening Period; improving renal disease is defined as > 30% increase in eGFR, or > 50% decrease in proteinuria
3. Evidence of rejection on the baseline renal biopsy collected during screening, that requires treatment, in a renal transplant patient
4. C3G/IC-MPGN secondary to another condition (eg, infection, malignancy, monoclonal gammopathy, autoimmunity, chronic antibody-mediated rejection, or a medication)
5. Current or prior diagnosis of human immunodeficiency virus (HIV), hepatitis B (HBV), or hepatitis C (HCV) infection, or positive serology during screening that is indicative of infection
6. Malignancy, except for the following:
   a. Cured basal or squamous cell skin cancer
   b. Curatively treated in situ disease
   c. Malignancy-free and off treatment for ≥ 5 years
7. Significant other renal disease that would confound interpretation of study results
8. Participation in any other investigational drug trial or exposure to other investigational agent, device or procedure within 30 days or 5 half-lives from the last dose of investigational agent (whichever is longer) prior to Screening Period
9. Use of rituximab, belimumab, or any approved or investigational anti-complement therapy other than APL 2 within 5 half-lives of that product prior to the Screening Period
10. Female subjects who are currently breastfeeding and are unwilling to discontinue for the duration of the study and for at least 90 days after the final dose of study drug
11. Inability to cooperate or any condition that, in the opinion of the Investigator, creates an undue risk for the patient by participating in the study, or is likely to confound interpretation of the study results
12. Evidence of, in the opinion of the Investigator, drug or alcohol abuse or dependence
13. Presence or suspicion of severe recurrent or chronic infections that, in the opinion of the Investigator, may place the subject at unacceptable risk by study participation
14. Presence or suspicion of liver dysfunction as indicated by elevated alanine aminotransferase (ALT), aspartate aminotransferase (AST), or bilirubin levels > 2× upper limit of normal (ULN).

## 8.3. Subject Withdrawal Criteria

Subjects will be considered to have completed the study when they have completed the Week 76 exit visit activities described in the Schedule of Assessments (Table 1), when they have enrolled in a subsequent study of APL-2, or when they have discontinued study treatment and completed any early termination activities.

A subject may withdraw from the study at any time, for any reason, without prejudice to his/her future medical care. The Investigator or Sponsor may withdraw the subject at any time (eg, in the

interest of the subject's safety). If a subject discontinues or is withdrawn from study treatment for any reason, the study site must immediately notify the Medical Monitor.

Once withdrawn from the study, the subject may not re-enter the study.

Subjects who discontinue study treatment prior to the end of the Continuation Period should undergo Part 4 of the study, including all follow-up visits and procedures through study completion, unless unwilling or unable, or consent has been withdrawn. Subjects who wish to fully withdraw from the study before completion should be encouraged to complete the activities described for the Week 76 exit visit in the Schedule of Assessments (Table 1).

### 8.3.1. Reasons for Discontinuation

The date of and reason for treatment discontinuation must be determined by the investigator and recorded in the patient's medical record and on the appropriate electronic case report form (eCRF). If it is determined that there was more than 1 reason for the discontinuation, each reason should be documented in the source document and the most clinically relevant reason, as determined by the Investigator, should be entered on the eCRF.

Any subject who discontinues treatment early, or is withdrawn from the study early, due to a treatment-emergent adverse event (TEAE), whether serious or non-serious, will be followed until the TEAE resolves (returns to normal or baseline values) or stabilizes, or until it is judged by the Investigator to no longer be clinically significant.

If consent is withdrawn, no further study evaluations are to be performed and no attempts are to be made to collect additional data.

Additionally, the Sponsor may stop the study at any time for safety, regulatory, legal, or other reasons aligned with good clinical practice (GCP). This study may be terminated at the discretion of the Sponsor or any regulatory agency. An Investigator may elect to discontinue or stop the study at his/her study site for any reason, including safety or low enrollment.

### 8.3.2. Subjects Lost to Follow-up Prior to Last Scheduled Visit

A minimum of 3 documented attempts must be made to contact any subjects lost to follow-up at any time point prior to the last scheduled contact (office visit or telephone contact). At least 1 of these documented attempts must include a written communication sent to the subject's last known address via courier or mail (with an acknowledgment of receipt request) asking that he/she return to the site for final safety evaluations and to return any investigational product.

Should the participant continue to be unreachable, he/she will be considered to have withdrawn from the study.

# 9. TREATMENT OF SUBJECTS

## 9.1. Description of Study Drug

The study drug is APL-2 (pegcetacoplan) which will be provided as a sterile solution of APL-2, 1,080 mg/20 mL in acetate-buffered sorbitol, supplied in stoppered glass vials. Additional information is provided in the APL-2 IB.

## 9.2. Vaccinations

In order to receive the investigational product, subjects must have documented evidence of vaccination against the following within 5 years of Baseline Visit 2b:

1. *Neisseria meningitidis* types A, C, W, Y, and B (administered as 2 separate vaccinations)
2. *Streptococcus pneumoniae* (with a PCV13 and PPSV23 vaccine)
3. *Haemophilus influenzae* type B (Hib vaccine).

For subjects who do not have documented evidence of receiving any of the above vaccinations within 5 years prior to Visit 3, the required missing vaccination(s) will be administered at least 2 weeks prior to Visit 3; however, in the event that an expeditious study start is warranted, the vaccination schedule may be adjusted with written approval from the sponsor. Vaccination is mandatory, unless documented evidence exists that subjects are non-responders to vaccination (as evidenced by titers or display titer levels within acceptable local limits). The Investigator will discuss with the Sponsor regarding individual patient circumstances. If the subject requires vaccination against *N. meningitidis*, first vaccination should be administered at least 2 weeks prior to Visit 3 and a booster should be administered at least 8 weeks after the initial vaccination. If the subject requires vaccination against *S. pneumoniae*, PCV13 will be administered at least 2 weeks prior to Visit 3, and PPSV23 will be administered at least 8 weeks after the PCV13 vaccination. If the subject requires vaccination against *H. influenzae*, the Hib vaccine will be administered at least 2 weeks prior to Visit 3.

### 9.2.1. Empiric Antibiotic Treatment for Possible Infection

Body temperature, vital signs, and relevant blood parameters will be monitored regularly throughout the study to assess for signs of infection. The Investigator should be contacted immediately in the event of a suspected infection for guidance on appropriate action to be taken. Action to be taken may include empiric administration of a broad-spectrum antibiotic to cover possible serious infections, such as meningococcus.

## 9.3. Concomitant Medications

All medications administered (including over the counter or prescription medicines, vitamins, and/or herbal supplements) and all procedures performed within 12 weeks of Screening will be collected as prior medications and procedures. Medications administered and procedures performed from the time of informed consent through the Week 76 Exit Visit are regarded as concomitant and will be documented.

Patients should be on stable doses of all medications relevant to their renal disease for at least 4 weeks prior to the baseline renal allograft biopsy collected during screening. During the

Screening Period and Part 2, changes to the baseline treatment regimens for C3G/IC-MPGN (including medications, dietary restrictions and lifestyle modifications) should be minimized to the extent possible, and made only when required for the well-being of the patient (with the exception of post-transplant immunosuppression – see below). During Part 3, if the PI plans to make changes to the baseline treatment regimen, these should be approved by the Sponsor before implementation.

### 9.3.1. Post-Transplant Immunosuppression

If the patient is in a post-transplant timeframe in which immunosuppression or other transplant-related medication adjustments are anticipated, these adjustments should still occur, adhering to the center's standard protocol for post-transplant care. For all post-transplant patients, the plan for post-transplant immunosuppression for the duration of the study should be documented prior to randomization. Any deviation during the study from the pre-specified plan should be documented with a justification for the change.

Post-transplant patients with evidence of rejection on the baseline biopsy collected during screening that requires treatment will need to undergo their anti-rejection treatment, and then can be reconsidered for APL-2 dosing, but will need to meet all other study eligibility criteria, including a repeat biopsy to serve as the baseline (refer to Section 11.5 for further details). Patients with evidence of subclinical rejection on the biopsy collected during screening may proceed with APL-2 treatment, at the discretion of the Investigator, provided no treatment is initiated for the rejection.

### 9.3.2. Restricted and Prohibited Medications

The use of rituximab, belimumab, or any approved or investigational anti-complement therapy other than APL-2 is prohibited within 5 half-lives of that product prior to screening.

## 9.4. Treatment Compliance

Patients must be instructed to bring their empty/unused investigational product packaging and diaries to every visit. The pharmacist/nominated person will record details on the drug accountability form.

## 9.5. Measures to Minimize Bias: Study Treatment Assignment

### 9.5.1. Randomization

Subjects will be randomized in a ratio of 1:1 via a computer-generated randomization schedule to receive APL-2 or no APL-2 treatment. The randomization will be performed centrally. To achieve balance between the 2 arms, there will be 2 stratification factors that will be assigned a 1:1 ratio between the 2 arms. The first stratification factor examines subjects with post-transplant recurrence versus non-transplant subjects. This is followed by the second stratification factor that examines adolescent subjects (ages 12-17) who opt out of biopsies versus all subjects who did not opt out of biopsies. The stratification will ensure balance between treatment groups for the respective stratification factors.

### 9.5.2. Blinding

This study is not blinded; however, the central pathologist will be blinded to treatment assignment.

# 10. STUDY DRUG MATERIALS AND MANAGEMENT

## 10.1. Study Drug

The test product is APL-2 (pegcetacoplan) which will be provided as a sterile solution of APL-2, 1080 mg/20 mL in acetate-buffered sorbitol, supplied in stoppered glass vials. Additional information is provided in the APL-2 IB.

## 10.2. Study Drug Packaging and Labeling

### 10.2.1. Packaging

Investigational product is supplied in 20 mL glass vials. Please refer to the Pharmacy Manual for details.

Changes to Sponsor-supplied packaging prior to dosing may not occur without full agreement, in advance, by the Sponsor.

### 10.2.2. Labeling

Labels containing study information and pack identification are applied to the investigational product(s) container.

All investigational product is labeled with a minimum of the following: protocol number, dosage form (including product name and quantity in pack), route of administration, directions for use, storage conditions, batch number and/or packaging reference, the statements "For clinical trial use only" and/or "CAUTION: New Drug – Limited by Federal (or United States [US]) Law to Investigational Use," and name and address of the Sponsor.

Space is allocated on the label so that the site representative can record a unique subject identifier and date dispensed by the site to the subject.

Additional labels may, on a case-by-case basis, be applied to the investigational product in order to satisfy national, local, or institutional requirements, but must not do any of the following: contradict the clinical study label, obscure the clinical study label, identify the study subject by name.

Additional labels may not be added without the Sponsor's prior full agreement.

## 10.3. Study Drug Storage

The investigational product should be stored refrigerated at 2°C-8°C, both at home and in the clinic. Temperature monitoring is required at the Investigator site (or documented storage location) to ensure that the investigational product is maintained within an established temperature range.

The investigator or appropriately qualified site staff will be responsible for the following:

- ensuring that the investigational product is stored in a secure, limited-access location at the site
    - limited responsibility may be delegated to the pharmacy or member of the study team, but this delegation must be documented

- ensuring that the temperature is monitored throughout the duration of the study and that records are maintained

A pharmacist or appropriately-qualified designated person will be responsible for the following:

- storing the investigational product appropriately
- dispensing the vials of investigational product to the subject
- entering the unique subject identifier on the investigational product bottle/carton labels as they are distributed

When the subject receives the investigational product from the site, it should be transported in a Sponsor-approved bag or box, containing previously temperature-conditioned cold plates to ensure that the storage temperature (2°C-8°C) is maintained. Temperature monitoring will not be required during transport or at the subject's residence, but a log will be kept for every injection/infusion to ensure that all investigational product was kept refrigerated.

## 10.4. Injection/Infusion Supplies

The Sponsor will supply syringes, vial adapters, injection/infusion sets, and ambulatory syringe injection/infusion pumps, as required. Refer to the Pharmacy Manual for further details.

## 10.5. APL-2 Administration

Investigational product, APL-2, will be administered as a 20 mL SC injection/infusion.

The preferred site of injection/infusion will be the abdomen. If administration into the abdomen is not feasible, alternative appropriate sites are acceptable (see Pump Instructions for Use for more details). Research nurses or other appropriately qualified research personnel will administer the SC injections/infusions (as needed) and will qualify and supervise the self-administration (or caregiver administration). These qualified personnel will be made available for a minimum of 6 days on treatment (2 doses) to ensure the subject has been qualified to conduct self-administration, duration could be shortened if qualification happens sooner. During qualification, the subject (or caregiver) must demonstrate to the research personnel their ability to safely and effectively administer study drug using the injection/infusion pump. Following self-administration qualification, subjects may self-administer the SC injections/infusions without supervision. Once qualified, the subject or caregiver will continue to self-administer injections/infusions at the clinic on those days when a clinic visit occurs and at an off-site location convenient to the subject on all other days. Self-administration conducted at the clinic will be supervised to ensure that the subject continues to remain compliant with the administration guidelines.

NOTE: If the subject requires further training, the self-administration qualification period may be extended beyond 6 days on treatment (2 doses). Self-administration may also be conducted by a member of the subject's household or family member, etc. who will undergo the same qualification criteria by the in-home medical professional (qualification is not intended to be restricted to the subject).

## 10.6. Study Drug Accountability

Accountability for the study drug at the study center is the responsibility of the Investigator. The Investigator will ensure that the study drug is used only in accordance with this protocol. Where allowed, the Investigator may choose to delegate drug accountability responsibilities to a pharmacist or other appropriate individual.

Investigators will be provided with sufficient amounts of the investigational product to carry out this protocol for the agreed number of subjects. The Investigator or designee will acknowledge receipt of the investigational product, documenting shipment content and condition. Accurate records of all investigational product dispensed, used, returned, and/or destroyed must be maintained. Accountability records will include dates, quantities, batch/serial numbers, expiration dates (if applicable), and participant numbers. The Sponsor or its designee will review study drug accountability at the study center on an ongoing basis during monitoring visits.

Study drug must not be used for any purpose other than the present study. Study drug that has been dispensed to a participant and returned unused must not be re-dispensed to a different participant.

The Investigator is responsible for ensuring the retrieval of all returnable study supplies from subjects.

## 10.7. Study Drug Handling and Disposal

All unused and used study drug vials should be retained at the center until they are inventoried by the study monitor. All used, unused, or expired study drug vials will be returned to the Sponsor or its designee for destruction and destruction will be documented, or if authorized, disposed of at the study center per the center's standard operating procedures and documented.

# 11. ASSESSMENT OF EFFICACY

## 11.1. Retrospective Data Collection for eGFR and Measured GFR

Retrospective data will be collected on GFR to calculate historical eGFR for up to 3 years prior to study entry, depending on the date of diagnosis (for renal transplant recipients, this refers to diagnosis of recurrent disease. Three years of retrospective data will be collected to calculate eGFR for those diagnosed at least 3 years prior to randomization. Retrospective data for the period between randomization and diagnosis will be collected for those diagnosed less than 3 years prior to randomization. For historical eGFR calculations, data will be collected to enable calculation of eGFR by CKD-EPI (with serum creatinine, serum cystatin C, or both) in adults, and by Bedside Schwartz or the creatinine-cystatin C-based CKiD equation in adolescents. Specifically, age, sex, race, height (adolescents only), serum creatinine, serum BUN (adolescents only), and serum cystatin C (if available) will be collected.

If a patient has historical data on measured GFR, then this data, including the method used to measure GFR, should also be collected (in addition to required data to calculate eGFR in the same patient) for the same timeframe.

## 11.2. 24-Hour Urine Collection

A 24-hour urine collection is defined as collection of all urine produced over a 24-hour period. As 24-hour urine collections will be done at home, patients will be provided with containers for the collection as well as an instruction sheet that details the collection procedure. The 24-hour urine collection should begin following the first urinary output for the day, and then continue for a 24-hour period. In brief, the first urine (on the day of collection) must be discarded (as this is urine made prior to the start of the collection date/time). The time and date of this discarded void must be recorded, as it represents the start of the collection. The patient should then collect all urine over the next 24 hours. At the 24-hour timepoint, the patient should empty their bladder and collect this urine as the final void of the 24-hour urine collection. The date and time of this last collected void should be recorded, as it represents the end of the 24-hour urine collection.

There will be five 24-hour urine collections, at the time points outlined in the Schedule of Assessments (Table 1):

- Week 1 (screening; prior to the renal biopsy)
- Week 12
- Week 24 (prior to the renal biopsy)
- Week 48
- Week 76

If menses are occurring at the scheduled time of a 24-hour urine collection, then the collection should be delayed until completion of menses, with this information recorded in the eCRF.

Urine will need to be stored at 2°C-8°C (ie, in refrigerator). Courier arrangements can be made by the site to pick up the collection container from the patients and return it to the site, or the patient may return it directly to the site. Total urine volume, total protein concentration, total

albumin concentration, total B-2 microglobulin, and total creatinine concentration will be measured on the 24-hour urine collections, with calculation of total protein excretion, total albumin excretion, total creatinine excretion, total B-2 microglobulin excretion, uPCR, urinary albumin-to-creatinine ratio (uACR), and urinary B2-microglobulin-to-creatinine ratio (uBCR) based on the 24-hour collection.

## 11.3. Triplicate First-Morning Spot Urine Collections

Triplicate first-morning spot urine collection is defined as a collection of first-morning urine on 3 consecutive mornings, with each morning collected into a separate container.

At each scheduled clinic visit, the site will dispense the number of spot urine containers needed prior to the next scheduled visit (see Table 2). For collection weeks in which there is a scheduled clinic visit, patients will return the triplicate spot urine collections at the visit. On weeks where no clinic visit is scheduled, a courier service can be arranged to pick up samples from the patient's home for delivery to the clinic, or the patient can deliver the samples directly to the clinic.

It is important that each of these collections represent the first urinary output upon awakening, after an extended period of rest. The date and time of each collected sample should be recorded. In the event that a patient has an atypical sleep/wake schedule, the appropriate timing for these spot urine collections may not be morning. In this case, the timing of the collection should be adjusted to ensure collection of the first urinary output after an extended period of rest (eg, at least 6 hours), and the alternate timing should be documented in the eCRF. After collection, the urine samples must be stored at 2°C-8°C (ie, in refrigerator). Urine protein, albumin, B-2 microglobulin, and creatinine concentrations should be measured on all spot urine samples, with calculation of uPCR, uACR, and uBCR values.

The schedule for the triplicate first-morning spot urine collections is as follows:

- Dispense uPCR containers for consecutive collection every 2 weeks, per the following container dispersal schedule:
  - Dispense 3 containers (visits spaced every 2 weeks): Weeks 1, 2, 12, 14, 52, 54, 56
  - Dispense 6 containers (visits spaced every 4 weeks): Weeks 4, 8, 16, 20, 48
  - Dispense 9 containers (visits spaced every 6 weeks): Weeks 24, 30, 36, 42, 58, 64, 70

## 11.4. Random Spot Urine Collection

At each clinic visit, patients will be asked to submit a urine sample. As this sample will not necessarily reflect a specific time of day, it is considered a "random" spot urine sample, so as to distinguish it from the first-morning spot collections. After collection, the urine samples must be stored at 2°C-8°C (ie, in a refrigerator). Urine protein, albumin, B-2 microglobulin, and creatinine concentrations should be measured on all spot urine samples, with calculation of uPCR, uACR, and uBCR values.

## 11.5. Renal Biopsy

A percutaneous biopsy will be performed at 2 timepoints: once during screening and again at Week 26, as indicated in the Schedule of Assessments (Table 1).

A renal biopsy will be performed during screening to confirm diagnosis and eligibility, and to serve as the baseline renal biopsy. A prior renal biopsy done within 6 months of randomization may be used for eligibility confirmation, and serve as the baseline biopsy, if confirmed as adequate per central pathology review. Copies of prior renal biopsy reports will be requested for review by the central pathology laboratory. For both adults and adolescents, if a prior renal biopsy is used for eligibility and/or will serve as the baseline biopsy, then images and/or slides may also be required. Tissue blocks and/or unstained slides may also be requested for staining at the central pathology laboratory.

Participants under the age of 18 may opt out of the baseline biopsy, provided they have an adequate prior renal biopsy to establish the diagnosis, even if the prior biopsy is more than 6 months prior to randomization. If the historical biopsy was more than 6 months prior to randomization or is not available, then the central medical reviewer will seek alternative evidence of ongoing complement activation as described in Section 12.1.2.

A follow-up renal biopsy will be performed at Week 26, which is required for subjects 18 years of age and older to advance to Part 3 of the study (Continuation Period). Subjects under the age of 18 who opt out of the biopsies, may still advance to Part 3 upon completion of all other Week 24 – Week 26 assessments.

The renal biopsy procedure should be performed according to the local standard practices at the site, including use of anesthesia/sedation, pre-procedure evaluation (eg, laboratory testing prior to renal biopsy), and post-procedure observation and monitoring. The renal biopsy must include adequate material for light microscopy (7 or more glomeruli), immunofluorescence microscopy (3 or more glomeruli) and electron microscopy (2 or more glomeruli). Renal biopsy samples should be handled and processed, as per the Pathology Manual. All renal pathology will be reviewed by the central pathology laboratory, which will be blinded to treatment assignment.

As a reminder, if a coagulation panel is performed for any reason, including as a pre-biopsy assessment, the use of silica reagents for this test should be avoided in patients treated with APL-2.

## 11.6. Ophthalmologic Evaluation

It is requested that patients have a baseline ophthalmologic evaluation, including spectral domain optical coherence tomography (SD-OCT), at an approved ophthalmologic clinical site at any time during the Screening Period. For those patients with drusen prior to APL-2 administration, a follow-up ophthalmologic evaluation, including an SD-OCT, is requested to occur at a convenient time point between Week 40 and Week 52 of the study. The SD-OCT will be transmitted to a central reading center for evaluation.

## 11.7. Measured Glomerular Filtration Rate

Measured glomerular filtration rate (GFR) is an optional assessment that may be done by a site that routinely performs this assessment. In this case, the site should follow their standard

procedure for measured GFR (and document the procedure within the eCRF) at 1 timepoint prior to the first APL-2 dose (but no more than 8 weeks prior to the first dose), and at 1 timepoint between Week 44 and Week 52, inclusive. In the event that retrospective measured GFR data are available, these should be collected for the same timeframe as retrospective data for calculation of eGFR (see Section 11.1).

## 11.8. Health-Related Quality of Life Assessments

Health-Related Quality of Life (HRQoL) assessments will be completed during clinic visits as outlined in the Schedule of Events (Table 1).

### 11.8.1. Functional Assessment of Illness Therapy Fatigue Scale

The Functional Assessment of Illness Therapy (FACIT) Fatigue scale is a 13 item Likert scaled instrument which is self-administered by subjects. Subject are presented with 13 statements and asked to indicate their responses as it applies to the past 7 days. The 5 possible responses are ‘Not at all’ (0), ‘A little bit (1), ‘Somewhat’ (2), ‘Quite a bit’ (3) and ‘Very much’ (4). With 13 statements the total score has a range of 0 to 52. Before calculating the total score, some responses are reversed to ensure that the higher score corresponds to a higher quality of life.

### 11.8.2. Short Form (36) Health Survey

The Short Form (36) Health Survey (SF-36) is a 36-item, patient-reported survey of patient health developed by the RAND Corporation. SF-36 is a set of generic, coherent, and easily administered quality-of-life measures. These measures rely upon patient self-reporting and are widely utilized by managed care organizations and health authorities for routine monitoring and assessment of care outcomes in adult patients.

### 11.8.3. 5-Level EuroQol-5 Dimension

The 5-Level EuroQol-5 Dimension (EQ-5D-5L) assessment is a standardized measure of health status developed to assess HRQoL by the EuroQoL Group. The questionnaire consists of a descriptive system and a visual analogue scale (VAS). The descriptive system contains 5 dimensions (mobility, self-care, usual activities, pain/discomfort, and anxiety/depression) with 5 levels in each dimension.

### 11.8.4. Kidney Disease Quality of Life

The Kidney Disease Quality of Life (KDQOL) survey is a kidney disease-specific measure of HRQoL developed by the RAND Corporation, which is self-administered by subjects. It includes a generic chronic disease core, and additional items relevant to patients with kidney disease, such as symptoms, burden of illness, social interaction, staff encouragement, and patient satisfaction. Scores are reported separately for each of 5 subscales.

# 12. ASSESSMENT OF SAFETY

## 12.1. Safety Parameters

### 12.1.1. Demographic/Medical History

Medical history will be recorded at screening. Investigators should document the occurrence, signs, and symptoms of the subject's preexisting conditions, including all prior significant illnesses, up to and including 1 year before screening. Additional pre-existing conditions, present at the time when informed consent is given, up to the time of first dosing, are to be regarded as concomitant. Medical history will include alcohol consumption and smoking history, if applicable.

A detailed medical history with regard to disease indication (C3G or IC-MPGN) will be requested, and will include prior surgeries, renal biopsies, hospitalizations, dialysis, transplantation, laboratory data, and extra-renal manifestations of the disease. This information will be recorded on a detailed disease-specific eCRF. Available disease medical history for the 3 years preceding screening will be requested. Submission of additional historical data is encouraged, when available. Copies of prior renal biopsy reports will be requested for review by the central pathology laboratory. If a prior renal biopsy is used for eligibility and/or will serve as the baseline biopsy, then images and/or slides may also be required. Tissue blocks or unstained slides may also be requested for staining at the central pathology laboratory.

Illnesses first occurring or detected during the study and/or worsening of a concomitant illness during the study are to be documented as AEs on the electronic case report form (eCRF) in accordance with Section 12.2.3.

Additionally, demographic data will be collected for all subjects, as allowed per applicable regulations.

### 12.1.2. Central Medical Review

As the proper diagnosis of primary C3G or IC-MPGN requires clinicopathologic correlation by a qualified medical expert, a central medical review will be implemented to ensure that the diagnosis is confirmed in each patient by an appropriately trained medical expert. The central medical reviewer will perform a comprehensive review of available clinical, laboratory and pathology data to confirm the diagnosis of primary C3G or IC-MPGN with ongoing complement activation. In adolescents in whom a renal biopsy within 6 months of Day 1 is not available, the central medical reviewer will seek alternative evidence for ongoing complement activation and/or active disease (eg, low serum C3, elevated plasma C5b-9 level, presence of hematuria). Details of the central medical review will be provided in a central medical review manual.

### 12.1.3. Vital Signs

Vital signs (body temperature, respiration rate, heart rate, and systolic and diastolic blood pressure measurements) will be evaluated at the visits indicated in the Schedule of Assessments (Table 1). All vital signs will be measured after the subject has been resting in a sitting position for at least 5 minutes, except when they are supine or semi-reclined because of study procedures

and/or AEs, or if deemed necessary by the Investigator. Blood pressure measurements are to be taken in the same arm for the duration of the study.

Vital signs measurements will be repeated, if clinically significant or if machine/equipment errors occur. Out-of-range blood pressure, respiratory rate, or heart rate measurements will be repeated, at the Investigator's discretion. Any confirmed, clinically significant vital signs measurements must be recorded as AEs.

When APL-2 is administered at the study site, vital signs will be measured within 2 hours prior to injection/infusion. Following the first infusion, vital signs will also be measured at 30 minutes (± 5 minutes) after dosing.

#### 12.1.4. Weight and Height

Body weight will be recorded at each visit. Height will be recorded at screening for adults and every 12 weeks throughout the study for adolescents. Both body weight and height will be assessed without shoes on.

#### 12.1.5. Physical Examination

All full physical examinations, performed by the Investigator or designee, will include, at a minimum, assessment of the following: general, head, ears, eyes, nose, throat, dentition, thyroid (endocrine), heart, chest, lungs, abdomen, skin, extremities, back/neck, musculoskeletal, and lymph nodes.

Brief physical examinations will include general appearance, heart, lungs, abdomen, extremities, and are to be performed at all visits where a full physical examination does not occur.

Edema will be assessed at each visit.

Additional symptom-driven physical examinations may be performed at any time, as deemed necessary by the Investigator.

See the Schedule of Assessments (Table 1) for the details regarding which type of physical examination should be given at each visit.

#### 12.1.6. Menses

For women of childbearing potential (WOCBP), the start and stop date of each menstrual period will be recorded in the eCRF for the duration of the study.

#### 12.1.7. Electrocardiogram (ECG)

All 12-lead ECGs will be measured once, prior to dosing, at the time points outlined in the Schedule of Assessments (Table 1). The ECG will be taken following resting in the supine position for 10 minutes in a quiet environment and prior to any blood sampling procedures, unless specified at time points after timed blood sampling procedures.

The ECGs will be classified as normal, having a not clinically significant abnormality, or having a clinically significant abnormality. In addition, ECG parameters of ventricular rate, PR interval, QRS duration, and QT internal (uncorrected and corrected using Fridericia's method [QTcF]) will be reviewed for eligibility and ongoing safety.

#### 12.1.8. Injection/Infusion Site/Pump Safety Assessment

On the days of clinic visits, pump use safety will be assessed within 30 minutes after study drug administration. This technique evaluation will be performed by a physician or other licensed healthcare provider (eg, study nurse), as delegated by the Investigator. The subject will be asked about any issues related to pump use.

Subjects will be instructed to notify the Investigator or other study personnel if an injection/infusion site reaction occurs after self-administration of APL-2. All clinically significant findings related to injection/infusion procedures will be recorded as AEs.

#### 12.1.9. Laboratory Assessments

Laboratory assessment samples (Table 3) are to be obtained at designated visits as detailed in the Schedule of Assessments (Table 1).

### Table 3: Laboratory Assessments

| **Hematology** | **Serum Chemistry** | **Urine Studies** |
|---|---|---|
| Hb<br>Hematocrit<br>Platelet count<br>RBC count<br>WBC count with differential | Albumin<br>ALT<br>ALP<br>AST<br>Bicarbonate<br>Bilirubin (total, direct and indirect)<br>BUN<br>Calcium<br>Chloride<br>Creatinine<br>Creatine kinase<br>Cystatin C<br>Estimated Glomerular Filtration Rate (using CKD-EPI formula for adults; Bedside Schwartz for adolescents)<br>GGT<br>Glucose<br>HDL<br>LDH<br>LDL<br>Phosphorus<br>Potassium<br>Sodium<br>Triglycerides<br>Total cholesterol<br>Total protein<br>Uric acid | Urinalysis<br>• Blood<br>• Bilirubin<br>• Glucose<br>• Ketones<br>• Leukocyte esterase<br>• Microscopic examination of urine sediment, including for presence of RBCs, WBCs, and casts, will be performed on all urinalyses<br>• Nitrite<br>• pH<br>• Pregnancy, when applicable<br>• Protein<br>• Specific gravity<br>• Urobilinogen<br><br>Spot Urines (First-Morning and Random)<br>• ACR<br>• BCR<br>• PCR<br>• Total albumin concentration<br>• Total B-2 microglobulin concentration<br>• Total creatinine concentration<br>• Total protein concentration<br><br>24-Hour Urine<br>• ACR<br>• BCR<br>• PCR<br>• Total albumin concentration<br>• Total B-2 microglobulin concentration<br>• Total creatinine concentration<br>• Total protein concentration<br>• Total volume |
| **Coagulation**[1] | **Additional** | |
| aPTT<br>Fibrinogen<br>INR | FSH (post-menopausal women)[2]<br>Hep B Panel[2, 3]<br>Hep C Panel[2, 4]<br>HIV antibodies[2]<br>SPEP[2]<br>ANA (with reflex anti-ds DNA)[2]<br>ANCA (with reflex anti-MPO and anti-PR3)[2] | Serum pregnancy test<br>Complement tests (serum/plasma) [5]<br>Anti-APL-2/PEG Antibody<br>Vaccination Antibody Titer[6] |

Abbreviations: ACR = albumin-to-creatinine ratio; ALP = alkaline phosphatase; ALT = alanine aminotransferase; ANA = anti-nuclear antibodies; ANCA = Antineutrophil cytoplasmic antibodies; aPTT = activated partial thromboplastin time; AST = aspartate aminotransferase; BCR = B-2 microglobulin:creatinine ratio; BUN = blood urea nitrogen; CKD-EPI = Chronic Kidney Disease – Epidemiology Collaboration; FSH = follicle stimulating hormone; GGT = gamma-glutamyltransferase; Hb = hemoglobin; HBsAg = Hepatitis-B surface antigen; HCV = hepatitis-C virus; HDL = high-density lipoproteins; Hib = *Haemophilus influenzae* type B vaccine; HIV = human immunodeficiency virus; INR = International Normalized Ratio; LDH = lactate dehydrogenase; LDL = low-density lipoproteins; MPO = myeloperoxidase; PCR = protein-to-creatinine ratio; PCV13 = Pneumococcal conjugate vaccine; PPSV23 = Pneumococcal polysaccharide vaccine 23; PR3 = proteinase 3; RBC = red blood cells; SOA = Schedule of Asseements; SPEP = Serum protein electrophoresis; WBC = white blood cells.

[1] The use of silica reagents in coagulation panels should be avoided in patients treated with APL-2.

[2] Screening only.
[3] HBsAg and HBsAb.
[4] Hep C Ab with reflex HCV RNA PCR
[5] Complement tests (serum/plasma): C3, C4, AH50, CH50, C3a, C3b/iC3b, C5a, and C5b-9.
[6] Serum samples will be collected for analysis of antibody titers to N. meningitidis (types A, C, W, Y, and B), PCV13 or PPSV23, and the Hib vaccination. Samples will only be analyzed in the event that the patient has a positive infection for Streptococcus pneumoniae, Neisseria meningitidis, and Haemophilus influenzae.

Blood and urine samples will be analyzed at a central or local laboratory facility, as defined in the Laboratory Manual. Urine samples will be analyzed by dipstick and a microscopic analysis. All laboratory reports must be reviewed, signed, and dated by the Investigator. A legible copy of all reports must be filed with both the subject's eCRF and medical record (source document) for that visit. Any laboratory test result considered by the Investigator to be clinically significant should be considered an AE (clinically significant AEs include those that require an intervention). Clinically significant abnormal values occurring during the study will be followed up until repeat test results return to normal, stabilize, or are no longer clinically significant.

#### 12.1.9.1. Pregnancy Screen

For WOCBP, a serum pregnancy test will be performed at screening. Subjects with a positive result will be excluded or discontinued from the study. A urine pregnancy test will also be performed starting at baseline and then at all study visits through the end of the study, including the follow-up visits. Male subjects will be counseled to avoid donating semen during the time between the first screening and the final Week 76 exit visit and for the 90 days after their last dose of study drug.

### 12.1.10. Pharmacokinetic Assessment

Blood samples for PK assessment of APL-2 will be collected before dosing via direct venipuncture at the time points delineated in the Schedule of Events (Table 1).

Instructions for collection, handling, processing, storage, and shipping of samples will be provided in a separate Laboratory Reference Manual prior to study initiation.

The concentration of study drug will be determined from the serum samples using a validated analytical method. Details of the method validation and sample analysis will be included in the final clinical study report.

### 12.1.11. Anti-APL-2 and Anti-PEG Antibody Assessment

The proposed antidrug antibodies (ADA) sampling schedule was established to capture the ADA signal at baseline, along with any potential early onset and the dynamic profile (transient or persistent) of antibody formation while minimizing APL-2 level in the sample.

Subjects who exhibit a treatment-boosted response for anti-APL-2 or anti-PEG antibodies in the last dosed sample will be followed with ADA samples being collected approximately every 6 months until the antibody levels revert to baseline. Samples that test positive will be characterized by an assay that will determine antibody titer and measure neutralizing capacity.

Subjects will need to have ADA samples collected as outlined in the Schedule of Assessments.

## 12.2. Adverse and Serious Adverse Events

### 12.2.1. Definition of Adverse Events

#### 12.2.1.1. Adverse Event (AE)

An AE is any untoward medical occurrence associated with the use of a drug in humans, whether or not it is considered drug-related. An AE can, therefore, be any unfavorable and unintended sign, including a clinically significant abnormal laboratory finding, symptom, or disease temporally associated with the use of a study drug, whether or not considered related to the study drug (FDA Guidance for Industry: Clinical Safety Data Management: Definitions and Standards for Expedited Reporting (ICH E2A)). Any abnormal finding that is deemed not clinically significant is not an AE.

Adverse events include the onset of new illness and the exacerbation of preexisting conditions. Any medical condition that is present at the time when the subject is screened should be recorded on the medical history eCRF and not reported as an AE. However, if that condition deteriorates or severity changes at any time during the study, it should be recorded as an AE.

Any AEs that occur prior to dosing on Study Day 1 will be categorized as pre-treatment events. Treatment-emergent adverse events will be defined as those AEs that occur after initial dosing, or worsen in severity, and up to 30 days after the last dose of APL-2.

A suspected adverse reaction means any AE for which there is a reasonable possibility that the drug caused the AE. Reasonable possibility means there is evidence to suggest a causal relationship between the drug and the AE.

#### 12.2.1.2. Serious Adverse Event (SAE)

If any AEs are serious, special procedures will be followed. All serious adverse events (SAEs) will be reported to the Safety Monitor by the Investigator via eCRF within 24 hours of becoming aware of the event, whether or not the SAEs are deemed drug-related. Serious adverse event reporting contact information will be provided separately and as included in the Serious Adverse Event report form/guidelines. All SAEs must be reported to the applicable ethics committee by the Investigator, in accordance with their regulations.

An SAE is any AE or suspected adverse reaction that, in the view of either the Investigator or Sponsor, results in any of the following outcomes: death; a life-threatening AE; inpatient hospitalization or prolongation of existing hospitalization; a persistent or significant incapacity or substantial disruption of the ability to conduct normal life functions; or a congenital anomaly/birth defect. Important medical events that may not result in death, be life-threatening, or require hospitalization may be considered serious when, based upon appropriate medical judgment, they may jeopardize the subject and may require medical or surgical intervention to prevent one of the outcomes listed in the above definition. Examples of such medical events may include a diagnosis of sepsis that requires antibiotic treatment, allergic bronchospasm requiring intensive treatment in an emergency room or at home, blood dyscrasias or convulsions that do not result in inpatient hospitalization, or the development of drug dependency or drug abuse.

*Life-threatening* is defined as an AE or suspected adverse reaction, which, in the view of either the Investigator or Sponsor, places the subject at immediate risk of death. It does not include an

AE or suspected adverse reaction that, had it occurred in a more severe form, might have caused death.

*Unexpected* is defined as an AE or suspected adverse reaction that is not listed in the IB; or is not listed at the specificity or severity that has been observed; or is not consistent with the risk information described in the general investigational plan or elsewhere in the current application, as amended.

#### 12.2.2. Relationship to Study Drug

All AEs that occur during this study will be recorded. The Investigator will review each event and assess its relationship to study drug treatment (unknown, not related, unlikely related, possibly related, and definitely related). The date and time of onset, time relationship to drug dosing, duration, and outcome (recovered/resolved, recovered/resolved with sequelae, recovering/resolving, not recovered/not resolved, fatal, or unknown) of each event will be noted.

Table 4 should be considered when evaluating the relationship of AEs/SAEs to study treatment.

**Table 4: Definitions of Adverse Event Relatedness**

| Classification | Definition |
|---|---|
| DEFINITELY RELATED | Strong evidence of a causal relationship; the influence of other factors is unlikely. |
| POSSIBLY RELATED | Some evidence of a causal relationship, but other factors may have caused or contributed to the event (eg, another illness or concomitant treatment) |
| UNLIKELY RELATED | A causal relationship is not a reasonable possibility, but it cannot be completely ruled out with the available evidence. |
| NOT RELATED | No evidence of a causal relationship |
| UNKNOWN | Temporal relationship exists between study drug and the event, but more data is needed for a medical assessment |

Table 5 presents the definitions that should be considered when evaluating the severity of AEs and SAEs.

**Table 5: Severity of Events**

| Severity | Definition/Description |
|---|---|
| Mild | Event results in mild or transient discomfort, not requiring intervention or treatment; does not limit or interfere with daily activities (eg, insomnia, mild headache). |
| Moderate | Event is sufficiently discomforting, so as to limit or interfere with daily activities; may require interventional treatment (eg, febrile illness requiring oral medication). |
| Severe | Event results in significant symptoms that prevents normal daily activities; may require hospitalization or invasive intervention (eg, anemia resulting in blood transfusion). |

### 12.2.3. Recording Adverse Events

Adverse events spontaneously reported by the subject and/or in response to an open question from the study personnel or revealed by observation will be recorded during the study at the investigational site. Clinically significant changes in laboratory values, blood pressure, and pulse need not be reported as AEs. However, abnormal values that constitute an SAE or lead to discontinuation of administration of study drug must be reported and recorded as an AE. AEs and SAEs will be collected from the signing of the consent form until the Exit/ET visit, 6 weeks after the last dose of APL-2. The AE term should be reported in standard medical terminology when possible. For each AE, the investigator will evaluate and report the onset (date and time), resolution (date and time), intensity, causality, action taken, serious outcome (if applicable), and whether or not it caused the subject to discontinue the study.

It is important to distinguish between serious and severe AEs. Severity is a measure of intensity whereas seriousness is defined by the criteria under Section 12.2.1.2. An AE of severe intensity may not be considered serious.

Should a pregnancy occur, it must be reported and recorded on the Apellis pregnancy form. Pregnancy in itself is not regarded as an AE unless there is a suspicion that an investigational product may have interfered with the effectiveness of a contraceptive medication.

The outcome of all pregnancies (spontaneous miscarriage, elective termination, normal birth or congenital abnormality) must be followed up and documented even if the subject was discontinued from the study.

All reports of congenital abnormalities/birth defects are SAEs. Spontaneous miscarriages should also be reported and handled as SAEs. Elective abortions without complications should not be handled as AEs.

### 12.2.4. Reporting Adverse Events

All SAEs and pregnancies must be reported to the Sponsor via eCRF within 24 hours of becoming aware of the events, whether or not the event is deemed treatment-related. If the electronic data capture (EDC) system is not operational, the site must complete the appropriate paper SAE form and fax/email to the number/address listed on the SAE form, also within 24 hours of becoming aware of the event. The reported information submitted as a paper SAE must be entered into the EDC system once it becomes operational.

The collection of clinical information will begin after the subject's written consent to participate in the study has been obtained. Adverse events will be collected after signing the informed consent form (ICF) through completion of the exit visit. Any events that occur prior to dosing will be categorized as pre-treatment events; events occurring after dosing will be recorded as TEAEs (start date of dosing and, therefore, categorization of the event will be dependent on randomization assignment). Adverse events may be either spontaneously reported or elicited during questioning and examination of a subject.

All identified AEs, including clinically significant laboratory findings, must be recorded and described on the appropriate AE or SAE page of the eCRF. If known, the diagnosis of the underlying illness or disorder should be recorded, rather than its individual symptoms. Adverse

events will be coded in accordance with the Medical Dictionary for Regulatory Activities (MedDRA®).

Subjects experiencing AEs that cause interruption or discontinuation of study drug, or those experiencing AEs that are present at the exit visit should receive follow-up, as appropriate. If possible, the outcome of any AE that caused permanent discontinuation or was present at the end of the study should be reported, particularly if the AE was considered by the Investigator to be related to the study drug.

The Sponsor and/or its designee are responsible for reporting SAEs to all applicable regulatory agencies and the central ethics committees within the required timeline.

The Investigators are responsible for submitting required safety information to their local Institutional Review Board (IRB) or Independent Ethics Committee (IEC) as per local regulations. This information includes, but is not limited to, any safety alert letter received from the Sponsor and any SAEs occurring at their investigative site.

## 12.3. Pregnancy

Although pregnancy is not considered an AE, the outcome of a pregnancy, if there is a spontaneous abortion, congenital anomaly, or other adverse fetal outcome, may be an SAE. All SAEs are to be reported to the study Sponsor on the SAE Reporting Form.

WOCBP and male subjects with female partners of child-bearing potential will be instructed to practice an acceptable method of birth control (see Section 12.3.1) throughout the duration of the study and for 90 days following the end of APL-2 administration.

If a female subject/partner of a male subject becomes pregnant during the study, the Investigator should report the pregnancy to the Safety Monitor within 1 calendar day of being notified. The subject or partner should be followed by the Investigator until completion of the pregnancy. At the completion of the pregnancy, the Investigator will document and report the outcome. If the outcome of the pregnancy meets the criteria for classification as an SAE (ie, postpartum complication, stillbirth, neonatal death, or congenital anomaly) the Investigator should follow the procedures for reporting an SAE (Section 12.2.4).

### 12.3.1. Acceptable Methods of Contraception

Approved methods of contraception include:

- Combined (estrogen and progestogen containing) hormonal contraception associated with inhibition of ovulation:
    - oral, intravaginal, or transdermal
- Progestogen-only hormonal contraception associated with inhibition of ovulation:
    - oral, injectable, or implantable
- Intrauterine device (IUD)
- Intrauterine hormone-releasing system (IUS)
- Bilateral tubal occlusion

- Vasectomized partner (*provided that partner is the sole sexual partner of the WOCBP trial participant and that the vasectomized partner has received medical assessment of the surgical success*)
- Sexual abstinence (*defined as refraining from heterosexual intercourse during the entire period of risk associated with study treatments*)
- Male condom with or without spermicide (for male study patients with female partners of childbearing potential only)

Not all methods of contraception may be available in all of the countries in which this study is being conducted.

Note: Sexual abstinence is only accepted when it is the preferred and usual lifestyle of the patient.

Patients must agree to use an approved method of contraception during the study and for 90 days after their last dose of study drug.

# 13. STATISTICS

A formal statistical analysis plan (SAP) will be developed and finalized prior to locking the database. The full details of data presentations and analyses will be provided in the SAP. Additional statistical analyses other than those described in this section may be performed if deemed appropriate and included in the SAP. Any deviations from the final SAP or from what is outlined in the protocol will be discussed in the final study report.

## 13.1. Determination of Sample Size

Assuming a difference in the change from baseline of -1.30 mg/mg in uPCR at Week 26 between treatment groups with a standard deviation of 1.67 mg/mg, a total of 36 subjects per treatment group is required for 90% power at the significance level of 0.025 (one-sided) using a two-sample t-test with equal allocation to treatment groups. These assumptions regarding the difference in the change from baseline and standard deviation are based on 11 patients from the APL2-201 Phase 2 study.

A total of 90 subjects will be randomized to account for an anticipated 20% of randomized subjects prematurely discontinuing the study without providing a post-baseline uPCR measurement.

A sample size re-estimation based on the conditional power approach without adjustment of Type I error rate may be considered. The sole purpose of the planned sample size re-estimation (which may take place when approximately 50% of all patients have completed the Week 26 visit or withdrew prior to the Week 26 visit) is to adjust the sample size, which will be changed only under certain conditions (details will be provided in the interim analysis SAP). This assessment will not lead to a reduction in the sample size. The sample size of the study may be increased to a maximum of 120. An increase beyond this maximum would require a protocol amendment.

## 13.2. Analysis Set

### 13.2.1. Screened Set

The screened set will include all subjects who provide written informed consent. This set will be used only for the purpose of describing subject disposition.

### 13.2.2. Safety Set

The safety set will include all subjects who receive at least 1 dose of APL-2, or subjects who are randomized to no APL-2 treatment. Subjects will be analyzed according to the treatment they received.

### 13.2.3. Intent-to-Treat Set

The intent-to-treat (ITT) set will include all randomized subjects. Subjects will be analyzed according to their assigned treatment, regardless of the treatment actually received.

### 13.2.4. Modified Intent-to-Treat Set

The modified intent-to-treat (mITT) set will include all subjects in the ITT set who continue study treatment beyond Week 4.

### 13.2.5. Per Protocol Set

The per protocol (PP) set will include all subjects in the ITT set who have not violated any inclusion or exclusion criteria and/or deviated from the protocol in a way that could influence their efficacy assessment. Decisions concerning the exclusion of subjects from the PP analysis set will be made and documented prior to database lock.

### 13.2.6. Pharmacokinetic Set

The pharmacokinetic (PK) set will include all subjects in the ITT set who receive APL-2 and have at least 1 evaluable postdose PK measurement.

### 13.2.7. Pharmacodynamic Set

The pharmacodynamic (PD) set will include all subjects in the ITT set who receive APL-2 and have at least 1 evaluable postdose PD measurement.

### 13.2.8. Data Review for Analysis Set

After all the data have been verified/coded/entered into the database, a review will be performed. The purpose of this review will be to define the analysis populations. The review will also check the quality of the data, identifying outliers, and make decisions on how to deal with any data issues (eg, missing values, withdrawals, protocol deviations). After the pre-analysis review, resolution of all issues and documentation of all decisions, the database will be locked.

## 13.3. Efficacy Analysis

### 13.3.1. Analysis of Primary Efficacy Endpoint

The change from baseline in uPCR will be calculated using the following steps:

1. Baseline uPCR value will be calculated as the average of up to 9 uPCR measurements preceding and including Day 1.
2. Week 26 uPCR value will be calculated as the average of up to 9 uPCR measurements preceding and including Week 26.

The between-treatment group comparison for the primary efficacy endpoint will be performed using a mixed effect model for repeated measures (MMRM) (Mallinckrodt, 2008). The model will include fixed categorical effects for treatment group, study visit, stratification factors and the study visit-by-treatment group interaction, as well as the continuous, fixed covariate of baseline uPCR level. An unstructured covariance matrix will be used. The difference between treatment groups will be estimated, along with its 97.5% one-sided CI and corresponding p-value.

The following sensitivity analyses will be provided:

- Control-based imputation

- Tipping point analysis
- Multiple imputations (MI) assuming missing at random

Sensitivity analyses will be detailed in the SAP.

Supporting Analyses will be repeated using the PP set and mITT set.

Subgroup analyses will also be conducted to describe findings within both stratification factors separately.

### 13.3.2. Analysis of Secondary Efficacy Endpoint

To preserve the Type 1 error a fixed sequence testing strategy will be used. The ordering of the secondary endpoints in this testing strategy will match the order in which they are presented in Section 6.2.2.

- The proportion of patients who achieve at least a 30% reduction in uPCR will be calculated using the following steps:
  1. Baseline uPCR value will be calculated as the average of up to 9 uPCR measurements preceding and including Day 1.
  2. Week 26 uPCR value will be calculated as the average of up to 9 uPCR measurements preceding and including Week 26.
  3. Patients will be determined as either success or failures based on whether the change from baseline in uPCR calculated from steps 1 and 2 achieves at least a 30% reduction.

  The proportion of patients who achieve at least a 30% reduction in uPCR from baseline at Week 26 will be analyzed using mixed-model for repeated measures (MMRM) with the effects of treatment; visit, visit-by treatment interaction; the relevant baseline level; and the baseline level-by-visit interaction using an unstructured covariance matrix. The odds ratio of achieving at least a 30% reduction in uPCR from baseline at Week 26 for the APL-2 Arm versus the No APL-2 Arm and associated 97.5% one-sided CI will be provided.
- The number and proportion of patients with serum albumin above the lower limit of normal, for those in whom the parameter was below the lower limit of normal at baseline, will be calculated using the following steps:
  1. Baseline serum albumin value will be calculated as the average of up to 2 serum albumin measurements preceding and including Day 1. Patients will only be included in the denominator of this calculation if the baseline albumin value is below the lower limit of normal.
  2. Week 26 serum albumin value will be calculated as the average of up to 2 serum albumin measurements preceding and including Week 26.
  3. Patients will be determined as either success or failures based on whether the Week 26 serum albumin value as calculated in Step 2 is above the lower limit or normal.

The number and proportion of patients with serum albumin above the lower limit of normal at Week 26, for those in whom the parameter was below the lower limit of normal at Baseline, will be tabulated by treatment group and compared between treatment groups using Fisher's exact test. The odds ratio of showing serum albumin above the lower limit of normal at Week 26 for the APL-2 Arm versus the No APL-2 Arm and associated 97.5% one-sided CI will be provided.

- The proportion of patients who achieve at least a 50% reduction in uPCR will be calculated using the following steps:
  1. Baseline uPCR value will be calculated as the average of up to 9 uPCR measurements preceding and including Day 1.
  2. Week 26 uPCR value will be calculated as the average of up to 9 uPCR measurements preceding and including Week 26.
  3. Patients will be determined as either success or failures based on whether the change from baseline in uPCR calculated from steps 1 and 2 achieves at least a 50% reduction.

  The proportion of patients who achieve at least a 50% reduction in uPCR from baseline at Week 26 will be analyzed using mixed-model for repeated measures (MMRM) with the effects of treatment; visit, visit-by treatment interaction; the relevant baseline level; and the baseline level-by-visit interaction using an unstructured covariance matrix. The odds ratio of showing at least a 50% reduction in uPCR from baseline at Week 26 for the APL-2 Arm versus the No APL-2 Arm and associated 97.5% one-sided CI will be provided.
- The change from baseline in uPCR at Week 52 will be calculated using the following steps:
  1. Baseline uPCR value will be calculated as the average of up to 9 uPCR measurements preceding and including Day 1.
  2. Week 52 uPCR value will be calculated as the average of up to 9 uPCR measurements preceding and including Week 52.

  The change from baseline in uPCR at Week 52 will be analyzed using a one-sample t-test (APL-2 arm). This difference will be estimated, along with its 97.5% one-sided CI and corresponding p-value.
- A matched pair t-test will be conducted to compare the durability of treatment between the uPCR reduction from baseline to Week 26 and uPCR reduction from Week 26 to Week 52. The difference between uPCR reduction at the end of the Continuation Period and uPCR reduction at the end of Randomized Treatment Period will be estimated, along with its 97.5% one-sided CI and p-value.
- A matched paired t-test will be conducted to compare the change in eGFR from historical eGFR (up to 3 years prior to screening) to baseline and change in eGFR from baseline to Week 52. The difference will be estimated, along with its 97.5% one-sided CI and p-value.

- The number and proportion of patients who show a reduction in C3c staining on renal biopsy from baseline to Week 26 will be tabulated by treatment group and compared between treatment groups using Fisher's exact test. The odds ratio of showing a reduction from baseline in C3c staining on renal biopsy at Week 26 for the APL-2 Arm versus the No APL-2 Arm and associated 97.5% one-sided CI will be provided.
- The change from Week 26 in uPCR at Week 52 will be calculated using the following steps:
  1. Week 26 uPCR value will be calculated as the average of up to 9 uPCR measurements preceding and including Week 26.
  2. Week 52 uPCR value will be calculated as the average of up to 9 uPCR measurements preceding and including Week 52.

  The change from Week 26 in uPCR at Week 52 will be analyzed using a one-sample t-test. This difference will be estimated, along with its 97.5% one-sided CI and corresponding p-value.

## 13.4. Safety Analysis

All safety analyses will be summarized for the Safety Set.

Adverse events will be coded using MedDRA. All AEs, including TEAEs, will be summarized by System Organ Class, Preferred Term, treatment group for number of subjects and proportion reporting the event. A similar summary will be produced for SAEs, AEs leading to termination, severe AEs, and AEs related to the investigational product. The intensity of AEs and the relationship to investigational product will be summarized for each System Organ Class and Preferred Term by treatment group.

Withdrawals due to AEs will be summarized for each body system and Preferred Term by treatment group.

Laboratory assessments, anti-APL-2/PEG antibodies, and ECGs will be summarized by treatment group using appropriate descriptive statistics.

Treatment emergent adverse events are defined as those AEs that develop or worsen after the first dose of study medication and up to 30 days beyond the last dose of study medication. The number and incidence of TEAEs (both serious and non-serious), as well as the number and incidence of discontinuations due to TEAEs will be tabulated.

The AE summaries will be presented across all subjects. All AEs will be listed by subject, along with information regarding onset, duration, relationship, and severity to study drug, action taken with study drug, treatment of event, and outcome. In addition, the number and incidence of rejection episodes and graft loss will be tabulated.

Changes from baseline in clinical laboratory tests will be summarized, using descriptive statistics, by visit and nominal time postdose. Baseline will be taken as the measurement closest, but prior, to randomization. Out-of-range values will be flagged in data listings.

Changes from baseline in vital signs and ECG parameters will be summarized, using descriptive statistics, by treatment, visit and nominal time postdose. Baseline will be taken as the measurement closest, but prior, to randomization.

Values of potential clinical significance (eg, increase in QTcF ≥ 30 ms from baseline) will be flagged in listings and summarized by treatment.

ECG parameters will be analyzed using concentration effect models.

Changes in physical examinations will be described in a data listing.

## 13.5. Pharmacokinetic Analysis

The PK concentrations will be evaluated using the PK set.

Concentrations will be summarized, using descriptive statistics, over time, in the randomized treatment group (APL-2 group).

Individual subject concentration-time data will be plotted against actual sampling time. Median profiles of the concentration-time data, using nominal sampling times, will also be presented. Both linear-linear and linear-log plots will be presented.

Population PK and exposure-response modeling of the safety and efficacy data will be described in an APL-2 Population PK/PD Analysis Plan.

## 13.6. Pharmacodynamic Analysis

The PD endpoints will be evaluated using the PD set.

Absolute values, changes from baseline and percentage changes from baseline will be summarized using descriptive statistics, over time by treatment group.

Individual subject time profiles will be plotted against actual sampling time. Median profiles, over time, using nominal sampling time, will also be presented.

The PD endpoints will be compared between treatment groups using mixed effect repeated measures analyses.

## 13.7. Other Analyses

Demographics, baseline characteristics, concomitant medication, medical history, and study medication exposure will be summarized by treatment group.

World Health Organization (WHO) and MedDRA coding dictionaries will be used for the concomitant medications and medical histories, respectively.

## 13.8. Interim Analysis

No formal interim analyses are planned for the primary endpoint; however, a sample size re-estimation may be conducted when approximately 50% of all patients have completed the Week 26 Visit or withdrew prior to the Week 26 Visit. In addition, data will be reported once all subjects have completed their Week 26 Visit and the database has been cleaned for all visits up to and including Week 26.

## 13.9. Data Monitoring Committee

A formal independent data monitoring committee (DMC) will be used for this study. In addition to the Sponsor's medical monitor, external medical and scientific experts will be invited to participate in the reviews, as needed.

DMC meetings will be held according to the schedule in the DMC Charter. The remit, roles, and responsibilities of the DMC will be specified in a DMC charter.

# 14. DIRECT ACCESS TO SOURCE DATA/DOCUMENTS

## 14.1. Study Monitoring

Before an investigational site can enter a patient into the study, a representative of Apellis Pharmaceuticals, Inc. will visit the investigational study site to:

- Determine the adequacy of the facilities
- Discuss with the investigator(s) and other personnel their responsibilities with regard to protocol adherence, and the responsibilities of Apellis Pharmaceuticals, Inc. or its representatives. This will be documented in a Clinical Study Agreement between Apellis Pharmaceuticals, Inc. and the investigator.

During the study, a monitor from Apellis Pharmaceuticals, Inc. or representative will have regular contacts with the investigational site, for but not limited to the following:

- Provide information and support to the investigator(s)
- Confirm that facilities remain acceptable
- Confirm that the investigational team is adhering to the protocol, that data are being accurately recorded in the case report forms, and that investigational product accountability checks are being performed
- Perform source data verification. This includes a comparison of the data in the case report forms with the patient's medical records at the hospital or practice, and other records relevant to the study. This will require direct access to all original records for each patient (eg, clinic charts).
- Record and report any protocol deviations not previously sent to Apellis Pharmaceuticals, Inc..
- Confirm AEs and SAEs have been properly documented on CRFs and confirm any SAEs have been forwarded to Apellis Pharmaceuticals, Inc. and those SAEs that met criteria for reporting have been forwarded to the IRB.

The monitor will be available between visits if the investigator(s) or other staff needs information or advice.

## 14.2. Audits and Inspections

Authorized representatives of Apellis Pharmaceuticals, Inc., a regulatory authority, an Independent Ethics Committee or an Institutional Review Board may visit the site to perform audits or inspections, including source data verification. The purpose of an Apellis Pharmaceuticals, Inc. audit or inspection is to systematically and independently examine all study-related activities and documents to determine whether these activities were conducted, and data were recorded, analyzed, and accurately reported according to the protocol, Good Clinical Practice guidelines of the International Conference on Harmonisation, and any applicable regulatory requirements. The investigator should contact Apellis Pharmaceuticals, Inc. immediately if contacted by a regulatory agency about an inspection.

## 14.3. Institutional Review Board

The Principal Investigator must obtain IRB approval for the investigation. Initial IRB approval, and all materials approved by the IRB for this study including the patient consent form and recruitment materials must be maintained by the Investigator and made available for inspection.

# 15. QUALITY CONTROL AND QUALITY ASSURANCE

To ensure compliance with Good Clinical Practices and all applicable regulatory requirements, Apellis Pharmaceuticals, Inc. may conduct a quality assurance audit. Please see Section 14.2 for more details regarding the audit process.

# 16. ETHICS

## 16.1. Ethics Review

The final study protocol, including the final version of the informed consent form, must be approved or given a favorable opinion in writing by an IRB or IEC as appropriate. The investigator must submit written approval to Apellis Pharmaceuticals, Inc. before he or she can enroll any patient/subject into the study.

The Principal Investigator is responsible for informing the IRB or IEC of any amendment to the protocol in accordance with local requirements. In addition, the IRB or IEC must approve all advertising used to recruit patients for the study. The protocol must be re-approved by the IRB or IEC upon receipt of amendments and annually, as local regulations require.

The Principal Investigator is also responsible for providing the IRB with reports of any reportable serious adverse drug reactions from any other study conducted with the investigational product. Apellis Pharmaceuticals, Inc. will provide this information to the Principal Investigator.

Progress reports and notifications of serious adverse drug reactions will be provided to the IRB or IEC according to local regulations and guidelines.

## 16.2. Ethical Conduct of the Study

The study will be performed in accordance with ethical principles that have their origin in the Declaration of Helsinki and are consistent with ICH/Good Clinical Practice, applicable regulatory requirements and the Apellis Pharmaceuticals, Inc.'s policy on Bioethics.

## 16.3. Written Informed Consent

The Principal Investigator(s) at each center will ensure that the patient is given full and adequate oral and written information about the nature, purpose, possible risk and benefit of the study. Patients must also be notified that they are free to discontinue from the study at any time. The patient should be given the opportunity to ask questions and allowed time to consider the information provided.

The patient's signed and dated informed consent must be obtained before conducting any study procedures.

The Principal Investigator(s) must maintain the original, signed informed consent form. A copy of the informed consent form must be given to the patient.

# 17. DATA HANDLING AND RECORDKEEPING

## 17.1. Inspection of Records

Apellis Pharmaceuticals, Inc. will be allowed to conduct site visits to the investigation facilities for the purpose of monitoring any aspect of the study. The Investigator agrees to allow the monitor to inspect the drug storage area, study drug stocks, drug accountability records, subject charts and study source documents, and other records relative to study conduct.

## 17.2. Retention of Records

The Principal Investigator must maintain all documentation relating to the study for a period of 2 years after the last marketing application approval, or if not approved 2 years following the discontinuance of the test article for investigation. If it becomes necessary for Apellis Pharmaceuticals, Inc. or the Regulatory Authority to review any documentation relating to the study, the Investigator must permit access to such records.

# 18. PUBLICATION POLICY

Both the use of data and the publication policy are detailed within the clinical study agreement. Intellectual property rights (and related matters), generated by the Investigator and others performing the clinical study, will be subject to the terms of a clinical study agreement that will be agreed between the institution and the Sponsor or its designee. With respect to such rights, the Sponsor or its designee will solely own all rights and interests in any materials, data, and intellectual property rights developed by Investigators and others performing the clinical study described in this protocol, subject to the terms of any such agreement. In order to facilitate such ownership, Investigators will be required to assign all such inventions, either to their institution or directly to the Sponsor or its designee, as will be set forth in the clinical study agreement.

# 19. LIST OF REFERENCES

Apellis Pharmaceuticals Inc. APL-2 Investigator's Brochure. 26 August 2019.

Bomback AS, Santoriello D, Avasare RS, et al. C3 glomerulonephritis and dense deposit disease share a similar disease course in a large United States cohort of patients with C3 glomerulopathy. Kidney Int. 2018 Apr;93(4):977-985.

Cook HT. Evolving complexity of complement-related diseases: C3 glomerulopathy and atypical haemolytic uremic syndrome. Curr Opin Nephrol Hypertens. 2018 May;27(3):165-170.

FDA Guidance for Industry: Clinical Safety Data Management: Definitions and Standards for Expedited Reporting (ICH E2A). Mar 1995 (https://www.fda.gov/media/71188/download).

Holle J, Berenberg-Goßler L, Wu K, et al. Outcome of membranoproliferative glomerulonephritis and C3-glomerulopathy in children and adolescents. Pediatr Nephrol. 2018 Dec;33(12):2289-2298.

Iatropoulos P, Daina E, Curreri M, et al. Cluster Analysis Identifies Distinct Pathogenetic Patterns in C3 Glomerulopathies/Immune Complex-Mediated Membranoproliferative GN. J Am Soc Nephrol. 2018 Jan;29(1):283-294.

Lu DF, Moon M, Lanning LD, et al. Clinical features and outcomes of 98 children and adults with dense deposit disease. Pediatr Nephrol. 2012 May;27(5):773-81.

Mager DE, Jusko WJ. General pharmacokinetic model for drugs exhibiting target-mediated drug disposition. J Pharmacokinet Pharmacodyn. 2001 Dec;28(6):507-32.

Mallinckrodt CH, Lane PW, Schnell D, et al. Recommendations for the Primary Analysis of Continuous Endpoints in Longitudinal Clinical Trials. Drug Inf J. 2008 Jul;42(4):303-319.

Medjeral-Thomas NR, O'Shaughnessy MM, O'Regan JA, et al. C3 glomerulopathy: clinicopathologic features and predictors of outcome. Clin J Am Soc Nephrol. 2014 Jan;9(1):46-53.

Noris M, Donadelli R, Remuzzi G. Autoimmune abnormalities of the alternative complement pathway in membranoproliferative glomerulonephritis and C3 glomerulopathy. Pediatr Nephrol. 2019 Aug;34(8):1311-1323.

Okpechi IG, Dlamini TA, Duffield M, et al. Outcome of patients with primary immune-complex type mesangiocapillary glomerulonephritis (MCGN) in Cape Town South Africa. PLoS One. 2014 Nov 20;9(11):e113302.

Pickering MC, D'Agati VD, Nester CM, et al. C3 glomerulopathy: consensus report. Kidney Int. 2013 Dec;84(6):1079-89.

Regunathan-Shenk R, Avasare RS, Ahn W, et al. Kidney Transplantation in C3 Glomerulopathy: A Case Series. Am J Kidney Dis. 2019 Mar;73(3):316-323.

Servais A, Noël LH, Roumenina LT, et al. Acquired and genetic complement abnormalities play a critical role in dense deposit disease and other C3 glomerulopathies. Kidney Int. 2012 Aug;82(4):454-64.

Smith RJH, Appel GB, Blom AM, et al. C3 glomerulopathy - understanding a rare complement-driven renal disease. Nat Rev Nephrol. 2019 Mar;15(3):129-143.

Zhang Y, Nester CM, Martin B, et al. Defining the complement biomarker profile of C3 glomerulopathy. Clin J Am Soc Nephrol. 2014 Nov 7;9(11):1876-82.

# PEGCETACOPLAN (APL-2)

# APL2-C3G-310

# A PHASE 3, RANDOMIZED, PLACEBO-CONTROLLED, DOUBLE-BLINDED, MULTICENTER STUDY TO EVALUATE THE EFFICACY AND SAFETY OF PEGCETACOPLAN IN PATIENTS WITH C3 GLOMERULOPATHY OR IMMUNE-COMPLEX MEMBRANOPROLIFERATIVE GLOMERULONEPHRITIS

| | |
|---|---|
| **Regulatory Agency Identifier Number(s)**<br>IND: 136409<br>EudraCT: 2020-003767-25 | **Phase: 3** |
| | **Version:** Amendment 4 |
| | **Approval Date:** 25 April 2024 |

**Confidentiality Statement**

This document is confidential. It contains proprietary information of Apellis Pharmaceuticals, Inc. Any viewing or disclosure of such information that is not authorized in writing by Apellis Pharmaceuticals, Inc, is strictly prohibited. Such information may be used solely for the purpose of reviewing or performing this study.

# SIGNATURE PAGE

## Sponsor's Approval

The protocol has been approved by Apellis Pharmaceuticals, Inc.

**Responsible Medical Director:**

PPD

25-Apr-2024 | 14:12 EDT

Date

PPD

Medical Director, Nephrology

Apellis Pharmaceuticals, Inc

PPD

# INVESTIGATOR'S AGREEMENT

I have read the Study APL2-C3G-310 protocol and agree to conduct the study as outlined. I agree to maintain the confidentiality of all information received or developed in connection with this protocol.

______________________________________

Printed Name of Investigator

______________________________________

Signature of Investigator

___________________

Date

# PROTOCOL AMENDMENT SUMMARY OF CHANGES

## Protocol Amendment 4

## Overall Rationale for the Amendment:

The protocol was amended to revise the order of the key secondary endpoints in response to updated knowledge from the nephrology field. Intercurrent event (ICE) strategies for the statistical analysis of the endpoints were updated to align with comments from the FDA. The strategy for closing enrollment was clarified. Additional revisions were made to clarify study procedures and activities in response to feedback from investigators and/or to align with current company practices.

Minor formatting and stylistic revisions were made to align with current templates, and any identified typographical errors were corrected; these are not identified individually in the descriptions below.

| Description of change | Section(s) affected | Rationale for change |
|---|---|---|
| **Study endpoint changes** | | |
| Order of key secondary endpoints revised; change in eGFR at 26 weeks now the last key secondary endpoint | Synopsis<br>Section 5.2.1<br>Section 12.3.2.1 | Based on emerging data and recent expert discussions, histopathologic changes are expected to occur before improvements in eGFR are observed. The order of the key secondary endpoints was amended to prioritize the histopathologic endpoints over the eGFR endpoint. |
| Removed exploratory endpoint evaluating normalization of hematuria | Section 5.2.1 | No post-baseline hematuria evaluations will be conducted. |
| Removed exploratory endpoint of evaluation of changes in drusen at 26 weeks | Section 5.2.1 | If conducted, ophthalmologic evaluations are at baseline and between weeks 42 and 52 only. |
| **Study assessment changes** | | |
| Revised description of the window for conducting the optional activities (ophthalmologic evaluations and measured GFR) | Schedule of Activities (Table 2), footnotes t and u<br>Section 10.6<br>Section 10.7 | Description revised to align with study visit schedule. |
| **Safety reporting changes** | | |
| Moved definition of TEAEs and the period for recording and reporting TEAEs | Section 11.2.1.1<br>Section 11.2.2<br>Section 12.4 | Moved so provided with other definitions related to safety reporting. |
| **Statistical analysis changes** | | |
| Updated strategies for addressing ICEs | Section 12.3 and subsections | Updated to align with FDA comments. |
| Revised description of the data review for the analysis set | Section 12.2.7 | Revised for clarity. |

| Description of change | Section(s) affected | Rationale for change |
|---|---|---|
| Clarified the analysis set for the evaluation of endpoints related to renal biopsy observations | Section 12.3.2 | Revised for clarity. |
| **Study conduct changes** | | |
| Added description of the activities at the closure of enrollment | Section 6.2 | As the study was approaching target enrollment, the sponsor announced the planned closure of enrollment.<br>Sites continued patient screening activities and all eligible participants were enrolled, resulting in a final enrollment of 124 participants. Added a description to clarify activities at the closure of enrollment. |
| Revised description of antidrug antibody assessments | Section 11.1.13 | Revised to align with current Apellis practice. |
| **Document organization changes** | | |
| Added an Appendix containing country-specific modifications to the protocol. Country-specific modifications are noted for Canada, Czech Republic, France, Germany, Netherlands, Israel, Japan, and Switzerland. | Appendix 2 | Added to align with current Apellis practice. |

# 1. SYNOPSIS

| |
|---|
| **Name of Sponsor/Company:**<br>Apellis Pharmaceuticals, Inc |
| **Name of Active Substance:**<br>Pegcetacoplan (APL-2) |
| **Protocol Number:** APL2-C3G-310 |
| **Title of Study:**<br>A Phase 3, Randomized, Placebo-Controlled, Double-Blinded, Multicenter Study to Evaluate the Efficacy and Safety of Pegcetacoplan in Patients with C3 Glomerulopathy or Immune-Complex Membranoproliferative Glomerulonephritis |
| **Objectives:**<br>**Primary:**<br>To assess the efficacy of twice-weekly subcutaneous (SC) doses of pegcetacoplan compared with that of placebo in patients with primary C3 glomerulopathy (C3G) or immune-complex membranoproliferative glomerulonephritis (IC-MPGN) on the basis of a reduction in proteinuria.<br>**Secondary:**<br>• To assess the effect of pegcetacoplan on estimated glomerular filtration rate (eGFR)<br>• To assess the effect of pegcetacoplan on additional C3G/IC-MPGN disease–related parameters<br>• To evaluate the safety of pegcetacoplan over 52 weeks of treatment |
| **Endpoints:**<br>**Primary:**<br>The log-transformed ratio of uPCR at week 26 compared to baseline<br>**Key Secondary Endpoints:**<br>**Key secondary endpoints (to be evaluated at week 26)**<br>• The proportion of participants who meet the criteria for achieving a composite renal endpoint (a stable or improved eGFR compared to the baseline visit (≤15% reduction in eGFR), and a ≥50% reduction in uPCR compared to the baseline visit.)<br>• The proportion of participants with a reduction of at least 50% from baseline in uPCR<br>• For participants with evaluable renal biopsies, the change from baseline in the activity score of the C3G histologic index score<br>• The proportion of participants with evaluable renal biopsies showing decreases in C3c staining on renal biopsy from baseline<br>• Change from baseline in eGFR<br>**Additional Secondary Endpoints (to be evaluated at week 26):**<br>• The proportion of participants achieving proteinuria <1 g/day<br>• For participants with serum albumin levels below the lower limit of normal (LLN) at baseline, the proportion of participants with normalization of serum albumin levels<br>• For participants with serum C3 levels below the LLN at baseline, the proportion of participants with serum C3 levels above the LLN<br>• The change from baseline in Functional Assessment of Chronic Illness Therapy (FACIT)–Fatigue Scale score<br>• The change from baseline in Kidney Disease Quality of Life score<br>The primary, key secondary, and additional secondary endpoints will also be evaluated at week 52 as exploratory endpoints. |

**Safety Endpoints:**

- Incidence and severity of treatment-emergent adverse events (TEAEs)
- Change from baseline in vital signs measurements, clinical laboratory tests, and electrocardiogram results
- Number and incidence of rejection episodes (posttransplant participants only)
- Number and incidence of graft loss (posttransplant participants only)
- Incidence of death, stratified by transplant history

**Study Design:**

**Methodology:**

This is a phase 3, randomized, placebo-controlled, double-blinded, multicenter study to evaluate the safety and efficacy of twice-weekly SC infusions of pegcetacoplan in patients diagnosed with primary C3G or IC-MPGN.

The planned duration of participation in the study for each participant is a maximum of approximately 70 weeks. The study will consist of 4 parts:

- Part 1: 10-week screening period
- Part 2: 26-week randomized controlled period (RCP)
- Part 3: 26-week open-label period
- Part 4: 8-week follow-up period (only for participants who do not roll into a long-term extension study)

Informed consent (and assent if applicable) will be obtained prior to the conduct of any study-related procedures.

Part 1: Screening Period (10 Weeks)

- Participants will be screened to confirm that eligibility criteria are met.
- One 24-hour urine sample and 3 sets of triplicate first-morning spot urine (FMU) samples (first-morning void collected on 3 consecutive days) will be collected to determine eligibility and establish a baseline uPCR.
- For adult participants, a renal biopsy will be performed to confirm diagnosis and eligibility and to serve as the baseline renal biopsy. A historic renal biopsy done within 28 weeks of randomization may be used for eligibility confirmation and serve as the baseline biopsy if it is confirmed as adequate by central pathology review. Biopsies will not be required for adolescent participants if they have an adequate previous renal biopsy to establish the diagnosis, even if the previous biopsy is more than 28 weeks prior to randomization.
- Retrospective data for a period of up to 3 years prior to study entry will be collected to calculate historical eGFR (using the Chronic Kidney Disease–Epidemiology Collaboration creatinine equation for adults and the Bedside Schwartz equation for adolescents).
- Vaccinations against *Streptococcus pneumoniae*, *Neisseria meningitidis* (types A, C, W, Y, and B), and *Haemophilus influenzae* (type B) are mandatory unless documented evidence exists that participants have received the recommended vaccinations or are nonresponders to vaccination. If required, vaccination series should be initiated at least 14 days prior to randomization.

Part 2: Randomized Controlled Period (26 Weeks)

Approximately 80 to 100 participants who meet all of the inclusion criteria and none of the exclusion criteria will be randomly assigned in a 1:1 ratio to either the pegcetacoplan treatment arm or the placebo treatment arm. To achieve balance between the arms, 2 stratification factors will be applied to the randomization. The first stratification factor examines participants with posttransplant recurrence versus nontransplant participants; the second stratification factor examines participants with baseline renal biopsies (either collected during screening or a historic biopsy collected within 28 weeks prior to randomization) versus participants without baseline renal biopsies.

- **Pegcetacoplan group:** Participants will receive SC pegcetacoplan twice weekly.
- **Placebo group**: Participants will receive SC placebo twice weekly.
- Every effort will be made to minimize changes to medications related to C3G/IC-MPGN disease.

- Triplicate first-morning spot urines will be collected at each visit, and an additional triplicate first-morning sample will be collected during week 25.
- Safety and efficacy will be assessed.
- At the end of the RCP, there will be a renal biopsy, which is required for adult participants to advance to the open-label period (Part 3). Participants younger than 18 years are not required to provide renal biopsies and may advance to the open-label period upon completion of all assessments for weeks 24 through 26 other than the renal biopsy.

Part 3: Open-Label Period (26 Weeks)

- All participants will be treated with pegcetacoplan twice weekly to assess durability of response and long-term safety and efficacy. Open-label dosing will begin at the first visit after the week 26 renal biopsy for adults and any adolescents providing renal biopsies. Open-label dosing for adolescents not providing renal biopsies should start at the week 26 visit.
- At the end of the open-label period, there will be an optional renal biopsy.

Part 4: Follow-up Period (8 Weeks)

- Participants who would benefit from continuing to receive pegcetacoplan, as per the investigator's opinion, may roll over into a long-term extension study after completion of the open-label period.
- Participants who do not enter a long-term extension study will discontinue pegcetacoplan treatment and complete the 8-week follow-up period.

**Number of Participants (Planned) and Sample Size Justification:**

Approximately 80 to 100 participants, including patients with disease in native kidney or post-transplant, will be randomized 1:1 to pegcetacoplan or placebo with 40 to 50 participants per arm.

Based on preliminary data from Study APL2-201, a reduction of 60% in uPCR in the pegcetacoplan group at week 26 is assumed vs a reduction of 20% in uPCR in the placebo arm, which corresponds to mean log ratio to baseline of −0.92 vs −0.22 respectively, and a standard deviation of 0.88 (on log-scale). Based on this assumption, a sample size of 70 participants in total provides at least 90% power at 1-sided significance level of 0.025. Considering a 10% attrition to account for potential missing assessments and impact by COVID-19, it is expected that at least 78 participants with native kidney disease should be enrolled.

A minimum of 63 participants with C3G in native kidneys will be enrolled, which is approximately 80% of the enrolled participants with native kidney disease.

**Diagnosis:**

Patients with a diagnosis of primary C3G or IC-MPGN, including those with posttransplant recurrence

**Main Criteria for Inclusion:**

Inclusion Criteria

1. Aged at least 18 years; where approved, adolescents (aged 12-17 years) weighing at least 30 kg may also be enrolled.
2. A diagnosis of primary C3G or IC-MPGN (with or without previous renal transplant).
3. Evidence of active renal disease, based on one or more of the following:
   a. In adults or adolescents with a baseline renal biopsy (either one collected during screening or a historic biopsy collected within 28 weeks prior to randomization), at least 2+ C3c staining on the baseline renal biopsy.
   b. In adolescents not providing a baseline renal biopsy, at least one of the following:
      – Plasma sC5b-9 level above the upper limit of normal during screening
      – Serum C3 below the LLN during screening
      – Presence of an active urine sediment during screening, as evidenced by hematuria with at least 5 red blood cells per high-power field and/or red blood cell casts on routine local or central microscopic analysis of urine
      – Presence of C3 nephritic factor within 6 months of screening, based on central laboratory results or medical history

4. No more than 50% global glomerulosclerosis or interstitial fibrosis on the baseline biopsy for adult participants or adolescent participants providing a baseline biopsy.
5. At least 1 g/day of proteinuria on a screening 24-hour urine collection and a uPCR of at least 1000 mg/g in at least 2 first-morning spot urine samples collected during screening.
6. eGFR ≥30 mL/min/1.73 $m^2$ calculated by the Chronic Kidney Disease–Epidemiology Collaboration creatinine equation for adults or the Bedside Schwartz equation for adolescents.
7. Stable regimen for C3G/IC-MPGN treatment, as described below:
    a. Angiotensin-converting enzyme inhibitor, angiotensin receptor blocker, and/or sodium-glucose cotransporter-2 inhibitor therapy that is stable and optimized, in the opinion of the investigator, for at least 12 weeks prior to randomization
    b. Stable doses of other medications that can affect proteinuria (eg, steroids, mycophenolate mofetil, and/or other allowed immunosuppressants that the participant is receiving for treatment of C3G or IC-MPGN) for at least 12 weeks prior to randomization.
    c. If a participant is on prednisone (or other systemic corticosteroid) for C3G or IC-MPGN treatment, the dosage is stable and no higher than 20 mg/day (or equivalent dosage of a corticosteroid other than prednisone) for at least 12 weeks prior to randomization.
8. Have received vaccinations against *S pneumoniae*, *N meningitidis* (types A, C, W, Y, and B), and *H influenzae* (type B) as per ACIP recommendations for adults or children with complement deficiencies. Vaccination series should be initiated at least 14 days prior to randomization. Vaccination is mandatory unless documented evidence exists that participants are nonresponders to vaccination.
9. Female participants of childbearing potential, defined as any women who have experienced menarche and who are not permanently sterile or postmenopausal, must have negative blood pregnancy tests at screening (and negative urine pregnancy tests on day 1) and must agree to use protocol-defined methods of contraception from screening through at least 90 days after receiving the last dose of pegcetacoplan.
10. Male participants must agree to use protocol-defined methods of contraception and agree to refrain from donating semen from screening through at least 90 days after receiving the last dose of pegcetacoplan.
11. Participants above the legal age of consent, in accordance with local regulations, must be willing and able to provide informed consent. The legally authorized representative of participants under the legal age of consent must be willing and able to provide informed consent; where appropriate, participants under the legal age of consent must also give their assent to participation in the study.
12. Willing and able to self-administer pegcetacoplan or have an identified caregiver who can perform the administration.

<u>Exclusion Criteria</u>

1. Previous exposure to pegcetacoplan.
2. Evidence of improving renal disease in the 8 weeks prior to screening or during the screening period according to available data; improving renal disease is defined as >30% increase in eGFR or >50% decrease in proteinuria.
3. From a renal transplant participant, evidence of rejection that requires treatment in the baseline renal biopsy collected during screening.
4. C3G/IC-MPGN secondary to another condition (eg, infection, malignancy, monoclonal gammopathy, a systemic autoimmune disease such as systemic lupus erythematosus, chronic antibody-mediated rejection, or a medication), in the opinion of the investigator.
5. Current or prior diagnosis of HIV, hepatitis B, or hepatitis C infection or positive serology during screening that is indicative of infection with any of these viruses.
6. Body weight greater than 100 kg at screening.
7. Hypersensitivity to pegcetacoplan or to any of the excipients.
8. History of meningococcal disease.
9. Malignancy, except for the following:
    a. Cured basal or squamous cell skin cancer
    b. Curatively treated in situ disease

c. Malignancy-free and off treatment for ≥5 years

10. Severe infection (eg, requiring IV antibiotic therapy) within 14 days prior to the first dose of pegcetacoplan.
11. An absolute neutrophil count <1000 cells/mm$^3$ at screening.
12. Significant other renal disease that would, in the opinion of the investigator, confound interpretation of study results.
13. Participation in any other investigational drug trial or exposure to other investigational agent, device, or procedure within 30 days or 5 half-lives from the last dose of investigational agent (whichever is longer) prior to screening period.
14. Use of rituximab, belimumab, or any approved or investigational anticomplement therapy other than pegcetacoplan within 5 half-lives of that product prior to the screening period.
15. Female participants who are pregnant or who are currently breastfeeding and are unwilling to discontinue for the duration of the study and for at least 90 days after the final dose of study drug.
16. Inability to cooperate or any condition that, in the opinion of the investigator, creates an undue risk for the participant by participating in the study or is likely to confound interpretation of the study results.
17. Evidence of ongoing drug or alcohol abuse or dependence, in the opinion of the investigator.
18. Presence or suspicion of severe infection during the screening period (including but not limited to recurrent or chronic infections) that, in the opinion of the investigator, may place the participant at unacceptable risk by study participation.
19. Known or suspected hereditary fructose intolerance.

**Investigational Product, Dosage and Mode of Administration:**

Pegcetacoplan will be provided as a sterile solution in single-use glass vials. Each vial contains an isotonic sterile solution of pegcetacoplan 1080 mg/20 mL (54 mg/mL) in 10 mM acetate buffer, pH 5.0, containing 4.1% sorbitol. Placebo will be provided as a sterile solution of 10 mM acetate buffer, pH 5.0, containing 4.1% sorbitol supplied in single-use glass vials.

All participants will receive SC infusions of pegcetacoplan or matching volumes of placebo twice weekly. All adult participants (regardless of weight), and adolescent participants who weigh at least 50 kg, will receive 20-mL SC infusions. Adolescent participants who weigh at least 35 kg but less than 50 kg will receive a reduced infusion volume (12 mL for the first infusion and 15 mL for each infusion thereafter). Adolescent participants who weigh at least 30 kg but less than 35 kg will receive a further reduced infusion volume (10 mL for the first 2 infusions and 12 mL twice weekly thereafter), as presented below. Study drug will be self-administered by the participant or administered by their caregiver, after receiving appropriate training and sign-off by a research nurse or other qualified personnel in their first treatment week.

**Planned Dosing Regimens**

| Weight | First dose (infusion volume) | Second dose (infusion volume) | Maintenance dose (infusion volume) |
|---|---|---|---|
| All adult participants, adolescent participants ≥50 kg | 1080 mg (20 mL) | 1080 mg (20 mL) | 1080 mg twice weekly (20 mL) |
| Adolescent participants 35 to <50 kg | 648 mg (12 mL) | 810 mg (15 mL) | 810 mg twice weekly (15 mL) |
| Adolescent participants 30 to <35 kg | 540 mg (10 mL) | 540 mg (10 mL) | 648 mg twice weekly (12 mL) |

**Duration of Participation:**

Total participation will be 70 weeks (screening: 10 weeks; RCP: 26 weeks; open-label period: 26 weeks; follow-up: 8 weeks). Depending on treatment assignment, treatment with pegcetacoplan will be for a maximum of 52 weeks (RCP: 26 weeks; open-label period: 26 weeks).

**Statistical Methodology:**

**Efficacy:**

Primary Efficacy:

The targeted treatment regimen to be used for this study is pegcetacoplan or placebo plus stable regimen for C3G/IC-MPGN treatment. The primary estimand on the treatment effect of pegcetacoplan is for the participant population as defined through the study inclusion/exclusion criteria, with the primary endpoint based on the log-transformed ratio of uPCR (sampled from first-morning urine collections) at week 26 compared to baseline using an equal-weighted average over weeks 24, 25, and 26. Intercurrent events (ICEs) will be addressed as defined in Table 8.

The primary endpoint will be analyzed using a mixed effect model for repeated measures (MMRM). The model will include fixed categorical effects for treatment group, visit, stratification factors, disease type (C3G vs IC-MPGN), and the visit-by-treatment group interaction, as well as the continuous, fixed covariate of baseline log-transformed uPCR. Initially, an unstructured covariance matrix will be investigated. If this analysis fails to converge, other covariance structures will be used; details will be provided in the statistical analysis plan. The difference between treatment groups will be estimated with its 95% CI and corresponding *P* value.

**Key Secondary Efficacy:**

To preserve the overall type I error among the primary endpoint and key and additional secondary endpoints, a fixed-sequence testing strategy will be used; hence, statistical significance of the first secondary endpoint will be evaluated only if statistical significance is achieved with the primary analysis of the primary endpoint. The remaining secondary endpoints will also adhere to this testing strategy, and their order will match the order in which they are presented in Section 5.2.1.

- The numbers and proportion of participants who meet the criteria for achieving a composite renal endpoint at week 26 will be tabulated by treatment group and analyzed using a logistic regression model with treatment group as the independent variable and adjusted for baseline eGFR values, baseline log-transformed uPCR values, disease type, and stratification factors. The *P* value and odds ratio of being a responder for the pegcetacoplan group to being a responder for the placebo group and associated 95% CI will be provided.
- The numbers and proportion of participants who achieve uPCR response at week 26 (defined as a reduction from baseline in uPCR of at least 50% sampled from triplicate first-morning urine collections) will be tabulated by treatment group and compared using a logistic regression model similar to that for the composite renal endpoint. The odds ratio of being a responder for the pegcetacoplan group to being a responder for the placebo group with associated 95% CI and *P* value will be provided.
- For participants with evaluable renal biopsies, evaluation of the change from baseline to week 26 in the activity score in the C3G histologic index will use analysis of covariance (ANCOVA) model with treatment as a fixed effect, adjusted for baseline C3G histologic index activity score, disease type, and stratification factors. *P* value and least-square (LS) means will be presented for each treatment group, along with the between-treatment difference and 95% confidence interval.
- For participants with evaluable renal biopsies, the numbers and proportion of participants showing decreases in C3c staining on renal biopsy from baseline to week 26 will be tabulated by treatment group and compared using a logistic regression model similar to that for the composite renal endpoint. The odds ratio of being a responder for the pegcetacoplan group to being a responder for the placebo group and associated 95% CI and *P* value will be provided.
- Change from baseline in eGFR at week 26 will be analyzed using a MMRM model similar to that for the primary endpoint. The difference between treatment groups will be estimated with its 95% CI and corresponding *P* value.

**Safety:**

Adverse events (AEs) will be coded using the current version of the Medical Dictionary for Drug Regulatory Activities. TEAEs will be summarized by System Organ Class, Preferred Term, and treatment group for the number of participants and proportion reporting the event. A similar summary will be produced for serious adverse events, AEs leading to termination of study drug, severe AEs, and AEs related to the investigational product. The intensity of AEs and the relationship to investigational product will be summarized for each System Organ Class and Preferred Term by treatment group.

AEs leading to withdrawal will be summarized for each body system and Preferred Term by treatment group.

Laboratory assessments, antidrug antibody results, chest radiography, and electrocardiogram results will be summarized by treatment group using appropriate descriptive statistics.

**Interim Analyses:**

No interim analyses are planned.

# 2. TABLE OF CONTENTS

TITLE PAGE ....1
SIGNATURE PAGE ....2
INVESTIGATOR'S AGREEMENT ....3
PROTOCOL AMENDMENT SUMMARY OF CHANGES ....4
1. SYNOPSIS ....6
2. TABLE OF CONTENTS ....13
LIST OF TABLES ....18
LIST OF FIGURES ....18
3. LIST OF ABBREVIATIONS AND DEFINITIONS OF TERMS ....19
4. INTRODUCTION ....21
4.1. C3G and IC-MPGN and Unmet Medical Need ....21
4.2. Study Rationale ....21
4.2.1. Justification of Study Population ....22
4.2.2. Justification of Study Design ....22
4.2.3. Justification of Dose ....23
4.3. Summary of Clinical Experience With Pegcetacoplan ....23
4.4. Clinical Risks/Benefits of Pegcetacoplan ....24
4.4.1. COVID-19 Risk Mitigation Measures ....25
5. STUDY OBJECTIVES AND ENDPOINTS ....26
5.1. Study Objectives ....26
5.1.1. Primary Objective ....26
5.1.2. Secondary Objectives ....26
5.1.3. Exploratory Objectives ....26
5.2. Study Endpoints ....26
5.2.1. Efficacy Endpoints ....26
5.2.2. Safety Endpoints ....28
5.2.3. Pharmacokinetic, Pharmacodynamic, and Immunogenicity Endpoints ....28
6. INVESTIGATIONAL PLAN ....30
6.1. Overall Study Design ....30
6.1.1. Part 1: Screening Period (10 Weeks) ....33
6.1.1.1. Rescreening ....34

6.1.2. Part 2: Randomized Controlled Period (26 Weeks) ....................................34
6.1.3. Part 3: Open-Label Period (26 Weeks) .....................................................34
6.1.4. Part 4: Follow-up Period (8 Weeks) .......................................................35
6.2. Number of Participants ..............................................................................40
6.3. Treatment Assignment ................................................................................40
6.4. End of Study ...............................................................................................40
7. SELECTION AND WITHDRAWAL OF PARTICIPANTS .......................................41
7.1. Inclusion Criteria ........................................................................................41
7.1.1. Acceptable Methods of Contraception.....................................................42
7.2. Exclusion Criteria .......................................................................................43
7.3. Discontinuations and Participants Lost to Follow-up...............................................44
7.3.1. Early Treatment Discontinuation and Study Withdrawal..........................44
7.3.2. Reasons for Discontinuation of Treatment or Withdrawal From the Study ...............................................................................44
7.3.3. Lost to Follow-up Prior to Last Scheduled Visit .....................................45
7.3.4. Replacement of Participants ....................................................................45
8. TREATMENT OF PARTICIPANTS.......................................................................46
8.1. Study Interventions.....................................................................................46
8.2. Vaccinations ...............................................................................................46
8.2.1. Empiric Antibiotic Treatment for Possible Infection................................47
8.3. Concomitant Medications............................................................................47
8.3.1. Treatment Regimens for C3G or IC-MPGN.............................................48
8.3.1.1. Posttransplant Immunosuppression ....................................................48
8.3.2. Rescue Therapy........................................................................................48
8.3.3. Prohibited Medications ............................................................................48
8.4. Treatment Compliance.................................................................................49
8.5. Measures to Minimize Bias: Study Treatment Assignment ......................................49
8.5.1. Randomization .........................................................................................49
8.5.2. Blinding....................................................................................................49
8.5.2.1. Study Drug Unblinding........................................................................49
9. STUDY DRUG MATERIALS AND MANAGEMENT .............................................51
9.1. Study Drug...................................................................................................51
9.2. Study Drug Packaging and Labeling ..........................................................51

9.2.1. Packaging ....51
9.2.2. Labeling ....51
9.3. Study Drug Storage....51
9.4. Infusion Supplies ....52
9.5. Study Drug Administration....52
9.6. Study Drug Accountability ....53
9.7. Study Drug Handling and Disposal ....53
10. ASSESSMENT OF EFFICACY ....54
10.1. Retrospective Data Collection for eGFR and Measured Glomerular Filtration Rate....54
10.2. 24-Hour Urine Collection ....54
10.3. Triplicate FMU Collections....55
10.4. Random Spot Urine Collection....55
10.5. Renal Biopsy....56
10.5.1. Renal Biopsies in Adults....56
10.5.2. Renal Biopsies in Adolescents....56
10.6. Ophthalmologic Evaluation ....57
10.7. Measured GFR....57
10.8. Health-Related Quality of Life Assessments....58
10.8.1. Functional Assessment of Illness Therapy–Fatigue Scale....58
10.8.2. 5-Level EuroQol-5 Dimension ....58
10.8.3. Kidney Disease Quality of Life ....58
10.8.4. Patient Global Impression of Change ....58
10.8.5. Work Productivity and Activity Impairment Questionnaire: Specific Health Problem ....58
11. ASSESSMENT OF SAFETY ....59
11.1. Safety Parameters ....59
11.1.1. Demographic/Medical History....59
11.1.2. Vital Signs....59
11.1.3. Weight and Height ....60
11.1.4. Physical Examination....60
11.1.5. Menses ....60
11.1.6. Electrocardiograms ....60

11.1.7. Chest Radiography .....60
11.1.8. Infusion Site/Pump Safety Assessment .....61
11.1.9. Laboratory Assessments .....61
11.1.9.1. Pregnancy Screen .....62
11.1.10. Pharmacokinetic Assessment .....63
11.1.11. Pharmacodynamic Assessment .....63
11.1.12. Blood Volume for Study Assessments .....63
11.1.13. Antidrug Antibody Assessment .....64
11.1.14. COVID-19 Assessments .....65
11.2. Adverse and Serious Adverse Events .....65
11.2.1. Definitions .....65
11.2.1.1. Adverse Events .....65
11.2.1.2. Serious Adverse Events .....65
11.2.1.3. Unexpected AEs .....66
11.2.2. Recording and Reporting AEs .....66
11.2.2.1. Relationship to Study Drug .....66
11.2.2.2. Severity of Events .....67
11.2.3. Reporting AEs .....67
11.2.4. Pregnancy .....67
11.2.5. AEs of Special Interest .....68
11.2.6. Abuse, Misuse, Overdose, and Medication Errors .....68
12. STATISTICS .....69
12.1. Determination of Sample Size .....69
12.2. Analysis Set .....69
12.2.1. Screened Set .....69
12.2.2. Safety Set .....69
12.2.3. Intent-to-Treat Set .....69
12.2.4. Per-Protocol Set .....69
12.2.5. Pharmacokinetic Set .....70
12.2.6. Pharmacodynamic Set .....70
12.2.7. Data Review for Analysis Set .....70
12.3. Efficacy Analysis .....70
12.3.1. Analysis of Primary Efficacy Endpoint .....70

12.3.2. Analysis of Secondary Efficacy Endpoints ..... 71
12.3.2.1. Key Secondary Efficacy Endpoints: ..... 71
12.3.2.2. Additional Secondary Efficacy Endpoints: ..... 73
12.3.3. Analysis of Exploratory Endpoints ..... 74
12.4. Safety Analysis ..... 74
12.5. Pharmacokinetic Analysis ..... 75
12.6. Pharmacodynamic Analysis ..... 76
12.7. Other Analyses ..... 76
12.8. Interim Analysis ..... 76
12.9. Data Monitoring Committee ..... 76
13. DIRECT ACCESS TO SOURCE DATA/DOCUMENTS ..... 77
13.1. Study Monitoring ..... 77
13.2. Audits and Inspections ..... 77
13.3. Institutional Review Board ..... 78
14. QUALITY CONTROL AND QUALITY ASSURANCE ..... 79
15. ETHICS ..... 80
15.1. Ethics Review ..... 80
15.2. Ethical Conduct of the Study ..... 80
15.3. Written Informed Consent ..... 80
16. DATA HANDLING AND RECORDKEEPING ..... 82
16.1. Inspection of Records ..... 82
16.2. Retention of Records ..... 82
17. PUBLICATION POLICY ..... 83
18. LIST OF REFERENCES ..... 84
19. APPENDICES ..... 86
Appendix 1. Protocol Amendment History ..... 87
Appendix 2. Country-Specific Protocol Modifications ..... 97
Appendix 3. COVID-19 Study Continuity Plan ..... 108

# LIST OF TABLES

Table 1: Efficacy Endpoints....27
Table 2: Schedule of Activities....36
Table 3: Planned Dosing Regimens (Pegcetacoplan and Placebo)....46
Table 4: Laboratory Assessments....61
Table 5: Approximate Blood Volume Collected....64
Table 6: Definitions of Adverse Event Relatedness....66
Table 7: Severity of Events....67
Table 8: Intercurrent Events During the Randomized Treatment Phase and Strategies Addressing Intercurrent Events for the Primary Efficacy Endpoint....71
Table 9: Intercurrent Events During the Randomized Treatment Phase and Strategies Addressing Intercurrent Events for the Composite Renal Endpoint....72

# LIST OF FIGURES

Figure 1: Study APL2-C3G-310 Design....32

# 3. LIST OF ABBREVIATIONS AND DEFINITIONS OF TERMS

The following abbreviations and specialist terms are used in this study protocol.

| Abbreviation | Definition |
|---|---|
| ACIP | Advisory Committee on Immunization Practices |
| ADA | antidrug antibodies |
| AE | adverse event |
| AH50 | 50% alternative hemolytic complement pathway activity |
| AP | alternative pathway |
| C3G | C3 glomerulopathy |
| C3GN | C3 glomerulonephritis |
| CH50 | 50% classical hemolytic complement pathway activity |
| CKD-EPI | Chronic Kidney Disease–Epidemiology Collaboration |
| CRF | case report form |
| DDD | dense deposit disease |
| DMC | data monitoring committee |
| ECG | electrocardiogram |
| eGFR | estimated glomerular filtration rate |
| ESRD | end-stage renal disease |
| FACIT-Fatigue | Functional Assessment of Chronic Illness Therapy–Fatigue |
| FDA | Food and Drug Administration |
| FMU | first-morning spot urine |
| GCP | Good Clinical Practice |
| GFR | glomerular filtration rate |
| HHCP | home health care provider |
| HRQoL | health-related quality of life |
| IB | investigator's brochure |
| IC-MPGN | immune-complex membranoproliferative glomerulonephritis |
| ICE | intercurrent event |
| ICF | informed consent form |
| ICH | International Council for Harmonisation of Technical Requirements for Pharmaceuticals for Human Use |
| IEC | independent ethics committee |
| IND | Investigational New Drug |
| IRB | institutional review board |
| ITT | intent-to-treat |
| KDQOL | Kidney Disease Quality of Life |

| Abbreviation | Definition |
|---|---|
| LLN | lower limit of normal |
| MAR | missing at random |
| MMRM | mixed effect model for repeated measures |
| PD | pharmacodynamic |
| PEG | polyethylene glycol |
| PK | pharmacokinetic |
| PNH | paroxysmal nocturnal hemoglobinuria |
| PP | per-protocol |
| RCP | randomized controlled period |
| SAE | serious adverse event |
| SAP | statistical analysis plan |
| SC | subcutaneous |
| SD-OCT | spectral domain optical coherence tomography |
| SUSAR | suspected unexpected serious adverse reaction |
| TEAE | treatment-emergent adverse event |
| uACR | urine albumin-to-creatinine ratio |
| uBCR | urinary β2-microglobulin–to-creatinine ratio |
| uPCR | urine protein-to-creatinine ratio |
| WOCBP | women of childbearing potential |
| WPAI | Work Productivity and Activity Impairment |

# 4. INTRODUCTION

Two similar diseases, C3 glomerulopathy (C3G) and immune-complex membranoproliferative glomerulonephritis (IC-MPGN), will be studied in this protocol. This study will include patients with native kidney disease or with posttransplant disease recurrence and will exclude patients whose C3G or IC-MPGN is secondary to an underlying condition (eg, infection, monoclonal gammopathy, malignancy, or medication).

## 4.1. C3G and IC-MPGN and Unmet Medical Need

C3G is a disease in which the complement system, specifically the alternative pathway (AP), is indiscriminately overactive, leading to excessive deposition of C3 breakdown products in the glomeruli of the kidney, and damage to the renal parenchyma. The underlying AP hyperactivity can be due to various factors, most commonly, an acquired autoantibody which stabilizes the AP C3 or C5 convertase (C3 or C5 nephritic factor). AP hyperactivity leads to uncontrolled C3 activation, resulting in an excess of C3 breakdown products that are deposited in the glomerular basement membrane and mesangium. This stimulates a brisk inflammatory response, disrupting the normal glomerular architecture.

There are 2 subtypes of C3G: dense deposit disease (DDD) and C3 glomerulonephritis (C3GN), which are distinguishable only by renal biopsy. The clinical presentation, features, and disease course are similar between the 2 subtypes, but with some distinctions. DDD tends to present earlier in life than C3GN (median ages of diagnosis for DDD and C3GN are 12 and 26 years old, respectively), and DDD is also associated with extrarenal manifestations, such as acquired partial lipodystrophy and macular degeneration. The incidence of C3GN is about 3-fold greater than that of DDD (Medjeral-Thomas et al. 2014; Servais et al. 2012; Lu et al. 2012).

Although IC-MPGN is typically considered a distinct pathologic entity from C3G, the underlying pathophysiology and clinical course of the 2 primary diseases are remarkably similar (Noris et al. 2019; Holle et al. 2018). As in C3G, patients with primary IC-MPGN often have evidence of AP hyperactivity, including the presence of genetic or acquired causes of AP dysregulation. In these patients, uncontrolled C3 activation and glomerular deposition of C3 breakdown products is also observed, similar to C3G. The only pathologic distinction from C3G is that the glomerular C3 deposition in IC-MPGN is accompanied by immunoglobulin deposition. The common clinical manifestations of primary IC-MPGN are the same as those of C3G and other glomerulonephritides – proteinuria, hematuria, hypertension, and reduced glomerular filtration rate.

## 4.2. Study Rationale

There are no therapies approved to prevent or reverse disease progression in C3G or IC-MPGN. Similar to other glomerular diseases, disease management includes nonspecific measures to manage proteinuria, hypertension, hyperlipidemia, edema, and other facets of glomerular and chronic kidney disease. Despite these various measures, the prognosis of C3G and IC-MPGN is poor as patients steadily progress to end-stage renal disease (ESRD) (Smith et al. 2019; Regunathan-Shenk et al. 2019; Medjeral-Thomas et al. 2014; Zhang et al. 2014; Pickering et al. 2013; Lu et al. 2012; Okpechi et al. 2014).

Renal transplantation is an option for patients who reach ESRD, but the incidence of disease recurrence is high, with up to 50% of patients losing their renal allografts because of disease recurrence (Medjeral-Thomas et al. 2014; Zhang et al. 2014; Pickering et al. 2013; Lu et al. 2012). Therefore, a therapy that can protect the kidneys, native or transplanted, from ongoing damage due to complement hyperactivity is an unmet need.

As an inhibitor of C3 and its fragment, C3b, pegcetacoplan has the potential to address the underlying disease pathophysiology of complement hyperactivity in C3G and IC-MPGN. Specifically, by inhibiting C3, it is expected that pegcetacoplan will halt the inappropriate and excessive C3 activation that characterizes these diseases. Additionally, by inhibiting C3b, pegcetacoplan inhibits the activity of the AP C3 convertase (whether it is stabilized by nephritic factors or not) through a second complementary mechanism of action in addition to the inhibition of C3 as a substrate in the complement cascade. These 2 complementary mechanisms are expected to prevent further deposition of C3 breakdown products in the glomeruli, affording the opportunity for the kidney to recover by clearing existing deposits and resolving inflammation. This renal recovery would be expected, initially, to yield a reduction in proteinuria, and ultimately, to result in prolonged renal survival. Therefore, pegcetacoplan is a potentially life-altering therapy for C3G and IC-MPGN.

### 4.2.1. Justification of Study Population

Based on the mechanism of action of pegcetacoplan, Apellis aims to study patients with glomerular disease driven by complement over-activation and as such, will include patients with either C3G or IC-MPGN, as both are characterized by complement dysregulation leading to excessive deposition of C3 breakdown products in glomeruli (Iatropoulos et al. 2018; Cook 2018; Servais et al. 2012). Because the same underlying pathophysiology of complement dysregulation that leads to C3G/IC-MPGN in the native kidney also leads to disease recurrence posttransplant, and the clinical course for both is characterized by significant proteinuria and risk of progression to ESRD, the trial includes patients with posttransplant disease recurrence, in addition to nontransplant patients. Finally, the trial includes adolescents (ages 12–17), as the disease features in adolescents are similar to those of adults.

### 4.2.2. Justification of Study Design

A randomized, placebo-controlled, double-blinded trial design was selected in order to evaluate the treatment effect of pegcetacoplan on proteinuria and renal function in patients with C3G or IC-MPGN in a robust and minimally biased fashion. The placebo control enables identification of potential study effects that are not related to the study drug and provides data on the course of the disease in the absence of study treatment. Given that participants must have had stable or worsening disease for the preceding 3 months prior to study entry, a spontaneous disease remission is unlikely to occur during the study. However, participants may have improved (or worsened) compliance with nonstudy medications related to study participation. These types of study effects are expected to occur in the first 2 to 3 months of the study, so any placebo-related effect should be evident and stable by week 26.

To minimize unnecessary exposure for participants to placebo infusions, the study will include a 26-week placebo-controlled period, after which the placebo group will be transitioned to pegcetacoplan treatment. In this way, all participants will have the opportunity to receive pegcetacoplan in a reasonable time frame while still establishing the placebo response rate.

### 4.2.3. Justification of Dose

The dosing regimen for adults will be 1080 mg administered subcutaneously (SC) twice weekly. This dosing regimen is intended to result in systemic exposures of pegcetacoplan similar to those seen in the ongoing phase 2 study in patients with complement-mediated glomerulopathies, Study APL2-201, and the phase 3 studies in patients with paroxysmal nocturnal hemoglobinuria (PNH), Study APL2-302 and Study APL2-308, in which pegcetacoplan has been generally safe and well tolerated.

The dosing regimens for adolescents are intended to yield similar exposures as 1080 mg twice weekly in adults. Modeling-based simulations were performed for adolescents on the basis of an adult population PK model incorporating pegcetacoplan serum concentration-time data from 10 clinical studies (five phase 1 studies in healthy volunteers, one special population study in adults with severe renal impairment, three phase 1b/2a studies in adult PNH patients, and one phase 3 study [Study APL2-302; data up to week 16] in adult PNH patients). The results of these model analyses were used to select the dosing regimens for adolescents, as summarized below:

- In adolescents aged 12 to 17 years with body weights of at least 50 kg, a dose regimen of 1080 mg twice weekly is expected to result in exposure similar to that seen in adults at the same dosage.
- In adolescents aged 12 to 17 years with body weights of 35 to 49 kg, a 648-mg initial dose followed by 810 mg twice weekly is anticipated to result in exposures at weeks 1, 2, and 16 (steady-state) that are similar to those of the adult dosage of 1080 mg twice weekly.
- In adolescents aged 12 to 17 years with body weights of 30 to 34 kg, the first 2 doses will be 540 mg, and following doses will be 648 mg twice weekly. This regimen is anticipated to result in exposures at weeks 1, 2, and 16 (steady-state) that are similar to those of the adult dosage of 1080 mg twice weekly.

Modeling of exposure in high body weight participants (those weighing >100 kg) results in predicted exposures at least 15% lower than those for a reference 70-kg participant; participants with body weights of >100 kg will therefore be excluded from the study.

## 4.3. Summary of Clinical Experience With Pegcetacoplan

Pegcetacoplan is being studied in APL2-201, an ongoing phase 2 study that includes patients with 1 of 4 glomerular diseases: C3G, immunoglobulin (Ig) A nephropathy, primary membranous nephropathy, or lupus nephritis. Preliminary data from the 8 C3G participants in this study indicate that pegcetacoplan is able to target the complement hyperactivity of C3G, as well as reduce proteinuria in patients with C3G.

Clinical data is also available from 5 studies with pegcetacoplan in patients with PNH (phase 1b: Studies APL-CP0514 and APL2-CP-PNH-204; phase 2a: Study APL2-202; phase 3: Studies APL2-302 and APL2-308). Results from these studies indicate that pegcetacoplan is effective in providing broad control of intravascular and extravascular hemolysis, as evidenced by the following: increased and stable hemoglobin levels, normalization of lactate dehydrogenase levels, normalization of absolute reticulocyte count, normalization of total bilirubin levels, reduction in C3 fragment opsonization, and increased numbers of PNH red blood cells.

Pegcetacoplan at doses of 360 mg/d and 1080 mg twice weekly has been generally well tolerated when administered via SC infusion, including to patients with C3G and other glomerular diseases.

Refer to the latest version of the pegcetacoplan investigator's brochure (IB) for the overall risk/benefit assessment and the most current information regarding the drug metabolism, pharmacokinetic (PK), efficacy, and safety of pegcetacoplan.

## 4.4. Clinical Risks/Benefits of Pegcetacoplan

Pegcetacoplan has the potential to address the underlying disease pathophysiology of complement hyperactivity in C3G and IC-MPGN, and, therefore, to provide benefit in these diseases with a high unmet medical need.

The safety of SC pegcetacoplan administration has been studied in multiple phase 2 and 3 studies for C3G, PNH, and autoimmune hemolytic anemia, with an acceptable safety profile to date. Nonetheless, a number of safety monitoring practices are being employed by this protocol to ensure participant safety, including physical examination, vital signs monitoring, electrocardiograms (ECGs), hematology (including coagulation), serum chemistry, urinalysis at specified intervals, as well as prompt reporting of adverse events (AEs).

Infusion site/pump safety will be assessed during clinical visits, and any significant finding from the assessment will be recorded as an AE (see Section 11.2.2). The volume of blood planned for collection from each participant over the course of the study will be minimized to limit the impact on the overall health of these anemic participants.

Systemic complement inhibition might predispose individuals to infections caused by encapsulated organisms, including *Streptococcus pneumoniae*, *Neisseria meningitidis*, and *Haemophilus influenzae*. Vaccinations against these organisms according to the most current Advisory Committee on Immunization Practices (ACIP) recommendations for patients with complement deficiencies will be required to minimize potential risk of infection. Vaccinations will be initiated at least 14 days prior to receiving the first dose of pegcetacoplan; therefore, prophylactic antibiotic use is not required. Body temperature and vital signs will be monitored periodically, and relevant blood parameters monitored regularly throughout the study to assess for signs of infection. Participants will be counseled regarding this potential risk for infection and given a participant safety wallet card in the event of an emergency. The investigator should be contacted immediately in the event of a suspected infection for guidance on appropriate action to be taken.

### 4.4.1. COVID-19 Risk Mitigation Measures

Apellis is not currently aware of any evidence associating pegcetacoplan use with specific risks or complications of COVID-19. Apellis recognizes the need to consider the public health risks of the COVID-19 pandemic within the context of conducting a clinical trial. Because these risks may change as the pandemic evolves and may vary on the basis of geographic location, Apellis will continue to evaluate the risk/benefit around study conduct on an ongoing and participant-by-participant basis.

In the event that an investigative site is closed or a participant is unable or unwilling to travel because of the COVID-19 pandemic, and if in the opinion of the investigator it is in the participant's best interest to continue in the study, the following mitigation measures may be implemented for the study and used if deemed necessary and authorized by the sponsor, including but not limited to:

- Collection of AEs or serious adverse events (SAEs) should be done over the phone and documented in the source documents.
- A minimized schedule of activities is presented in Table A1 of Appendix 3.
- Any activities not performed because of COVID-19–related restrictions will be identified in the participant source document.
- Any change in COVID-19 status (serology or antigen), if available, will be captured in the participant's source document.
- Where applicable, relevant study documentation will be updated and communicated to health authorities and/or institutional review boards (IRBs) or independent ethics committees (IECs) as required.

Apellis is currently not aware of any contraindications to vaccination (of any modality) and concurrent treatment with pegcetacoplan. In the absence of data, Apellis currently recommends that the decision to vaccinate study participants against COVID-19 should be made by the investigator and the participant, with consideration of local and national vaccination recommendations and a benefit-risk assessment. Vaccination against COVID-19 is not mandatory for continued study participation.

These measures are described in more detail Appendix 3.

# 5. STUDY OBJECTIVES AND ENDPOINTS

## 5.1. Study Objectives

### 5.1.1. Primary Objective

The primary objective of this study is to assess the efficacy of twice-weekly SC doses of pegcetacoplan compared with that of placebo in patients with primary C3G or IC-MPGN on the basis of a reduction in proteinuria.

### 5.1.2. Secondary Objectives

The secondary objectives of the study are as follows:

- To assess the effect of pegcetacoplan on estimated glomerular filtration rate (eGFR)
- To assess the effect of pegcetacoplan on additional C3G/IC-MPGN disease–related parameters
- To evaluate the safety of pegcetacoplan over 52 weeks of treatment

### 5.1.3. Exploratory Objectives

The exploratory objectives are to characterize the additional clinical, laboratory, and histologic findings of C3G/IC-MPGN in response to pegcetacoplan.

## 5.2. Study Endpoints

### 5.2.1. Efficacy Endpoints

Table 1 lists the efficacy endpoints and the time points at which they will be evaluated. The primary and secondary endpoints will be evaluated at week 26. The primary and secondary endpoints will also be evaluated at week 52 as exploratory endpoints. The table also lists additional exploratory endpoints which will be evaluated at both week 26 and week 52.

### Table 1: Efficacy Endpoints

| Study endpoints | Week 26 | Week 52 |
|---|---|---|
| **Primary efficacy endpoint** | | |
| The log-transformed ratio of uPCR at week 26 compared to baseline | X | |
| **Key secondary efficacy endpoints** | | |
| The proportion of participants who meet the criteria for achieving a composite renal endpoint (a stable or improved eGFR compared to the baseline visit (≤15% reduction in eGFR), and a ≥50% reduction in uPCR compared to the baseline visit.) | X | |
| The proportion of participants with a reduction of at least 50% from baseline in uPCR | X | |
| For participants with evaluable renal biopsies, the change from baseline in the activity score of the C3G histologic index score (Bomback et al. 2018) | X | |
| The proportion of participants with evaluable renal biopsies showing decreases in C3c staining on renal biopsy from baseline | X | |
| Change from baseline in eGFR | X | |
| **Additional secondary efficacy endpoints** | | |
| The proportion of participants achieving proteinuria <1 g/day | X | |
| For participants with serum albumin levels below the LLN at baseline, the proportion of participants with normalization of serum albumin levels | X | |
| For participants with serum C3 levels below the LLN at baseline, the proportion of participants with serum C3 levels above the LLN | X | |
| The change from baseline in the FACIT-Fatigue Scale score | X | |
| The change from baseline in the KDQOL score | X | |
| **Exploratory efficacy endpoints** | | |
| The log-transformed ratio of uPCR at week 52 compared to baseline | | X |
| The proportion of participants who meet the criteria for achieving a composite renal endpoint | | X |
| The proportion of participants with a reduction from baseline in uPCR of at least 50% | | X |
| Change from baseline in eGFR | | X |
| For participants with evaluable renal biopsies, the change from baseline in the activity score of the C3G histologic index (Bomback et al. 2018) | | X |
| The proportion of participants with evaluable renal biopsies showing decreases in C3c staining on renal biopsy from baseline | | X |
| The proportion of participants achieving proteinuria <1 g/day | | X |
| For participants with serum albumin levels below the LLN at baseline, the proportion of participants with normalization of serum albumin levels | | X |
| For participants with serum C3 levels below the LLN at baseline, the proportion of participants with serum C3 levels above the LLN | | X |
| The change from baseline in the FACIT-Fatigue Scale score | | X |
| The change from baseline in the KDQOL score | | X |
| The change from baseline in uPCR using the 24-hour urine collections | X[1] | X[1] |
| The annual rate of change from up to 3 years prior to screening in eGFR | X | X |

**Table 1: Efficacy Endpoints**

| Study endpoints | Week 26 | Week 52 |
|---|---|---|
| The proportion of participants with reductions from baseline in proteinuria of at least 30% | X | X |
| The proportion of participants with normalization of proteinuria | X | X |
| The time to 50% reduction in uPCR with a stable or improved eGFR | n/a | n/a |
| The time to normalization for the following parameters for participants in whom the parameter is abnormal at baseline:<br>• serum C3<br>• uPCR<br>• serum albumin<br>• blood pressure | n/a | n/a |
| The change in glomerular macrophage count, as determined by CD68 staining | X | X |
| The change from baseline in PGIC score | X | X |
| The change from baseline in EQ-5D-5L score | X | X |
| The change from baseline in WPAI score | X | X |
| Change in drusen from baseline:<br>• change in maximum drusen size<br>• change in number of intermediate or large drusen | | X |

Abbreviations: C3G = C3 glomerulopathy; eGFR = estimated glomerular filtration rate; FACIT-Fatigue = Functional Assessment of Chronic Illness Therapy–Fatigue; KDQOL = Kidney Disease Quality of Life; LLN = lower limit of normal; PEG = polyethylene glycol; PGIC = Patient Global Impression of Change; uPCR = urine protein-to-creatinine ratio; WPAI = Work Productivity and Activity Impairment.

1. 24-hour urine collections are at week 24 and week 48.

### 5.2.2. Safety Endpoints

The safety endpoints are as follows:

- The incidence and severity of treatment-emergent adverse events (TEAEs)
- The change from baseline in:
  - vital signs measurements
  - clinical laboratory tests
  - ECG results
- The number and incidence of rejection episodes (posttransplant participants only)
- The number and incidence of graft loss (posttransplant participants only)
- The incidence of death, stratified by transplant history

### 5.2.3. Pharmacokinetic, Pharmacodynamic, and Immunogenicity Endpoints

The PK endpoint is:

- Pegcetacoplan serum concentrations over time

The pharmacodynamic (PD) endpoints are:

- Changes from baseline in complement levels at week 26 and week 52:
  - CH50 (50% classical hemolytic complement pathway activity)
  - AH50 (50% alternative hemolytic complement pathway activity)
  - sC5b-9

Additional complement components may be measured, as noted in Table 4 and Section 11.1.11, and evaluated as exploratory endpoints.

The immunogenicity endpoint is:

- The incidence of antidrug antibodies (ie, antibodies against the peptide and polyethylene glycol [PEG] domains of pegcetacoplan)

# 6. INVESTIGATIONAL PLAN

This phase 3 randomized, placebo-controlled, double-blinded, multicenter clinical study is designed to evaluate the safety and efficacy of twice-weekly SC infusions of pegcetacoplan in patients with primary C3G or IC-MPGN. There will be approximately 80 to 100 participants enrolled in this study, at least 78 of whom will have native kidney disease and up to 22 of whom may have posttransplant recurrence of C3G or IC-MPGN. At least 63 participants with C3G in native kidney will be enrolled. The enrollment of participants with C3G or IC-MPGN will be monitored to ensure balance between the groups. At least 10 adolescent participants (aged 12-17 years) will be enrolled; adolescent participants may be either patients with native kidney disease or posttransplant disease recurrence. Participants initially screened as adolescents will follow adolescent procedures and requirements through the duration of their participation in study, even if they pass their 18th birthday while enrolled in the study.

The planned length of participation in the study for each participant is a maximum of approximately 70 weeks. This study will consist of 4 parts:

- Part 1: 10-week screening period
- Part 2: 26-week randomized controlled period (RCP)
- Part 3: 26-week open-label period
- Part 4: 8-week follow-up period (only for participants who do not roll into a long-term extension study)

Informed consent (and assent, when applicable) will be obtained prior to any study-related procedures being conducted. The screening period will start once informed consent has been obtained.

See Figure 1 for the study schematic, and Table 2 for the schedule of activities.

## 6.1. Overall Study Design

This is a placebo-controlled, double-blinded, phase 3 study in patients with clinical and pathologic evidence of primary C3G or IC-MPGN. Participants will be randomly assigned 1:1 to either the pegcetacoplan group (pegcetacoplan treatment throughout the RCP and open-label period) or the placebo group (placebo treatment during the RCP and pegcetacoplan treatment during the open-label period). The visit schedule will be the same, regardless of randomization.

The study includes renal biopsies, one during screening and a second at the end of the RCP (week 26). An optional third biopsy may be collected at the end of the open-label period (week 52). The biopsy requirements are discussed in Section 10.5, including additional information on biopsies for participants under the age of 18.

After completion of the RCP and open-label period, any participant who, in the opinion of the investigator, is experiencing clinical benefit from pegcetacoplan administration will be invited to participate in a planned long-term extension study to continue to receive treatment with pegcetacoplan until it is commercially available for the treatment of C3G and/or IC-MPGN and accessible in their countries, or the development program for C3G and/or IC-MPGN is terminated, in compliance with all applicable national and local laws and regulations.

Participants who do not continue on pegcetacoplan in a planned long-term extension study will enter the 8-week follow-up period of this study.

See Figure 1 for the study schematic.

**Figure 1: Study APL2-C3G-310 Design**

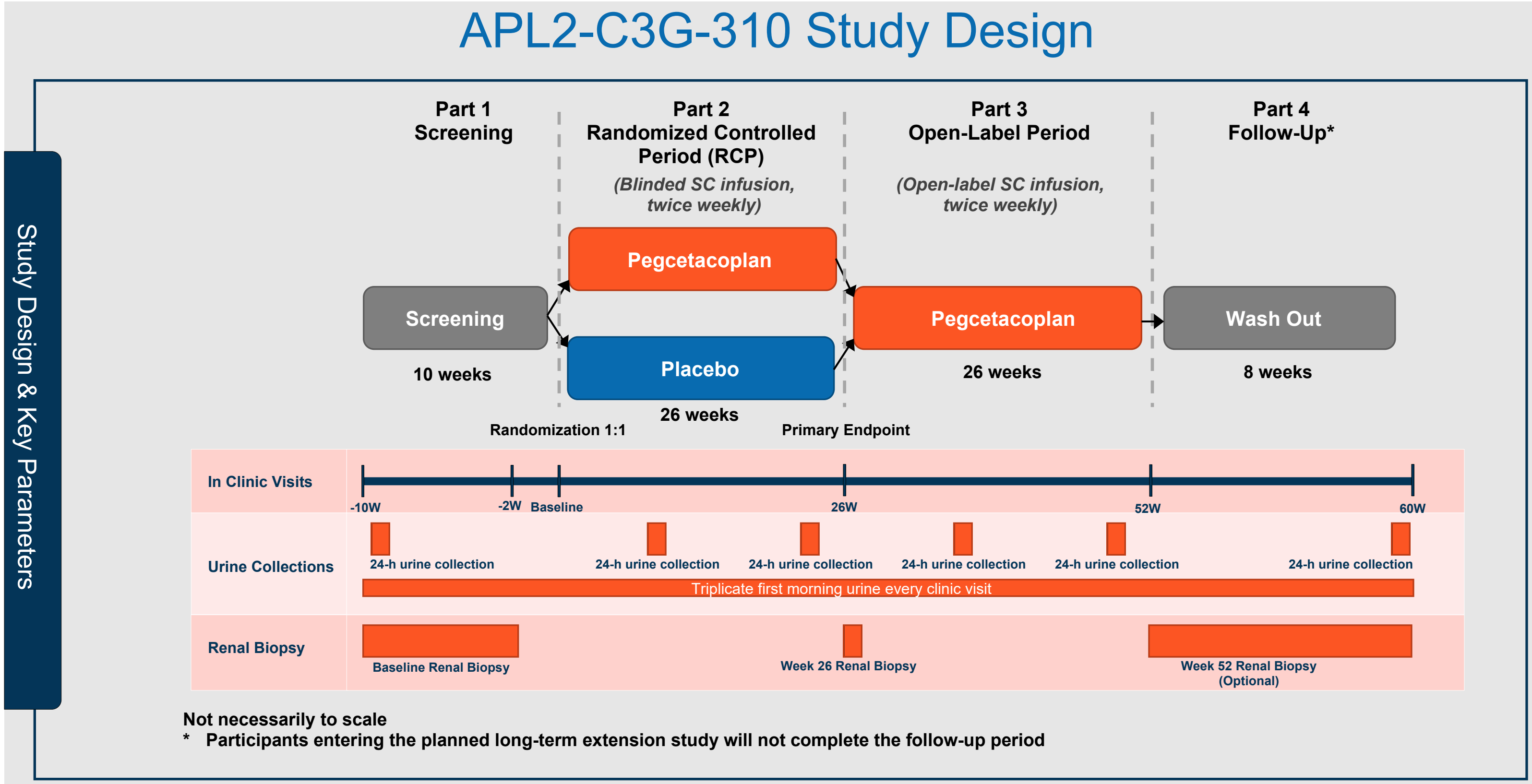


Abbreviations: SC = subcutaneous; uPCR = urine protein-to-creatinine ratio; W = week.

### 6.1.1. Part 1: Screening Period (10 Weeks)

Participants will be screened to confirm that the selection criteria for the study have been met. The screening period will occur in 2 stages, and all assessments described in the schedule of activities (Table 2) at screening visit 1 should occur before those in screening visit 2. This ensures that the more invasive procedures (vaccinations and renal biopsy) do not occur unless the participants meet all other eligibility criteria.

- One 24-hour urine sample and 3 sets of triplicate first-morning spot urine (FMU) samples (first-morning void collected on 3 consecutive days) will be collected to determine eligibility and establish a baseline uPCR. Collection of urine samples is discussed in more detail in Section 10.2 and Section 10.3. Calculation of baseline uPCR is discussed in Section 12.3.1 and Section 12.3.2.
- For adult participants, a renal biopsy will be performed to confirm diagnosis and eligibility and to serve as the baseline renal biopsy. The baseline renal biopsy may be conducted at any time during screening once eligibility is confirmed by the assessments from screening visit 1. All biopsies will be evaluated by the central pathology laboratory. Renal biopsies are discussed in more detail in Section 10.5.
    - A historic renal biopsy done within 28 weeks of randomization may be used for eligibility confirmation and serve as the baseline biopsy if it is confirmed as adequate by the central pathology laboratory on the basis of review of the biopsy report as well as slides and/or images.
    - Participants under the age of 18 are not required to provide a biopsy if they have an adequate previous renal biopsy to establish the diagnosis, even if the previous biopsy is more than 28 weeks prior to randomization.
- Retrospective data for a period of up to 3 years prior to study entry will be collected to calculate historical eGFR using the Chronic Kidney Disease–Epidemiology Collaboration [CKD-EPI] creatinine equation for adults and the Bedside Schwartz equation ($eGFR = 0.413 \times [\text{height (in centimeters)}] / \text{serum creatinine}$) for adolescents. Retrospective data for eGFR are discussed in more detail in Section 10.1.
- The participant's medical history and screening data will be reviewed by the investigator, in collaboration with the medical monitor, to confirm the diagnosis of primary C3G or IC-MPGN. The investigator and medical monitor will consider all available historical, laboratory, and pathology data. For an adolescent in whom a renal biopsy within 28 weeks of day 1 is not available, the investigator and medical monitor will consider alternative evidence for active renal disease as described in Section 7.1.
- Vaccinations against *S pneumoniae*, *N meningitidis* (types A, C, W, Y, and B), and *H influenzae* (type B) are mandatory unless documented evidence exists that participants are nonresponders to vaccination. If required, vaccination series should be initiated at least 14 days prior to randomization. Vaccinations are discussed in more detail in Section 8.2.

Individuals who consent to participate in the study but who do not initially meet all the requirements as outlined in the inclusion and exclusion criteria are not able to be enrolled; however, these individuals may be held in the screening period until such time that they meet inclusion requirements and are able to be enrolled. In this case, additional screening visits and/or repeat screening assessments may be conducted, as needed, to establish eligibility. The medical monitor will contact the investigator to identify screening activities related to the confirmation of participant eligibility that must be repeated.

#### 6.1.1.1. Rescreening

In the event that rescreening occurs and the participant has not remained in the screening period (ie, is a screening failure), the individual is required to reconsent and must be assigned a new identification number. For rescreened participants, the historic biopsy (within 28 weeks before randomization), chest radiography, vaccination, ophthalmologic evaluations, and measured GFR in the previous screening period may be considered as validated testing for rescreening.

### 6.1.2. Part 2: Randomized Controlled Period (26 Weeks)

Approximately 80 to 100 participants who meet all of the inclusion criteria and none of the exclusion criteria will be randomly assigned in a 1:1 ratio to receive either pegcetacoplan or placebo. Randomization will be performed as described in Section 8.5.1.

- **Pegcetacoplan group**: Participants will receive SC pegcetacoplan twice weekly.
- **Placebo group**: Participants will receive SC placebo infusions twice weekly.
- Every effort will be made in both groups to minimize changes to medications related to C3G/IC-MPGN disease.
- Triplicate FMU samples will be collected at each visit, and an additional triplicate FMU uPCR sample will be collected during week 25.
- Safety and efficacy will be assessed.
- At the end of the RCP, there will be a renal biopsy, which is required for participants aged 18 years and older to advance to the open-label period (Part 3). Participants younger than 18 years are not required to provide renal biopsies and may advance to the open-label period upon completion of all assessments for weeks 24 through 26 other than the renal biopsy. Biopsy requirements are discussed in more detail in Section 10.5.

### 6.1.3. Part 3: Open-Label Period (26 Weeks)

At the end of the RCP, participants from both groups will proceed to the 26-week open-label period, in which all participants will be treated with pegcetacoplan twice weekly. Open-label dosing will begin at the first visit after the week 26 renal biopsy for adults and any adolescents providing renal biopsies. Open-label dosing for adolescents not providing renal biopsies should start at the week 26 visit. At the end of the open-label period, there will be an optional renal biopsy. Participants younger than 18 years who did not have a baseline biopsy within 28 weeks of day 1 will not have a biopsy at the end of the open-label period. Upon completion of the open-label period, participants will enter the follow-up period (Part 4) unless they enter a long-term extension study.

### 6.1.4. Part 4: Follow-up Period (8 Weeks)

After completion of the open-label period, participants who would benefit from continuing to receive pegcetacoplan, in the investigator's opinion, may roll over into a long-term extension study. Participants who do not enter a long-term extension study will discontinue pegcetacoplan treatment and complete the 8-week follow-up period. In addition, any participant who discontinues study drug during the RCP or open-label period should complete follow-up activities, as described in Section 7.3.1.

## Table 2: Schedule of Activities

| Study period | Screening period[a] | | Randomized controlled period | | | | | | | | Open-label period | | | | | | Follow-up period | | |
|---|---|---|---|---|---|---|---|---|---|---|---|---|---|---|---|---|---|---|---|
| **Study week** | **–10 to –4** | **–2** | **1** | **4** | **8** | **12** | **16** | **20** | **24** | **26** | **28** | **32** | **36** | **42** | **48** | **52** | **54** | **56** | **60 Exit** |
| **Study day** | **–70** | **–14** | **1** | **28** | **56** | **84** | **112** | **140** | **168** | **182** | **196** | **224** | **252** | **294** | **336** | **364** | **378** | **392** | **420** |
| **Study visit** | **1** | **2** | **3** | **4** | **5** | **6** | **7** | **8** | **9** | **10** | **11** | **12** | **13** | **14** | **15** | **16** | **17** | **18** | **19** |
| **Visit window (± days)** | **N/A[b]** | | **0** | **3** | **7** | **7** | **7** | **7** | **3** | **3** | **3** | **7** | **7** | **7** | **7** | **7** | **3** | **3** | **7** |
| **<u>Assessments</u>** | | | | | | | | | | | | | | | | | | | |
| Informed consent | X | | | | | | | | | | | | | | | | | | |
| Demographics | X | | | | | | | | | | | | | | | | | | |
| Medical history | X | | | | | | | | | | | | | | | | | | |
| Post-transplant immunosuppression plan documentation[c] | | X | | | | | | | | | | | | | | | | | |
| Inclusion/exclusion | X | X | X | | | | | | | | | | | | | | | | |
| Vaccination[d] | | X[b] | | | | X | | | | | | | | | | | | | |
| Adverse events | X | X | X | X | X | X | X | X | X | X | X | X | X | X | X | X | X | X | X |
| Concomitant medications | X | X | X | X | X | X | X | X | X | X | X | X | X | X | X | X | X | X | X |
| Physical examination (full)[e] | X | | X | | | | | | | X[e] | | | | | | X | | | |
| Physical examination (brief)[e] | | X | | X | X | X | X | X | X | | X[e] | X | X | X | X | | X | X | X |
| 12-lead ECG | X | | X | | X | | X | | X | X | X | | X | | X | X | X | | X |
| Chest radiography | | X[b] | | | | | | | | X | | | | | | X | | | |
| Renal biopsy[f] | X[b] | | | | | | | | | X[g] | | | | | | X[h] | | | |
| Randomization | | | X | | | | | | | | | | | | | | | | |
| Study drug administration[i] | | | X | X | X | X | X | X | X | X | X | X | X | X | X | X | | | |
| Infusion site/pump safety assessment[j] | | | X | X | X | X | X | X | X | X | X | X | X | X | X | X | X | | |
| Vital sign measurements[k] | X | X | X | X | X | X | X | X | X | X | X | X | X | X | X | X | X | X | X |
| HRQoL[l] | | X | | | | | | | | X | | | | | | X | | | |

## Table 2: Schedule of Activities

| Study period | Screening period[a] | | Randomized controlled period | | | | | | | | Open-label period | | | | | | Follow-up period | | |
|---|---|---|---|---|---|---|---|---|---|---|---|---|---|---|---|---|---|---|---|
| **Study week** | **–10 to –4** | **–2** | **1** | **4** | **8** | **12** | **16** | **20** | **24** | **26** | **28** | **32** | **36** | **42** | **48** | **52** | **54** | **56** | **60 Exit** |
| **Study day** | **–70** | **–14** | **1** | **28** | **56** | **84** | **112** | **140** | **168** | **182** | **196** | **224** | **252** | **294** | **336** | **364** | **378** | **392** | **420** |
| **Study visit** | **1** | **2** | **3** | **4** | **5** | **6** | **7** | **8** | **9** | **10** | **11** | **12** | **13** | **14** | **15** | **16** | **17** | **18** | **19** |
| **Visit window (± days)** | **N/A[b]** | | **0** | **3** | **7** | **7** | **7** | **7** | **3** | **3** | **3** | **7** | **7** | **7** | **7** | **7** | **3** | **3** | **7** |
| **Urine** | | | | | | | | | | | | | | | | | | | |
| 24-hour urine collection[m] | X | | | | | X | | | X | | | | X | | X | | | | X |
| Triplicate FMU uPCR[n] | X | X | X[o] | X | X | X | X | X | X | X | X | X | X | X | X | X | X | X | X |
| In-clinic (random) uPCR | X | X | X | X | X | X | X | X | X | X | X | X | X | X | X | X | X | X | X |
| Urinalysis (dipstick & microscopic)[p] | X | | | | | | | | | | | | | | | | | | |
| Urine pregnancy test | | X | X | X | X | X | X | X | X | X | X | X | X | X | X | X | X | X | X |
| **Blood** | | | | | | | | | | | | | | | | | | | |
| Hematology[p] & chemistry[p] | X | X | X | X | X | X | X | X | X | X | X | X | X | X | X | X | X | X | X |
| PK sample collection | | | X | X | X | X | X | X | X | X | X | X | X | X | X | X | X | X | X |
| ADA assays[q] | | | X | X | | | X | | X | | | | X | | | X | X | | X |
| Serum complement profile[p] | X | X | X | X | X | X | | X | X | X | X | X | X | | X | X | X | X | X |
| Plasma complement profile[p] | X | | X | | X | | X | | X | X | X | | X | | X | X | X | | X |
| eGFR[r] | X | X | X | X | X | X | X | X | X | X | X | X | X | X | X | X | X | X | X |
| Pregnancy (β-HCG) | X | | | | | | | | | | | | | | | | | | |
| Screening assays[s] | X | | | | | | | | | | | | | | | | | | |
| **Optional assessments** | | | | | | | | | | | | | | | | | | | |
| Ophthalmologic evaluations[t] | | X[t] | | | | | | | | | | | | X[t] | | | | | |
| Measured GFR[u] | | | X[u] | | | | | | | X[u] | | | | X[u] | | | | | |

## Table 2: Schedule of Activities

Abbreviations: Ab = antibodies; ADA = antidrug antibodies; AE = adverse events; AH50 = 50% alternative hemolytic complement pathway activity; ANA = antinuclear antibodies; ANCA = antineutrophil cytoplasmic antibodies; β-HCG = beta human chorionic gonadotropin; CH50 = 50% classical hemolytic complement pathway activity; ECG = electrocardiogram; eGFR = estimated glomerular filtration rate; EQ-5D-5L = 5-Level EuroQol-5 Dimension; FACIT-Fatigue = Functional Assessment of Chronic Illness Therapy–Fatigue Scale; FMU = first-morning spot urine; FSH = follicle-stimulating hormone; GFR = glomerular filtration rate; HIV = human immunodeficiency virus; HRQoL = health-related quality of life; KDQOL = Kidney Disease Quality of Life; N/A = not applicable; PGIC = Patient Global Impression of Change; PK = pharmacokinetic; SC = subcutaneous; SD-OCT = spectral domain optical coherence tomography; SPEP = serum protein electrophoresis; uPCR = urine protein-to-creatinine ratio; WPAI = Work Productivity and Activity Impairment.

**NOTE:** When multiple assessments/procedures occur at the same visit, order should be: vital signs, ECGs, blood collection/sampling, study drug dosing.

a. All visit 2 assessments need not occur in a single visit and visit 2 can be split into multiple visits (eg, visit 2a, visit 2b).

b. Vaccinations, baseline renal biopsy, and screening chest radiography can occur any time during screening after confirmation of eligibility based on visit 1 data.

c. For transplant participants only. Must be documented prior to assignment to study treatment.

d. Vaccine series should be initiated at least 14 days prior to randomization; additional vaccines may be required at visit 6. Please see Section 8.2 for more details on vaccination requirements. Vaccination serum samples will be collected on the day of vaccination before receiving vaccinations on that visit. These samples will be analyzed to evaluate response to vaccinations in the event that the participant has a positive infection for *Streptococcus pneumoniae*, *Neisseria meningitidis*, or *Haemophilus influenzae*.

e. A full physical examination is required at visits 1, 3 (week 1), 10 (week 26), and 16 (week 52). A full physical examination should also be conducted on the first day of open-label dosing, if that is not at the week 26 visit. Brief physical examinations, including weight (kg) and assessment of edema, will be conducted at all other visits noted. A symptom-driven physical examination may be performed at any time, at the investigator's discretion. Body height (cm) will be measured only during screening for adults but will be measured at screening and every 12 weeks throughout the study for adolescents; weight (kg) will be measured throughout study, during brief and full physical examinations for all participants. Both body weight and height will be assessed without shoes on; height will be measured using a calibrated stadiometer. Edema should be assessed at every visit.

f. Renal biopsies will not be required for participants younger than 18 years provided that they have adequate previous renal biopsies to establish the diagnosis as per the central pathology laboratory.

g. The week 26 renal biopsy need not occur on the same day as all other assessments for that visit. However, the week 26 triplicate FMU uPCR collections must occur before the renal biopsy. In addition, the week 26 biopsy must occur before the first visit of the open-label period at week 28. Participants younger than 18 years are not required to provide renal biopsies and may advance to the open-label period upon completion of all assessments for weeks 24 through 26 other than the renal biopsy.

h. The week 52 renal biopsy is optional for all participants. If performed it should be after collection of the week 48 24-hour urine and FMU samples, and not more than 8 weeks after the week 52 visit. In the event that a participant has a renal biopsy as part of their clinical management within this window, it may serve as the week 52 biopsy provided that it includes the required components for this study.

i. Study drug will be self-administered by the participant or administered by their caregiver, after receiving appropriate training and sign-off by a research nurse (or other qualified personnel) in their first treatment week, as described in Section 9.5. Once qualified, the participant or caregiver should administer study drug at site visits (as done at home) on those days when a clinic visit occurs on a dosing day. If a home nurse is administering study drug on nonvisit days, the site staff may administer study drug on days of site visits.

j. Between site visits, participants will be instructed to report any infusion site reactions to the study staff. Pump use safety will be reviewed by licensed health care professionals (eg, investigator or nurse) for each study drug administration at clinic visits and during at-home qualification.

k. Vital signs should be measured a maximum of 2 hours before study drug infusion. On day 1 and on the first day of open-label dosing (week 26 or week 28, depending on whether a week 26 renal biopsy is required and when it is performed), vital signs should also be measured approximately 30 minutes to 1 hour after the first infusion of study drug dosing, timed from the completion of the study drug administration. Blood pressure and heart rate should be evaluated after the participant has been resting in a seated position for at least 5 minutes, except when they are supine or semireclined because of study procedures and/or AEs, or if deemed necessary by the investigator.

l. FACIT-Fatigue, EQ-5D-5L, KDQOL, PGIC, and WPAI.

m. The screening 24-hour urine collection may be done any time between screening visit 1 and screening visit 2. After week 1, collections should be within ±1 week of the visit (except for the week 24 collection, which cannot be earlier than week 24). Courier arrangements can be made to pick up the collection container from the participant, or the participant may return the container directly to the site.

**Table 2: Schedule of Activities**

n. Triplicate FMU samples will be collected by the participant at home on 3 consecutive days throughout the duration of the study. These should be the first urinary output of the day. An additional triplicate FMU uPCR sample will be collected at week 25. Samples should be collected within ±1 week of the clinic visit (except for weeks 24 through 26, when sample collection should be within ±3 days of the clinic visit). Courier arrangements can be made to pick up the collection containers from the participants, or the participant may return the containers directly to the site. At every visit, enough uPCR collection containers should be dispensed to the participant to enable all at-home uPCR collections until the next clinic visit.

o. The day 1 triplicate FMU uPCR samples should be collected before the first dose of study drug (eg, day –2, day –1, and before dosing on day 1).

p. Serum complement profile includes AH50, CH50, and C3NeF; C3NeF will only be assayed in samples collected at the baseline (screening) visits. Plasma complement profile includes C3a, C3b/iC3b, C5a, and sC5b-9. See laboratory assessments (Table 4) for more details.

q. The day 1 samples should be collected before dosing with study drug. Participants who discontinue dosing will have ADA samples collected at 2 and 8 weeks after the last treatment. Participants who have a treatment-emergent or treatment-boosted ADA response at any time will have ADA samples collected approximately every 6 months until the antibody levels revert to baseline.

r. eGFR will be calculated using the Chronic Kidney Disease–Epidemiology Collaboration creatinine equation for adults or the Bedside Schwartz equation for adolescents. For each participant, eGFR will be calculated using the same formula for the duration of the study; the choice of formula will be determined by the participant's age at study entry.

s. Serum FSH (to be measured in female participants only), hepatitis B panel, hepatitis C panel, HIV antibodies, SPEP (adult participants only), ANA, and ANCA (see Table 4 and laboratory manual for more details).

t. Ophthalmologic evaluations are optional and will be performed at selected sites. If evaluations are performed, each participant should have a baseline ophthalmologic evaluation, including a basic ophthalmologic examination, SD-OCT, and color fundus photography, at an approved ophthalmologic clinical site at any time during the screening period. For participants who have drusen before pegcetacoplan administration, a follow-up ophthalmologic evaluation, including an SD-OCT and color fundus photography, should occur at a convenient time between weeks 42 and 52.

u. Measured GFR is an optional assessment that, if performed, should occur at 3 time points: once on day 1 or within 10 weeks before day 1, again at week 26, and a third time between week 42 and week 52, inclusive. Measured GFR should only be done at sites where it is routinely performed, as per the site's standard protocol.

## 6.2. Number of Participants

Approximately 80 to 100 patients with clinical and pathologic evidence of C3G or IC-MPGN will be randomized into this study, at least 78 of whom will have native kidney disease and up to 22 of whom may have posttransplant recurrence of C3G or IC-MPGN. At least 63 participants with C3G in native kidneys will be enrolled. At least 10 adolescent participants (aged 12-17 years) will be enrolled; adolescent participants may be patients with either native kidney disease or posttransplant disease recurrence. When the study is approaching the target enrollment, the sponsor will notify sites and investigators of the anticipated enrollment closure. Potentially eligible participants who have started screening may complete screening activities and enroll in the study if they meet eligibility criteria, even if this results in enrollment of more than the planned maximum of 100 participants.

## 6.3. Treatment Assignment

All participants who meet all the inclusion criteria and none of the exclusion criteria will be enrolled in the study, until such time that the intended enrollment has been met. After confirmation of study eligibility, participants will be randomized in a ratio of 1:1 to receive pegcetacoplan or placebo as described in Section 8.5.1.

Following completion of the RCP, all participants will receive pegcetacoplan starting on the first visit of the open-label period, after which they will enter the follow-up period, unless they enroll in a long-term extension study.

## 6.4. End of Study

Participants will be considered to have completed the study when they have completed the exit visit activities described in the schedule of activities (Table 2), when they have enrolled in a subsequent study of pegcetacoplan, or when they have discontinued study treatment and completed the activities described in Section 7.3.1.

The end of the study will be the last participant's last visit or the last participant's last scheduled visit/assessment as indicated in the schedule of activities (Table 2) or as requested by the sponsor.

# 7. SELECTION AND WITHDRAWAL OF PARTICIPANTS

## 7.1. Inclusion Criteria

1. Aged at least 18 years; where approved, adolescents (aged 12-17 years) weighing at least 30 kg may also be enrolled.
2. A diagnosis of primary C3G or IC-MPGN (with or without previous renal transplant).
3. Evidence of active renal disease, based on one or more of the following:
   a. In adults or adolescents with a baseline renal biopsy (either one collected during screening or a historic biopsy collected within 28 weeks prior to randomization), at least 2+ C3c staining on the baseline renal biopsy
   b. In adolescents not providing a baseline renal biopsy, at least one of the following:
      - Plasma sC5b-9 level above the upper limit of normal during screening
      - Serum C3 below the lower limit of normal (LLN) during screening
      - Presence of an active urine sediment during screening, as evidenced by hematuria with at least 5 red blood cells per high-power field and/or red blood cell casts on routine local or central microscopic analysis of urine
      - Presence of C3 nephritic factor within 6 months of screening, based on central laboratory results or medical history
4. No more than 50% global glomerulosclerosis or interstitial fibrosis on the baseline biopsy for adult participants or adolescent participants providing a baseline biopsy.
5. At least 1 g/day of proteinuria on a screening 24-hour urine collection and a uPCR of at least 1000 mg/g in at least 2 FMU samples collected during screening.
6. eGFR $\geq$30 mL/min/1.73 $m^2$ calculated by the CKD-EPI creatinine equation for adults or the Bedside Schwartz equation for adolescents.
7. Stable regimen for C3G/IC-MPGN treatment, as described below:
   a. Angiotensin-converting enzyme inhibitor, angiotensin receptor blocker, and/or sodium-glucose cotransporter-2 inhibitor therapy that is stable and optimized, in the opinion of the investigator, for at least 12 weeks prior to randomization
   b. Stable doses of other medications that can affect proteinuria (eg, steroids, mycophenolate mofetil, and/or other allowed immunosuppressants that the participant is receiving for treatment of C3G or IC-MPGN) for at least 12 weeks prior to randomization.
   c. If a participant is on prednisone (or other systemic corticosteroid) for C3G or IC-MPGN treatment, the dosage is stable and no higher than 20 mg/day (or equivalent dosage of a corticosteroid other than prednisone) for at least 12 weeks prior to randomization.
8. Have received vaccinations against *S pneumoniae*, *N meningitidis* (types A, C, W, Y, and B), and *H influenzae* (type B) as per ACIP recommendations for adults or children with complement deficiencies. Vaccination series should be initiated at least 14 days prior to randomization. Vaccination is mandatory unless documented evidence exists that participants are nonresponders to vaccination.

9. Female participants of childbearing potential, defined as any women who have experienced menarche and who are not permanently sterile or postmenopausal, must have negative blood pregnancy tests at screening (and negative urine pregnancy tests on day 1) and must agree to use protocol-defined methods of contraception from screening through at least 90 days after receiving the last dose of pegcetacoplan.
10. Male participants must agree to use protocol-defined methods of contraception and agree to refrain from donating semen from screening through at least 90 days after receiving the last dose of pegcetacoplan.
11. Participants above the legal age of consent, in accordance with local regulations, must be willing and able to provide informed consent. The legally authorized representative of participants under the legal age of consent must be willing and able to provide informed consent; where appropriate, participants under the legal age of consent must also give their assent to participation in the study.
12. Willing and able to self-administer pegcetacoplan or have an identified caregiver who can perform the administration.

### 7.1.1. Acceptable Methods of Contraception

Women of childbearing potential (WOCBP) and male participants with female partners of childbearing potential will be instructed to practice an acceptable method of birth control throughout the duration of the study and for 90 days following the end of study drug administration.

A woman is considered to be of childbearing potential following menarche and until becoming postmenopausal, unless she is permanently sterile. Permanent sterilization methods include hysterectomy, bilateral salpingectomy, and bilateral oophorectomy. A postmenopausal state is defined as no menses for 12 months without an alternative medical cause.

Approved methods of contraception include:

- Combined (estrogen and progestogen containing) hormonal contraception associated with inhibition of ovulation:
    - oral, intravaginal, or transdermal
- Progestogen-only hormonal contraception associated with inhibition of ovulation:
    - oral, injectable, or implantable
- Intrauterine device
- Intrauterine hormone-releasing system
- Bilateral tubal occlusion
- Vasectomized partner (*provided that partner is the sole sexual partner of the WOCBP trial participant and that the vasectomized partner has received medical assessment of the surgical success*)
- Sexual abstinence (*defined as refraining from heterosexual intercourse during the entire period of risk associated with study treatments*)
- Male condom with or without spermicide (for male study participants with female partners of childbearing potential only)

Not all methods of contraception may be available in all of the countries in which this study is being conducted.

Note: Sexual abstinence is only accepted when it is the preferred and usual lifestyle of the participant.

## 7.2. Exclusion Criteria

1. Previous exposure to pegcetacoplan.
2. Evidence of improving renal disease in the 8 weeks prior to screening or during the screening period according to available data; improving renal disease is defined as >30% increase in eGFR or >50% decrease in proteinuria.
3. From a renal transplant participant, evidence of rejection that requires treatment in the baseline renal biopsy collected during screening.
4. C3G/IC-MPGN secondary to another condition (eg, infection, malignancy, monoclonal gammopathy, a systemic autoimmune disease such as systemic lupus erythematosus, chronic antibody-mediated rejection, or a medication), in the opinion of the investigator.
5. Current or prior diagnosis of HIV, hepatitis B, or hepatitis C infection or positive serology during screening that is indicative of infection with any of these viruses.
6. Body weight greater than 100 kg at screening.
7. Hypersensitivity to pegcetacoplan or to any of the excipients.
8. History of meningococcal disease.
9. Malignancy, except for the following:
    a. Cured basal or squamous cell skin cancer
    b. Curatively treated in situ disease
    c. Malignancy-free and off treatment for ≥5 years
10. Severe infection (eg, requiring IV antibiotic therapy) within 14 days prior to the first dose of pegcetacoplan.
11. An absolute neutrophil count <1000 cells/$mm^3$ at screening.
12. Significant other renal disease that would, in the opinion of the investigator, confound interpretation of study results.
13. Participation in any other investigational drug trial or exposure to other investigational agent, device, or procedure within 30 days or 5 half-lives from the last dose of investigational agent (whichever is longer) prior to screening period.
14. Use of rituximab, belimumab, or any approved or investigational anticomplement therapy other than pegcetacoplan within 5 half-lives of that product prior to the screening period.
15. Female participants who are pregnant or who are currently breastfeeding and are unwilling to discontinue for the duration of the study and for at least 90 days after the final dose of study drug.

16. Inability to cooperate or any condition that, in the opinion of the investigator, creates an undue risk for the participant by participating in the study or is likely to confound interpretation of the study results.
17. Evidence of ongoing drug or alcohol abuse or dependence, in the opinion of the investigator.
18. Presence or suspicion of severe infection during the screening period (including but not limited to recurrent or chronic infections) that, in the opinion of the investigator, may place the participant at unacceptable risk by study participation.
19. Known or suspected hereditary fructose intolerance.

## 7.3. Discontinuations and Participants Lost to Follow-up

### 7.3.1. Early Treatment Discontinuation and Study Withdrawal

A participant may discontinue study treatment or withdraw from the study at any time, for any reason, without prejudice to his/her future medical care. The investigator or sponsor may discontinue study treatment or withdraw the participant from the study at any time (eg, in the interest of the participant's safety). If a participant discontinues study treatment or is withdrawn from the study for any reason, the study site must immediately notify the medical monitor.

Once withdrawn from the study, the participant may not reenter the study.

Participants who discontinue study treatment prior to the end of the RCP should be encouraged to complete the remaining visits for the RCP. If discontinuing study treatment after week 18, participants should also complete the activities described for the exit visit in the schedule of activities (Table 2) approximately 8 weeks after their last dose of study drug. If the participant is unwilling or unable to complete the exit visit activities, they should be encouraged to complete the other follow-up visits and procedures described in the schedule of activities (Table 2).

Participants who discontinue study treatment during the open-label period should enter the follow-up period of the study, including all follow-up visits and procedures through study completion, unless unwilling, unable, or consent has been withdrawn. Participants who wish to fully withdraw from the study during the open-label period but prior to the week 52 visit and are unwilling or unable to complete the follow-up visits and procedures should complete the activities described for the exit visit in the schedule of activities (Table 2) at their last visit.

### 7.3.2. Reasons for Discontinuation of Treatment or Withdrawal From the Study

The date of and reason for treatment discontinuation must be determined by the investigator and recorded in the participant's medical record. If it is determined that there was more than one reason for the discontinuation, each reason should be recorded in the source document and the most clinically relevant reason, as determined by the investigator, should be noted. Treatment will be discontinued if any of the following occur:

- Any SAE, clinically significant AE, severe laboratory abnormality, intercurrent illness, or other medical condition (including infections) or circumstance that indicates to the investigator that continued participation is not in the best interest of the participant.

- Participant withdrawal of consent: at any time, a participant's participation in the study may be terminated at his or her request or on the basis of the investigator's clinical judgment. The reason for participant withdrawal will be noted in the participant's source document.
- Participant fails to adhere to the protocol requirements (eg, drug noncompliance, failure to return for defined number of visits, failure to meet randomization criteria), such that continued participation in the study is not in the best interest of the participant (in the opinion of the investigator) or compromises the ability of the sponsor to appropriately analyze the data from the study.
- Participant is lost to follow-up: the participant stopped coming for visits, and study personnel were unable to contact the participant.
- Termination of the study by the sponsor or regulatory authorities.
- Pregnancy, as indicated in Section 11.2.4.

Any participant who discontinues treatment early, or is withdrawn from the study early, because of a TEAE, whether serious or nonserious, will be followed until the TEAE resolves (returns to normal or baseline values) or stabilizes, or until it is judged by the investigator to no longer be clinically significant.

Participants for whom study treatment is discontinued should be encouraged to complete additional activities as described in Section 7.3.1, unless unwilling, unable, or consent has been withdrawn. If consent is withdrawn, no further study evaluations are to be performed and no attempts are to be made to collect additional data.

Additionally, the sponsor may stop the study at any time for safety, regulatory, legal, or other reasons aligned with Good Clinical Practice (GCP). This study may be terminated at the discretion of the sponsor or any regulatory agency. An investigator may elect to discontinue or stop the study at his/her study site for any reason, including safety or low enrollment.

### 7.3.3. Lost to Follow-up Prior to Last Scheduled Visit

A minimum of 3 documented attempts must be made to contact any participants lost to follow-up at any time prior to the last scheduled contact (office visit or telephone contact). At least 1 of these documented attempts must include a written communication sent to the participant's last known address via courier or mail (with an acknowledgment of receipt request) asking that he/she return to the site for final safety evaluations and to return any study drug.

Should the participant continue to be unreachable, he/she will be considered to have withdrawn from the study.

### 7.3.4. Replacement of Participants

Participants who discontinue the study will not be replaced.

# 8. TREATMENT OF PARTICIPANTS

## 8.1. Study Interventions

All participants will receive SC infusions of pegcetacoplan or matching volumes of placebo twice weekly. The planned dosing regimens for pegcetacoplan and the matching volume of placebo are presented in Table 3. All adult participants (regardless of weight), and adolescent participants who weigh at least 50 kg, will receive 20-mL SC infusions. Adolescent participants who weigh at least 35 kg but less than 50 kg will receive a reduced infusion volume (12 mL for the first infusion and 15 mL for each infusion thereafter). Adolescent participants who weigh at least 30 kg but less than 35 kg will receive a further reduced infusion volume (10 mL for the first 2 infusions and 12 mL twice weekly thereafter). As described in Section 11.1.3, participant weight will be assessed at each visit; if an adolescent participant's weight has changed, the dose and infusion volume should be adjusted accordingly.

**Table 3: Planned Dosing Regimens (Pegcetacoplan and Placebo)**

| Weight | First dose (infusion volume) | Second dose (infusion volume) | Maintenance dosing regimen (infusion volume) |
|---|---|---|---|
| All adult participants, adolescent participants ≥50 kg | 1080 mg (20 mL) | 1080 mg (20 mL) | 1080 mg twice weekly (20 mL) |
| Adolescent participants 35 to <50 kg | 648 mg (12 mL) | 810 mg (15 mL) | 810 mg twice weekly (15 mL) |
| Adolescent participants 30 to <35 kg | 540 mg (10 mL) | 540 mg (10 mL) | 648 mg twice weekly (12 mL) |

Participants and/or their caregivers will be trained to administer the infusions, as described in Section 9.5.

If a participant misses any regularly scheduled doses for any reason, all efforts should be made to restart dosing as soon as possible to reach and maintain therapeutic steady-state concentrations of pegcetacoplan. A single missed dose should be administered as soon as possible, regardless of whether the missed dose is administered on a regularly scheduled dosing day. Dosing should then continue twice weekly at the prescribed dose level (see Table 3 above) according to the original dosing schedule, even if this results in an interval of less than 3 days between 2 doses. If multiple doses are missed, dosing should resume as soon as possible at the prescribed dose level (see Table 3 above) according to the original dosing schedule. The medical monitor should be notified of any gaps in dosing.

## 8.2. Vaccinations

To receive study drug, participants will be required to be vaccinated as follows on the basis of ACIP recommendations for adults or children with complement deficiencies (available at https://www.cdc.gov/vaccines/schedules/hcp/index.html) or other similar local guidelines.

- *N meningitidis* types A, C, W, and Y: First dose at least 14 days prior to randomization with a second dose 2 months later, and then boosters every 5 years.

- *N meningitidis* type B: First dose at least 14 days prior to randomization with a second dose after at least 1 month. First booster dose 1 year later, and then additional booster doses every 2 to 3 years.
- *S pneumoniae*: pneumococcal conjugate vaccine (PCV13, PCV15, or PCV20, depending on participant age and vaccine availability) and/or pneumococcal polysaccharide vaccine 23 (PPSV23). Adults who have not previously received any pneumococcal vaccine may receive PCV20, if the site is able to procure it, and a subsequent dose of PPSV23 would not be required. Multiple scenarios exist depending on previous vaccination history and investigators should consult ACIP guidelines for adults or children with complement deficiencies or other similar local guidelines.
- *H influenzae* type B: Documentation of childhood vaccination or one dose at least 14 days prior to randomization.

Vaccination is mandatory, unless documented evidence exists that participants have received the recommended vaccinations or are nonresponders to vaccination. For participants who do not have this documented evidence, the required missing vaccination(s) will be administered as needed to bring participants up to date. The investigator will discuss with the medical monitor any individual participant circumstances relevant to the vaccination requirements that would make the above schedule not possible or reasonable, of if the investigator has any questions or concerns about the vaccination requirements. If local or national guidelines for immunizations differ from the ACIP recommendations, the differences should be discussed with the medical monitor to determine an appropriate course of vaccinations. On an ongoing basis, including upon entry into a long-term extension, participants should be reevaluated for the need for any additional vaccinations or boosters on the basis of ACIP recommendations.

Vaccination serum samples will be collected on the day of vaccination before receiving vaccinations on that visit. These samples will be analyzed to evaluate response to vaccinations if the participant tests positive for infection with *S pneumoniae*, *N meningitidis*, or *H influenzae*.

### 8.2.1. Empiric Antibiotic Treatment for Possible Infection

Body temperature, vital signs, and relevant blood parameters will be monitored regularly throughout the study to assess for signs of infection. The investigator should be contacted immediately in the event of a suspected infection for guidance on appropriate action to be taken. Action to be taken may include empiric administration of a broad-spectrum antibiotic to cover possible serious infections, such as meningococcus.

## 8.3. Concomitant Medications

All medications administered (including over the counter or prescription medicines, vitamins, and/or herbal supplements) and all procedures performed within 12 weeks of screening will be collected as prior medications and procedures. Medications administered and procedures performed from the time of informed consent through the final visit (the week 60 exit visit, an exit visit following early discontinuation as described in Section 7.3.1, or the final visit before transition to the long-term extension study, as applicable) are regarded as concomitant and will be documented.

### 8.3.1. Treatment Regimens for C3G or IC-MPGN

Participants should be on stable doses of all medications relevant to their renal disease for at least 12 weeks prior to randomization, as described in Section 7.1. During the screening period and the RCP, changes to the baseline treatment regimens for C3G/IC-MPGN (including medications, dietary restrictions, and lifestyle modifications) should be minimized to the extent possible and made only when required for the well-being of the participant (with the exception of posttransplant immunosuppression—see below). During the open-label period, if the investigator plans to make changes to the baseline treatment regimen, these should be approved by the sponsor before implementation.

#### 8.3.1.1. Posttransplant Immunosuppression

If the participant is in a posttransplant time frame in which immunosuppression or other transplant-related medication adjustments are anticipated, these adjustments should still occur, adhering to the center's standard protocol for posttransplant care. For all posttransplant participants, the plan for posttransplant immunosuppression for the duration of the study should be documented prior to randomization. Any deviation during the study from the prespecified plan should be documented with a justification for the change.

Posttransplant participants with evidence of rejection on the baseline biopsy collected during screening that requires treatment will need to undergo their antirejection treatment, and then can be reconsidered for pegcetacoplan dosing, but will need to meet all other study eligibility criteria, including a repeat biopsy to serve as the baseline (refer to Section 10.5 for further details). Participants with evidence of subclinical rejection on the biopsy collected during screening may proceed with pegcetacoplan treatment, at the discretion of the investigator, provided no treatment is initiated for the rejection.

### 8.3.2. Rescue Therapy

In the event of an increase in serum creatinine to at least 2× the baseline level that in the opinion of the investigator is due to the underlying C3G/IC-MPGN disease, rescue therapy may be considered, such as high-dose corticosteroids and/or C5 inhibitor treatment.

If the participant receives rescue therapy that is not a prohibited concomitant medication, then they will be allowed to remain on study drug. If a participant receives rescue therapy that is a prohibited concomitant medication, they will be discontinued from study drug, as described in Section 8.3.3.

### 8.3.3. Prohibited Medications

The use of rituximab, belimumab, or any approved or investigational anticomplement therapy other than pegcetacoplan (including, but not limited to, eculizumab and/or ravulizumab) is prohibited within 5 half-lives of that product prior to screening and for the duration of the study.

If a participant receives a prohibited concomitant medication, they will be discontinued from study drug and the procedures described in Section 7.3 should be followed.

Analysis of results from participants receiving prohibited medications is described in Table 8 and Table 9.

## 8.4. Treatment Compliance

Participants must be instructed to bring their empty/unused study drug packaging and diaries to every visit. The pharmacist/nominated person will record drug accountability information in the interactive response technology.

## 8.5. Measures to Minimize Bias: Study Treatment Assignment

### 8.5.1. Randomization

Participants will be randomized to receive pegcetacoplan or placebo in a ratio of 1:1 via a computer-generated randomization schedule. The randomization will be performed centrally.
To achieve balance between the arms, 2 stratification factors will be applied to the randomization. The first stratification factor examines participants with posttransplant recurrence versus nontransplant participants; at least 78 participants with C3G or IC-MPGN in a native kidney will be enrolled. This is followed by the second stratification factor, which examines participants with baseline renal biopsies (either collected during screening or a historic biopsy collected within 28 weeks prior to randomization) versus participants without baseline renal biopsies.
The stratification will ensure balance between treatment groups for the stratification factors.

### 8.5.2. Blinding

Dosing will be double-blinded in the RCP; the open-label period is not blinded. All central evaluations (including evaluation of renal biopsies by the central pathologist) will be blinded to treatment assignment.

Participants, the sponsor, investigators, and all study site personnel conducting study-related activities (including carrying out study procedures, evaluating participants, and entering and/or evaluating study data) will remain blinded to treatment allocations during the RCP at least until all participants have completed the week 26 assessments and the RCP portion of the database has been locked. Although treatment in the open-label period is not blinded, investigators, study site personnel, and participants will remain blinded to the RCP treatment assignment until the blind has been broken. Only the drug supply distributor/logistics personnel who are not site personnel will be unblinded to study treatment. These individuals are not allowed to discuss treatment and/or participant outcome with blinded study staff, including the evaluating physician. Active and placebo product labels will not include any information which would unblind the treatment assignment during the RCP, as described in Section 9.2.2.

#### 8.5.2.1. Study Drug Unblinding

Except as noted below, investigators shall not break the study blind. If knowledge of the participant's treatment assignment is necessary for clinical management, the investigator should first attempt to contact the medical monitor or designee to discuss the need for unblinding to occur. The medical monitor or designee will be available at all times for such discussions. If the investigator is unable to reach the medical monitor or designee, or in the case of a medical emergency, the investigator should use their best judgment based on the nature and urgency of the clinical situation, and if necessary may proceed with unblinding without having successfully contacted the medical monitor or designee. If a participant's treatment assignment is unblinded under these circumstances, the medical monitor and study coordinator must be notified within 24 hours.

Information describing the unblinding (eg, date and time of the call to the medical monitor by the investigator, reason for unblinding, and date and time of unblinding) must be clearly recorded in the participant's study file. In addition, the investigator should consider whether the clinical event prompting unblinding should be considered an AE or an SAE according to the definitions in Section 11.2.1; if so, such events must be recorded and reported as described in Section 11.2.2 and Section 11.2.3.

# 9. STUDY DRUG MATERIALS AND MANAGEMENT

## 9.1. Study Drug

The investigational product is pegcetacoplan (APL-2). The study drug may be pegcetacoplan or placebo during the RCP, depending on the treatment assignment. The study drug will be pegcetacoplan during the OLP.

Pegcetacoplan will be provided as a sterile solution in single-use glass vials. Each vial contains an isotonic sterile solution of pegcetacoplan 1080 mg/20 mL (54 mg/mL) in 10 mM acetate buffer, pH 5.0, containing 4.1% sorbitol. Additional information is provided in the pegcetacoplan IB.

Placebo will be provided as a sterile solution of 10 mM acetate buffer, pH 5.0, containing 4.1% sorbitol supplied in single-use glass vials.

## 9.2. Study Drug Packaging and Labeling

### 9.2.1. Packaging

Both pegcetacoplan and placebo will be supplied in 20-mL glass vials. Please refer to the pharmacy manual for details.

Changes to sponsor-supplied packaging prior to dosing may not occur without full agreement, in advance, by the sponsor.

### 9.2.2. Labeling

Labels containing study information and pack identification are applied to the investigational product container.

All investigational product (pegcetacoplan drug product or placebo) is labeled with a minimum of the following items: protocol number, dosage form (including product name and quantity in pack), route of administration, directions for use, storage conditions, batch number and/or packaging reference, statements required per local regulations (eg, “For clinical trial use only”), and name and address of the sponsor.

Space is allocated on the label so that the site representative can record a unique participant identifier and date dispensed by the site to the participant.

Additional labels may, on a case-by-case basis, be applied to the investigational product to satisfy national, local, or institutional requirements but must not contradict the clinical study label, obscure the clinical study label, unblind the treatment assignment during the RCP, or identify the participant by name.

Additional labels may not be added without the sponsor’s prior full agreement.

## 9.3. Study Drug Storage

The study drug should be stored refrigerated at 2-8 °C, both at home and in the clinic. Temperature monitoring is required at the investigator site (or documented storage location) to ensure that the study drug is maintained within an established temperature range.

The investigator or appropriately qualified site staff will be responsible for the following:

- ensuring that the study drug is stored in a secure, limited-access location at the site
    - limited responsibility may be delegated to the pharmacy or member of the study team, but this delegation must be documented
- ensuring that the temperature is monitored throughout the duration of the study and that records are maintained

A pharmacist or appropriately qualified designated person will be responsible for the following:

- storing the study drug appropriately
- dispensing the vials of study drug to the participant
- entering the unique participant identifier on the study drug bottle/carton labels as they are distributed

When the participant receives the study drug from the site, it should be transported in a sponsor-approved bag or box, containing previously temperature-conditioned cold plates to ensure that the storage temperature (2-8 °C) is maintained. Temperature monitoring will not be required during transport or at the participant's residence, but a log will be kept for every infusion to ensure that all study drug was kept refrigerated.

With sponsor prior approval, investigational product and/or ancillary supplies may be shipped from the study site to a participant's designated location. Such shipments will only be implemented at sites where this activity is approved by the IRB/IEC and health authority (if required). Participant consent will be required prior to any participant information being provided to a courier. The responsibility to return both empty vials and any unused investigational product as described in Section 8.4 shall remain unchanged.

## 9.4. Infusion Supplies

The sponsor will supply syringes, vial adapters, infusion sets, and ambulatory syringe infusion pumps, as required. Refer to the pharmacy manual for further details.

## 9.5. Study Drug Administration

Study drug will be administered as an SC infusion. The infusion volume depends on the dose received, as described in Section 8.1.

The preferred site of infusion will be the anterior abdominal wall. If administration into the abdomen is not feasible, alternative appropriate sites are acceptable. Alternative sites include the anterior aspects of the thighs or upper arms (see Study Medication Administration Instructions—Participant's Guide for more details). It is advisable to change infusion site after each drug administration in order to avoid skin irritation. Research nurses or other appropriately qualified research personnel will administer the SC infusions (as needed) and will qualify and supervise the self-administration. Study drug administration may also be conducted by a caregiver or a member of the participant's household, etc, who will undergo the same training (training is not intended to be restricted to the participant).

These qualified personnel will be made available for a minimum of 6 days on treatment (2 doses) to ensure the participant or caregiver has been qualified to conduct administration, but duration could be shortened if qualification happens sooner or extended if the participant or caregiver requires further training. During qualification, the participant or caregiver must demonstrate to the research personnel their ability to safely and effectively administer study drug using the infusion pump. Following administration qualification, the participant or caregiver may administer the SC infusions without supervision. Once qualified, the participant or caregiver will continue to administer infusions at the clinic on those days when a clinic visit occurs on a dosing day and at an off-site location convenient to the participant on all other days. Self-administration or caregiver administration conducted at the clinic will be supervised to ensure that the participant or caregiver continues to remain compliant with the administration guidelines.

## 9.6. Study Drug Accountability

Accountability for the study drug at the study center is the responsibility of the investigator. The investigator will ensure that the study drug is used only in accordance with this protocol. Where allowed, the investigator may choose to delegate drug accountability responsibilities to a pharmacist or other appropriate individual.

Investigators will be provided with sufficient amounts of the study drug to carry out this protocol for the agreed number of participants. The investigator or designee will acknowledge receipt of the investigational product and placebo, documenting shipment content and condition. Accurate records of all study drug dispensed, used, returned, and/or destroyed must be maintained. Accountability records will include dates, quantities, batch/serial numbers, expiration dates (if applicable), and participant numbers. The sponsor or its designee will review study drug accountability at the study center on an ongoing basis during monitoring visits.

Study drug must not be used for any purpose other than the present study. Study drug that has been dispensed to a participant and returned unused must not be redispensed to a different participant.

The investigator is responsible for ensuring the retrieval of all returnable study supplies from participants.

## 9.7. Study Drug Handling and Disposal

All unused and used study drug vials should be retained at the center until they are inventoried by the study monitor. All used, unused, or expired study drug vials will be returned to the sponsor or its designee for destruction and destruction will be documented, or if authorized, disposed of at the study center per the center's standard operating procedures and documented.

# 10. ASSESSMENT OF EFFICACY

## 10.1. Retrospective Data Collection for eGFR and Measured Glomerular Filtration Rate

Retrospective data will be collected on glomerular filtration rate (GFR) to calculate historical eGFR for up to 3 years prior to study entry, depending on the date of diagnosis (for renal transplant recipients, this refers to diagnosis of recurrent disease, not diagnosis of the original disease in native kidneys). Three years of retrospective data will be collected to calculate eGFR for those diagnosed at least 3 years prior to randomization. Retrospective data for the period between randomization and diagnosis will be collected for those diagnosed less than 3 years prior to randomization. For historical eGFR calculations, data will be collected to enable calculation of eGFR by CKD-EPI (with serum creatinine) in adults and by the Bedside Schwartz equation in adolescents. Specifically, age, sex, race, height (adolescents only), serum creatinine, serum blood urea nitrogen (adolescents only), and serum cystatin C (if available) will be collected. For each participant, eGFR will be calculated using the same formula for the duration of the study; the choice of formula will be determined by the participant's age at study entry.

If a participant has historical data on measured GFR, then this data, including the method used to measure GFR, should also be collected (in addition to the data required to calculate eGFR in that participant) for the same timeframe.

## 10.2. 24-Hour Urine Collection

A 24-hour urine collection is defined as collection of all urine produced over a 24-hour period. As 24-hour urine collections will be done at home, participants will be provided with containers for the collection as well as an instruction sheet that details the collection procedure. The 24-hour urine collection should begin following the first urinary output for the day, and then continue for a 24-hour period. Samples should be collected within ±1 week of the clinic visit (except for the week 24 collection, which cannot be earlier than week 24).

In brief, the first urine (on the day of collection) must be discarded (as this is urine made prior to the start of the collection date/time). The time and date of this discarded void must be recorded, as it represents the start of the collection. The participant should then collect all urine over the next 24 hours. At the 24-hour time point, the participant should empty their bladder and collect this urine as the final void of the 24-hour urine collection. The date and time of this last collected void should be recorded, as it represents the end of the 24-hour urine collection.

There will be six 24-hour urine collections, at the visits outlined in the schedule of activities (Table 2):

- screening (between week –8 and week –4 and before the renal biopsy)
- week 12
- week 24 (before the week 26 renal biopsy)
- week 36
- week 48 (before the week 52 renal biopsy, if performed)
- week 60

If menses are occurring at the scheduled time of a 24-hour urine collection, then the collection should be delayed until completion of menses, with this information recorded in the participant's source document. Urine must be stored at 2 to 8 °C (ie, in refrigerator). Courier arrangements can be made by the site to pick up the collection container from the participant's home and return it to the site, or the participant may return it directly to the site. Total urine volume, total protein concentration, total albumin concentration, total β-2 microglobulin, and total creatinine concentration will be measured on the 24-hour urine collections, with calculation of total protein excretion, total albumin excretion, total creatinine excretion, total β-2 microglobulin excretion, uPCR, urinary albumin-to-creatinine ratio (uACR), and urinary β2-microglobulin–to-creatinine ratio (uBCR) based on the 24-hour collection.

## 10.3. Triplicate FMU Collections

Triplicate FMU collection is defined as a collection of first-morning urine on 3 consecutive mornings, with each morning urine sample collected into a separate container. Samples should be collected within ±1 week of the clinic visit (except for weeks 24 to 26, when sample collection should be within ±3 days of the clinic visit).

It is important that each of these collections represent the first urinary output upon awakening, after an extended period of rest. The date and time of each collected sample should be recorded. In the event that a participant has an atypical sleep/wake schedule, the appropriate timing for these spot urine collections may not be morning. In this case, the timing of the collection should be adjusted to ensure collection of the first urinary output after an extended period of rest (eg, at least 6 hours), and the alternate timing should be documented in the source document. If menses are repeatedly occurring at the scheduled times of a triplicate FMU collection, the collection dates should be adjusted to avoid collection during menses to the extent possible, with this information recorded in the participant's source document.

After FMU collection, the urine samples must be stored at 2-8 °C (ie, in a refrigerator). Courier arrangements can be made by the site to pick up the collection containers from the participants, or the participant may return them directly to the site. Urine protein, albumin, β-2 microglobulin, and creatinine concentrations should be measured on all spot urine samples, with calculation of uPCR, uACR, and uBCR values.

Triplicate FMU collections should be before each clinic visit (ie, approximately every 4 weeks), with an additional triplicate to be collected at week 25. At each scheduled clinic visit, the site will dispense the number of spot urine containers needed before the next scheduled visit (dispense 3 containers at each visit except week 24, when 6 containers should be dispensed); participants will return the triplicate spot urine collections at their next visit.

## 10.4. Random Spot Urine Collection

At each clinic visit, participants will be asked to submit a urine sample. As this sample will not necessarily reflect a specific time of day, it is considered a "random" spot urine sample, so as to distinguish it from the first-morning spot collections. After collection, the urine samples must be stored at 2 to 8 °C (ie, in a refrigerator). Urine protein, albumin, β-2 microglobulin, and creatinine concentrations should be measured on all spot urine samples, with calculation of uPCR, uACR, and uBCR values.

## 10.5. Renal Biopsy

The renal biopsy procedure should be performed according to the local standard practices at the site, including use of anesthesia/sedation, pre-procedure evaluation (eg, laboratory testing prior to renal biopsy), and post-procedure observation and monitoring. The renal biopsy must include adequate material for light microscopy (7 or more glomeruli), immunofluorescence microscopy (3 or more glomeruli) and electron microscopy (2 or more glomeruli). Renal biopsy samples should be handled and processed as per the pathology manual. All renal pathology will be reviewed by the central pathology laboratory, which will be blinded to treatment assignment.

If a coagulation panel is performed for any reason, including as a prebiopsy assessment, the use of silica reagents for this test should be avoided in participants participating in this study because of the potential for interference with pegcetacoplan.

### 10.5.1. Renal Biopsies in Adults

For adult participants, a percutaneous biopsy will be performed at the visits indicated in the schedule of activities (Table 2).

A renal biopsy will be performed during screening to confirm diagnosis and eligibility, and to serve as the baseline renal biopsy. Once participant eligibility has been confirmed on the basis of the assessments from screening visit 1 and the initial 24-hour urine collection is complete as described in Section 10.2, the baseline renal biopsy may be conducted at any time during screening. A prior renal biopsy done within 28 weeks of randomization may be used for eligibility confirmation and serve as the baseline biopsy, if confirmed as adequate per central pathology review. Copies of prior renal biopsy reports will be requested for review by the central pathology laboratory. If a prior renal biopsy is used to establish eligibility and/or will serve as the baseline biopsy, images and/or slides from that biopsy will also be required. Tissue blocks and/or unstained slides may also be requested for staining at the central pathology laboratory.

Follow-up renal biopsies will be performed at week 26 and week 52. The week 26 biopsy is required for participants aged 18 years and older. The week 26 renal biopsy need not occur on the same day as all other assessments for that visit. However, the week 26 biopsy must occur after the week 26 triplicate FMU collections and before the first visit of the open-label period at week 28. The week 52 biopsy is optional. If performed it should be after collection of the week 48 24-hour urine and not more than 8 weeks after the week 52 visit.

In the event that a participant has a renal biopsy as part of their clinical management, biopsy samples should be reviewed by the central pathology laboratory and the results should be entered into the study database, if possible. If this biopsy includes the required components for this study and is collected within the window defined above, it may serve as the week 52 biopsy.

### 10.5.2. Renal Biopsies in Adolescents

Participants younger than 18 years will be requested to provide renal biopsies in countries and at sites where permitted but are not required to do so, and refusal of a participant younger than 18 years to provide any or all renal biopsies will not be considered noncompliance. Renal biopsies will not be collected for research purposes from adolescent participants in the US or from adolescent participants at sites where not permitted by national or local regulations.

An adequate prior renal biopsy will be used to establish the diagnosis and eligibility for adolescent participants who do not provide a baseline renal biopsy during screening, even if the prior biopsy was performed more than 28 weeks prior to randomization. Copies of prior renal biopsy reports will be requested for review by the central pathology laboratory. If a prior renal biopsy is used to establish eligibility and/or will serve as the baseline biopsy, images and/or slides from that biopsy will also be required. If the historical biopsy was performed more than 28 weeks prior to randomization, then eligibility will be determined on the basis of alternative evidence of active renal disease as described in Section 7.1.

Participants younger than 18 years may advance to the open-label period upon completion of all week 24 though week 26 assessments other than the renal biopsy. Participants younger than 18 years who did not have a baseline biopsy within 28 weeks of day 1 will not have week 26 or week 52 biopsies under any circumstances. Participants under the age of 18 who had a baseline biopsy within 28 weeks of day 1 will be requested to provide week 26 and week 52 renal biopsies, in countries and at sites where allowed. When collected, renal biopsies in adolescent participants should be collected within the windows defined in Section 10.5.1 for biopsies in adult participants.

In the event that a participant younger than 18 years has a renal biopsy as part of their clinical management, biopsy samples should be reviewed by the central pathology laboratory and the results should be entered into the study database, if possible. If this biopsy includes the required components for this study and is collected within the windows defined in Section 10.5.1 for biopsies in adult participants, it may serve as one of the study biopsies.

## 10.6. Ophthalmologic Evaluation

Ophthalmologic evaluation is an optional assessment and will be performed at selected sites. If performed, participants should have a baseline ophthalmologic evaluation, including a basic ophthalmologic examination, spectral domain optical coherence tomography (SD-OCT), and color fundus photography, at an approved ophthalmologic clinical site at any time during the screening period. For those participants with drusen prior to pegcetacoplan administration, a followup- ophthalmologic evaluation, including a basic ophthalmologic examination, SD-OCT, and color fundus photography, is requested to occur at a convenient time between week 42 and week 52 of the study. The SD-OCT and color fundus photography will be acquired using a standardized protocol, performed by reading center certified imagers on registered equipment, and will be transmitted to a central reading center for evaluation. The basic ophthalmologic examination should include best-corrected visual acuity, intraocular pressure, and a dilated fundus examination with comment on presence or absence of pigment, drusen, hemorrhage, atrophy, or other evidence of macular degeneration.

## 10.7. Measured GFR

Measured GFR is an optional assessment that may be done by a site that routinely performs this assessment. In this case, the site should follow its standard procedure for measured GFR (and document the procedure within the participant's source document) once prior to the first pegcetacoplan dose (but no more than 10 weeks prior to the first dose), again at week 26, and a third time between week 42 and week 52, inclusive. In the event that retrospective measured GFR data are available, these should be collected for the same time frame as retrospective data for calculation of eGFR (see Section 10.1).

## 10.8. Health-Related Quality of Life Assessments

Health-related quality of life (HRQoL) assessments will be completed during clinic visits as outlined in the schedule of activities (Table 2).

### 10.8.1. Functional Assessment of Illness Therapy–Fatigue Scale

The FACIT-Fatigue scale is a 13 item Likert scaled instrument that is self-administered by participants. Participants are presented with 13 statements and asked to indicate their responses as it applies to the past 7 days. The 5 possible responses are "Not at all" (0), "A little bit" (1), "Somewhat" (2), "Quite a bit" (3) and "Very much" (4). With 13 statements the total score has a range of 0 to 52. Before calculating the total score, some responses are reversed to ensure that the higher score corresponds to a higher quality of life.

### 10.8.2. 5-Level EuroQol-5 Dimension

The EQ-5D-5L assessment is a standardized measure of health status developed to assess HRQoL by the EuroQoL Group. The questionnaire consists of a descriptive system and a visual analog scale. The descriptive system contains 5 dimensions (mobility, self-care, usual activities, pain/discomfort, and anxiety/depression) with 5 levels in each dimension.

### 10.8.3. Kidney Disease Quality of Life

The KDQOL survey is a kidney disease-specific measure of HRQoL developed by the RAND Corporation, which is self-administered by participants. It includes a generic chronic disease core, and additional items relevant to patients with kidney disease, such as symptoms, burden of illness, social interaction, staff encouragement, and patient satisfaction. Scores are reported separately for each of 5 subscales.

### 10.8.4. Patient Global Impression of Change

The Patient Global Impression of Change is a 1-item questionnaire that measures the participant's impression of the change in their health and assesses whether there has been an improvement or decline in clinical status since the start of the study.

### 10.8.5. Work Productivity and Activity Impairment Questionnaire: Specific Health Problem

The Work Productivity and Activity Impairment Questionnaire: Specific Health Problem (WPAI:SHP) is a patient-reported quantitative assessment of the amount of absenteeism, presenteeism, and daily activity impairment attributable to a specific health problem for working patients. It includes 6 questions assessing health-related work productivity loss over the previous 7 days.

# 11. ASSESSMENT OF SAFETY

## 11.1. Safety Parameters

### 11.1.1. Demographic/Medical History

Medical history will be recorded at screening. Investigators should document the occurrence, signs, and symptoms of the participant's preexisting conditions, including all prior significant illnesses, up to and including 1 year before screening. Additional preexisting conditions, present at the time when informed consent is given, up to the time of first dosing, are to be regarded as concomitant. Medical history will include alcohol consumption and smoking history, if applicable.

A detailed medical history with regard to disease indication (C3G or IC-MPGN) will be requested, and will include prior surgeries, renal biopsies, hospitalizations, dialysis, transplantation, laboratory data, and extrarenal manifestations of the disease. Available disease medical history for the 3 years preceding screening will be requested. Submission of additional historical data is encouraged, when available. Copies of prior renal biopsy reports will be requested for review by the central pathology laboratory. If a prior renal biopsy is used for eligibility and/or will serve as the baseline biopsy, images and/or slides from that biopsy will also be required. Tissue blocks or unstained slides may also be requested for staining at the central pathology laboratory.

Illnesses first occurring or detected during the study and/or worsening of a concomitant illness during the study are to be documented as AEs in accordance with Section 11.2.2.

Additionally, demographic data will be collected for all participants, as allowed per applicable regulations.

### 11.1.2. Vital Signs

Vital signs (body temperature, respiration rate, heart rate, and systolic and diastolic blood pressure measurements) will be evaluated at the visits indicated in the schedule of activities (Table 2). All vital signs will be measured after the participant has been resting in a sitting position for at least 5 minutes, except when they are supine or semireclined because of study procedures and/or AEs, or if deemed necessary by the investigator. Blood pressure measurements are to be taken in the same arm for the duration of the study.

Vital signs measurements will be repeated, if clinically significant or if machine/equipment errors occur. Out-of-range blood pressure, respiratory rate, or heart rate measurements will be repeated, at the investigator's discretion. Any confirmed, clinically significant vital signs measurements must be recorded as AEs.

When pegcetacoplan is administered at the study site, vital signs will be measured within 2 hours prior to infusion. Following the infusions on day 1 and on the first day of open-label dosing (week 26 or week 28, depending on whether a week 26 renal biopsy is required and when it is performed), vital signs will also be measured approximately 30 minutes to 1 hour after dosing.

### 11.1.3. Weight and Height

Body weight will be recorded at each visit. Height will be recorded at screening for adults and every 12 weeks throughout the study for adolescents. Height will be measured using a calibrated stadiometer. Both body weight and height will be assessed without shoes on.

### 11.1.4. Physical Examination

All full physical examinations, performed by the investigator or designee, will include, at a minimum, assessment of the following: general, head, ears, eyes, nose, throat, dentition, thyroid (endocrine), heart, chest, lungs, abdomen, skin, extremities, back/neck, musculoskeletal, and lymph nodes.

Brief physical examinations will include general appearance, heart, lungs, abdomen, extremities, and are to be performed at all visits where a full physical examination does not occur.

Edema will be assessed at each visit.

Additional symptom-driven physical examinations may be performed at any time, as deemed necessary by the investigator.

See the schedule of activities (Table 2) for the details regarding which type of physical examination should be given at each visit.

### 11.1.5. Menses

For WOCBP, the start and stop date of each menstrual period will be recorded in the participant's source document for the duration of the study.

### 11.1.6. Electrocardiograms

All 12-lead ECGs will be measured once, prior to dosing, at the visits outlined in the schedule of activities (Table 2). The ECG will be taken following resting in the supine position for 10 minutes in a quiet environment and prior to any blood sampling procedures.

The ECGs will be classified as normal, having a not clinically significant abnormality, or having a clinically significant abnormality. In addition, ECG parameters of ventricular rate, PR interval, QRS duration, and QT internal (uncorrected and corrected using Fridericia's method [QTcF]) will be reviewed for eligibility and ongoing safety.

### 11.1.7. Chest Radiography

Chest radiography will be performed prior to dosing at the visits outlined in the schedule of activities (Table 2). Posterior-anterior and lateral views of the chest will be recorded via chest radiography; both views are required. The actual chest radiography technical procedure should be performed according to the site's standard protocol. The radiographs will be classified as normal, having a not clinically significant abnormality, or having a clinically significant abnormality per radiology report, and this classification should be included in the source documentation.

### 11.1.8. Infusion Site/Pump Safety Assessment

On the days of clinic visits, pump use safety will be reviewed after study drug administration. This technique evaluation will be performed by a physician or other licensed health care provider (eg, study nurse), as delegated by the investigator. The participant will be asked about any issues related to pump use.

Participants will be instructed to notify the investigator or other study personnel if an infusion site reaction occurs after self-administration or caregiver administration of study drug. All clinically significant findings related to infusion procedures will be recorded as AEs.

### 11.1.9. Laboratory Assessments

Laboratory assessment samples (Table 4) are to be obtained at designated visits as detailed in the schedule of activities (Table 2).

**Table 4: Laboratory Assessments**

| Hematology | Serum chemistry | Urine studies |
|---|---|---|
| Hb<br>Hematocrit<br>Platelet count<br>RBC count<br>Reticulocytes<br>WBC count with differential | Albumin<br>ALT<br>ALP<br>AST<br>Bicarbonate<br>Bilirubin (total, direct, and indirect)<br>BUN<br>C3<br>C4<br>Calcium<br>Chloride<br>Creatinine<br>Creatine kinase<br>Cystatin C<br>Estimated GFR (using CKD-EPI formula for adults; Bedside Schwartz for adolescents)<br>GGT<br>Glucose<br>HDL<br>LDH<br>LDL<br>Phosphorus<br>Potassium<br>Sodium<br>Triglycerides<br>Total cholesterol<br>Total protein<br>Uric acid | Urinalysis<br>• Blood<br>• Bilirubin<br>• Glucose<br>• Ketones<br>• Leukocyte esterase<br>• Nitrite<br>• pH<br>• Pregnancy, when applicable<br>• Protein<br>• Specific gravity<br>• Urobilinogen<br>• Microscopic examination of urine sediment, including for presence of RBCs, WBCs, and casts, will be performed on all urinalyses<br><br>Spot Urines (First-Morning and Random)<br>• ACR<br>• BCR<br>• PCR<br>• Total albumin concentration<br>• Total β-2 microglobulin concentration<br>• Total creatinine concentration<br>• Total protein concentration<br><br>24-Hour Urine<br>• ACR<br>• BCR<br>• PCR<br>• Total albumin concentration<br>• Total β-2 microglobulin concentration<br>• Total creatinine concentration<br>• Total protein concentration<br>• Total volume |

**Table 4: Laboratory Assessments**

| Hematology | Serum chemistry | Urine studies |
|---|---|---|
| **Coagulation**[a] | **Additional** | |
| PT<br>aPTT<br>INR | FSH (postmenopausal women)[b]<br>Hepatitis B panel[b,c]<br>Hepatitis C panel[b,d]<br>HIV antibodies[b]<br>SPEP[2]<br>ANA (with reflex anti-dsDNA)[b]<br>ANCA (with reflex anti-MPO and anti-PR3)[b] | Serum pregnancy test[b]<br>Complement tests (serum/plasma)[e]<br>Anti-drug antibodies<br>Vaccination response[f] |

Abbreviations: ACR = albumin-to-creatinine ratio; ALP = alkaline phosphatase; ALT = alanine aminotransferase; ANA = antinuclear antibodies; ANCA = antineutrophil cytoplasmic antibodies; aPTT = activated partial thromboplastin time; AST = aspartate aminotransferase; BCR = β-2 microglobulin:creatinine ratio; BUN = blood urea nitrogen; CKD-EPI = Chronic Kidney Disease–Epidemiology Collaboration; ds DNA = double stranded DNA; FSH = follicle-stimulating hormone; GFR = glomerular filtration rate; GGT = γ-glutamyltransferase; Hb = hemoglobin; HBcAb = hepatitis B core antibody; HbsAg = hepatitis B surface antigen; HCV = hepatitis C virus; HDL = high-density lipoprotein; Hib = *Haemophilus influenzae* type B vaccine; HIV = human immunodeficiency virus; INR = international normalized ratio; LDH = lactate dehydrogenase; LDL = low-density lipoprotein; MPO = myeloperoxidase; PCR = protein-to-creatinine ratio; PCV13 = pneumococcal conjugate vaccine; PPSV23 = pneumococcal polysaccharide vaccine 23; PR3 = proteinase 3; PT = prothrombin time; RBC = red blood cell; SPEP = serum protein electrophoresis; WBC = white blood cell.

a. The use of silica reagents in coagulation panels should be avoided in participants participating in this study because of the potential for interference with pegcetacoplan.
b. Screening only. SPEP will be evaluated only in adult participants.
c. HbsAg and HbcAb.
d. Hepatitis C antibody with reflex HCV RNA polymerase chain reaction.
e. Complement tests (serum/plasma): AH50, CH50, C3a, C3b/iC3b, C5a, sC5b-9, C3NeF, and potentially additional complement components. C3NeF will only be assayed in samples collected at the baseline (screening) visits.
f. Serum samples will be collected to evaluate response to vaccinations to *N. meningitidis* (types A, C, W, Y, and B), PCV13 or PPSV23, and Hib. Samples will only be analyzed in the event that the participant has a positive infection for *Streptococcus pneumoniae*, *Neisseria meningitidis*, or *Haemophilus influenzae*.

Blood and urine samples will be analyzed at a central or local laboratory facility, as defined in the laboratory manual. Urine samples will be analyzed by dipstick and a microscopic analysis. All laboratory reports must be reviewed, signed, and dated by the investigator. A legible copy of all reports must be filed with the participant's medical record (source document) for that visit. Any laboratory test result considered by the investigator to be clinically significant should be considered an AE (clinically significant AEs include those that require an intervention). Clinically significant abnormal values occurring during the study will be followed up until repeat test results return to normal, stabilize, or are no longer clinically significant.

#### 11.1.9.1. Pregnancy Screen

For WOCBP, a serum pregnancy test will be performed at screening. Participants with a positive result will be excluded or discontinued from the study as described in Section 7.3. A urine pregnancy test will also be performed starting at baseline and then at all visits through the end of the study, including the follow-up visits. Male participants will be counseled to avoid donating semen during the time between the first screening and the final week 60 exit visit and for the 90 days after their last dose of study drug.

### 11.1.10. Pharmacokinetic Assessment

Blood samples for PK assessment of pegcetacoplan will be collected before dosing via direct venipuncture at the visits delineated in the schedule of activities (Table 2).

Instructions for collection, handling, processing, storage, and shipping of samples will be provided in a separate laboratory reference manual prior to study initiation.

The concentration of study drug will be determined from the serum samples using a validated analytical method. Details of the method validation and sample analysis will be included in the final clinical study report.

### 11.1.11. Pharmacodynamic Assessment

Blood samples for assessment of the effects of pegcetacoplan on the complement profile (CH50 and AH50, sC5b-9, and potentially additional complement components) will be collected before dosing via direct venipuncture at the visits delineated in the schedule of activities (Table 2).

Instructions for collection, handling, processing, storage, and shipping of samples will be provided in a separate laboratory reference manual prior to study initiation.

PD assessment results will be included in the final clinical study report.

### 11.1.12. Blood Volume for Study Assessments

Table 5 presents the approximate blood volume to be collected for this study. See the laboratory manual for additional details regarding the blood volume required throughout the study.
The maximum volume of blood drawn at any single visit is 24.7 mL for adult participants and 22.2 mL for adolescent participants and occurs at the first screening visit. For the remainder of the study, the maximum volume of blood drawn at any single visit is 18.2 mL for both adult and adolescent participants. For an adolescent participant at the minimum permitted body weight of 30 kg, the 22.2-mL and 18.2-mL volumes would be 0.74 and 0.61 mL of blood per kg of body weight, respectively. Over a 4-week period, the maximum blood volume to be drawn is approximately 55 mL for adult participants and 52.5 mL for adolescent participants. This would occur only if the first screening visit was 4 weeks before the start of the RCP. For an adolescent participant at the minimum permitted body weight of 30 kg, this would be a maximum of 1.75 mL/kg in a 4-week period.

**Table 5: Approximate Blood Volume Collected**

| Sample type | Approximate volume per visit | Collections during screening | Collections during main period | Collections during follow-up | Approximate volume over course of study |
|---|---|---|---|---|---|
| Serum chemistry (with serum complement profile) | 5 mL | 2 | 12 | 3 | 85 mL |
| Serum chemistry (without serum complement profile) | 3.5 mL | 0 | 2 | 0 | 7 mL |
| Plasma complement profile | 1 mL | 1 | 9 | 2 | 12 mL |
| Hematology | 1 mL | 2 | 14 | 3 | 19 mL |
| Serum coagulation | 2.7 mL | 2 | 14 | 3 | 51.3 mL |
| Additional screening tests | 15 mL[a] | 1 | 0 | 0 | 15 mL[a] |
| PK samples | 2.5 mL | 0 | 8 | 1 | 22.5 mL |
| PK and immunogenicity (antidrug antibodies) combined | 8.5 mL | 0 | 6 | 2 | 68 mL |
| Vaccination response samples | 3.5 mL | 1 | 1 | 0 | 7 mL |
| **Approximate blood volume per visit** | | **12-25 mL** | **10-18 mL** | **11-18 mL** | |
| **Total approximate blood volume for study** | | | | | **287 mL** |

Abbreviation: PK = pharmacokinetic.
[a] 12.5 mL for adolescent participants.

### 11.1.13. Antidrug Antibody Assessment

Blood samples for assessment of antidrug antibodies (ADAs) will be collected at the visits indicated in the schedule of activities (Table 2). Samples will be assayed for both antibodies against polyethylene glycol (PEG) (anti-PEG antibodies) and antibodies against the peptide moiety of pegcetacoplan (anti–pegcetacoplan peptide antibodies).

The proposed ADA sampling schedule was established to capture the ADA signal at baseline, any potential early onset antibody formation, and the dynamic profile (transient or persistent) of antibody formation while minimizing pegcetacoplan level in the sample.

Participants who have a treatment-emergent or treatment-boosted antidrug antibody response at any time will have ADA samples collected approximately every 6 months until resolution (participants with a treatment-boosted response will be followed until the titer returns to within four-fold of baseline and participants with a treatment-emergent response will be followed until there is a negative sample). Antibody titers will be determined for any samples that are confirmed to be positive for anti–pegcetacoplan peptide or anti-PEG antibodies, and any samples that are confirmed to be positive for anti–pegcetacoplan peptide antibodies will be further characterized with a neutralizing antibody assay.

#### 11.1.14. COVID-19 Assessments

If a participant has been tested for COVID-19, the results, if available, will be documented in the participant's source document.

## 11.2. Adverse and Serious Adverse Events

### 11.2.1. Definitions

#### 11.2.1.1. Adverse Events

An AE is any untoward medical occurrence associated with the use of a drug in humans, whether or not it is considered drug-related. An AE can, therefore, be any unfavorable and unintended sign, including a clinically significant abnormal laboratory finding, symptom, or disease temporally associated with the use of an investigational product, whether or not it is considered related to the investigational product. TEAEs are defined as AEs that develop or worsen after the first dose of study medication.

AEs can be reported spontaneously by the participant and/or in response to an open question from the study personnel or revealed by observation and will be recorded during the study at the investigational site. All identified AEs must be recorded and described in the participant's source document. Fluctuating or nonsignificant changes in laboratory values do not necessarily qualify for AE reporting. If these changes in laboratory values are assessed as clinically significant and/or lead to discontinuation of administration of investigational product, they should be reported as an AE. If these laboratory values are linked to a diagnosis, only the diagnosis should be reported as an AE.

#### 11.2.1.2. Serious Adverse Events

An SAE is any AE or suspected adverse reaction that, in the view of the investigator, results in death, is life-threatening, requires inpatient hospitalization or prolongation of existing hospitalization, results in a persistent or significant incapacity or substantial disruption of the ability to conduct normal life functions, or is a congenital anomaly or birth defect. Important medical events that may not result in death, be life-threatening, or require hospitalization may be considered serious when, based upon appropriate medical judgment, they may jeopardize the participant or may require medical or surgical intervention to prevent one of the outcomes listed in the above definition. Examples of important medical events include allergic bronchospasm requiring intensive treatment in an emergency room or at home, blood dyscrasias or convulsions that do not result in inpatient hospitalization, or the development of drug dependency or drug abuse.

*Life-threatening* is defined as an AE or suspected adverse reaction, which, in the view of either the investigator or sponsor, places the participant at immediate risk of death as it occurred. It does not include an AE or suspected adverse reaction that, had it occurred in a more severe form, might have caused death.

All SAEs (irrespective of causality) must be reported to Apellis Safety immediately, no later than 24 hours of the investigator or their representative becoming aware of the event, as described in Section 11.2.3.

#### 11.2.1.3. Unexpected AEs

An AE or suspected adverse reaction is considered "unexpected" if it is not listed in the reference safety information section of the IB that is in effect at the time of event onset.

### 11.2.2. Recording and Reporting AEs

All AEs and SAEs will be collected from the signing of the informed consent form (ICF) until the exit visit.

SAEs will be followed until satisfactory resolution, until baseline level is reached, or until the SAE is considered by the investigator to be chronic or the patient is stable, as appropriate.

All SAEs that are suspected of being related to study treatment must be reported immediately to the sponsor if the investigator becomes aware of them, regardless of the time since the completion of the clinical trial.

Any events that occur prior to the start of dosing will be categorized as pretreatment events; events occurring after the start of dosing and up to 56 days beyond the last dose of study medication will be recorded as TEAEs.

For each AE, the investigator will evaluate and report the onset date (and time, if applicable), resolution date (and time, if applicable), intensity, causality, action taken, seriousness criteria met (if applicable), and whether or not the participant discontinued the study as a result of the event. AEs characterized as intermittent require documentation of the start and stop of each incidence. If possible, the outcome of any AE resulting in permanent discontinuation or that was present at the end of the study should be reported. AEs resulting in interruption or discontinuation of study drug or that are present at the exit visit should receive follow-up as appropriate.

#### 11.2.2.1. Relationship to Study Drug

The investigator will review each event and assess its relationship to study drug treatment (not related, unlikely related, possibly related, or definitely related). The date and time of onset, time relationship to drug dosing, duration, severity, action taken, and outcome (recovered/resolved, recovered/resolved with sequelae, recovering/resolving, not recovered/not resolved, fatal, or unknown) of each event will be noted.

Table 6 should be considered when evaluating the relationship of AEs/SAEs to study treatment.

**Table 6: Definitions of Adverse Event Relatedness**

| Classification | Definition |
|---|---|
| Definitely related | Strong evidence of a causal relationship; the influence of other factors is unlikely |
| Possibly related | Some evidence of a causal relationship, but other factors may have caused or contributed to the event (eg, another illness or concomitant treatment) |
| Unlikely related | A causal relationship is not a reasonable possibility, but it cannot be completely ruled out with the available evidence. |
| Not related | No evidence of a causal relationship |

#### 11.2.2.2. Severity of Events

The investigator will review each event and assess its severity. Note that severity is a measure of intensity whereas seriousness is defined by the criteria in Section 11.2.1.2. An AE can be of severe intensity but not be considered serious.

Table 7 presents the definitions that should be considered when evaluating the severity of AEs and SAEs.

**Table 7: Severity of Events**

| Severity | Definition/description |
|---|---|
| Mild | Event results in mild or transient discomfort, not requiring intervention or treatment; does not limit or interfere with daily activities (eg, insomnia, mild headache) |
| Moderate | Event is sufficiently discomforting so as to limit or interfere with daily activities; may require interventional treatment (eg, febrile illness requiring oral medication) |
| Severe | Event results in significant symptoms that prevents normal daily activities; may require hospitalization or invasive intervention (eg, anemia resulting in blood transfusion) |

### 11.2.3. Reporting AEs

All SAEs must be reported to Apellis Safety by completing the SAE form within 24 hours of the investigator or their representative becoming aware of the event, whether or not the event is deemed treatment related. Completed SAE forms should be emailed to Safety@Apellis.com.

The sponsor has the responsibility to inform concerned health authorities, IRBs, IECs, and investigators about suspected unexpected serious adverse reactions (SUSARs) in line with GCP guidance and applicable regulatory requirements.

If applicable, specific SAEs should be reported to the concerned IRB or IEC in compliance with local requirements.

### 11.2.4. Pregnancy

Although pregnancy is not an AE, all pregnancies and suspected pregnancies (including a positive pregnancy test regardless of the pregnant person's age or disease state) occurring with a female participant or the female partner of a male participant must be followed to conclusion to determine their outcome and are considered immediately reportable events.

Any pregnancy, suspected pregnancy, or positive pregnancy test must be reported to Apellis Safety within 24 hours of the investigator or their representative becoming aware of the event, using the pregnancy report form. The pregnancy report form must be signed and dated by the investigator and submitted via email to Safety@Apellis.com.

The investigator should make every effort to obtain the consent from the pregnant woman to follow the status of the pregnancy until completion. The outcome of the pregnancy (eg, delivery, termination) and, as applicable, the neonatal outcome should be reported within 24 hours of awareness. In the event of an abnormal outcome (outcome other than live, healthy birth), an SAE report form must be reported within 24 hours of investigator awareness, as described in Section 11.2.3.

### 11.2.5. AEs of Special Interest

The following events are classified as AEs of special interest:

- Thrombocytopenia
- Severe infection (see Section 11.2.2.2 for a discussion of severity grading)
- Hypersensitivity, including pneumonitis
- Severe acute kidney injury (see Section 11.2.2.2 for a discussion of severity grading)

An AE of special interest can be serious or nonserious but must be reported to the sponsor/Apellis Safety in the same manner as an SAE, as described in Section 11.2.3.

### 11.2.6. Abuse, Misuse, Overdose, and Medication Errors

Occurrences of events of abuse or misuse of the medicinal product, overdose, and medication error must be reported to Apellis Safety.

**Abuse of a medicinal product:** Persistent or sporadic intentional, excessive use of medicinal products that is accompanied by harmful physical or psychological effects.

**Misuse:** Intentional and inappropriate use of a medicinal product not in accordance with the prescribed or authorized dose, route of administration, and/or intended indication(s) or not within the legal status of its supply.

**Overdose:** Any dose administered to or taken by a participant (accidentally or intentionally) that exceeds the highest daily dose or is at a higher frequency than that described in the protocol.

**Medication Error:** An error made in prescribing, dispensing, administration, and/or use of the study drug. Medication errors are reportable to the sponsor as defined below.

- The dispensing, administration, and/or use of unassigned study drug
- The administration and/or use of an expired study drug

All AEs or SAEs associated with abuse or misuse of the medicinal product, overdose, or medication error must be reported appropriately. The investigator, in consultation with the sponsor, will decide whether a dose is to be considered an overdose. In the event of an overdose, the actual dose administered must be recorded in the participant's source document.

# 12. STATISTICS

A formal statistical analysis plan (SAP) will be developed and finalized prior to locking the database. The full details of data presentations and analyses will be provided in the SAP. Additional statistical analyses other than those described in this section may be performed if deemed appropriate and included in the SAP. Any deviations from the final SAP or from what is outlined in the protocol will be discussed in the final study report.

## 12.1. Determination of Sample Size

Approximately 80 to 100 participants, including patients with native kidney disease or post-transplant, will be randomized 1:1 to pegcetacoplan or placebo with 40 to 50 participants per arm.

Based on preliminary data from Study APL2-201, a reduction of 60% in uPCR in the pegcetacoplan group at week 26 is assumed vs a reduction of 20% in uPCR in the placebo arm, which corresponds to mean log ratio to baseline of −0.92 vs −0.22 respectively, and a standard deviation of 0.88 (on log-scale). Based on this assumption, a sample size of 70 participants in total provides at least 90% power at 1-sided significance level of 0.025. Considering a 10% attrition to account for potential missing assessments and impact by COVID-19, it is expected that at least 78 participants with native kidney disease should be enrolled.

A minimum of 63 participants with C3G in native kidneys will be enrolled, which is approximately 80% of the enrolled participants with native kidney disease.

## 12.2. Analysis Set

### 12.2.1. Screened Set

The screened set will include all participants who provide written informed consent. This set will be used only for the purpose of describing participant disposition.

### 12.2.2. Safety Set

The safety set will include all participants who receive at least 1 dose of study drug in the RCP. Participants will be analyzed according to the treatment they received.

### 12.2.3. Intent-to-Treat Set

The intent-to-treat (ITT) set will include all randomized participants. Participants will be analyzed according to their assigned treatment, regardless of the treatment actually received. All efficacy analyses will be performed with the ITT set.

### 12.2.4. Per-Protocol Set

The per-protocol (PP) set will include all participants in the ITT set who have not violated any inclusion or exclusion criteria and/or deviated from the protocol in a way that could influence their efficacy assessment. Decisions concerning the exclusion of participants from the PP analysis set will be made and documented prior to database lock.

### 12.2.5. Pharmacokinetic Set

The PK set will include all participants in the ITT set who receive pegcetacoplan and have at least 1 evaluable postdose PK measurement.

### 12.2.6. Pharmacodynamic Set

The PD set will include all participants in the ITT set who receive pegcetacoplan and have at least 1 evaluable postdose PD measurement.

### 12.2.7. Data Review for Analysis Set

After all the data have been verified, coded, and entered into the database, a review will be performed to check the quality of the data, identify any outliers, and make decisions on how to deal with any data issues (eg, missing values, withdrawals, protocol deviations). After this review, resolution of all issues, and documentation of all decisions, the database will be locked.

## 12.3. Efficacy Analysis

### 12.3.1. Analysis of Primary Efficacy Endpoint

The targeted treatment regimen to be used for this study is pegcetacoplan or placebo plus stable regimen for C3G/IC-MPGN treatment. The primary estimand on the treatment effect of pegcetacoplan is for the participant population as defined through the study inclusion/exclusion criteria, with the primary endpoint based on the log-transformed ratio of uPCR (sampled from first-morning urine collections) at week 26 compared to baseline using an equal-weighted average over weeks 24, 25, and 26.

Population level summary is the treatment difference in the mean change from baseline in log-transformed ratio to baseline in uPCR of pegcetacoplan over placebo.

A hypothetical strategy will be used for addressing the intercurrent events (ICEs) that are listed in ICE categories 1 and 2 in Table 8. In the hypothetical strategy, uPCR data collected at visits after the occurrence of ICEs will be set as missing and will be imputed using different approaches as described in Table 8. A treatment policy strategy will be used for ICEs of premature discontinuation of treatment (ICE category 3). In the treatment policy strategy, any measured value will be used as is.

The primary endpoint will be analyzed using a mixed effect model for repeated measures (MMRM). The model will include fixed categorical effects for treatment group, visit, disease type (C3G vs IC-MPGN), stratification factors, and the visit-by-treatment group interaction, as well as the continuous, fixed covariate of baseline log-transformed uPCR. Initially, an unstructured covariance matrix will be investigated. If this analysis fails to converge, other covariance structures will be used; details will be provided in the statistical analysis plan. The difference between treatment groups using a composite contrast of equal-weighted average over weeks 24, 25, and 26 will be estimated with its 95% CI and corresponding *P* value. The model will be applied to each multiply imputed data set, and results from each imputed data set will be combined using Rubin's rule (Rubin 1976).

The primary efficacy analysis will be conducted using the ITT set. Supplemental analysis based on alternative estimands, analysis using the PP set, and sensitivity analyses for assessing the robustness of the primary analysis results will be performed and prespecified in the SAP.

Subgroup analyses will also be conducted to describe findings within both stratification factors in addition to other important prognostic factors such as disease type, age, sex, baseline uPCR, etc.

**Table 8: Intercurrent Events During the Randomized Treatment Phase and Strategies Addressing Intercurrent Events for the Primary Efficacy Endpoint**

| ICEs | Strategies for addressing ICEs |
|---|---|
| ICE Category 1<br>Use of prohibited concomitant medication in the protocol (Section 8.3.3)<br>Use of rescue therapies defined in the protocol (Section 8.3.2) | Hypothetical strategy will be used where all measurements after events under any ICE in this category will be set to missing. Missing data resulting from these ICEs will be imputed using reference-based imputation. |
| ICE Category 2<br>Start renal replacement therapy (dialysis) | Hypothetical strategy will be used where all measurements after the ICEs will be set as missing. Missing data resulting from these ICEs will be imputed based on the worst change of all participants across visits plus a random error. |
| ICE Category 3<br>Permanent discontinuation of study treatment. | Treatment policy strategy will be used whereby any measured value will be used as is. Missing data resulting from the ICEs will be imputed using reference-based imputation. |

Abbreviations: ICE = intercurrent event.

### 12.3.2. Analysis of Secondary Efficacy Endpoints

To preserve the overall type I error among the primary endpoint and key and additional secondary endpoints, a fixed-sequence testing strategy will be used; hence, statistical significance of the first secondary endpoint will be evaluated only if statistical significance is achieved with the primary analysis of the primary endpoint. The remaining secondary endpoints will also adhere to this testing strategy, and their order will match the order in which they are presented in Section 5.2.1.

#### 12.3.2.1. Key Secondary Efficacy Endpoints:

- The numbers and proportion of participants who meet the criteria for achieving a composite renal endpoint at week 26 will be tabulated by treatment group and analyzed using a logistic regression model with treatment group as the independent variable and adjusted for baseline eGFR values, baseline log-transformed uPCR values, disease type, and stratification factors. The *P* value and odds ratio of being a responder for the pegcetacoplan group to being a responder for the placebo group with associated 95% CI will be provided.

  A participant meets the requirements of the composite renal endpoint if he/she satisfies: (1) a stable or improved eGFR compared to baseline (≤15% reduction in eGFR), and (2) a ≥50% reduction in uPCR compared to baseline.

  A composite strategy will be used for addressing the ICEs listed in Table 9. In the composite strategy, the composite renal endpoint status at or after the occurrence of any of these ICEs below will be regarded as non-responder.

**Table 9: Intercurrent Events During the Randomized Treatment Phase and Strategies Addressing Intercurrent Events for the Composite Renal Endpoint**

| ICEs | Strategies for addressing ICEs |
|---|---|
| Use of prohibited concomitant medication in the protocol (Section 8.3.3)<br>Start renal replacement therapy (dialysis)<br>Use of rescue therapies defined in the protocol (Section 8.3.2)<br>Permanent discontinuation of study treatment | Composite strategy. The composite renal endpoint status at or after the initiation of any of ICEs will be regarded as non-responder. |

Abbreviations: ICE = intercurrent event.

- The numbers and proportion of participants who achieve uPCR response at week 26 will be tabulated by treatment group and compared using a logistic regression model with treatment group as the independent variable and adjusted for baseline uPCR values, disease type, and stratification factors. The *P* value and odds ratio of being a responder for the pegcetacoplan group to being a responder for the placebo group with associated 95% CI will be provided. ICEs will be handled as described in Table 9.

  The uPCR response, defined as a reduction of at least 50% from baseline in uPCR at week 26, will be calculated using the following steps:

  1. Baseline uPCR value will be calculated as the average of the uPCR measurements from at least 6 of the 9 FMU samples collected between the start of screening and day 1, inclusive. The uPCR values used to calculate baseline should include those from the samples collected on day –2, day –1, and before dosing on day 1.
  2. Week 26 uPCR value will be calculated as the average of the uPCR measurements from at least 6 of the 9 FMU samples collected in week 24, week 25, and week 26.
  3. Each participant will be categorized as either a success or a failure according to whether the change from baseline in uPCR calculated from steps 1 and 2 is a reduction of at least 50%.

  For participants with evaluable renal biopsies, evaluation of the change from baseline to week 26 in the C3G histologic index activity score will use an analysis of covariance (ANCOVA) model with treatment as a fixed effect, adjusted for baseline C3G histologic index activity score, disease type, and stratification factors. Least-square (LS) means will be presented for each treatment group, along with the between-treatment difference, 95% confidence interval, and *P* value. The strategies to handle intercurrent events are the same as those for the primary endpoint. This endpoint will be evaluated based on adult participants only because neither the week 26 renal biopsy nor an on-study baseline renal biopsy are required for adolescent participants. All data, including any data from adolescent participants, will be included in a summary table.

For participants with evaluable renal biopsies, the numbers and proportion of participants showing decreases in C3c staining intensity of at least 2 orders of magnitude from baseline to the week 26 renal biopsy will be tabulated by treatment group and compared using a logistic regression model with treatment group as the independent variable and adjusted for baseline C3c staining, disease type, and stratification factors. The odds ratio of being a responder for the pegcetacoplan group to being a responder for the placebo group and associated 95% CI and *P* value will be provided. ICE will be handled the same as Table 9. This endpoint will be evaluated based on adult participants only because neither the week 26 renal biopsy nor an on-study baseline renal biopsy are required for adolescent participants.

- Change from baseline in eGFR at week 26 will be tabulated by treatment group and analyzed using a MMRM model. The model will include fixed categorical effects for treatment group, visit, disease type, stratification factors, and the visit-by-treatment group interaction, as well as the continuous, fixed covariate of baseline eGFR values. Initially, an unstructured covariance matrix will be investigated, if this analysis fails to converge, other covariance structures will be used, details will be provided in the statistical analysis plan. The difference between treatment groups will be estimated with its 95% CI and corresponding *P* value. The strategies to handle intercurrent events are similar to those for the primary endpoint.

#### 12.3.2.2. Additional Secondary Efficacy Endpoints:

- The proportion of participants achieving proteinuria <1 g/d at week 26 will be analyzed using the same approach as the first key secondary endpoint.
- For participants with serum albumin levels below the LLN at baseline, the number and proportion of participants with normalization of serum albumin at week 26 will be calculated using the following steps:

  1. Baseline serum albumin value will be calculated as the average of up to 2 serum albumin measurements preceding and including day 1. Participants will be included in the denominator of this calculation only if the baseline albumin value is below the LLN.
  2. Week 26 serum albumin values will be calculated as the average of up to 2 serum albumin measurements preceding and including week 26.
  3. Each participant will be categorized as either a success or a failure according to the week 26 serum albumin values as calculated in Step 2. Values greater than or equal to the LLN will be categorized as a success; values less than the LLN will be categorized as a failure.

  For participants with serum albumin levels below the LLN at baseline, the numbers and proportion of participants with normalization of serum albumin at week 26 will be tabulated by treatment group and compared using a logistic regression model with treatment group as an independent variable and adjusted for baseline albumin value, disease type, and stratification factors. The *P* value and odds ratio of showing serum albumin above the LLN at week 26 for the pegcetacoplan group vs. the placebo group and associated 95% CI will be provided. The strategies to handle intercurrent events are the same as those for the first key secondary endpoint.

- For participants with low serum C3 levels at baseline, the number and proportion of participants with normalization of serum C3 levels at week 26 will be calculated using the following steps:
  1. Baseline serum C3 value will be calculated as the average of up to 2 serum C3 measurements preceding and including day 1. Participants will only be included in the denominator of this calculation if the baseline C3 value is below the LLN.
  2. Week 26 serum C3 value will be calculated as the average of up to 2 serum C3 measurements preceding and including week 26.
  3. Each participant will be categorized as either a success or a failure based on the week 26 serum C3 value as calculated in Step 2. Values greater than or equal to the LLN will be categorized as a success; values less than the LLN will be categorized as a failure.

  For participants with low serum C3 levels at baseline, the numbers and proportion of participants with normalization of serum C3 levels at week 26 will be tabulated by treatment group and compared using a logistic regression model adjusting for baseline serum C3 level, disease type, and stratification factors. The odds ratio of showing a serum C3 level above the LLN at week 26 for the pegcetacoplan group vs. the placebo group and associated 95% CI and *P* value will be provided. The strategies to handle intercurrent events are the same as those for the first key secondary endpoint.
- The change from baseline in FACIT-Fatigue score at week 26 will be analyzed using analysis of covariance (ANCOVA) model with treatment as a fixed effect, adjusted for baseline FACIT-Fatigue score, disease type, and stratification factors. LS means will be presented for each treatment group, along with the between-treatment difference and 95% confidence interval. The strategies to handle intercurrent events are the same as those for the primary endpoint.
- The change from baseline in KDQOL score at week 26 will be analyzed using analysis of covariance (ANCOVA) model with treatment as a fixed effect, adjusted for baseline KDQOL score, disease type, and stratification factors. LS means will be presented for each treatment group, along with the between-treatment difference and 95% confidence interval. The strategies to handle intercurrent events are the same as those for the primary endpoint.

### 12.3.3. Analysis of Exploratory Endpoints

Details regarding the analysis of exploratory endpoints will be presented in the SAP.

## 12.4. Safety Analysis

All safety analyses will be summarized for the safety set.

AEs will be coded using the current version of the Medical Dictionary for Regulatory Activities at the time of analysis. TEAEs will be summarized by System Organ Class, Preferred Term, and treatment group for the number of participants and proportion reporting the event. A similar summary will be produced for SAEs, AEs leading to discontinuation of study drug, severe AEs, and AEs related to the investigational product.

The intensity of AEs and the relationship to investigational product will be summarized for each System Organ Class and Preferred Term by treatment group.

Withdrawals due to AEs will be summarized for each body system and Preferred Term by treatment group.

The AE summaries will be presented across all participants. All AEs will be listed by participant along with information regarding onset, duration, relationship to study drug, severity, action taken with study drug, treatment of event, and outcome. In addition, the number and incidence of rejection episodes and graft loss as well as the incidence of death stratified by transplant history will be tabulated.

In participants who are post transplant, the number and incidence of rejection episodes at week 26, will be tabulated by treatment group and compared between treatment groups using Fisher's exact test or a stratified Cochran-Mantel-Haenszel χ-square test as appropriate. The number and incidence of rejection episodes at week 52 will be tabulated.

In participants who are post transplant, the number and incidence of graft loss at week 26 will be tabulated by treatment group, and the results for treatment groups will be compared using Fisher's exact test or a stratified Cochran-Mantel-Haenszel χ-square test as appropriate. The number and incidence of graft loss at week 52 will be tabulated.

Changes from baseline in clinical laboratory test results will be summarized, using descriptive statistics, by visit and nominal time postdose. Baseline will be taken as the measurement closest to but before randomization. Out-of-range values will be flagged in data listings.

ADA results, ECG results, and changes from baseline in vital sign measurements will be summarized by treatment group, using appropriate descriptive statistics. Baseline will be taken as the measurement closest to but before randomization.

Values of potential clinical significance (eg, increase in QTcF ≥30 milliseconds from baseline) will be flagged in listings and summarized by treatment.

ECG parameters will be analyzed using concentration effect models.

Changes in physical examination results will be described in a data listing.

## 12.5. Pharmacokinetic Analysis

The PK concentrations will be evaluated using the PK set.

Concentrations will be summarized, using descriptive statistics, over time, in the randomized treatment group (pegcetacoplan group).

Individual participant concentration-time data will be plotted against actual sampling time. Median profiles of the concentration-time data, using nominal sampling times, will also be presented. Both linear-linear and linear-log plots will be presented.

Population PK and exposure-response modeling of the safety and efficacy data will be described in a pegcetacoplan population PK/PD analysis plan. The methods will be based on the FDA guidances for both exposure-response and population PK (FDA Guidance for Industry: Population Pharmacokinetics; FDA Guidance for Industry: Exposure-Response Relationships).

## 12.6. Pharmacodynamic Analysis

The PD endpoints will be evaluated using the PD set.

Absolute values, changes from baseline, and percentage changes from baseline will be summarized using descriptive statistics, over time by treatment group.

Individual participant time profiles will be plotted against actual sampling time. Median profiles, over time, using nominal sampling time, will also be presented.

The PD endpoints will be compared between treatment groups using mixed effect repeated measures analyses.

## 12.7. Other Analyses

Demographics, baseline characteristics, concomitant medication, medical history, and study medication exposure will be summarized by treatment group.

World Health Organization and Medical Dictionary for Regulatory Activities coding dictionaries will be used for the concomitant medications and medical histories, respectively.

## 12.8. Interim Analysis

An analysis will be conducted for administrative purposes or regulatory submissions when all participants have completed the randomized controlled period and the data is cleaned. No type I error adjustment is necessary.

## 12.9. Data Monitoring Committee

A formal independent data monitoring committee (DMC) will be used for this study. Representatives of Apellis Pharmaceuticals, Inc, will not participate in the review of unblinded data, but if requested by the DMC may participate in the review of blinded data or attend portions of DMC meetings at which blinded data are presented and discussed to facilitate or answer questions. In addition, external medical and scientific experts may be invited to participate in the reviews, as needed.

DMC meetings will be held according to the schedule in the DMC charter. The remit, roles, and responsibilities of the DMC will be specified in a DMC charter.

# 13. DIRECT ACCESS TO SOURCE DATA/DOCUMENTS

## 13.1. Study Monitoring

Before an investigational site can enter a participant into the study, a representative of Apellis Pharmaceuticals, Inc, will visit the investigational study site to:

- Determine the adequacy of the facilities
- Discuss with the investigator(s) and other personnel their responsibilities with regard to protocol adherence, and the responsibilities of Apellis Pharmaceuticals, Inc, or its representatives. This will be documented in a clinical study agreement between Apellis Pharmaceuticals, Inc, and the investigator.

During the study, a monitor from Apellis Pharmaceuticals, Inc, or representative will have regular contacts with the investigational site, for but not limited to the following:

- Provide information and support to the investigator(s)
- Confirm that facilities remain acceptable
- Confirm that the investigational team is adhering to the protocol, that data are being accurately recorded in the case report forms, and that study drug accountability checks are being performed
- Perform source data verification. This includes a comparison of the data in the case report forms with the participant's medical records at the hospital or practice, and other records relevant to the study. This will require direct access to all original records for each participant (eg, clinic charts).
- Record and report any protocol deviations not previously sent to Apellis Pharmaceuticals, Inc
- Confirm AEs and SAEs have been properly documented on CRFs and confirm any SAEs have been forwarded to Apellis Pharmaceuticals, Inc, and those SAEs that met criteria for reporting have been forwarded to the IRB

The monitor will be available between visits if the investigator(s) or other staff needs information or advice.

## 13.2. Audits and Inspections

Authorized representatives of Apellis Pharmaceuticals, Inc, a regulatory authority, an Independent Ethics Committee, or an IRB may visit the site to perform audits or inspections, including source data verification. The purpose of an Apellis Pharmaceuticals, Inc, audit or inspection is to systematically and independently examine all study-related activities and documents to determine whether these activities were conducted, and data were recorded, analyzed, and accurately reported according to the protocol, GCP guidelines of the International Council for Harmonisation of Technical Requirements for Pharmaceuticals for Human Use (ICH), and any applicable regulatory requirements. The investigator should contact Apellis Pharmaceuticals, Inc, immediately if contacted by a regulatory agency about an inspection.

## 13.3. Institutional Review Board

The principal investigator must obtain IRB approval for the investigation. Initial IRB approval and all materials approved by the IRB for this study, including the informed consent and assent forms and any recruitment materials, must be maintained by the investigator and made available for inspection.

# 14. QUALITY CONTROL AND QUALITY ASSURANCE

To ensure compliance with GCP and all applicable regulatory requirements, Apellis Pharmaceuticals, Inc, may conduct a quality assurance audit. Please see Section 13.2 for more details regarding the audit process.

# 15. ETHICS

## 15.1. Ethics Review

The final study protocol, including the final version of the ICF, must be approved or given a favorable opinion in writing by an IRB or IEC as appropriate. The investigator must submit written approval to Apellis Pharmaceuticals, Inc, before he or she can enroll any participant into the study.

The principal investigator is responsible for informing the IRB or IEC of any amendment to the protocol in accordance with local requirements. In addition, the IRB or IEC must approve all advertising used to recruit participants for the study. The protocol must be reapproved by the IRB or IEC upon receipt of amendments and annually, as local regulations require.

The principal investigator is also responsible for providing the IRB with reports of any reportable serious adverse drug reactions from any other study conducted with the investigational product. Apellis Pharmaceuticals, Inc, will provide this information to the principal investigator.

Progress reports and notifications of serious adverse drug reactions will be provided to the IRB or IEC according to local regulations and guidelines.

## 15.2. Ethical Conduct of the Study

The study will be performed in accordance with ethical principles that have their origin in the Declaration of Helsinki and are consistent with the GCP guideline of the ICH, applicable regulatory requirements, and the Apellis Pharmaceuticals, Inc, policy on bioethics.

## 15.3. Written Informed Consent

The principal investigator(s) at each center will ensure that written informed consent is obtained for each participant before any study procedures are conducted. The informed consent process must at a minimum include the following:

- The investigator or his/her representative will explain the nature of the study to the participant and/or his or her legally authorized representative, including full and adequate oral and written information about the nature, purpose, and possible risks and benefits of the study, and answer all questions regarding the study. Participants and/or their legally authorized representatives or guardians should have the opportunity to consider the information provided before providing their consent.
- Participants must be informed that their participation is voluntary and that they are free to discontinue from the study at any time. Each participant or their legally authorized representative will be required to sign a statement of informed consent that meets the requirements of Chapter 50 of Title 21 of the US Code of Federal Regulations, local regulations, ICH guidelines, Health Insurance Portability and Accountability Act requirements, where applicable, and the requirements of the IRB/IEC or study center. Where appropriate, the participant must also give assent to participation in the study in accordance with local regulations.

- The medical record must include a statement that written informed consent was obtained before the participant was enrolled in the study and the date the written consent was obtained. The authorized person obtaining the informed consent must also sign the ICF.
- If the ICF is revised, participants must be reconsented to the most current version of the ICF(s) during their participation in the study.
- If informed consent is initially provided by a participant's legally authorized representative and the participant acquires the ability to provide informed consent during their participation in the study (eg, by reaching the age of majority), informed consent must be obtained from the participant before additional study-related activities are conducted.

The principal investigator(s) must maintain the original, signed ICF. A copy of the ICF must be given to the participant.

# 16. DATA HANDLING AND RECORDKEEPING

## 16.1. Inspection of Records

Apellis Pharmaceuticals, Inc, will be allowed to conduct site visits to the investigation facilities for the purpose of monitoring any aspect of the study. The investigator agrees to allow the monitor to inspect the drug storage area, study drug stocks, drug accountability records, participant charts and study source documents, and other records relative to study conduct.

## 16.2. Retention of Records

The principal investigator must maintain all documentation relating to the study for a period of 2 years after the last marketing application approval, or if not approved 2 years following the discontinuance of the test article for investigation. If it becomes necessary for Apellis Pharmaceuticals, Inc, or the regulatory authority to review any documentation relating to the study, the investigator must permit access to such records.

# 17. PUBLICATION POLICY

Both the use of data and the publication policy are detailed within the clinical study agreement. Intellectual property rights (and related matters), generated by the investigator and others performing the clinical study, will be participant to the terms of a clinical study agreement that will be agreed between the institution and the sponsor or its designee. With respect to such rights, the sponsor or its designee will solely own all rights and interests in any materials, data, and intellectual property rights developed by investigators and others performing the clinical study described in this protocol, participant to the terms of any such agreement. To facilitate such ownership, investigators will be required to assign all such inventions, either to their institution or directly to the sponsor or its designee, as will be set forth in the clinical study agreement.

# 18. LIST OF REFERENCES

Bomback AS, Santoriello D, Avasare RS, et al. C3 glomerulonephritis and dense deposit disease share a similar disease course in a large United States cohort of patients with C3 glomerulopathy. *Kidney Int*. 2018 Apr;93(4):977-985. doi: 10.1016/j.kint.2017.10.022

Cook HT. Evolving complexity of complement-related diseases: C3 glomerulopathy and atypical haemolytic uremic syndrome. *Curr Opin Nephrol Hypertens*. 2018 May;27(3):165-170. doi: 10.1097/MNH.0000000000000412

Holle J, Berenberg-Goßler L, Wu K, et al. Outcome of membranoproliferative glomerulonephritis and C3-glomerulopathy in children and adolescents. *Pediatr Nephrol*. 2018 Dec;33(12):2289-2298. doi: 10.1007/s00467-018-4034-z

Iatropoulos P, Daina E, Curreri M, et al. Cluster analysis identifies distinct pathogenetic patterns in C3 glomerulopathies/immune complex-mediated membranoproliferative GN. *J Am Soc Nephrol*. 2018 Jan;29(1):283-294. doi: 10.1681/ASN.2017030258

Lu DF, Moon M, Lanning LD, et al. Clinical features and outcomes of 98 children and adults with dense deposit disease. *Pediatr Nephrol*. 2012 May;27(5):773-81. doi: 10.1007/s00467-011-2059-7

Mallinckrodt CH, Lane PW, Schnell D, et al. Recommendations for the primary analysis of continuous endpoints in longitudinal clinical trials. *Drug Inf J*. 2008 Jul;42(4):303-319.

Medjeral-Thomas NR, O'Shaughnessy MM, O'Regan JA, et al. C3 glomerulopathy: clinicopathologic features and predictors of outcome. *Clin J Am Soc Nephrol*. 2014 Jan;9(1):46-53. doi: 10.2215/CJN.04700513

Noris M, Donadelli R, Remuzzi G. Autoimmune abnormalities of the alternative complement pathway in membranoproliferative glomerulonephritis and C3 glomerulopathy. *Pediatr Nephrol*. 2019 Aug;34(8):1311-1323. doi: 10.1007/s00467-018-3989-0

Okpechi IG, Dlamini TA, Duffield M, et al. Outcome of patients with primary immune-complex type mesangiocapillary glomerulonephritis (MCGN) in Cape Town South Africa. *pLoS One*. 2014 Nov 20;9(11):e113302. doi: 10.1371/journal.pone.0113302

Pickering MC, D'Agati VD, Nester CM, et al. C3 glomerulopathy: consensus report. *Kidney Int*. 2013 Dec;84(6):1079-89. doi: 10.1038/ki.2013.377

Regunathan-Shenk R, Avasare RS, Ahn W, et al. Kidney transplantation in C3 glomerulopathy: a case series. *Am J Kidney Dis*. 2019 Mar;73(3):316-323. doi: 10.1053/j.ajkd.2018.09.002

Rubin DB. Inference and missing data. *Biometrika*. 1976 Dec;63(3):581-592. https://doi.org/10.1093/biomet/63.3.581

Servais A, Noël LH, Roumenina LT, et al. Acquired and genetic complement abnormalities play a critical role in dense deposit disease and other C3 glomerulopathies. *Kidney Int*. 2012 Aug;82(4):454-64. doi: 10.1038/ki.2012.63

Smith RJH, Appel GB, Blom AM, et al. C3 glomerulopathy—understanding a rare complement-driven renal disease. *Nat Rev Nephrol*. 2019 Mar;15(3):129-143. doi: 10.1038/s41581-018-0107-2

Zhang Y, Nester CM, Martin B, et al. Defining the complement biomarker profile of C3 glomerulopathy. *Clin J Am Soc Nephrol*. 2014 Nov 7;9(11):1876-1882. doi: 10.2215/CJN.01820214

# 19. APPENDICES

# Appendix 1. Protocol Amendment History

## A.1.1. Protocol Amendment History

| DOCUMENT HISTORY | |
|---|---|
| **Document** | **Date** |
| Country-specific modifications to Protocol Amendment 3 | |
| Amendment 3.1 DEU | 02 November 2023 |
| Amendment 3.1 CHE | 12 September 2023 |
| Amendment 3.1 FRA, NLD | 31 July 2023 |
| Addendum 3.1 ISR | 06 April 2023 |
| Addendum 3.1 JPN | 06 April 2023 |
| Addendum 3.1 CZE | 06 April 2023 |
| Addendum 3.1 CAN | 06 April 2023 |
| Amendment 3 | 03 March 2023 |
| Amendment 2 | 14 August 2021 |
| Amendment 1 | 12 March 2021 |
| Original Protocol | 26 September 2019 |

Country-specific protocol modifications are only listed for the most recent global amendment. Any outstanding country-specific modifications are evaluated at each global amendment and incorporated into the global amendment as appropriate.

## Amendment 3: Summary of Changes From the Previous Version

## Overall Rationale for the Amendment:

The protocol was amended to revise and reorganize the study endpoints in response to feedback and comments from the FDA. Strategies for the statistical analysis of the endpoints were updated to align with the revised endpoints. Additional revisions were made to clarify study procedures and activities in response to feedback from investigators and/or to align with current company practices.

Minor formatting and stylistic revisions were made to align with current templates, and any identified typographical errors were corrected; these are not identified individually in the descriptions below.

| Description of change | Section(s) affected | Rationale for change |
|---|---|---|
| **Study design and rationale changes** | | |
| Updated summary of clinical experience to include Study APL2-308 | Section 4.3 | Updated to reflect current clinical experience with pegcetacoplan |
| Increased duration of screening period from 8 to 10 weeks with a corresponding increase of total duration of study participation to 70 weeks | Synopsis, Section 6, Section 6.1.1, Figure 1 | Updated to allow extra time for the transfer, processing, and reading of biopsy materials within the screening period |
| Increased the minimum number of adolescent participants from 8 to 10 | Section 6, Section 6.2 | Increased to align with the current pediatric investigational plan, and clarified that participants initially screened as adolescents will follow adolescent procedures and requirements through the duration of the study. |
| Revised study scheme diagram | Figure 1 | Reflects revised screening period |
| **Study endpoint changes** | | |
| Changed primary efficacy endpoint from the proportion of subjects with a reduction from baseline in uPCR of at least 50% to the log-transformed ratio of uPCR at week 26 compared to baseline | Synopsis, Section 5.2.1, Section 12.3.1 | Dichotomization of continuous outcomes in clinical trials can result in loss of statistical power. Revised in response to FDA suggestion to assess the primary endpoint as a continuous variable |
| Changed key secondary efficacy endpoint to the proportion of participants who meet the criteria for achieving a composite renal endpoint (a stable or improved eGFR compared to the baseline visit (≤15% reduction in eGFR), and a ≥50% reduction in uPCR compared to the baseline visit.) | Synopsis, Section 5.2.1, Section 12.3.2 | Revised to provide analysis of the population with reduced proteinuria who maintain a stable eGFR. Revised in response to FDA suggestion to redefine the criteria for a stable or improved eGFR; combined eGFR and uPCR responses in a new composite endpoint |
| Escalated change in eGFR to a key secondary endpoint | Synopsis, Section 5.2.1, Section 12.3.2 | Reorganized secondary endpoints following the change to the primary endpoint |
| Added the proportion of participants with a reduction of at least 50% from baseline in uPCR as a key secondary endpoint | Synopsis, Section 5.2.1, Section 12.3.2 | Reorganized secondary endpoints following the change to the primary endpoint |

| Description of change | Section(s) affected | Rationale for change |
|---|---|---|
| Removed change from baseline in uPCR as an additional secondary efficacy endpoint | Synopsis, Section 5.2.1, Section 12.3.2 | Reorganized secondary endpoints following the change to the primary endpoint |
| Revised and reorganized exploratory endpoints to align with changes to primary and secondary endpoints | Section 5.2.1 | Reorganized exploratory endpoints following the change to the primary and secondary endpoints |
| Reorganized pharmacokinetic, pharmacodynamic, and immunogenicity endpoints to appear in a single heading | Section 5.2.3 | Organizational change in presentation of endpoints |
| **Participant selection changes** | | |
| Clarified that biopsy-based inclusion criteria apply to both baseline biopsies collected as part of the study and historical biopsies collected within 28 weeks of randomization and used as baseline biopsies | Synopsis, Section 7.1 | Clarified to provide additional guidance for investigators |
| Added sodium-glucose cotransporter-2 inhibitors to the description of stable treatment regimens for C3G/IC-MPGN | Synopsis, Section 7.1 | Added to reflect recognition of treatment with sodium-glucose cotransporter-2 inhibitors as standard of care treatment for chronic kidney disease |
| Updated the time requirements for stable dosing regimens to remove reference to baseline renal biopsy; timing of stable treatment requirements is now based solely on randomization | Synopsis, Section 7.1 | Clarified to provide additional guidance for investigators and for consistency with stable treatment requirement period |
| Added recent severe infection as an exclusion criterion | Synopsis, Section 7.2 | Incorporated changes requested by health authorities |
| **Study intervention changes** | | |
| Clarified when open-label dosing will begin | Synopsis, Section 6.1.3, Table 2 (Schedule of Activities), Section 11.1.2 | Clarified that open-label dosing will begin at the first visit after the week 26 renal biopsy for adults and any adolescents providing renal biopsies. Open-label dosing for adolescents not providing renal biopsies should start at the week 26 visit. Clarified procedures and requirements for the transition to open-label dosing to provide additional guidance for investigators |
| Updated vaccination guidance to recognize the potential use of local guidelines as an alternative to ACIP guidelines | Section 8.2 | Added to recognize that vaccination requirements and practices may differ between countries |
| Updated discussion of vaccination against *S pneumoniae* to reflect recent changes to ACIP guidelines | Section 8.2 | Revised to reflect current ACIP guidelines |

| Description of change | Section(s) affected | Rationale for change |
|---|---|---|
| **Study assessment changes** | | |
| Removed testing of C3Nef from samples collected after screening | Table 2 (Schedule of Activities), Table 4 (Laboratory Assessments) | Revised protocol to incorporate guidance in a previous protocol clarification letter, as the evidence suggests C3 inhibition does not change C3NF |
| Clarified the timing of the collection of baseline and week 26 renal biopsies and the requirements for using a prior biopsy as the baseline biopsy | Section 6.1.1, Section 10.5 | Clarified the windows for collection of the baseline and 26-week renal biopsies. |
| Clarified requirements for chest radiography, moved baseline radiography to visit 2 | Section 11.1.7, Table 2 (Schedule of Activities) | Clarified requirements for chest radiography to provide additional guidance for investigators. Moved collection of the baseline chest radiography to follow the initial screening visit for consistency with other invasive procedures (eg, vaccination, renal biopsy) |
| **Safety reporting changes** | | |
| Updated discussion of assessment of severity of adverse events | Section 11.2.2.2 | Updated to align with current company practices |
| Updated description of procedures for reporting SAEs to Apellis | Section 11.2.3 | Updated to align with current company practices |
| Updated description of procedures for monitoring and reporting pregnancies to Apellis | Section 11.2.4 | Updated to align with current company practices |
| **Statistical analysis changes** | | |
| Updated discussion of the determination of sample size | Synopsis, Section 6, Section 6.2, Section 12.1 | Updated to provide additional background, and to reflect determination of the sample size based on the revised primary endpoint. Changing the primary endpoint led to an increase in the number of participants with native kidney disease to 78. Also provided enrolment targets for participants with posttransplant recurrence and for participants with C3G in native kidneys. |
| Updated the discussion of the efficacy analyses | Synopsis, Section 12.3 | Updated to reflect the revised the primary, key secondary, and additional secondary endpoints |
| **Study conduct changes** | | |
| Clarified screening procedures, including rescreening activities | Section 6.1.1, Section 6.1.1.1 | Clarified procedures based on feedback from investigational sites |
| Clarified discussion of study treatment discontinuation, study withdrawal, and the activities to be undertaken in these situations | Section 7.3 | Updated to align with current company practices |

| Description of change | Section(s) affected | Rationale for change |
|---|---|---|
| Provided guidance for the resumption of dosing if regularly scheduled doses are missed | Section 8.1 | Updated to provide additional guidance for investigators |
| Clarified discussion of study treatment blinding requirements | Section 8.5.2 | Updated to align with current company practices |
| Added the potential to ship investigational product and/or ancillary study supplies directly to the participant, where permitted | Section 9.3 | Updated to align with current company practices |
| Clarified guidance regarding infusion sites | Section 9.5 | Updated to align with current company practices |
| **Document organization changes** | | |
| Updated Responsible Medical Director | Signature Page | Updated to reflect organizational change in study management |
| Changed wording from “subjects” to “participants” | Global | Updated to align with current company practices |

## Amendment 2: Summary of Changes From the Previous Version

### Overall Rationale for the Amendment:

The protocol was amended to address comments from the FDA. Additional revisions were made for clarification of study activities or to align with current company practices.

Minor formatting and stylistic revisions were made to align with current templates, and any identified typographical errors were corrected; these are not identified individually in the descriptions below.

| Description of change | Section(s) affected by change |
|---|---|
| **Study design and rationale changes** | |
| Changed planned enrollment from 90 to 80-100 subjects | Synopsis, Section 6, Section 6.1.2, Section 6.2, Section 12.1 |
| Clarified vaccination strategy and prophylactic antibiotic use in Risk/Benefit discussion | Section 4.4 |
| Changed primary and key secondary objectives to align with endpoint changes | Synopsis, Section 5.1 |
| Changed from 10 posttransplant minimum to 77 native kidney minimum | Section 6, Section 6.2, Section 8.5.1, Section 12.1 |
| Added 8-adolescent minimum | Section 6, Section 6.2 |
| Revised study scheme diagram | Section 6.1 (Figure 1) |
| **Study endpoint changes** | |
| Changed primary efficacy endpoint to remove eGFR component | Synopsis, Section 5.2, Section 12.3.1 |
| Removed proportion of subjects with uPCR reductions of at least 50% as a secondary efficacy endpoint | Synopsis, Section 5.2, Section 12.3.2 |
| Changed primary and key secondary efficacy endpoints from week 52 to week 26 | Synopsis, Section 5.2, Section 12.3.1, Section 12.3.2 |
| Revised and reorganized secondary and exploratory endpoints (including timing of evaluations) | Synopsis, Section 5.2, Section 12.3.1, Section 12.3.2 |
| **Subject selection changes** | |
| Increased minimum body weight to 30 kg | Synopsis, Section 7.1, Section 8.1 |
| Updated inclusion criteria to define evidence of active renal disease and remove central medical review | Synopsis, Section 7.1 |
| Updated inclusion criteria to define acceptable stable regimens for treatment of C3G/IC-MPGN | Synopsis, Section 7.1 |
| Revised replacement of subjects section to clarify that subjects who discontinue will not be replaced | Section 7.3.4 |
| **Study intervention changes** | |
| Clarified wording of renal biopsies in adolescents section to explicitly note that biopsies will not be collected for research purposes from adolescent subjects in the US | Section 10.5.2 |

| Description of change | Section(s) affected by change |
|---|---|
| Created separate subsection in Concomitant Medications describing permitted prior medications | Section 8.3 |
| Added section discussing possible rescue treatment for C3G/IC-MPGN | Section 8.3.2 |
| **Study assessment changes** | |
| Added specification of use of Bedside Schwartz equation in adolescents where missing | Synopsis, Section 6.1.1 |
| Added chest radiography to schedule of activities and discussion of safety procedures | Table 2, Section 11.1.7, Table A1 |
| Removed fibrinogen and added prothrombin time to coagulation section of laboratory assessments table | Section 11.1.9 (Table 4) |
| **Safety reporting changes** | |
| Added section describing AEs of special interest | Section 11.2.5 |
| **Statistical analysis changes** | |
| Removed discussion of extrapolating placebo response rate from week 26 to week 52 | Synopsis, Section 4.2.2, Section 12.3.1 |
| Removed potential sample size revision | Synopsis, Section 6, Section 6.2, Section 12.8 |
| Changed baseline, week 26, and week 52 uPCR calculations to the average of 6 to 9 samples from the average of 9 samples | Section 6.1.1, Section 12.3.1, Section 12.3.2 |
| Added discussion of primary and alternate estimands | Section 12.3.1 |
| Added discussion of intercurrent events and how they will be handled in assessment of the primary endpoint | Section 12.3.1 |
| Updated descriptions of calculations of responder analysis for uPCR and eGFR endpoints | Section 12.3.1, Section 12.3.2 |
| Revised and clarified descriptions of analysis of secondary endpoints | Section 12.3.2 |
| Removed interim analyses (interim analysis 1 for sample size readjustment and interim analysis 2 for early efficacy assessment) | Synopsis, Section 12.8 |
| **Study conduct changes** | |
| Added section describing maximum blood collection volumes | Section 11.1.12 |
| Removed central medical review for confirmation of diagnosis and eligibility | Synopsis, Section 6.1.1, Section 7.1, Section 10.5.2, Section 11.1.2 |
| Clarified that Apellis representatives will not review unblinded data | Section 12.9 |
| **COVID-19 continuity changes** | |
| Added COVID-19 vaccine to COVID-19 risk mitigation section and COVID-19 Appendix | Section 4.4.1, Appendix 2 |
| **Document organization changes** | |
| Added appendix listing prior revision history of the document | Appendix 1 |

## Amendment 1: Summary of Changes From the Previous Version

### Overall Rationale for the Amendment:

The protocol was amended to revise the study design and endpoints following discussions with regulatory authorities and internal evaluation of the study, and to provide guidance to investigators in the case of COVID-19–related restrictions. Additional revisions were made for clarification of study activities, or to align with current company practices.

Minor formatting and stylistic revisions were made to align with current templates, and any identified typographical errors were corrected; these are not identified individually in the descriptions below.

| Description of change | Section(s) affected by change |
|---|---|
| **Study design and rationale changes** | |
| Updated study design so that the randomized controlled period is double-blinded and added justification of study design section. | Synopsis, Section 4.2.2, Section 6, Section 6.1, Section 8.5.2 |
| Shortened the follow-up period for subjects who do not continue receiving pegcetacoplan from 24 to 8 weeks. | Synopsis, Section 6, Section 6.1, Section 6.1.4 |
| Added requirement that the study will enroll at least 10 subjects with posttransplant recurrence of C3G or IC-MPGN. | Synopsis, Section 6, Section 6.2, Section 8.5.1 |
| Added an interim analysis of efficacy when all subjects have completed the 26-week randomized controlled period. | Synopsis, Section 12.8 |
| Added an optional renal biopsy at week 52 to support evaluation of the revised primary endpoint. | Synopsis, Section 6.1, Section 6.1.3, Table 1 (Schedule of Activities), Section 10.5 |
| **Study endpoint changes** | |
| Revised the primary endpoint from the change from baseline in urine protein-to-creatinine ratio (uPCR) at week 26 to the proportion of subjects with a reduction in uPCR of at least 50% and an estimated glomerular filtration rate (eGFR) that is stable or improved from baseline at week 52. | Synopsis, Section 5.2.1 |
| Extensively revised secondary efficacy endpoints, exploratory endpoints, and safety endpoints. | Synopsis, Section 5.2.2, Section 5.2.3, Section 5.2.4 |
| **Subject selection changes** | |
| Amended inclusion criteria to allow enrollment of adolescents of 20 kg or more, rather than 42 kg or more. | Synopsis, Section 7.1 |
| Amended inclusion criteria to include a definition of women of childbearing potential and provided a definition of childbearing potential. | Synopsis, Section 7.1, Section 7.1.1 |
| Added inclusion criterion that subjects must be willing and able to self-administer pegcetacoplan or have an identified caregiver who can perform the administration. | Synopsis, Section 7.1 |
| Added exclusion criterion requiring an absolute neutrophil count of at least 1000 cells/$mm^3$ at screening. | Synopsis, Section 7.2 |

| Description of change | Section(s) affected by change |
|---|---|
| Added exclusion criterion for subjects with hypersensitivity to pegcetacoplan or excipients. | Synopsis, Section 7.2 |
| Added exclusion criterion for subjects with a history of meningococcal disease. | Synopsis, Section 7.2 |
| Added exclusion criterion excluding subjects with known or suspected hereditary fructose intolerance. | Synopsis, Section 7.2 |
| Removed exclusion criterion for subjects with liver dysfunction. | Synopsis, Section 7.2 |
| Replaced inclusion criterion for body mass index ≤35 kg/$m^2$ with exclusion criterion for weight >100 kg. | Synopsis, Section 7.1, Section 7.2 |
| **Study intervention changes** | |
| Revised dosing recommendations, adding regimens for adolescent subjects ≥50 kg, 35 to <50 kg, and 20 to <35 kg. | Synopsis, Section 4.2.3, Section 8.1 |
| Revised wording of vaccination requirements to cite Advisory Committee on Immunization Practices guidelines and align with current company practices. | Section 8.2 |
| Added description of blinding and unblinding procedures. | Section 8.5.2 |
| **Study assessment changes** | |
| Clarified instructions for triplicate first-morning spot urine collection. | Section 10.3 |
| Provided additional guidance on the collection of renal biopsy samples, including clarification of the biopsy requirements for adolescent subjects. | Section 10.5 |
| Added color fundus photography to the optional ophthalmologic evaluation; clarified that the evaluation should also include a basic ophthalmologic examination. | Table 1 (Schedule of Activities), Section 10.6 |
| **Safety reporting changes** | |
| Update wording describing monitoring and reporting of AEs and SAEs to align with current company practices, including:<br>• Updated wording defining terms<br>• Updated description of evaluation and recording of adverse events<br>• Clarified SAE reporting responsibilities and procedures | <br><br>Section 11.2.1<br>Section 11.2.2<br>Section 11.2.3 |
| • Clarified pregnancy reporting responsibilities and procedures | Section 11.2.4 |
| • Added guidance on the definition and reporting of abuse, misuse, overdose, and medication errors | Section 11.2.5 |

| Description of change | Section(s) affected by change |
|---|---|
| **Statistical analyses changes** | |
| Updated descriptions of statistical analyses to align with revised endpoints. | Synopsis, Section 12.3, Section 12.4 |
| Revised description of sample size evaluation and added a description of the interim analysis of efficacy. | Synopsis, Section 12.1, Section 12.8 |
| **Study conduct changes** | |
| Updated and clarified procedures for obtaining informed consent. | Section 15.3 |
| **COVID-19 continuity changes** | |
| Added wording describing risks related COVID-19 and activities to mitigate these risks. | Section 4.4.1, Appendix 1 |

## Appendix 2. Country-Specific Protocol Modifications

Country-specific modifications to this protocol are listed below.

Additions to the protocol are marked by underlined text, and deletions of existing protocol text are marked by ~~strikethrough text~~.

**Canada**

**Rationale for Modifications**

These modifications to the APL2-C3G-310 protocol are being implemented in Canada to support the Investigational Testing Authorization for the Crono Super PID infusion pump and CRN Crono 20-mL syringe reservoir.

**Summary of Changes**

Changes in the sections noted below will be enacted by this addendum.

## 9.4 Infusion Supplies

The sponsor will provide infusion supplies ~~supply syringes, vial adapters, infusion sets, and ambulatory syringe infusion pumps,~~ as required. Refer to the pharmacy manual for further details.

The Crono Super PID infusion pump and the CRN Crono 20-mL syringe reservoir, which will be used during the clinical study to deliver each dose of the isotonic sterile solution of pegcetacoplan 1080 mg/20 mL (54 mg/mL) transferred from the vial to the syringe, do not have a medical device license authorizing their commercial sale in Canada and are therefore considered investigational medical devices. The infusion pump and reservoir will be used in conjunction with the Neria multi infusion set (MDL #88990; Unomedical Devices SA de CV, Aaholmvej 1-3, Osted, 4320 Lejre, Denmark) and the vial adaptor (MDL #70132; West Pharmaceutical Services IL, Ltd, 4 Hasheizaf St, Ra'Anana, Israel, 4366411), which are approved for use in Canada.

### 9.4.1. Investigational Medical Devices: Crono Super PID Infusion Pump and CRN Crono 20-mL Reservoir

#### 9.4.1.1. Descriptions of the Investigational Medical Devices

The Crono Super PID infusion pump is an electromechanical, software-controlled, reusable infusion pump manufactured by Canè SpA Medical Technology (Via Cuorgnè 42/a, 10098 Rivoli, Cascine Vica [TO], Italy) that is supplied to the participant and used throughout the duration of the study. The CRN Crono 20-mL syringe reservoirs are sterile, nonpyrogenic, single-use syringes specifically for use with the Crono pumps and are supplied in individual sterile packaging. A new syringe reservoir is filled from the drug vial with the transfer set, used for one dose of pegcetacoplan, and then disposed of. During the study, infusion site/pump safety will be assessed during clinical visits, and any significant finding from the assessment will be recorded.

#### 9.4.1.2. Justification for the Use of the Investigational Medical Devices

Apellis assessed the potential hazards associated with the delivery of the drug product in relation to the uses for which the pump carries a CE mark in the EU. The hazards evaluated were related to the required critical performance attributes, the user profile, and the use environment relevant to use with the drug product in the clinical trial. This assessment included testing to confirm performance to the required delivery requirements and the delivery process and evaluation of the user population in comparison with those for which these devices are already CE-marked for use.

The assessment concluded that the Crono Super PID infusion pump could meet the delivery requirements of Study APL2-C3G-310, does not present any unique usability issues to the user groups in Study APL2-C3G-310, and does not introduce any additional or residual risks.

The use of the investigational devices in human participants is justified on the basis of the design and intended uses of the device constituent parts (Canè Crono Super PID Infusion Pump and CRN Crono 20-mL syringe reservoir), which are appropriate.

#### 9.4.1.3. Anticipated Exposure to the Investigational Medical Devices

The anticipated exposure to the investigational device is approximately 30 minutes per infusion twice weekly.

#### 9.4.1.4. Anticipated Adverse Device Effects

The anticipated adverse device effects are discussed in Section 9.4.2.

#### 9.4.1.5. Number of Investigational Devices to be Used

The sponsor plans to open 3 sites in Canada and expects to assign to treatment 2 participants across the sites in Canada. Each participant assigned to treatment will receive a primary pump, a backup pump, and enough infusion sets for 52 weeks of biweekly infusions. Sites will be provided with additional pumps and infusion supplies (approximately 20% overage, rounded up). Actual need will depend on enrollment, but 14 Super PID pumps and approximately 360 syringe reservoirs are planned for the study sites in Canada.

### 9.4.2. Risks of Investigational Medical Devices

The use of this delivery system with this drug in this clinical trial does not create any risks additional to the residual risk inherent with the current use of these devices. There is a possibility that participants could experience redness, swelling, and pain or tenderness at the site of infusion. There is also a slight possibility of infection. In addition, because pegcetacoplan is to be administered by SC infusion, participants may feel some discomfort from the infusion. None of these risks are affected by the use of these specific devices.

### 9.4.3. Reporting of Incidents Related to Investigational Medical Devices

All incidents related to investigational medical devices must be reported to Apellis immediately, within 24 hours of the investigator or their representative becoming aware of the event. The site must complete the complaints form and email it to complaints@apellis.com immediately, within 24 hours of becoming aware of the event.

The sponsor is responsible for submitting reports of incidents related to an investigational medical device to Health Canada and the device manufacturer within 72 hours of becoming aware of the issue. The device manufacturer will then further assess the incident according to the timelines in the guidance and report their findings accordingly.

#### 9.4.4. Investigational Medical Device Accountability

Accountability for the investigational medical devices at the study center is the responsibility of the investigator. The investigator will ensure that the investigational medical devices are used only in accordance with this protocol. Where allowed, the investigator may choose to delegate accountability responsibilities to a pharmacist or other appropriate individual.

Investigators will be provided with sufficient amounts of the investigational medical devices to carry out this protocol for the agreed number of participants. The investigator or designee will acknowledge receipt of the investigational medical devices, documenting shipment content and condition. Accurate records of all the investigational medical devices provided, used, returned, and/or destroyed must be maintained. Accountability records will include dates, quantities, batch/serial numbers, expiration dates (if applicable), and participant numbers. The sponsor or its designee will review the investigational medical device accountability at the study center on an ongoing basis during monitoring visits.

The investigational medical devices must not be used for any purpose other than the present study. The investigational medical devices that have been provided to a participant and returned unused must not be provided to a different participant.

#### 9.4.5. Investigational Medical Device Handling

All used and unused investigational medical devices should be retained at the study center until they are inventoried by the study monitor. All used, unused, or expired investigational medical devices will be returned to the sponsor or its designee for disposition. Disposition will be documented, or if authorized disposed of at the study center according to the center's standard operating procedures and documented.

**Czech Republic**

**Rationale for Modifications**

These modifications to the APL2-C3G-310 protocol are being implemented in the Czech Republic, as requested by the State Institute for Drug Control.

**Summary of Changes**

**Enrollment of Adolescent Participants**

As described in Section 7.1, Inclusion Criteria, adolescents (aged 12-17 years) may be enrolled where approved. The enrollment of adolescent participants is not approved in the Czech Republic, and therefore only adult participants, aged 18 years or older, will be enrolled in this country.

**France, Netherlands, Germany**

**Rationale for Modifications**

These modifications to the APL2-C3G-310 protocol are being implemented to temporarily allow participants in Study APL2-C3G-310 in France, the Netherlands, or Germany to continue to receive pegcetacoplan after the completion of the 52 weeks of treatment defined in Protocol APL2-C3G-310 if they are eligible and intend to enter Study APL2-C3G-314, the long-term extension study, but that study is not yet fully approved and active.

**Summary of Changes**

Changes in the sections noted below will be enacted by this addendum.

**Section 6.1.3, Part 3: Open-Label Period (26 Weeks)**

At the end of the RCP, participants from both groups will proceed to the 26-week open-label period, in which all participants will be treated with pegcetacoplan twice weekly. Open-label dosing will begin at the first visit after the week 26 renal biopsy for adults and any adolescents providing renal biopsies. Open-label dosing for adolescents not providing renal biopsies should start at the week 26 visit. At the end of the open-label period, there will be an optional renal biopsy. Participants younger than 18 years who did not have a baseline biopsy within 28 weeks of day 1 will not have a biopsy at the end of the open-label period. Upon completion of the open-label period, participants will enter the follow-up period (Part 4) unless they enter a long-term extension study.

Participants who have completed the planned 52 weeks study participation and are eligible and intend to enter Study APL2-C3G-314, the long-term extension study, may continue to receive pegcetacoplan in this study if immediate transition to the long-term extension study is not possible because that study is not yet fully approved and active. If this occurs, participants should continue study visits every 6 weeks, and investigators should collect at least the information defined for the week 42 visit in the schedule of activities, excluding any optional assessments. This extension period may continue for up to approximately 3 months.

**Israel**

**Rationale for Modifications**

These modifications to the APL2-C3G-310 protocol are being implemented in Israel to satisfy requirements of the Ministry of Health (MOH) and to clarify that only adolescent study subjects will be enrolled in Israel.

**Summary of Changes**

**Enrollment of Participants**

To comply with the Ministry of Health requirement that in-study renal biopsies must be optional, only adolescent participants will be enrolled at study sites in Israel. Enrolled participants must follow the procedures described in the protocol, including any specific notations or considerations for adolescent participants.

Because only adolescent participants will be enrolled, the following portions of the protocol are modified as indicated:

**Synopsis and Section 7.1, Inclusion Criteria**

1. ~~Aged at least 18 years; where approved, a~~Adolescents (aged 12-17 years) weighing at least 30 kg ~~may also be enrolled.~~

**Section 10.5.1, Renal Biopsies in Adults,** is no longer applicable and should be disregarded.

## Japan

### Rationale for Modifications

These modifications to the APL2-C3G-310 protocol are being implemented in Japan to satisfy requirements of the Pharmaceuticals and Medical Devices Agency and to clarify study procedures in Japan by considering Japanese regulatory rules and the actual situation of general medical practice.

### Contraception Exceptions

The protocol includes implantable or injectable contraceptives and removable birth control devices as acceptable methods of contraception. These methods of contraception are not approved in Japan and therefore will not be allowed for Japanese participants included in this study.

### Vaccinations

Meningitis caused by *Neisseria meningitidis* type B is not endemic to Japan, and the number of patients with meningitis caused by *N meningitidis* type B in Japan has been decreasing in recent years. Given this, the meningococcal B vaccine is not approved in Japan. Participants enrolled in Japan for this clinical trial must have received the meningococcal conjugate vaccine for *N meningitidis* A, C, W, and Y and do not need to have received the meningococcal B vaccine.

The schedule of vaccinations is consistent with the United States Centers for Disease Control and Prevention rationale as well as the Japanese Respiratory Society/Japanese Association for Infectious Diseases recommendation "Considerations of Pneumococcal Vaccination for Adults Aged 65 and Over."

All other vaccinations defined in the protocol are marketed in Japan and should be administered as described in Section 8.2 of the global protocol. Investigators must follow the instructions in the package inserts of these vaccine products when planning vaccination schedules.

### Summary of Changes

Changes in the sections noted below will be enacted by this addendum.

### Contraception Exceptions

Section 7.1.1, Acceptable Methods of Contraception, is modified as below:

### 7.1.1. Acceptable Methods of Contraception

Women of childbearing potential (WOCBP) and male participants with female partners of childbearing potential will be instructed to practice an acceptable method of birth control throughout the duration of the study and for 90 days following the end of study drug administration.

A woman is considered to be of childbearing potential following menarche and until becoming postmenopausal, unless she is permanently sterile. Permanent sterilization methods include hysterectomy, bilateral salpingectomy, and bilateral oophorectomy. A postmenopausal state is defined as no menses for 12 months without an alternative medical cause.

Approved methods of contraception include:

- Combined (estrogen and progestogen containing) hormonal contraception associated with inhibition of ovulation:
  - oral~~, intravaginal, or transdermal~~
- Progestogen-only hormonal contraception associated with inhibition of ovulation:
  - oral~~, injectable, or implantable~~
- ~~Intrauterine device~~
- ~~Intrauterine hormone-releasing system~~
- Bilateral tubal occlusion
- Vasectomized partner (*provided that partner is the sole sexual partner of the WOCBP trial participant and that the vasectomized partner has received medical assessment of the surgical success*)
- Sexual abstinence (*defined as refraining from heterosexual intercourse during the entire period of risk associated with study treatments*)
- Male condom with or without spermicide (for male study participants with female partners of childbearing potential only)

Not all methods of contraception may be available in all of the countries in which this study is being conducted.

Note: Sexual abstinence is only accepted when it is the preferred and usual lifestyle of the participant.

**Vaccinations**

Vaccinations are discussed in multiple locations in the global protocol. The affected sections are modified as below:

**Study Design: Protocol Synopsis and Section 6.1.1**

**Part 1: Screening Period; last bullet**

- Vaccinations against *S pneumoniae*, *N meningitidis* (types A, C, W, and Y~~, and B~~), and *H influenzae* (type B) are mandatory unless documented evidence exists that participants are nonresponders to vaccination. If required, vaccination series should be initiated at least 14 days prior to randomization. Vaccinations are discussed in more detail in Section 8.2.

**Inclusion Criteria: Protocol Synopsis and Section 7.1**

**Inclusion Criterion 8**

8. Have received vaccinations against *S pneumoniae*, *N meningitidis* (types A, C, W, and Y, ~~and B~~), and *H influenzae* (type B) as per ACIP recommendations for adults or children with complement deficiencies. Vaccination series should be initiated at least 14 days prior to randomization. Vaccination is mandatory unless documented evidence exists that participants are nonresponders to vaccination.

**Vaccinations: Section 8.2**

**8.2. Vaccinations**

To receive study drug, participants will be required to be vaccinated as follows on the basis of ACIP recommendations for adults or children with complement deficiencies (available at https://www.cdc.gov/vaccines/schedules/hcp/index.html) or other similar local guidelines.

- *N meningitidis* types A, C, W, and Y: First dose at least 14 days prior to randomization with a second dose 2 months later, and then boosters every 5 years.
- ~~*N meningitidis* type B: First dose at least 14 days prior to randomization with a second dose after at least 1 month. First booster dose 1 year later, and then additional booster doses every 2 to 3 years.~~
- *S pneumoniae*: pneumococcal conjugate vaccine (PCV13, PCV15, or PCV20, depending on participant age and vaccine availability) and/or pneumococcal polysaccharide vaccine ~~23~~ (PPSV23). Adults who have not previously received any pneumococcal vaccine may receive PCV20, if the site is able to procure it, and a subsequent dose of PPSV23 would not be required. Multiple scenarios exist depending on previous vaccination history and investigators should consult ACIP guidelines for adults or children with complement deficiencies or other similar local guidelines.
- *H influenzae* type B: Documentation of childhood vaccination or one dose at least 14 days prior to randomization.

Vaccination is mandatory, unless documented evidence exists that participants have received the recommended vaccinations or are nonresponders to vaccination. For participants who do not have this documented evidence, the required missing vaccination(s) will be administered as needed to bring participants up to date. The investigator will discuss with the medical monitor any individual participant circumstances relevant to the vaccination requirements that would make the above schedule not possible or reasonable, or if the investigator has any questions or concerns about the vaccination requirements. If local or national guidelines for immunizations differ from the ACIP recommendations, the differences should be discussed with the medical monitor to determine an appropriate course of vaccinations. On an ongoing basis, including upon entry into a long-term extension, participants should be reevaluated for the need for any additional vaccinations or boosters on the basis of ACIP recommendations.

Vaccination serum samples will be collected on the day of vaccination before receiving vaccinations on that visit. These samples will be analyzed to evaluate response to vaccinations if the participant tests positive for infection with *S pneumoniae*, *N meningitidis*, or *H influenzae*.

**Laboratory Assessments: Section 11.1.9**

**Table 4: Laboratory Assessments, Footnote f**

f. Serum samples will be collected to evaluate response to vaccinations to *N meningitidis* (types A, C, W, and Y, and B), PCV13, PCV20, or PPSV23, and Hib. Samples will only be analyzed in the event that the participant has a positive infection for *Streptococcus pneumoniae*, *Neisseria meningitidis*, or *Haemophilus influenzae*.

**Switzerland**

**Rationale for Modifications**

These modifications to the APL2-C3G-310 protocol are being implemented in Switzerland to support the Investigational Testing Authorization for the Crono Super PID infusion pump (version 2).

**Summary of Changes**

Changes in the sections noted below will be enacted by this addendum.

**Section 9.4, Infusion Supplies**

The sponsor will provide infusion supplies supply syringes, vial adapters, infusion sets, and ambulatory syringe infusion pumps, as required. Refer to the pharmacy manual for further details.

This clinical study is a combined trial with medical devices. The Crono Super PID infusion pump (version 2) is an electromechanical, software-controlled, reusable infusion pump manufactured by CANÈ S.p.A. Medical Technology (Via Cuorgnè 42/a, 10098 RIVOLI - Cascine [TO] – Italy) that is supplied to the participant and used throughout the duration of the study. The CRN Crono 20 mL syringe reservoirs are sterile, nonpyrogenic, single-use syringes, specifically for use with Crono infusion pumps. Since the initiation of this clinical study, CANÈ S.p.A. Medical Technology has modified the intended use of the Crono Super PID infusion pump, and it is no longer licensed for administration of immunoglobulins and drugs in general, but only for the administration of immunoglobulins. Given this change, the use of the Crono Super PID infusion pump in this study is considered to be investigational.

The infusion pump and syringe reservoirs will be used in conjunction with the Neria multi-infusion set (Unomedical Devices S.A de C.V, Aaholmvej 1-3, Osted, 4320 Lejre Denmark). And the vial adaptor (West Pharma. Services IL, Ltd., 4 Hasheizaf St., Ra'Anana, Israel, 4366411), which are *conformité européenne* (CE)-marked and approved for use in Switzerland.

### 9.4.1 Investigational Medical Devices: Crono Super PID Infusion Pump and CRN Crono 20 mL Syringe Reservoir

#### 9.4.1.1 Justification for the Use of the Investigational Medical Devices

The Crono Super PID infusion pump was selected for use in this trial as it is the only available subcutaneous infusion pump that enables blinded administration of pegcetacoplan or the placebo used in this study by allowing the same delivery time despite their differing viscosities.

Apellis assessed the potential hazards associated with the delivery of the drug product in relation to the uses for which the pump carries a CE mark in the European Union. The hazards evaluated were related to the required Critical Performance Attributes, the user profile, and use environment relevant to use with the drug product in the clinical trial. This assessment included testing to confirm performance to the required delivery requirements and the delivery process and evaluation of the user population compared with those for which these devices are already CE-marked for use.

The assessment concluded that the Crono Super PID infusion pump could meet the delivery requirements of Study APL2-C3G-310, does not present any unique usability issues to the user groups in Study APL2-C3G-310, and does not introduce any additional or residual risks.

The use of the investigational devices in human participants is justified based on the design and intended uses of the device constituent parts (Crono Super PID infusion pump and CRN Crono 20 mL syringe reservoir), which are appropriate.

#### 9.4.1.2 Risks of Investigational Medical Devices

The use of this delivery system with this drug in this clinical trial does not create any risks additional to the residual risk inherent with the current use of these devices. There is a possibility that participants could experience redness, swelling, and pain or tenderness at the site of infusion. There is also a slight possibility of infection. In addition, as pegcetacoplan is to be administered by subcutaneous infusion, participants may feel some discomfort from the infusion. None of these risks are affected by the use of these specific devices.

### 9.4.2 Reporting of Device Deficiencies and Adverse Events Related to Investigational Medical Devices

All device deficiencies and serious adverse events where a relationship with the use of investigational medical devices cannot be ruled out must be reported to Apellis immediately, within 24 hours of the investigator or their representative becoming aware of the event. The site must complete the complaints form and email it to Complaints@Apellis.com immediately, within 24 hours of becoming aware of the event.

The sponsor is responsible for submitting reports of device deficiencies and serious adverse events related to the use of investigational medical devices, as described in according to Art. 33 para. 1 of the ClinO-MD, to SwissMedic and the device manufacturer within 72 hours of becoming aware of the issue. The device manufacturer will then further assess the incident per the timelines in the guidance and report their findings accordingly.

#### 9.4.2.1 Definitions for Reporting of Safety Events Related to Investigational Medical Devices

For the purpose of reporting safety events related to investigational medical devices, the following definitions will apply:

**Investigational Device**

A device that is assessed in a clinical investigation.

**Adverse Event**

Any untoward medical occurrence, unintended disease or injury, or any untoward clinical signs, including an abnormal laboratory finding, in participants, users or other persons, in the context of a clinical investigation, whether or not related to the investigational device. Note that this definition includes events that are anticipated as well as unanticipated events. This definition also includes events occurring in the context of a clinical investigation related to the investigational device, the comparator, or the procedures involved.

**Adverse Device Effect**

Any adverse event related to the use of an investigational medical device or a comparator.

**Anticipated Serious Adverse Device Effect**

Any serious adverse device effect which by its nature, incidence, severity, or outcome has been identified in the last risk assessment document upon serious adverse device effect occurred.

**Causality Assessment**

Safety events related to investigational medical devices should be classified as not related, possibly related, probably related, or definitely related to the use of the investigational medical device, according to the definitions in section 9 of the EU guidance (Safety Reporting in Clinical Investigations of Medical Devices Under the Regulatory [EU] 2017/745, MDCG2020-10/1, 2020).

**Device Deficiency**

Any inadequacy in the identity, quality, durability, reliability, safety, or performance of an investigational device, including malfunction, use errors, or inadequacy in information supplied by the manufacturer.

**Incident**

Any malfunction or deterioration in the characteristics or performance of a device made available on the market, including use-error due to ergonomic features, as well as any inadequacy in the information supplied by the manufacturer and any undesirable side-effect.

**Reportable Serious Adverse Device Effect**

Serious adverse event that is either possibly, probably, or definitely related to the use of the investigational medical devices. This includes relationship to the device or the procedure.

**Serious Adverse Device Effect**

Any adverse device effect that has resulted in any of the consequences characteristic of a serious adverse event.

**Serious Incident**

Any incident that directly or indirectly led, might have led, or might lead to any of the following:

- the death of a participant, user, or other person
- the temporary or permanent serious deterioration of a participant's, user's, or other person's state of health
- a serious public health threat

# Appendix 3. COVID-19 Study Continuity Plan

## A.3.1. Protocol Changes to be Followed During COVID-19 Restrictions

### A.3.1.1. Overview

As a response to the COVID-19 global public health emergency, Apellis will implement the measures outlined in this addendum to safeguard the rights, welfare, and safety of Study APL2-C3G-310 participants and investigative site staff when and if deemed necessary. These measures are based on guidance issued by global regulatory health authorities regarding the impact of COVID-19 on the management of clinical trials and can only be implemented at each clinical investigative site after written approval from the sponsor.

### A.3.1.2. Minimum Study Requirements

#### A.3.1.2.1. Safety Assessments

When feasible, the full list of protocol assessments should be conducted; however, the following are the **minimum** study safety requirements for Study APL2-C3G-310:

1. **Maintain** dosing schedules and management per protocol guidelines
2. **Obtain** key safety laboratory assessments:
    a. All laboratory assessments will be drawn at the frequency required by the minimized Schedule of Events (see Table A1), at a minimum. Collection of more than the minimum activities described in Table A1 should continue to the extent possible, and investigators should resume following the full schedule of activities as soon as possible.
    b. In the event that the clinical investigative site is closed and/or participants are unable or unwilling to travel, participants will be referred to a certified local laboratory that can perform the required safety testing specified in Table A1. Alternatively, a home health care provider may be sent to the participant's home to draw samples for the minimum laboratory tests required for safety testing (see Section A.3.1.5), which may then be sent to the central laboratory or to a local laboratory. Use of the central laboratory is preferable whenever feasible. In the event that a local laboratory is used, the investigator will collect the local laboratory reports, which must be redacted in accordance with local privacy requirements. The laboratory results, reference ranges, units of measure, and format of laboratory values are to be filed with the study records.
3. **Perform** minimal medical safety assessments:
    a. If the participant is unable to have an in-person assessment, the investigator (or delegated site staff) will have a telephone and/or video contact at the frequency noted in the protocol (at minimum) to solicit adverse events (including serious adverse events) and concomitant medications. The appropriate forms in the electronic case report form will be completed.

   b. **Serious adverse event reporting requirements are unchanged, and all serious adverse events are still required to be reported to Apellis within 24 hours of the investigator or their representative becoming aware of the event(s)**.

4. **Document** appropriate contact with the participant:
   a. At least 3 documented attempts, including a certified letter with return receipt, must be made to solicit adverse events and concomitant medications before considering the visit missed. If contact with the participant still cannot be established for the following visit after another 3 documented attempts, participant is considered lost to follow-up. The clinical research associate and medical monitor must be notified of any participants lost to follow-up.
   b. All communication with the participant (including failed attempts at contact) must be documented in the study records.

#### A.3.1.2.2. Efficacy Assessments

The participants must be able to complete the following assessments to continue in the trial:

- Renal biopsies during screening and at week 26 (adult participants only)
- Triplicate first-morning urine collection during screening and at weeks 26 and 52
- Serum chemistry and hematology during screening and at weeks 26 and 52

### A.3.1.3. Screening

#### A.3.1.3.1. Study Screening

A participant cannot be considered for screening using the minimum study requirements. The screening and baseline assessments must remain unchanged and must be completed in their entirety. The sponsor may decide to pause screening at any point because of the COVID-19 pandemic, at which point sites will be notified and should not attempt to screen new participants.

For sites continuing at full capacity (and not using the minimum study requirements), the investigator should assess and appropriately document the risk/benefit of participants continuing to come to the clinic for visits. These sites may continue to screen new participants using the protocol-outlined procedures.

#### A.3.1.3.2. Institutional Screening for Public Health or Clinical Purposes

##### A.3.1.3.2.1. Mandatory Clinical Screening Procedures

According to local guidance, if an institution implements mandatory public health screening procedures related to a public health emergency (eg, COVID-19, measles, etc) for all individuals coming into that institution, inclusive of research participants, IRB approval is not required before screening procedures are implemented. Additionally, because these screening procedures are not research procedures, the institution does not require IRB approval to share results with a public health authority or research participants, although adherence to other permissions or notice restrictions remain under applicable law and/or policy (see Section A.3.1.12).

#### A.3.1.3.2.2. Excluded Public Health Surveillance Activities

Some types of public health surveillance activities, including collection and testing of information or biospecimens, conducted, supported, requested, ordered, required, or authorized by a public health authority, may be conducted per local, regional, and national regulation and guidance as applicable (see Section A.3.1.12).

If a public health authority authorizes general screening for COVID-19 for public health surveillance purposes and requests that test results be shared as necessary with a public health authority to allow the public health authority to identify, monitor, assess, or investigate the COVID-19 outbreak, an investigator may incorporate these activities into an existing research visit without prior IRB review and approval.

Note, however, that US Food and Drug Administration regulations may apply if the screening procedure involves use of an investigational in vitro diagnostic device.

### A.3.1.4. Participants Testing Positive for COVID-19

The following are considerations and changes that might need to be made for participants who test positive for COVID-19.

**If a research participant tests positive for COVID-19**

1. Follow the clinical recommendations of the study site and public health authorities to manage the infection.
2. Follow the notification requirements of the study site and public health authorities. Investigators should inform the participant of the required reporting of results. Note: The results of the COVID-19 test should be recorded in the COVID-19 electronic case report form and documented as an adverse event if the result is positive.
3. Determine whether a positive test result meets criteria for withdrawal from the study, or for reporting as a serious adverse event by contacting the sponsor and notifying IRBs/ethics committees and/or health authorities as required per local guidelines.

### A.3.1.5. COVID-19 Vaccination

In the absence of data, Apellis currently recommends that the decision to vaccinate study participants against COVID-19 should be made by the investigator and the participant, with consideration of local and national vaccination recommendations and a benefit-risk assessment. Vaccination is not mandatory for continued study participation.

Apellis is currently not aware of any contraindications to vaccination (of any modality) and concurrent treatment with pegcetacoplan. Precaution should be taken when vaccinating participants with any concurrent illnesses that may affect the efficacy or safety of vaccination (eg, complement-activating condition) and as indicated by the labeled prescribing information.

All vaccinations (including boosters) should be recorded as concomitant medications. The information should include the type of COVID-19 vaccination administered. Any untoward medical events related to vaccination should be captured in the participants source document and should include the timing in relation to the vaccine. Any questions should be directed to the medical monitor or study team.

#### A.3.1.6. Home Health Care Provider

In the event that the clinical investigative site is closed and/or participants are unable or unwilling to travel, home health care providers (HHCPs) may be used to perform study activities, such as the below, with participants' consent:

- Blood draw
- Investigational product administration
- Investigational product accountability
- Study assessments

HHCPs will be required to follow all safety precautions set by the local guidance/regulations prior to and after contact with participant(s). HHCPs will summarize the activities completed, including any reported AEs, at the visit in a home visit report, which will be provided to the clinical investigative site for review and to file in the study records.

#### A.3.1.7. Monitoring

Remote monitoring measures may be implemented with participants' consent in accordance with local and institutional guidelines. Methods to review and perform remote monitoring will be outlined in the sponsor's clinical monitoring plan.

#### A.3.1.8. Investigational Product

To ensure that participants can continue treatment during the COVID-19 public health emergency and the associated changes with these clinical trials, additional study drug may need to be dispensed to participants or shipped to their homes. Additional dispensation of study drug and/or direct shipment of study drug to participants' homes will only be implemented if permitted by local regulations, in conjunction with participant consent and corresponding ethics committee and competent authority approval, as required. Participants are to return all study drug to the investigator site once on-site visits are permitted to enable full study drug accountability.

#### A.3.1.9. Regulatory Considerations

Actions taken as a result of the COVID-19 public health emergency (protocol deviations, urgent safety measures, etc) that fall outside of standard clinical trial conduct guidance and regulations should be documented as appropriate in the study file. Ensure compliance with all local, regional, and national regulation and guidance, as applicable.

#### A.3.1.10. Statistical Considerations

In accordance with the local, regional, and national guidelines, the SAP for the study might need to be updated on the basis of the guidance provided by regulatory authorities related to the COVID-19 public health emergency.

#### A.3.1.11. Schedule of Activities—Minimum Study Requirements

For all screening visits, day 1, and week 26, the visits must occur in clinic, and all assessments on those days are required. Every effort should be made to conduct a full and in-person visit at week 52, but if COVID-19 restrictions make that impossible, the visit may be conducted virtually or by an HHCP after consultation with the medical monitor. The remainder of the required visits can be done virtually or by an HHCP, and for these visits, the required assessments are indicated. Visits and/or assessments not required as part of the minimum activities are shown in strikethrough text in Table A1.

## Table A1: Revised COVID-19 Minimum Schedule of Activities

| **Study period** | **Screening period[a]** | | **Randomized controlled period** | | | | | | | | **Open-label period** | | | | | | **Follow-up period** | | |
|---|---|---|---|---|---|---|---|---|---|---|---|---|---|---|---|---|---|---|---|
| **Study week** | **–10 to –4** | **–2** | **1** | **4** | **~~8~~** | **12** | **~~16~~** | **20** | **24** | **26** | **28** | **32** | **~~36~~** | **42** | **48** | **52** | **~~54~~** | **56** | **60 Exit** |
| **Study day** | **–70** | **–14** | **1** | **28** | **~~56~~** | **84** | **~~112~~** | **140** | **168** | **182** | **196** | **224** | **~~252~~** | **294** | **336** | **364** | **~~378~~** | **392** | **420** |
| **Study visit** | **1** | **2** | **3** | **4** | **~~5~~** | **6** | **~~7~~** | **8** | **9** | **10** | **11** | **12** | **~~13~~** | **14** | **15** | **16** | **~~17~~** | **18** | **19** |
| **Visit window (± days)** | **N/A[b]** | | **0** | **3** | **~~7~~** | **7** | **~~7~~** | **7** | **3** | **3** | **3** | **7** | **~~7~~** | **7** | **7** | **7** | **~~3~~** | **3** | **7** |
| **<u>Assessments</u>** | | | | | | | | | | | | | | | | | | | |
| Informed consent | X | | | | | | | | | | | | | | | | | | |
| Demographics | X | | | | | | | | | | | | | | | | | | |
| Medical history | X | | | | | | | | | | | | | | | | | | |
| Post-transplant immunosuppression plan documentation[c] | | X | | | | | | | | | | | | | | | | | |
| Inclusion/exclusion | X | X | X | | | | | | | | | | | | | | | | |
| Vaccination[d] | | X[b] | | | | X | | | | | | | | | | | | | |
| Adverse events | X | X | X | X | ~~X~~ | X | ~~X~~ | X | X | X | X | X | ~~X~~ | X | X | X | ~~X~~ | X | X |
| Concomitant medications | X | X | X | X | ~~X~~ | X | ~~X~~ | X | X | X | X | X | ~~X~~ | X | X | X | ~~X~~ | X | X |
| Physical examination (full)[e] | X | | X | | | | | | | X[e] | | | | | | X | | | |
| Physical examination (brief)[e] | | X | | ~~X~~ | ~~X~~ | ~~X~~ | ~~X~~ | ~~X~~ | ~~X~~ | | ~~X[e]~~ | ~~X~~ | ~~X~~ | ~~X~~ | ~~X~~ | | ~~X~~ | ~~X~~ | ~~X~~ |
| 12-lead ECG | X | | X | | ~~X~~ | | ~~X~~ | | ~~X~~ | X | ~~X~~ | | ~~X~~ | | ~~X~~ | X | ~~X~~ | | ~~X~~ |
| Chest radiography | | X[b] | | | | | | | | X | | | | | | X | | | |
| Renal biopsy[f] | X[b] | | | | | | | | | X[g] | | | | | | ~~X[h]~~ | | | |
| Randomization | | | X | | | | | | | | | | | | | | | | |
| Study drug administration[i] | | | X | X | ~~X~~ | X | ~~X~~ | X | X | X | X | X | ~~X~~ | X | X | X | | | |
| Infusion site/pump safety assessment[j] | | | X | X | ~~X~~ | X | ~~X~~ | X | X | X | X | X | ~~X~~ | X | X | X | ~~X~~ | | |
| Vital sign measurements[k] | X | X | X | ~~X~~ | ~~X~~ | ~~X~~ | ~~X~~ | ~~X~~ | ~~X~~ | X | ~~X~~ | ~~X~~ | ~~X~~ | ~~X~~ | ~~X~~ | X | ~~X~~ | ~~X~~ | ~~X~~ |
| HRQoL[l] | | X | | | | | | | | X | | | | | | X | | | |

## Table A1: Revised COVID-19 Minimum Schedule of Activities

| Study period | Screening period[a] | | Randomized controlled period | | | | | | | | Open-label period | | | | | | Follow-up period | | |
|---|---|---|---|---|---|---|---|---|---|---|---|---|---|---|---|---|---|---|---|
| **Study week** | **–10 to –4** | **–2** | **1** | **4** | **~~8~~** | **12** | **~~16~~** | **20** | **24** | **26** | **28** | **32** | **~~36~~** | **42** | **48** | **52** | **~~54~~** | **56** | **60 Exit** |
| **Study day** | **–70** | **–14** | **1** | **28** | **~~56~~** | **84** | **~~112~~** | **140** | **168** | **182** | **196** | **224** | **~~252~~** | **294** | **336** | **364** | **~~378~~** | **392** | **420** |
| **Study visit** | **1** | **2** | **3** | **4** | **~~5~~** | **6** | **~~7~~** | **8** | **9** | **10** | **11** | **12** | **~~13~~** | **14** | **15** | **16** | **~~17~~** | **18** | **19** |
| **Visit window (± days)** | **N/A[b]** | | **0** | **3** | **~~7~~** | **7** | **~~7~~** | **7** | **3** | **3** | **3** | **7** | **~~7~~** | **7** | **7** | **7** | **~~3~~** | **3** | **7** |
| **Urine** | | | | | | | | | | | | | | | | | | | |
| 24-hour urine collection[m] | X | | | | | X | | | X | | | | ~~X~~ | | X | | | | X |
| Triplicate FMU uPCR[n] | X | X | X[o] | X | ~~X~~ | X | ~~X~~ | X | X | X | X | X | ~~X~~ | X | X | X | ~~X~~ | X | X |
| In-clinic (random) uPCR | X | X | X | ~~X~~ | ~~X~~ | ~~X~~ | ~~X~~ | ~~X~~ | ~~X~~ | X | ~~X~~ | ~~X~~ | ~~X~~ | ~~X~~ | ~~X~~ | X | ~~X~~ | ~~X~~ | ~~X~~ |
| Urinalysis (dipstick & microscopic)[p] | X | | | | | | | | | | | | | | | | | | |
| Urine pregnancy test | | X | X | X | ~~X~~ | X | ~~X~~ | X | X | X | X | X | ~~X~~ | X | X | X | ~~X~~ | X | X |
| **Blood** | | | | | | | | | | | | | | | | | | | |
| Hematology[p] & chemistry[p] | X | X | X | X | ~~X~~ | X | ~~X~~ | X | X | X | X | X | ~~X~~ | X | X | X | ~~X~~ | X | X |
| PK sample collection | | | X | X | ~~X~~ | X | ~~X~~ | ~~X~~ | ~~X~~ | X | X | X | ~~X~~ | ~~X~~ | ~~X~~ | X | ~~X~~ | X | X |
| ADA assays[q] | | | X | ~~X~~ | | | ~~X~~ | | ~~X~~ | | | | ~~X~~ | | | X | ~~X~~ | | X |
| Serum complement profile[p] | X | X | X | ~~X~~ | ~~X~~ | X | ~~X~~ | ~~X~~ | ~~X~~ | X | X | ~~X~~ | ~~X~~ | | ~~X~~ | X | ~~X~~ | X | X |
| Plasma complement profile[p] | X | | X | | ~~X~~ | | ~~X~~ | | ~~X~~ | X | ~~X~~ | | ~~X~~ | | ~~X~~ | X | ~~X~~ | | X |
| eGFR[r] | X | X | X | X | ~~X~~ | X | ~~X~~ | X | X | X | X | X | ~~X~~ | X | X | X | ~~X~~ | X | X |
| Pregnancy (β-HCG) | X | | | | | | | | | | | | | | | | | | |
| Screening assays[s] | X | | | | | | | | | | | | | | | | | | |
| **Optional assessments** | | | | | | | | | | | | | | | | | | | |
| ~~Ophthalmologic evaluations[t]~~ | | ~~X[t]~~ | | | | | | | | | | | | ~~X[t]~~ | | | | | |
| ~~Measured GFR[u]~~ | | | ~~X[u]~~ | | | | | | | ~~X[u]~~ | | | | ~~X[u]~~ | | | | | |

## Table A1: Revised COVID-19 Minimum Schedule of Activities

Abbreviations: Ab = antibodies; ADA = antidrug antibodies; AE = adverse events; AH50 = 50% alternative hemolytic complement pathway activity; ANA = antinuclear antibodies; ANCA = antineutrophil cytoplasmic antibodies; β-HCG = beta human chorionic gonadotropin; CH50 = 50% classical hemolytic complement pathway activity; ECG = electrocardiogram; eGFR = estimated glomerular filtration rate; EQ-5D-5L = 5-Level EuroQol-5 Dimension; FACIT-Fatigue = Functional Assessment of Chronic Illness Therapy- Fatigue Scale; FMU = first-morning spot urine; FSH = follicle-stimulating hormone; GFR = glomerular filtration rate; HIV = human immunodeficiency virus; HRQoL = health-related quality of life; KDQOL = Kidney Disease Quality of Life; N/A = not applicable; PGIC = Patient Global Impression of Change; PK = pharmacokinetic; SC = subcutaneous; SD-OCT = spectral domain optical coherence tomography; SPEP = serum protein electrophoresis; uPCR = urine protein-to-creatinine ratio; WPAI = Work Productivity and Activity Impairment.

**NOTE:** When multiple assessments/procedures occur at the same visit, order should be: vital signs, ECGs, blood collection/sampling, study drug dosing.

a) All visit 2 assessments need not occur in a single visit and visit 2 can be split into multiple visits (eg, visit 2a, visit 2b).

b) Vaccinations, baseline renal biopsy, and screening chest radiography can occur any time during screening after confirmation of eligibility based on visit 1 data.

c) For transplant participants only. Must be documented prior to randomization.

d) Vaccine series should be initiated at least 14 days prior to randomization; additional vaccines may be required at visit 6. Please see Section 8.2 for more details on vaccination requirements. Vaccination serum samples will be collected on the day of vaccination prior to receiving vaccinations on that visit. These samples will be analyzed to evaluate response to vaccinations in the event that the participant has a positive infection for *Streptococcus pneumoniae*, *Neisseria meningitidis*, or *Haemophilus influenzae*.

e) A full physical examination is required at visits 1, 3 (week 1), 10 (week 26), and 16 (week 52). A full physical examination should also be conducted on the first day of open-label dosing, if that not at the week 26 visit. Brief physical examinations, including weight (kg) and assessment of edema, will be conducted at all other visits noted. A symptom-driven physical examination may be performed at any time, at the investigator's discretion. Body height (cm) will be measured only during screening for adults but will be measured at screening as well as every 12 weeks throughout the study for adolescents; weight (kg) will be measured throughout study, during brief and full physical examinations for all participants. Both body weight and height will be assessed without shoes on; height will be measured using a calibrated stadiometer. Edema should be assessed at every visit.

f) Renal biopsies will not be required for participants under the age of 18, provided they have an adequate prior renal biopsy to establish the diagnosis as per the central pathology laboratory.

g) The week 26 renal biopsy need not occur on the same day as all other assessments for that visit. However, the week 26 triplicate uPCR collections must occur prior to the renal biopsy. In addition, the week 26 biopsy must occur prior to the first visit of the open-label period at week 28. Participants younger than 18 years are not required to provide renal biopsies and may advance to the open-label period upon completion of all assessments for weeks 24 through 26 other than the renal biopsy.

h) The week 52 renal biopsy is optional for all participants. If performed it should be after collection of the week 48 24-hour urine and FMU samples and not more than 8 weeks after the week 52 visit, In the event that a participant has a renal biopsy as part of their clinical management within this window, it may serve as the week 52 biopsy provided that it includes the required components for this study.

i) Study drug will be self-administered by the participant or administered by their caregiver, after receiving appropriate training and sign-off by a research nurse (or other qualified personnel) in their first treatment week, as described in Section 9.5. Once qualified, the participant or caregiver should administer study drug at site visits (as done at home) on those days when a clinic visit occurs on a dosing day. In the event that a home nurse is administering study drug on nonvisit days, then the site staff may administer study drug on days of site visits.

j) Between site visits, participants will be instructed to report any infusion site reaction to the study staff. Pump use safety will be reviewed by licensed health care professionals (ie, investigator or nurse) for each study drug administration at clinic visits and during at-home qualification.

k) Vital signs should be measured a maximum of 2 hours before study drug infusion. On day 1 and on the first day of open-label dosing (week 26 or week 28, depending on whether a week 26 renal biopsy is required and when it is performed), vital signs should also be measured approximately 30 minutes to 1 hour after the first infusion of study drug dosing, timed from the completion of the study drug administration. Blood pressure and heart rate should be evaluated after the participant has been resting in a seated position for at least 5 minutes, except when they are supine or semireclined because of study procedures and/or AEs, or if deemed necessary by the investigator.

l) FACIT-Fatigue, EQ-5D-5L, KDQOL, PGIC, and WPAI.

**Table A1: Revised COVID-19 Minimum Schedule of Activities**

m) The screening 24-hour urine collection may be done any time between screening visit 1 and screening visit 2. After week 1 collections should be within ±1 week of the visit (except for the week 24 collection, which cannot be earlier than week 24). Courier arrangements can be made to pick up the collection container from the participant, or the participant may return the container directly to the site.

n) Triplicate FMU samples will be collected by the participant at home on 3 consecutive days throughout the duration of the study. These should be the first urinary output of the day. An additional triplicate FMU uPCR sample will be collected at week 25. Samples should be collected within ±1 week of the clinic visit (except for weeks 24 through 26, when sample collection should be within ±3 days of the clinic visit). Courier arrangements can be made by the site to pick up the collection containers from the participants, or the participant may return them directly to the site. At every visit, enough uPCR collection containers need to be dispensed to the participant to enable all at-home uPCR collections until the next clinic visit.

o) The day 1 triplicate FMU uPCR samples should be collected before the first dose of study drug (eg, day –2, day –1, and before dosing on day 1).

p) Serum complement profile includes AH50, CH50, and C3NeF; C3NeF will only be assayed in samples collected at the baseline (screening) visits. Plasma complement profile includes C3a, C3b/iC3b, C5a, and sC5b-9. See laboratory assessments (Table 4) for more details.

q) The day 1 samples should be collected before dosing with study drug. Participants who discontinue dosing will have ADA samples collected at 2 and 8 weeks after the last treatment. Participants who have a treatment-emergent or treatment-boosted antidrug antibody response at any time will have ADA samples collected approximately every 6 months until the antibody levels revert to baseline.

r) eGFR will be calculated using the Chronic Kidney Disease–Epidemiology Collaboration creatinine equation for adults or the Bedside Schwartz equation for adolescents. For each participant, eGFR will be calculated using the same formula for the duration of the study; the choice of formula will be determined by the participant's age at study entry.

s) Serum FSH (to be measured in female participants only), hepatitis B panel, hepatitis C panel, HIV antibodies, SPEP (adult participants only), ANA, and ANCA (see Table 4 and laboratory manual for more details).

t) Ophthalmologic evaluations are optional and will be performed at selected sites. If evaluations are performed, each participants should have a baseline ophthalmologic evaluation, including a basic ophthalmologic examination, SD-OCT, and color fundus photography, at an approved ophthalmologic clinical site at any time during the screening period. For participants with drusen prior to pegcetacoplan administration, a follow-up ophthalmologic evaluation, including an SD-OCT and color fundus photography, should occur at a convenient time between weeks 42 and 52.

u) Measured GFR is an optional assessment that, if performed, should occur at 3 time points: once on day 1 or within 10 weeks before day 1, again at week 26, and a third time between week 42 and week 52, inclusive. Measured GFR should only be done at sites where it is routinely performed, as per the site's standard protocol.

### A.3.1.12. Regulatory Guidances

European Medicines Agency. Guidance on the management of clinical trials during the COVID-19 (Coronavirus) pandemic. Published 28 April 2020. Accessed 24 August 2020. https://ec.europa.eu/health/sites/health/files/files/eudralex/vol-10/guidanceclinicaltrials_covid19_en.pdf

Food and Drug Administration, US Department of Health and Human Services. Statistical considerations for clinical trials during the COVID-19 public health emergency: guidance for industry. Published 16 June 2020. https://www.fda.gov/regulatory-information/search-fda-guidance-documents/statistical-considerations-clinical-trials-during-covid-19-public-health-emergency-guidance-industry

Office for Human Research Protections, US Department of Health and Human Services. OHRP guidance on coronavirus. Published 2020. Updated 08 April 2020. Accessed 24 June 2020. https://www.hhs.gov/ohrp/regulations-and-policy/guidance/ohrp-guidance-on-covid-19/index.html

Office for Human Research Protections, US Department of Health and Human Services. Unanticipated problems involving risks & adverse events guidance. Published 2007. Updated 21 March 2016. Accessed 24 June 2020. https://www.hhs.gov/ohrp/regulations-and-policy/guidance/reviewing-unanticipated-problems/index.html

**SUMMARY OF VALIANT PROTOCOL CHANGES**

| **Protocol v2 Amendments – Description of Change** |
|---|
| Changed planned enrollment from 90 to 80-100 subjects |
| Clarified vaccination strategy and prophylactic antibiotic use in Risk/Benefit discussion |
| Changed primary and key secondary objectives to align with endpoint changes |
| Changed from 10 posttransplant minimum to 77 native kidney minimum |
| Added 8-adolescent minimum |
| Revised study scheme diagram |
| Changed primary efficacy endpoint to remove eGFR component |
| Removed proportion of subjects with uPCR reductions of at least 50% as a secondary efficacy endpoint |
| Changed primary and key secondary efficacy endpoints from Week 52 to Week 26 |
| Revised and reorganized secondary and exploratory endpoints (including timing of evaluations) |
| Increased minimum body weight to 30 kg |
| Updated inclusion criteria to define evidence of active renal disease and remove central medical review |
| Updated inclusion criteria to define acceptable stable regimens for treatment of C3G/IC-MPGN |
| Revised replacement of subjects section to clarify that subjects who discontinue will not be replaced |
| Clarified wording of renal biopsies in adolescents section to explicitly note that biopsies will not be collected for research purposes from adolescent subjects in the US |
| Created separate subsection in Concomitant Medications describing permitted prior medications |
| Added section discussing possible rescue treatment for C3G/IC-MPGN |
| Added specification of use of Bedside Schwartz equation in adolescents where missing |
| Added chest radiography to schedule of activities and discussion of safety procedures |
| Removed fibrinogen and added prothrombin time to coagulation section of laboratory assessments table |
| Added section describing AEs of special interest |
| Removed discussion of extrapolating placebo response rate from Week 26 to Week 52 |
| Removed potential sample size revision |
| Changed baseline, Week 26, and Week 52 uPCR calculations to the average of 6 to 9 samples from the average of 9 samples |
| Added discussion of primary and alternate estimands |
| Added discussion of intercurrent events and how they will be handled in assessment of the primary endpoint |
| Updated descriptions of calculations of responder analysis for uPCR and eGFR endpoints |
| Revised and clarified descriptions of analysis of secondary endpoints |
| Removed interim analyses (interim analysis 1 for sample size readjustment and interim analysis 2 for early efficacy assessment) |
| Added section describing maximum blood collection volumes |
| Removed central medical review for confirmation of diagnosis and eligibility |
| Clarified that Apellis representatives will not review unblinded data |
| Added COVID-19 vaccine to COVID-19 risk mitigation section and COVID-19 Appendix |
| Added appendix listing prior revision history of the document |
| **Protocol v3 Amendments – Description of Change** |
| Updated summary of clinical experience to include Study APL2-308 |
| Increased duration of screening period from 8 to 10 weeks with a corresponding increase of total duration of study participation to 70 weeks |
| Increased the minimum number of adolescent participants from 8 to 10 |
| Revised study scheme diagram |
| Changed primary efficacy endpoint from the proportion of subjects with a reduction from baseline in uPCR of at least 50% to the log-transformed ratio of uPCR at week 26 compared to baseline |

| |
|---|
| Changed key secondary efficacy endpoint to the proportion of participants who meet the criteria for achieving a composite renal endpoint (a stable or improved eGFR compared to the baseline visit (≤15% reduction in eGFR), and a ≥50% reduction in uPCR compared to the baseline visit.) |
| Escalated change in eGFR to a key secondary endpoint |
| Added the proportion of participants with a reduction of at least 50% from baseline in uPCR as a key secondary endpoint |
| Removed change from baseline in uPCR as an additional secondary efficacy endpoint |
| Revised and reorganized exploratory endpoints to align with changes to primary and secondary endpoints |
| Reorganized pharmacokinetic, pharmacodynamic, and immunogenicity endpoints to appear in a single heading |
| Clarified that biopsy-based inclusion criteria apply to both baseline biopsies collected as part of the study and historical biopsies collected within 28 weeks of randomization and used as baseline biopsies |
| Added sodium-glucose cotransporter-2 inhibitors to the description of stable treatment regimens for C3G/IC-MPGN |
| Updated the time requirements for stable dosing regimens to remove reference to baseline renal biopsy; timing of stable treatment requirements is now based solely on randomization |
| Added recent severe infection as an exclusion criterion |
| Clarified when open-label dosing will begin |
| Updated vaccination guidance to recognize the potential use of local guidelines as an alternative to ACIP guidelines |
| Updated discussion of vaccination against S pneumoniae to reflect recent changes to ACIP guidelines |
| Removed testing of C3Nef from samples collected after screening |
| Clarified the timing of the collection of baseline and week 26 renal biopsies and the requirements for using a prior biopsy as the baseline biopsy |
| Clarified requirements for chest radiography, moved baseline radiography to visit 2 |
| Updated discussion of assessment of severity of adverse events |
| Updated description of procedures for reporting SAEs to Apellis |
| Updated description of procedures for monitoring and reporting pregnancies to Apellis |
| Updated discussion of the determination of sample size |
| Updated the discussion of the efficacy analyses |
| Clarified screening procedures, including rescreening activities |
| Clarified discussion of study treatment discontinuation, study withdrawal, and the activities to be undertaken in these situations |
| Provided guidance for the resumption of dosing if regularly scheduled doses are missed |
| Clarified discussion of study treatment blinding requirements |
| Added the potential to ship investigational product and/or ancillary study supplies directly to the participant, where permitted |
| Clarified guidance regarding infusion sites |
| Updated Responsible Medical Director |
| Changed wording from "subjects" to "participants" |
| **Protocol v4 Amendments – Description of Change** |
| Order of key secondary endpoints revised; change in eGFR at 26 weeks now the last key secondary endpoint |
| Removed exploratory endpoint evaluating normalization of hematuria |
| Removed exploratory endpoint of evaluation of changes in drusen at 26 weeks |
| Revised description of the window for conducting the optional activities (ophthalmologic evaluations and measured GFR) |
| Moved definition of TEAEs and the period for recording and reporting TEAEs |
| Updated strategies for addressing ICEs |
| Revised description of the data review for the analysis set |

| |
|---|
| Clarified the analysis set for the evaluation of endpoints related to renal biopsy observations |
| Added description of the activities at the closure of enrollment |
| Revised description of antidrug antibody assessments |
| Added an Appendix containing countryspecific modifications to the protocol. Country-specific modifications are noted for Canada, Czech Republic, France, Germany, Netherlands, Israel, Japan, and Switzerland. |

# STATISTICAL ANALYSIS PLAN

# Pegcetacoplan (APL-2)

**PHASE 3**

# A Phase 3, Randomized, Placebo-Controlled, Double-Blinded, Multicenter Study to Evaluate the Efficacy and Safety of Pegcetacoplan in Patients with C3 Glomerulopathy or Immune-Complex Membranoproliferative Glomerulonephritis

**PROTOCOL IDENTIFIER: APL2-C3G-310**

| | |
|---|---|
| **Study Sponsor(s)**: | Apellis Pharmaceuticals, Inc<br>100 5th Ave, Waltham, MA 02451 |
| **IND Number** | 136409 |
| **EudraCT Number** | 2020-003767-25 |
| **Protocol Version** | Amendment 3 dated 03 MAR 2023 |
| **SAP Version** | Original |
| **SAP Author** | |
| **Date of Plan** | 07 SEP 2023 |

## REVISION HISTORY

| Version | Issue Date | Summary of Changes |
|---|---|---|
| 1.0 | 07 SEP 2023 | Original |
| | | |
| | | |

## APPROVAL SIGNATURES

**Date**

**Apellis Pharmaceuticals, Inc.**

**Date**

**Apellis Pharmaceuticals, Inc.**

# TABLE OF CONTENTS

REVISION HISTORY ....2
APPROVAL SIGNATURES ....2
TABLE OF CONTENTS ....3
LIST OF TABLES ....7
LIST OF FIGURES ....7
ABBREVIATIONS ....8
1. INTRODUCTION ....10
2. OBJECTIVES AND ENDPOINTS ....11
2.1. Objectives ....11
2.1.1. Primary Objective ....11
2.1.2. Secondary Objectives ....11
2.1.3. Exploratory Objectives ....11
2.2. Endpoints ....11
2.2.1. Primary Efficacy Endpoint ....11
2.2.2. Key Secondary Efficacy Endpoints ....11
2.2.3. Additional Secondary Efficacy Endpoints ....12
2.2.4. Exploratory Efficacy Endpoints ....12
2.2.5. Safety Endpoints ....13
2.2.6. Pharmacokinetic Endpoint ....13
2.2.7. Pharmacodynamic Endpoints ....13
2.2.8. Immunogenicity Endpoint ....13
3. STUDY DESIGN ....15
3.1. General Description ....15
3.2. Randomization ....16
3.3. Blinding ....16
3.4. Sample Size ....17
3.5. Analysis Timing and Unblinding ....17
4. STATISTICAL ANALYSIS SETS ....18
4.1. Screened Set ....18
4.2. Intent-to-Treat Set ....18
4.3. Modified Intent-to-Treat Set ....18
4.4. Safety Set ....18
4.5. Per-Protocol Set ....18

4.6. Pharmacokinetic Set ....................18
4.7. Pharmacodynamic Set ....................18

5. STUDY PARTICIPANTS ....................19

5.1. Disposition of Participants ....................19
5.2. Demographic and Other Baseline Characteristics ....................19
5.3. Medical History ....................20
5.4. Prior and Concomitant Medications ....................20
5.5. Prior and Concomitant Procedures ....................21
5.6. Exposure to Investigational Product ....................21
5.7. Measurements of Treatment Compliance ....................21
5.8. Protocol Deviations ....................21

6. EFFICACY ANALYSIS ....................22

6.1. Analysis Models ....................22
6.2. Multiplicity Adjustment ....................23
6.3. Estimands ....................23
6.4. Analyses of Primary Efficacy Endpoint ....................26
6.4.1. Main Analysis of Primary Efficacy Endpoint ....................26
6.4.2. Sensitivity Analyses of Primary Efficacy Endpoint ....................27
6.4.3. Supplemental Analyses of Primary Efficacy Endpoint ....................28
6.4.3.1. Per Protocol Set ....................28
6.4.3.2. Slope Analysis ....................28
6.4.4. Subgroup Analyses of Primary Efficacy Endpoint ....................28
6.5. Analyses of Key Secondary Efficacy Endpoints ....................29
6.5.1. The Proportion of Participants Who Meet the Criteria for Achieving a Composite Renal Endpoint ....................29
6.5.1.1. Main Analysis ....................30
6.5.1.2. Supplemental Analyses ....................30
6.5.1.3. Subgroup Analyses ....................30
6.5.2. The Proportion of Participants With a Reduction of at Least 50% from Baseline in uPCR ....................31
6.5.2.1. Main Analysis ....................31
6.5.2.2. Supplemental Analyses ....................31
6.5.2.3. Subgroup Analyses ....................32
6.5.3. Change From Baseline in eGFR ....................32
6.5.3.1. Main Analysis ....................32
6.5.3.2. Sensitivity Analyses ....................32
6.5.3.3. Supplemental Analyses ....................32

6.5.3.4. Subgroup Analyses ....................33
6.5.4. Change From Baseline in the Activity Score of the C3G Histologic Index Score ....................33
6.5.4.1. Main Analysis ....................33
6.5.4.2. Sensitivity Analyses ....................33
6.5.4.3. Supplemental Analyses ....................33
6.5.4.4. Subgroup Analyses ....................34
6.5.5. The Proportion of Participants Showing Decreases in C3c Staining From Baseline ....................34
6.5.5.1. Main Analysis ....................34
6.5.5.2. Supplemental Analyses ....................34
6.5.5.3. Subgroup Analyses ....................34
6.6. Analyses of Additional Secondary Efficacy Endpoints ....................35
6.6.1. The Proportion of Participants Achieving Proteinuria <1 g/day ....................35
6.6.2. The Proportion of Participants With Normalization of Serum Albumin Levels ....................35
6.6.3. The Proportion of Participants With Serum C3 Levels Above the LLN ...36
6.6.4. Change From Baseline in FACIT-Fatigue Scale Score ....................37
6.6.5. Change From Baseline in KDQOL Score ....................37
6.7. Analyses of Exploratory Efficacy Endpoints ....................38
6.7.1. The Change From Baseline in uPCR Using the 24-Hour Urine Collections38
6.7.2. The Annual Rate of Change From up to 3 Years Prior to Screening in eGFR ....................38
6.7.3. The Proportion of Participants With Reductions From Baseline in Proteinuria of at Least 30% ....................39
6.7.4. The Proportion of Participants With Normalization of Proteinuria ....................39
6.7.5. The Proportion of Participants With Normalization of Hematuria ....................39
6.7.6. Mean Time to 50% Reduction in uPCR With a Stable or Improved eGFR39
6.7.7. Time to Normalization for Selected Parameters for Participants in Whom the Parameter is Abnormal at Baseline ....................39
6.7.8. The Change From Baseline in Glomerular Macrophage Count ....................40
6.7.9. The Change From Baseline in PGIC Score ....................40
6.7.10. The Change From Baseline in EQ-5D-5L Score ....................40
6.7.11. The Change From Baseline in WPAI Score ....................41
6.7.12. Change in Drusen From Baseline ....................41
6.7.13. Correlation Analysis on Reduction in uPCR and Change in eGFR ....................41
7. SAFETY ANALYSIS ....................42
7.1. Adverse Events ....................42

7.2. Clinical Laboratory Data....43
7.3. Vital Signs....45
7.4. Electrocardiograms (ECGs)....46
7.5. Other Safety Data....47
8. PHARMACOKINETIC ANALYSIS....48
8.1. Drug Concentration....48
8.2. Handling BLQ Values....48
8.2.1. Handling of BLQ Concentrations in Summary Tables....48
8.2.2. Handling of BLQ Concentrations in Figures....48
9. PHARMACODYNAMIC ANALYSIS....49
9.1. Pharmacodynamic Data....49
10. IMMUNOGENICITY....50
11. INTERIM ANALYSIS....53
12. DATA MONITORING COMMITTEE....54
13. DATA HANDLING CONVENTIONS....55
13.1. General Data Reporting Conventions....55
13.2. Definition of Study Days....55
13.3. Definition of Baseline, RCP, and OLP....55
13.4. Definition of Analysis Visit Windows....56
13.5. Repeated or Unscheduled Assessments of Safety Parameters....57
13.6. Handling of Missing, Unused, and Spurious Data....57
13.6.1. Missing Date of Investigational Product....57
13.6.2. Missing Date Information for Prior or Concomitant Medications....57
13.6.2.1. Incomplete Start Date....57
13.6.2.2. Incomplete Stop Date....58
13.6.3. Missing Date Information for Adverse Events....59
13.6.4. Missing Severity Assessment for Adverse Events....59
13.6.5. Missing Relationship to Investigational Product for Adverse Events....59
13.6.6. Character Values of Clinical Laboratory Variables....60

14. ANALYSIS SOFTWARE ....61
15. CHANGES TO ANALYSIS SPECIFIED IN PROTOCOL ....62
16. REFERENCES ....63
17. APPENDICES ....64
APPENDIX 1. SCHEDULE OF ASSESSMENTS ....65
APPENDIX 2. SELECT SAMPLE SAS® CODE ....69

## LIST OF TABLES

Table 1: Estimands and Attributes for Primary, Key Secondary, and Additional Secondary Endpoints....24
Table 2: Criteria for Potentially Clinically Significant Laboratory Tests ....44
Table 3: Criteria for Potentially Clinically Significant Vital Signs ....46
Table 4: Criteria for Potentially Clinically Significant ECGs....47
Table 5: Analysis Visit Windows for Unscheduled and Early Termination Visits for Assessments Done Mostly Every 4 Weeks....56

## LIST OF FIGURES

Figure 1: Study Design ....16

# ABBREVIATIONS

| | |
|---|---|
| ADA | anti-drug antibodies |
| AE | adverse event |
| AH50 | 50% alternative hemolytic complement pathway activity |
| ALP | alkaline phosphatase |
| ALT | alanine aminotransferase |
| ANCOVA | analysis of covariance |
| AR | autoregressive |
| ARH | heterogenous autoregressive |
| AST | aspartate aminotransferase |
| ATC | anatomical therapeutic class |
| BLQ | below limit of quantification |
| BMI | body mass index |
| C3G | C3 glomerulopathy |
| C3GN | C3 glomerulonephritis |
| CH50 | 50% classical hemolytic complement pathway activity |
| CI | confidence interval |
| CKD-EPI | Chronic Kidney Disease–Epidemiology Collaboration |
| COVID-19 | coronavirus disease 2019 |
| CRO | contract research organization |
| CS | compound symmetry |
| CV | coefficient of variation |
| DBP | diastolic blood pressure |
| DDD | dense deposit disease |
| DMC | data monitoring committee |
| ECG | electrocardiogram |
| eGFR | estimated glomerular filtration rate |
| EQ-5D-5L | 5-Level EuroQol-5 Dimension |
| FACIT | Functional Assessment of Chronic Illness Therapy |
| FMU | first-morning spot urine |
| GGT | gamma-glutamyltransferase |
| IC-MPGN | immune-complex membranoproliferative glomerulonephritis |
| ICE | intercurrent event |
| INR | international normalized ratio |
| ITT | intention-to-treat |
| KDQOL | kidney disease quality of life |
| LLN | lower limit of normal |
| LLOQ | lower limit of quantification |
| LS | least square |
| MAR | missing at random |
| MedDRA | Medical Dictionary for Regulatory Activities |

| | |
|---|---|
| MI | multiple imputation |
| mITT | modified intent-to-treat |
| MMRM | mixed-effect model for repeated measures |
| MNAR | missing not at random |
| OLP | open-label period |
| PCS | potentially clinically significant |
| PD | pharmacodynamic |
| PEG | polyethylene glycol |
| PGIC | patient global impression of change |
| PK | pharmacokinetic |
| PP | per-protocol |
| PT | Preferred Term |
| QTcF | QT interval corrected for heart rate using Fridericia's formula |
| RBC | red blood cell |
| RCP | randomized controlled period |
| SAE | serious adverse event |
| SAP | statistical analysis plan |
| SBP | systolic blood pressure |
| SC | subcutaneous |
| SD | standard deviation |
| SE | standard error |
| SOC | system organ class |
| TBL | total bilirubin |
| TEAE | treatment-emergent adverse event |
| ULN | upper limit of normal |
| uPCR | urine protein-to-creatinine ratio |
| WBC | white blood cell |
| WHO | World Health Organization |
| WPAI | work productivity and activity impairment |

# 1. INTRODUCTION

This statistical analysis plan (SAP) provides a technical and detailed elaboration of the statistical analyses of efficacy, safety, and pharmacokinetic/pharmacodynamic/immunogenicity data as described in the study protocol Amendment 3 dated 03 March 2023. Specifications for tables, figures, and listings are contained in a separate document.

The current SAP describes analysis of the randomized controlled period (RCP, up to week 26). Analysis on the interim data post-week 26 at the time of the datacut, referred to as the open-label period (OLP) reporting, will be provided in an SAP addendum.

# 2. OBJECTIVES AND ENDPOINTS

## 2.1. Objectives

### 2.1.1. Primary Objective

The primary objective of this study is to assess the efficacy of twice-weekly subcutaneous (SC) doses of pegcetacoplan compared with that of placebo in patients with primary C3 glomerulopathy (C3G) or immune-complex membranoproliferative glomerulonephritis (IC-MPGN) on the basis of a reduction in proteinuria.

### 2.1.2. Secondary Objectives

The secondary objectives of the study are as follows:

- To assess the effect of pegcetacoplan on estimated glomerular filtration rate (eGFR)
- To assess the effect of pegcetacoplan on additional C3G/IC-MPGN disease–related parameters
- To evaluate the safety of pegcetacoplan over 52 weeks of treatment

### 2.1.3. Exploratory Objectives

The exploratory objectives are to characterize the additional clinical, laboratory, and histologic findings of C3G/IC-MPGN in response to pegcetacoplan.

## 2.2. Endpoints

### 2.2.1. Primary Efficacy Endpoint

The primary efficacy endpoint is the log-transformed ratio of urine protein-to-creatinine ratio (uPCR) at week 26 compared to baseline.

### 2.2.2. Key Secondary Efficacy Endpoints

The key secondary efficacy endpoints are (to be evaluated at week 26):

- The proportion of participants who meet the criteria for achieving a composite renal endpoint (a stable or improved eGFR compared to the baseline visit (≤15% reduction in eGFR), and a ≥50% reduction in uPCR compared to the baseline visit.)
- The proportion of participants with a reduction of at least 50% from baseline in uPCR
- Change from baseline in eGFR
- For participants with evaluable renal biopsies, the change from baseline in the activity score of the C3G histologic index score
- The proportion of participants with evaluable renal biopsies showing decreases in C3c staining on renal biopsy from baseline

### 2.2.3. Additional Secondary Efficacy Endpoints

The additional secondary endpoints are (to be evaluated at week 26):

- The proportion of participants achieving proteinuria <1 g/day
- For participants with serum albumin levels below the lower limit of normal (LLN) at baseline, the proportion of participants with normalization of serum albumin levels
- For participants with serum C3 levels below the LLN at baseline, the proportion of participants with serum C3 levels above the LLN
- The change from baseline in Functional Assessment of Chronic Illness Therapy (FACIT)–Fatigue Scale score
- The change from baseline in Kidney Disease Quality of Life (KDQOL) score

### 2.2.4. Exploratory Efficacy Endpoints

The primary, key secondary, and additional secondary efficacy endpoints will also be evaluated at week 52 as exploratory efficacy endpoints.

In addition, the following exploratory efficacy endpoints will be evaluated:

- The change from baseline in uPCR using the 24-hour urine collections at week 24 and week 48
- The annual rate of change from up to 3 years prior to screening in eGFR at week 26 and week 52
- The proportion of participants with reductions from baseline in proteinuria of at least 30% at week 26 and week 52
- The proportion of participants with normalization of proteinuria at week 26 and week 52
- For participants with abnormal hematuria results at baseline, the number and proportion of participants with normalization of hematuria at week 26 and week 52
- Mean time to 50% reduction in uPCR with a stable or improved eGFR
- The time to normalization for the following parameters for participants in whom the parameter is abnormal at baseline:
  - serum C3
  - uPCR
  - serum albumin
  - hematuria
  - blood pressure
- The change from baseline in glomerular macrophage count, as determined by CD68 staining at week 26 and week 52

- The change from baseline in Patient Global Impression of Change (PGIC) score at week 26 and week 52
- The change from baseline in 5-Level EuroQol-5 Dimension (EQ-5D-5L) score at week 26 and week 52
- The change from baseline in Work Productivity and Activity Impairment (WPAI) score at week 26 and week 52
- Change in drusen from baseline at week 26 and week 52:
  - change in maximum drusen size
  - change in number of intermediate or large drusen

### 2.2.5. Safety Endpoints

The safety endpoints are as follows:

- The incidence and severity of treatment-emergent adverse events (TEAEs)
- The change from baseline in:
  - vital signs measurements
  - clinical laboratory tests
  - Electrocardiogram (ECG) results
- The number and incidence of rejection episodes (posttransplant participants only)
- The number and incidence of graft loss (posttransplant participants only)
- The incidence of death, stratified by transplant history

### 2.2.6. Pharmacokinetic Endpoint

The pharmacokinetic (PK) endpoint is pegcetacoplan serum concentrations over time.

### 2.2.7. Pharmacodynamic Endpoints

The pharmacodynamic (PD) endpoints are:

- Changes from baseline in complement levels at week 26 and week 52:
  - CH50 (50% classical hemolytic complement pathway activity)
  - AH50 (50% alternative hemolytic complement pathway activity)
  - sC5b-9

Additional complement components may be measured, as noted in Protocol Table 4 and Protocol Section 11.1.11, and evaluated as exploratory endpoints.

### 2.2.8. Immunogenicity Endpoint

The immunogenicity endpoint is:

- The incidence of antidrug antibodies (ie, antibodies against the peptide and polyethylene glycol [PEG] domains of pegcetacoplan)

# 3. STUDY DESIGN

## 3.1. General Description

This phase 3 randomized, placebo-controlled, double-blinded, multicenter clinical study is designed to evaluate the safety and efficacy of twice-weekly SC infusions of pegcetacoplan in patients with primary C3G or IC-MPGN. There will be approximately 80 to 100 participants enrolled in this study, at least 78 of whom will have native kidney disease and up to 22 of whom may have posttransplant recurrence of C3G or IC-MPGN. At least 63 participants with C3G in native kidney will be enrolled. The enrollment of participants with C3G or IC-MPGN will be monitored to ensure balance between the groups. At least 10 adolescent participants (aged 12-17 years) will be enrolled; adolescent participants may be either patients with native kidney disease or posttransplant disease recurrence. Participants initially screened as adolescents will follow adolescent procedures and requirements through the duration of their participation in study, even if they pass their 18th birthday while enrolled in the study.

The planned length of participation in the study for each participant is a maximum of approximately 70 weeks. This study will consist of 4 parts:

- Part 1: 10-week screening period
- Part 2: 26-week randomized controlled period (RCP)
- Part 3: 26-week open-label period
- Part 4: 8-week follow-up period (only for participants who do not roll into a long-term extension study)

Informed consent (and assent, when applicable) will be obtained prior to any study-related procedures being conducted. The screening period will start once informed consent has been obtained.

See Figure 1 for the study schematic, and Appendix 1 for the schedule of activities.

**Figure 1: Study Design**

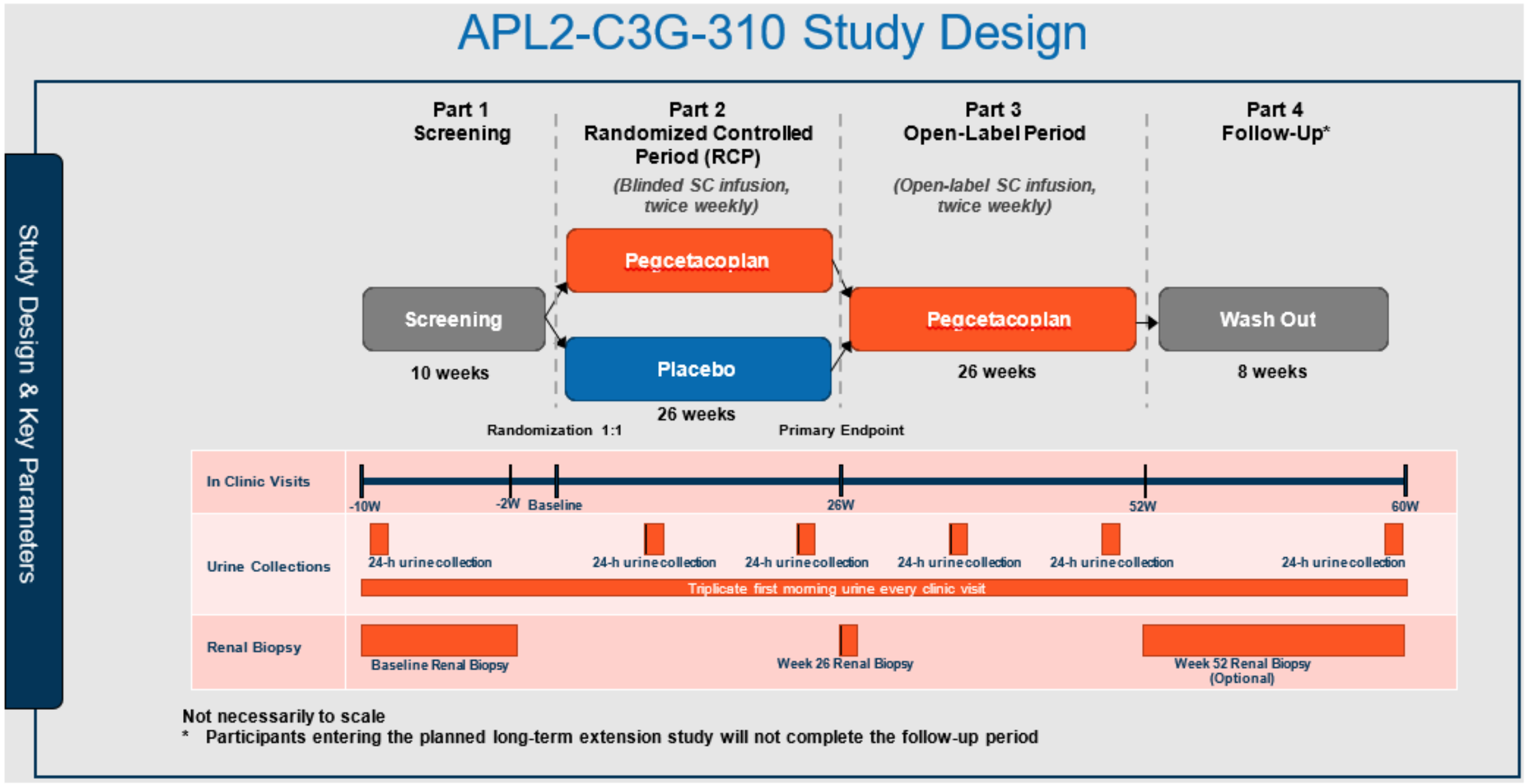


Abbreviation: SC = subcutaneous; uPCR = urine protein-to-creatinine ratio; W = week.

## 3.2. Randomization

Participants will be randomized to receive pegcetacoplan or placebo in a ratio of 1:1 via stratified permuted block randomization. The randomization will be performed centrally. To achieve balance between the arms, 2 stratification factors will be applied to the randomization. The first stratification factor examines participants with posttransplant recurrence versus nontransplant participants; at least 78 participants with C3G or IC-MPGN in a native kidney will be enrolled. This is followed by the second stratification factor, which examines participants with baseline renal biopsies (either collected during screening or a historic biopsy collected within 28 weeks prior to randomization) versus participants without baseline renal biopsies.

## 3.3. Blinding

This is a double-blinded study during the randomized controlled period. The open-label period is not blinded.

Designated blinded study staff (eg, research coordinators, nurses, technicians administering questionnaires, participants, central pathologists, assigned evaluating physicians, and the sponsor) will be blinded to treatment assignment. Access to unblinded study treatment information will be strictly limited as mandated in the study's Blinding Plan; any individuals who are unblinded are not allowed to discuss treatment and/or participant outcome with blinded study staff, including the evaluating physician. The principal investigator must be blinded to participants' treatment assignment. Although treatment in the open-label period is not blinded, investigators, study site personnel, and participants will remain blinded to the RCP treatment assignment until the blind has been broken.

## 3.4. Sample Size

Approximately 80 to 100 participants, including patients with native kidney disease or post-transplant, will be randomized 1:1 to pegcetacoplan or placebo with 40 to 50 participants per arm.

Based on preliminary data from Study APL2-201, a reduction of 60% in uPCR in the pegcetacoplan group at week 26 is assumed vs a reduction of 20% in uPCR in the placebo arm, which corresponds to mean log ratio to baseline of -0.92 vs -0.22 respectively, and a standard deviation of 0.88 (on log-scale). Based on this assumption, a sample size of 70 participants in total provides at least 90% power at 1-sided significance level of 0.025. Considering a 10% attrition to account for potential missing assessments and impact by coronavirus disease 2019 (COVID-19), it is expected that at least 78 participants with native kidney disease should be enrolled.

A minimum of 63 participants with C3G in native kidneys will be enrolled, which is approximately 80% of the enrolled participants with native kidney disease.

## 3.5. Analysis Timing and Unblinding

The analysis of data from the RCP of the study will be performed when all participants have completed the RCP or discontinued early and all corresponding data have been entered into the database, reviewed, cleaned, and finalized, and the week 26 Analysis database locked. At that time, the sponsor analysis team will be unblinded to the treatment code, and the primary analysis will be performed, which will include all efficacy and safety analyses for the RCP.

# 4. STATISTICAL ANALYSIS SETS

## 4.1. Screened Set

The screened set includes all participants who provide written informed consent. This set will be used only for the purpose of describing participant disposition.

## 4.2. Intent-to-Treat Set

The intent-to-treat (ITT) set includes all participants who are randomized. Participants will be analyzed in the treatment arm assigned at randomization.

## 4.3. Modified Intent-to-Treat Set

The modified intent-to-treat (mITT) set includes all randomized participants who receive at least one dose of pegcetacoplan or placebo and have baseline and at least one post-baseline value of triplicate first-morning spot urine (FMU) uPCR. Participants will be analyzed in the treatment arm assigned at randomization.

## 4.4. Safety Set

The safety set includes all participants who receive at least one dose of pegcetacoplan or placebo. Participants will be analyzed according to the actual treatment received. This population will be used for all safety analyses.

## 4.5. Per-Protocol Set

The per-protocol (PP) set includes all participants in the ITT set who have not violated any inclusion or exclusion criteria and/or deviated from the protocol in a way that could influence their efficacy assessment. Decisions concerning the exclusion of participants from the PP set will be made and documented prior to week 26 Analysis database lock.

The review and classification of protocol deviations is described in Section 5.7 and the study's Protocol Deviation Handling Plan, where the criteria for major and minor deviations are also defined. Deviations that affect exclusion from the PP set are a subset of protocol deviations; major protocol deviations do not necessarily result in exclusion of the participant from the PP set.

## 4.6. Pharmacokinetic Set

The pharmacokinetic (PK) set includes all participants in safety set who have at least one quantifiable post-dose concentration of pegcetacoplan (even with values below the limit of quantification [BLQ]).

## 4.7. Pharmacodynamic Set

The pharmacodynamic (PD) set includes all participants in the safety set who have at least one quantifiable post-dose PD endpoint (eg, C3, CH50, or AH50) evaluated.

# 5. STUDY PARTICIPANTS

## 5.1. Disposition of Participants

For summary of analysis sets, the number and percentage of participants screened, who failed screening, and who were included in each of the analysis sets as specified in Section 4 will be summarized and listed using the Screened Set.

For summary of disposition, the number and percentage of participants who were treated, who were ongoing with study treatment, who completed study treatment, who discontinued study treatment with a primary reason for discontinuation, who were still in the study, who completed the study, who withdrew from the study with a primary reason for withdrawal will be summarized and listed using the ITT set.

Participant disposition by region will also be provided for the ITT set.

## 5.2. Demographic and Other Baseline Characteristics

The following baseline demographic characteristics will be summarized and listed using the ITT, mITT, and Safety sets (if these three sets are different, otherwise will only generate for ITT): age at screening, age group (≤ 65 years vs > 65 years, alternative grouping based on age distribution may be considered if necessary), sex, ethnicity, race, weight, height, body mass index (BMI), and blood pressure.

The following baseline disease characteristics will be summarized and listed using the ITT, mITT, and Safety sets (if these three sets are different, otherwise will only generate for ITT): underlying disease based on screening biopsy (C3G including C3GN and DDD, or IC-MPGN), indication per disease specific medical history form, potential risk factors of C3G/IC-MPGN, disease manifestations, drusen, prior kidney transplant (Y/N), time since last kidney transplant, total number of kidney transplants, prior dialysis (Y/N), baseline 24h uPCR, baseline uPCR (triplicate first-morning spot urine), baseline eGFR, baseline creatinine, baseline serum albumin, baseline serum C3, time since initial diagnosis of C3G/IC-MPGN, time since most recent post-transplant recurrence, and time from last kidney transplant to the most recent post-transplant recurrence.

Time since initial diagnosis of C3G/IC-MPGN will be calculated as:

Time since initial diagnosis of C3G/IC-MPGN (years) = (day 1 date – start date of indication)/365.25.

Time since last kidney transplant will be calculated as:

Time since last kidney transplant (years) = (day 1 date – date of last kidney transplant)/365.25.

Time since most recent post-transplant recurrence will be calculated as:

Time since most recent post-transplant recurrence (years) = (day 1 date – date of most recent post-transplant recurrence)/365.25.

Time from last kidney transplant to the most recent post-transplant recurrence will be calculated as:

Time from last kidney transplant to the most recent post-transplant recurrence (years) = (date of most recent post-transplant recurrence – date of last kidney transplant)/365.25.

Note in the above time since calculations, day 1 date will be replaced with randomization date if participants are not dosed.

The following baseline biopsy characteristics will be summarized and listed using the ITT, mITT, and Safety sets (if these three sets are different, otherwise will only generate for ITT): C3 staining, number of glomeruli, light microscopic patterns, glomerular crescents, global sclerosis, interstitial fibrosis, tubular atrophy, deposits by electron microscopy (absent/present, characters of deposits, locations of deposits, degree of foot process effacement), total activity score, total chronicity score, and Banff score.

For baseline demographic characteristics and baseline disease characteristics, additional subgroup tables will be provided separately for adults vs adolescents.

For baseline demographic characteristics, baseline disease characteristics and baseline biopsy characteristics, additional subgroup tables will be provided separately for C3G vs IC-MPGN.

## 5.3. Medical History

Medical history will be coded using the latest Medical Dictionary for Regulatory Activities (MedDRA) coding dictionary. For participants in the Safety Set, medical history will be summarized by System Organ Class (SOC) and Preferred Term (PT) and listed. Each participant will be counted only once in each SOC or SOC/PT summary.

In the summary table, medical history will be presented by decreasing frequency of participants overall within each SOC and then similarly by decreasing frequency of participants overall within each PT. In the cases of SOCs or PTs with equal frequencies, medical history will be sorted alphabetically.

Vaccination will be summarized for the Safety Set and listed. Kidney transplant history will be listed for the Safety Set.

## 5.4. Prior and Concomitant Medications

Prior and concomitant medications will be coded using the latest World Health Organization (WHO) Drug Dictionary version available. Summaries of prior and concomitant medications will be presented by Anatomical Therapeutic Class (ATC) level 2 (therapeutic main group) and preferred term with numbers and percentages by treatment groups and overall for participants in the Safety Set. A participant who takes more than one medication will be counted only once if these medications belong to the same ATC level 2 classification.

In the summary tables, prior medications and concomitant medications will be presented by decreasing frequency of participants overall within each ATC level 2 class and then similarly by decreasing frequency of participants overall within each preferred term. In cases of ATC level 2 classes or preferred terms with equal frequencies, medications will be sorted alphabetically.

Prior medications are defined as those medications that started prior to the first administration of study drug. Concomitant medications are defined as those medications taken on/after the date of first administration of study drug. Medications started before the first dose of study drug and

continuing after will be considered as both prior and concomitant medications. A data listing of prior and concomitant medications will be provided for the Safety Set.

## 5.5. Prior and Concomitant Procedures

Prior and concomitant procedures will be coded using the latest MedDRA version available. Summaries of prior and concomitant procedures will be presented by SOC and PT with numbers and percentages by treatment groups and overall for participants in the Safety Set. Prior procedures are defined as those that started prior to the first administration of study drug. Concomitant procedures are defined as those that started on/after the date of first administration of study drug. Procedures that started before the first dose of study drug and continuing after will be considered as both prior and concomitant procedures. A data listing of all procedures will be provided for the Safety Set.

## 5.6. Exposure to Investigational Product

The following parameters will be summarized using the Safety Set and listed:

- Total dose administered (mg)
- Duration of treatment (days), defined as (last infusion date – first infusion date + 1)
- Number and percentage of participants who received at least one infusion
- Number and percentage of participants with any infusions missed
- Number and percentage of participants with one or more incomplete infusions
- Number and percentage of participants with any infusions interrupted

## 5.7. Measurements of Treatment Compliance

Percent compliance will be summarized using the Safety Set and listed. Compliance is calculated as follows: Compliance (%) = total number of study infusions taken / total number of expected infusions, multiplied by 100.

The number and percentage of participants who had a percentage of drug compliance range by increment of 10% (<80%, ≥ 80% - < 90%, ≥ 90 - ≤100%, and >100%) will also be summarized.

## 5.8. Protocol Deviations

All protocol deviations will be reviewed and documented before database lock. Protocol deviations are being captured in accordance with the protocol deviation management plan. They may also be identified through programmable checks of the data.

The CRO/Apellis will classify major and minor protocol deviations per the agreed Protocol Deviation Handling Plan. The Apellis study team will review the protocol deviations and their classification throughout the study and before database lock.

The number and percentage of participants with protocol deviations will be summarized by importance of deviation for ITT Set and listed.

# 6. EFFICACY ANALYSIS

Efficacy analysis including primary, key secondary, secondary, and exploratory analysis will be performed primarily using the mITT set and ITT set, with participants grouped according to the treatment assigned at randomization. Available data from all randomized participants regardless of adherence to the protocol will be included in the efficacy analysis, this includes data from participants who discontinued study drug early but continued with study assessments. All efficacy data will be listed for the ITT set.

All statistical tests will be performed at 2-sided 5% level of significance and all confidence intervals will be two-sided 95% confidence intervals.

Unless otherwise noted, the following randomization stratification factors will be adjusted for in analysis of efficacy endpoints in the overall population:

- Participants with posttransplant recurrence versus nontransplant participants
- Participants with baseline renal biopsies versus participants without baseline renal biopsies

If participants are found to have had the incorrect stratum assigned at randomization, they will be analyzed according to the randomization stratum.

## 6.1. Analysis Models

In general, data will be analyzed with the approaches described below.

*Mixed-effect model for repeated measures (MMRM) for continuous outcomes*

Longitudinal assessments for changes from baseline in continuous outcomes will be analyzed using a mixed-effect model for repeated measures (MMRM). The model will include fixed categorical effects for treatment group, visit, disease type (C3G vs. IC-MPGN), stratification factors, and the visit-by-treatment group interaction, as well as the continuous, fixed covariate of the baseline value of the endpoint. The least square (LS) means with standard errors (SEs) and 95% confidence intervals (CIs) of the change from baseline will be presented by treatment group and visit; between-treatment differences and 95% CIs and p-values will be presented by visit. LS means (±SE) will be plotted over time by treatment group.

A common unstructured covariance matrix will be used. If the model fails to converge, the following covariance structures will be fit in this order until convergence is met: (*i*) heterogenous autoregressive ARH(1), (*ii*) autoregressive AR(1), (*iii*) compound symmetry CS. The Kenward-Roger approximation will be used to estimate denominator degrees of freedom. The sandwich estimator (Diggle et al. 1994) will be used to estimate the standard errors of the fixed effects parameters for covariance structures other than the unstructured.

*Logistic regression model for binary outcomes*

Binary outcomes will be analyzed using a logistic regression model. The model will include treatment group as independent variable and adjusted for baseline value of the variable(s) used to define the endpoint, disease type, and stratification factors. Point estimates for proportions and difference in proportions will be presented. The p-value and odds ratio of being a responder for the pegcetacoplan group to being a responder for the placebo group with associated 95% CI will

be provided. In situations where event rates are low leading to convergence issue for the logistic regression model, alternative methods such as exact or penalized logistic regressions may be considered when appropriate.

*Analysis of covariance (ANCOVA)*

Analysis of covariance (ANCOVA) will be used to analyze C3G histologic index activity score, FACIT-Fatigue score, and KDQOL score with treatment as a fixed effect, adjusted for baseline score of the endpoint, disease type, and stratification factors. LS means will be presented for each treatment group, along with the between-treatment difference and 95% CI.

*Mixed effects model for slope analysis of continuous outcomes*

For slope analysis of continuous outcomes, a mixed effects model using the baseline and all postbaseline assessments will be used and will include treatment group, disease type, and stratification factors as fixed effects, time (study week, continuous assuming linearity), and the time-by-treatment interaction. A common unstructured covariance matrix will be used. If the model fails to converge, the following covariance structures will be fit in this order until convergence is met: (*i*) heterogenous autoregressive ARH(1), (*ii*) autoregressive AR(1), (*iii*) compound symmetry CS. The Kenward-Roger approximation will be used to estimate denominator degrees of freedom. The sandwich estimator (Diggle et al. 1994) will be used to estimate the standard errors of the fixed effects parameters for covariance structures other than the unstructured.

## 6.2. Multiplicity Adjustment

The primary endpoint of the study will be tested at the 2-sided 0.05 level, and if the null hypothesis for the primary endpoint is rejected, the secondary endpoints will be tested. The key secondary and additional secondary endpoints will be tested sequentially in the order in which they are presented in Section 2.2.2 and Section 2.2.3; the testing will stop once a null hypothesis is not rejected. This fixed-sequence testing procedure will ensure that trial-wise error rate is controlled to be 0.05.

## 6.3. Estimands

The primary objective of this study is to assess the efficacy of twice-weekly SC doses of pegcetacoplan compared with that of placebo in patients with primary C3G or IC-MPGN on the basis of a reduction in proteinuria.

The estimands and their attributes for the primary and all comparative key secondary and additional secondary endpoints are shown in Table 1 below. This includes strategies for addressing the following intercurrent events (ICEs):

- ProhibiRescue = use of prohibited concomitant medication specified in Protocol Section 8.3.3, or use of rescue therapies defined in Protocol Section 8.3.2
- RenalReplace = start renal replacement therapy (dialysis and/or renal transplant)
- DisctTrt = permanent discontinuation of study treatment

### Table 1: Estimands and Attributes for Primary, Key Secondary, and Additional Secondary Endpoints

For all estimands:

**A. Population**: participants with C3G or IC-MPGN defined through the study inclusion/exclusion criteria in the mITT Set

**B. Treatment regimens of interest**:

- Twice-weekly SC doses of pegcetacoplan for 26 weeks of treatment
- Twice-weekly SC doses of placebo for 26 weeks of treatment

| **C: Variable (or endpoint)** | **D: Strategies for addressing ICEs (event†: strategy‡)** | **E: Population-level summary** |
|---|---|---|
| **Primary Estimand** | | |
| Log-transformed ratio of uPCR at week 26 compared to baseline | ProhibiRescue: hypothetical strategy<br>RenalReplace: composite strategy<br>DisctTrt: treatment policy strategy | Difference in mean change of log-transformed uPCR from baseline to week 26 (measured by equal-weighted average over weeks 24, 25, and 26) between the pegcetacoplan group and the placebo group. |
| **Key Secondary Estimands (for comparative endpoints)** | | |
| The proportion of participants who meet the criteria for achieving a composite renal endpoint at week 26 | ProhibiRescue, RenalReplace, DisctTrt: composite strategy | Odds ratio of achieving a composite renal endpoint for the pegcetacoplan group to achieving a composite renal endpoint for the placebo group at week 26. |
| The proportion of participants with a reduction of at least 50% from baseline in uPCR at week 26 | ProhibiRescue, RenalReplace, DisctTrt: composite strategy | Odds ratio of achieving a reduction of at least 50% from baseline in uPCR for the pegcetacoplan group to achieving a reduction of at least 50% from baseline in uPCR for the placebo group at week 26. |
| Change from baseline in eGFR at week 26 | ProhibiRescue: hypothetical strategy<br>RenalReplace: composite strategy<br>DisctTrt: treatment policy strategy | Difference in mean change of eGFR from baseline to week 26 between the pegcetacoplan group and the placebo group. |
| For participants with evaluable renal biopsies, the change from baseline in the activity score of the C3G histologic index score at week 26 | ProhibiRescue: hypothetical strategy<br>RenalReplace: composite strategy<br>DisctTrt: treatment policy strategy | Difference in mean activity score between the pegcetacoplan group and the placebo group. |
| The proportion of participants with evaluable renal biopsies showing decreases in C3c staining on renal biopsy from baseline at week 26 | ProhibiRescue, RenalReplace, DisctTrt: composite strategy | Odds ratio of showing decreases in C3c staining for the pegcetacoplan group to showing decreases in C3c staining for the placebo group at week 26. |
| **Additional Secondary Estimands (for comparative endpoints)** | | |

**Table 1: Estimands and Attributes for Primary, Key Secondary, and Additional Secondary Endpoints**

| | | |
|---|---|---|
| The proportion of participants achieving proteinuria <1 g/day at week 24 | ProhibiRescue, RenalReplace, DisctTrt: composite strategy | Odds ratio of achieving proteinuria <1 g/day for the pegcetacoplan group to achieving proteinuria <1 g/day for the placebo group at week 24. |
| For participants with serum albumin levels below LLN at baseline, the proportion of participants with normalization of serum albumin levels at week 26 | ProhibiRescue, RenalReplace, DisctTrt: composite strategy | Odds ratio of achieving normalization of serum albumin for the pegcetacoplan group to achieving normalization of serum albumin for the placebo group at week 26. |
| For participants with serum C3 levels below the LLN at baseline, the proportion of participants with serum C3 levels above the LLN at week 26 | ProhibiRescue, RenalReplace, DisctTrt: composite strategy | Odds ratio of achieving serum C3 levels above the LLN for the pegcetacoplan group to achieving serum C3 levels above the LLN for the placebo group at week 26. |
| The change from baseline in FACIT–Fatigue Scale score | ProhibiRescue: hypothetical strategy<br>RenalReplace: composite strategy<br>DisctTrt: treatment policy strategy | Difference in mean FACIT–Fatigue Scale score between the pegcetacoplan group and the placebo group. |
| The change from baseline in KDQOL score | ProhibiRescue: hypothetical strategy<br>RenalReplace: composite strategy<br>DisctTrt: treatment policy strategy | Difference in mean KDQOL score between the pegcetacoplan group and the placebo group. |

†ICE definitions:

- ProhibiRescue = use of prohibited concomitant medication specified in Protocol Section 8.3.3, or use of rescue therapies defined in Protocol Section 8.3.2
- RenalReplace = start renal replacement therapy (dialysis and/or renal transplant)
- DisctTrt = permanent discontinuation of study treatment

‡Strategies**:**

- Composite strategy: (for continuous endpoints) all measurements after the ICEs will be set to missing. Missing data resulting from the ICEs will be imputed based on the worst change of all participants across visits plus a random error. (for binary endpoints) the endpoint status at or after the initiation of the ICEs will be regarded as non-responder.
- Hypothetical strategy: all measurements after the ICEs will be set to missing. Missing data resulting from the ICEs will be imputed using reference-based imputation.
- Treatment policy strategy: all measurements after the ICEs will be used as is. Missing data resulting from the ICEs will be imputed using reference-based imputation.

## 6.4. Analyses of Primary Efficacy Endpoint

The primary efficacy endpoint is the log-transformed ratio of uPCR at week 26 compared to baseline. The null ($H_{1,0}$) and alternative ($H_{1,1}$) hypotheses for the primary efficacy analysis are:

$H_{1,0}$: There is no difference in log-transformed ratio of uPCR at week 26 compared to baseline between the pegcetacoplan and placebo treatment groups.

$H_{1,1}$: There is a difference in log-transformed ratio of uPCR at week 26 compared to baseline between the pegcetacoplan and placebo treatment groups.

uPCR values are expected to be highly skewed, so the values will be natural log transformed in analysis and presentation. For the primary efficacy endpoint, sensitivity analyses (Section 6.4.2), supplemental analyses (Section 6.4.3), and subgroup analyses (Section 6.4.4) will be performed.

### 6.4.1. Main Analysis of Primary Efficacy Endpoint

The primary endpoint will be analyzed in the mITT set with participants grouped according to the treatment assigned at randomization.

Baseline uPCR value will be calculated as the average of the uPCR measurements from at least 6 of the 9 first-morning spot urine (FMU) samples collected between the start of screening and day 1, inclusive. The uPCR values used to calculate baseline should include those from the samples collected on day −2, day −1, and before dosing on day 1. In situations where less than 6 samples were collected, the average of all collected samples will still be used for baseline derivation. Such participants will still be included in the ITT/mITT set when applicable but will be excluded from the per protocol (PP) set. For scheduled visits where 3 collections are expected for calculating averages, if there are missing collections, the average will be based on those available collections.

An MMRM model will be used to analyze the primary endpoint. The model will include fixed categorical effects for treatment group, visit, disease type (C3G vs IC-MPGN), stratification factors, and the visit-by-treatment group interactions, as well as the continuous, fixed covariate of baseline log-transformed uPCR. As the primary estimate, the difference between treatment groups using a composite contrast of equal-weighted average over weeks 24, 25, and 26 will be estimated with its 95% CI and corresponding p-value. Further details on the presentation of the results are described in Section 6.1 above. In addition, the geometric means and geometric mean ratios relative to baseline will also be presented after converting the log-transformed values back to the original unit.

ICEs strategy and handling are listed in Table 1. Imputation for the non-monotone missing pattern (ie, arbitrary missing pattern) will be performed prior to the multiple imputation for the monotone missing pattern (ie, where a missing uPCR measurement at a visit for a participant implies that uPCR measurements at all subsequent visits for that participant are missing).

For the non-monotone missing pattern, missing value(s) between two visits with uPCR measurements will be imputed using the MCMC method, which was first proposed by Li (1988) and Liu (1993) described the algorithm. Multiple imputation will then be carried out for monotone missing pattern. For ICEs due to renal replacement therapy (dialysis and/or renal transplant), missing data will be imputed based on the worst change of all participants across

visits plus a random error. For other ICEs, missing data will be imputed based on reference-based imputation:

- Participants with monotone missing data in the placebo arm will have missing data imputed based on the observed values in the placebo arm.
- For the active treatment arm, participants with monotone missing data due to prohibited medication, rescue therapies, or treatment discontinuation will have missing data imputed based on the placebo arm.
- For the active treatment arm, participants with monotone missing data that are not due to prohibited medication, rescue therapies, or treatment discontinuation will have missing data imputed based on the observed values in the active treatment arm.

The number and percent of participants without monotone missing data (separated further by non-monotone missing vs. complete data), the number of participants with monotone missing data and a breakdown of reasons will be reported.

The imputation method will be implemented in SAS using the three standard steps to generate inference from imputed data: imputation step, analysis step, and pooling step. The randomization seed to be used is 230815.

- The missing data are filled in 100 times to generate 100 complete datasets.
- The 100 complete datasets are analyzed by using the MMRM approach described above.
- The results from the 100 complete datasets are combined for the inference using Rubin's rule.

The observed values for uPCR will be summarized by treatment group and visit. Summaries will present the descriptive statistics for baseline, absolute values, change and percentage change from baseline data by visit.

The mean change from baseline in uPCR (±SE) will be plotted over time by treatment group.

All uPCR data will be listed for the ITT set.

### 6.4.2. Sensitivity Analyses of Primary Efficacy Endpoint

Sensitivity analyses will be performed to evaluate the robustness of the primary analysis results. Analyses will be performed for the overall population based on the outcome of the primary analyses including all participants in the mITT set.

The following sensitivity analysis will be performed, as appropriate, using the same statistical approach as the one used in the primary analysis:

- Imputation based on MAR: Participants with missing data, regardless of the reason being the ICEs or not, will be handled implicitly within the MMRM analysis under the assumption of missing at random (MAR).
- Tipping point analysis: Participants with a monotone missing data pattern due to the ICEs will have missing data after the last assessment explicitly imputed by multiple

imputation using a tipping point analysis method assuming missing not at random (MNAR).

The tipping point analysis method (a delta-adjusted stress testing approach) will be implemented for the active treatment arm under the MNAR assumption by searching for a tipping point that reverses the conclusion regarding positive treatment effect (O'Kelly and Ratitch 2014). The following strategy will be used:

- For the control arm, participants will have missing data imputed based on the observed values in the control arm.
- For the active treatment arm, participants with monotone missing data that are not due to the ICEs will have missing data imputed based on the observed values in the active treatment arm.
- For the active treatment arm, participants with monotone missing data due to the ICEs will have missing data imputed based on the available values (observed values and values imputed for non-monotone missing data) in the active treatment arm with a shift parameter added to the imputed values. Multiple imputation will be implemented with the shift parameter allocated to the missing data point(s) proportionally across timepoints. (eg, for a shift parameter of 0.26 at week 26, incremental shifts of 0.04 would apply to week 4, …, and week 24, respectively, then a shift of 0.01 would apply to week 25 and week 26, respectively. 0.04 × 6 visits+ 0.01 × 2 visits= 0.26) The range of the shift parameters will be from 0.13 to 0.65 by increments of 0.13 for week 26. A tipping point may not exist within reasonable clinical assumptions.

To generate inference from imputed data, the same three standard steps as described in the main analysis will be implemented.

### 6.4.3. Supplemental Analyses of Primary Efficacy Endpoint

#### 6.4.3.1. Per Protocol Set

The main analysis described in Section 6.4.1 will be repeated using the PP set to investigate the impact of changing the population in the estimand.

#### 6.4.3.2. Slope Analysis

The mean rate of change (ie, slope) in observed log-transformed uPCR values will be compared between treatment groups using the linear mixed effects model for slope analysis of continuous outcomes described above in Section 6.1. The mean rate of change (slope), standard errors, and confidence interval will be estimated for the baseline to week 26 period for each treatment group. In addition, the estimated difference in slopes between the treatment groups along with the 95% CIs and p-value will be reported. Strategies for addressing ICEs are the same as the ones described in Section 6.4.1.

### 6.4.4. Subgroup Analyses of Primary Efficacy Endpoint

Subgroup analyses will be performed to evaluate the consistency of the primary analysis results across subgroups defined by demographic and baseline characteristics. Analyses will be

performed for the primary efficacy endpoint for each of the following subgroups (as appropriate per actual subgroup sample size, levels with low sample size may be pooled to allow for an analysis to be conducted):

- Age group: adolescent, adult
- Sex: male, female
- Race: Caucasian, non-Caucasian
- Geographic region: United States, Rest of World
- Disease type: C3G, IC-MPGN
- Transplant history: nontransplant, posttransplant
- Baseline FMU uPCR: <3000 mg/g, ≥3000 mg/g
- Baseline eGFR: ≥30 - <60 mL/min/1.73 $m^2$, ≥60 mL/min/1.73 $m^2$
- Baseline C3 level: normal, below the LLN

The estimated treatment effects and corresponding 95% CIs and p-values from the models will be displayed graphically for each level of the subgroups specified (eg, via forest plots).

## 6.5. Analyses of Key Secondary Efficacy Endpoints

The following subsections describe the analyses of the key secondary efficacy endpoints of the study. All analyses of the key secondary efficacy endpoints will be done primarily using the mITT set and ITT set.

### 6.5.1. The Proportion of Participants Who Meet the Criteria for Achieving a Composite Renal Endpoint

The key secondary efficacy endpoint of the proportion of participants who meet the criteria for achieving a composite renal endpoint at week 26 will be examined with the following null ($H_{2,0}$) and alternative ($H_{2,1}$) hypotheses:

$H_{2,0}$: There is no difference in proportion of participants who meet the criteria for achieving a composite renal endpoint at week 26 between the pegcetacoplan and placebo treatment groups.

$H_{2,1}$: There is a difference in proportion of participants who meet the criteria for achieving a composite renal endpoint at week 26 between the pegcetacoplan and placebo treatment groups.

A participant meets the requirements of the composite renal endpoint if they satisfy:

(1) A stable or improved eGFR compared to baseline (≤15% reduction in eGFR), and
(2) A ≥50% reduction in uPCR compared to baseline.

For requirement (1), the eGFR status at week 26 will be calculated using the following steps:

1. Baseline eGFR value will be calculated using the last non-missing assessment prior to first dose.

2. Week 26 eGFR value will be calculated based on the week 26 assessment result.
3. Each participant will be categorized as either a “stable or improved” or not “stable or improved” according to whether the change from baseline in eGFR calculated from steps 1 and 2 is a reduction of no more than 15%.

For requirement (2), the uPCR response status at week 26 will be calculated using the following steps:

1. Baseline uPCR value will be calculated as the average of the uPCR measurements from at least 6 of the 9 FMU samples collected between the start of screening and day 1, inclusive.
2. Week 26 uPCR value will be calculated as the average of the uPCR measurements from at least 6 of the 9 FMU samples collected in week 24, week 25, and week 26.
3. Each participant will be categorized as either a success or a failure according to whether the change from baseline in uPCR calculated from steps 1 and 2 is a reduction of at least 50%.

#### 6.5.1.1. Main Analysis

The key secondary endpoint will be analyzed in the ITT set with participants grouped according to the treatment assigned at randomization.

A logistic model will be used to analyze the key secondary endpoint. The model will include treatment group as the independent variable and adjusted for baseline eGFR values, baseline log-transformed uPCR values, disease type (C3G vs IC-MPGN), and stratification factors. Further details on the presentation of the results are described in Section 6.1.

A composite strategy will be used for addressing the ICEs listed in Table 1. In the composite strategy, the composite renal endpoint status at or after the occurrence of any of these ICEs will be regarded as non-responder. Additionally, participants with missing eGFR and/or uPCR values at week 26 for reasons other than those listed as ICEs will be regarded as non-responder.

The numbers and proportion of participants who meet the criteria for achieving a composite renal endpoint at week 26 will be tabulated by treatment group, including detailed breakdown on each of the two requirements.

All composite renal endpoint achievement data will be listed for the ITT set.

#### 6.5.1.2. Supplemental Analyses

##### 6.5.1.2.1. Per Protocol Set

The main analysis described in Section 6.5.1.1 will be repeated using the PP set to investigate the impact of changing the population in the estimand.

#### 6.5.1.3. Subgroup Analyses

Subgroup analyses will be performed for this key secondary endpoint to evaluate the consistency of the results across the subgroups specified in Section 6.4.4 above.

### 6.5.2. The Proportion of Participants With a Reduction of at Least 50% from Baseline in uPCR

The key secondary efficacy endpoint of the proportion of participants with a reduction of at least 50% from baseline in uPCR at week 26 will be examined with the following null ($H_{3,0}$) and alternative ($H_{3,1}$) hypotheses:

$H_{3,0}$: There is no difference in proportion of participants who have a reduction of at least 50% from baseline in uPCR at week 26 between the pegcetacoplan and placebo treatment groups.

$H_{3,1}$: There is a difference in proportion of participants who have a reduction of at least 50% from baseline in uPCR at week 26 between the pegcetacoplan and placebo treatment groups.

The uPCR response status at week 26 will be calculated using the following steps:

1. Baseline uPCR value will be calculated as the average of the uPCR measurements from at least 6 of the 9 FMU samples collected between the start of screening and day 1, inclusive.
2. Week 26 uPCR value will be calculated as the average of the uPCR measurements from at least 6 of the 9 FMU samples collected in week 24, week 25, and week 26.
3. Each participant will be categorized as either a success or a failure according to whether the change from baseline in uPCR calculated from steps 1 and 2 is a reduction of at least 50%.

#### 6.5.2.1. Main Analysis

The key secondary endpoint will be analyzed in the ITT set with participants grouped according to the treatment assigned at randomization.

A logistic model will be used to analyze the key secondary endpoint. The model will include treatment group as the independent variable and adjusted for baseline log-transformed uPCR values, disease type (C3G vs IC-MPGN), and stratification factors. Further details on the presentation of the results are described in Section 6.1 above. Strategies for addressing ICEs are the same as the ones described in Section 6.5.1.1.

The numbers and proportion of participants with a reduction of at least 50% from baseline at week 26 will be tabulated by treatment group.

All the uPCR response status data will be listed for the ITT set.

#### 6.5.2.2. Supplemental Analyses

##### 6.5.2.2.1. Per Protocol Set

The main analysis described in Section 6.5.2.1 will be repeated using the PP set to investigate the impact of changing the population in the estimand.

#### 6.5.2.3. Subgroup Analyses

Subgroup analyses will be performed for this key secondary endpoint to evaluate the consistency of the results across the subgroups specified in Section 6.4.4 above.

### 6.5.3. Change From Baseline in eGFR

The key secondary efficacy endpoint of change from baseline in eGFR at week 26 will be examined with the following null ($H_{4,0}$) and alternative ($H_{4,1}$) hypotheses:

$H_{4,0}$: There is no difference in mean change from baseline to week 26 in eGFR between the pegcetacoplan and placebo treatment groups.

$H_{4,1}$: There is a difference in mean change from baseline to week 26 in eGFR between the pegcetacoplan and placebo treatment groups.

#### 6.5.3.1. Main Analysis

The key secondary endpoint will be analyzed in the mITT set with participants grouped according to the treatment assigned at randomization.

A MMRM model will be used to analyze the key secondary endpoint. The model will include fixed categorical effects for treatment group, visit, disease type (C3G vs IC-MPGN), stratification factors, and the visit-by-treatment group interactions, as well as the continuous, fixed covariate of baseline eGFR values. Further details on the presentation of the results are described in Section 6.1. Strategies for addressing ICEs are the same as the ones described in Section 6.4.1.

The observed values for eGFR will be summarized by treatment group and visit. Summaries will present the descriptive statistics for baseline, absolute values, change and percentage change from baseline data by visit.

The mean change from baseline in eGFR (±SE) will be plotted over time by treatment group.

All eGFR data will be listed for the ITT set.

#### 6.5.3.2. Sensitivity Analyses

The two sensitivity analyses method described in Section 6.4.2 will be repeated for the key secondary endpoint. For tipping point analysis, the range of the shift parameters will be from −1.3 to −6.5 by increments of −1.3.

#### 6.5.3.3. Supplemental Analyses

##### 6.5.3.3.1. Per Protocol Set

The main analysis described in Section 6.5.3.1 will be repeated using the PP set to investigate the impact of changing the population in the estimand.

##### 6.5.3.3.2. Slope Analysis

The mean rate of change (ie, slope) in observed eGFR values will be compared between treatment groups using the linear mixed effects model for slope analysis of continuous outcomes

described above in Section 6.1. The mean rate of change (slope), standard errors, and confidence interval will be estimated for the baseline to week 26 period for each treatment group. In addition, the estimated difference in slopes between the treatment groups along with the 95% CIs and p-value will be reported.

#### 6.5.3.4. Subgroup Analyses

Subgroup analyses will be performed for this key secondary endpoint to evaluate the consistency of the results across the subgroups specified in Section 6.4.4.

### 6.5.4. Change From Baseline in the Activity Score of the C3G Histologic Index Score

For participants with evaluable renal biopsies, the key secondary efficacy endpoint is the activity score of the C3G histologic index score at week 26. It will be examined with the following null ($H_{5,0}$) and alternative ($H_{5,1}$) hypotheses:

$H_{5,0}$: There is no difference in activity score between the pegcetacoplan and placebo treatment groups.

$H_{5,1}$: There is a difference in activity score between the pegcetacoplan and placebo treatment groups.

#### 6.5.4.1. Main Analysis

The key secondary endpoint will be analyzed in the ITT set with participants grouped according to the treatment assigned at randomization.

An ANCOVA model will be used to analyze the key secondary endpoint. The model will include treatment as a fixed effect, adjusted for baseline C3G histologic index activity score, disease type (C3G vs IC-MPGN), and stratification factors. Further details on the presentation of the results are described in Section 6.1. Strategies for addressing ICEs are the same as the ones described in Section 6.4.1.

The observed values for activity score will be summarized by treatment group and visit. Summaries will present the descriptive statistics for baseline, absolute values, change and percentage change from baseline data by visit. In addition, each of the 7 components of the activity score will also by summarized descriptively.

All activity score data will be listed for the ITT set.

#### 6.5.4.2. Sensitivity Analyses

The tipping point analysis method described in Section 6.4.2 will be repeated for the key secondary endpoint. The range of the shift parameters will be from 1.3 to 6.5 by increments of 1.3.

#### 6.5.4.3. Supplemental Analyses

##### 6.5.4.3.1. Per Protocol Set

The main analysis described in Section 6.5.4.1 will be repeated using the PP set to investigate the impact of changing the population in the estimand.

#### 6.5.4.4. Subgroup Analyses

Subgroup analyses will be performed for this key secondary endpoint to evaluate the consistency of the results across the subgroups specified in Section 6.4.4 above.

### 6.5.5. The Proportion of Participants Showing Decreases in C3c Staining From Baseline

For participants with evaluable renal biopsies, the key secondary efficacy endpoint of the proportion of participants showing decreases in C3c staining (defined as decrease of at least 2 orders of magnitude of intensity) from baseline at week 26 will be examined with the following null ($H_{6,0}$) and alternative ($H_{6,1}$) hypotheses:

$H_{6,0}$: There is no difference in proportion of participants who show decreases in C3c staining from baseline at week 26 between the pegcetacoplan and placebo treatment groups.

$H_{6,1}$: There is a difference in proportion of participants who show decreases in C3c staining from baseline at week 26 between the pegcetacoplan and placebo treatment groups.

#### 6.5.5.1. Main Analysis

The key secondary endpoint will be analyzed in the ITT set with participants grouped according to the treatment assigned at randomization.

A logistic model will be used to analyze the key secondary endpoint. The model will include treatment group as the independent variable and adjusted for baseline C3c staining, disease type (C3G vs IC-MPGN), and stratification factors. Further details on the presentation of the results are described in Section 6.1. Strategies for addressing ICEs are the same as the ones described in Section 6.5.1.1.

The numbers and proportion of participants who meet the criteria of showing decreases in C3c staining at week 26 will be tabulated by treatment group.

All C3c staining data will be listed for the ITT set.

#### 6.5.5.2. Supplemental Analyses

##### 6.5.5.2.1. Per Protocol Set

The main analysis described in Section 6.5.5.1 will be repeated using the PP set to investigate the impact of changing the population in the estimand.

#### 6.5.5.3. Subgroup Analyses

Subgroup analyses will be performed for this key secondary endpoint to evaluate the consistency of the results across the subgroups specified in Section 6.4.4.

## 6.6. Analyses of Additional Secondary Efficacy Endpoints

The following subsections describe the analyses of the additional secondary efficacy endpoints of the study. All analyses of the additional secondary efficacy endpoints will be done on the ITT set.

### 6.6.1. The Proportion of Participants Achieving Proteinuria <1 g/day

The additional secondary efficacy endpoint of the proportion of participants achieving proteinuria <1 g/day (assessed by 24-hour urine protein) at week 24 will be examined with the following null ($H_{7,0}$) and alternative ($H_{7,1}$) hypotheses:

$H_{7,0}$: There is no difference in proportion of participants who achieve proteinuria <1 g/day at week 24 between the pegcetacoplan and placebo treatment groups.

$H_{7,1}$: There is a difference in proportion of participants who achieve proteinuria <1 g/day at week 24 between the pegcetacoplan and placebo treatment groups.

The additional secondary endpoint will be analyzed in the ITT set with participants grouped according to the treatment assigned at randomization.

A logistic model will be used to analyze the additional secondary endpoint. The model will include treatment group as the independent variable and adjusted for baseline proteinuria values, disease type (C3G vs IC-MPGN), and stratification factors. Further details on the presentation of the results are described in Section 6.1. Strategies for addressing ICEs are the same as the ones described in Section 6.5.1.1.

The numbers and proportion of participants achieving proteinuria <1 g/day at week 24 will be tabulated by treatment group.

All 24-hour urine protein data will be listed for the ITT set.

### 6.6.2. The Proportion of Participants With Normalization of Serum Albumin Levels

For participants with serum albumin levels below LLN at baseline, the additional secondary efficacy endpoint of the proportion of participants with normalization of serum albumin levels at week 26 will be examined with the following null ($H_{8,0}$) and alternative ($H_{8,1}$) hypotheses:

$H_{8,0}$: There is no difference in proportion of participants who achieve normalization of serum albumin levels at week 26 between the pegcetacoplan and placebo treatment groups.

$H_{8,1}$: There is a difference in proportion of participants who achieve normalization of serum albumin levels at week 26 between the pegcetacoplan and placebo treatment groups.

The serum albumin levels status at week 26 will be calculated using the following steps:

1. Baseline serum albumin value will be calculated as the average of up to 2 serum albumin measurements preceding and including day 1. Participants will be included in the denominator of this calculation only if the baseline albumin value is below the LLN.

2. Week 26 serum albumin values will be calculated as the average of up to 2 serum albumin measurements preceding and including week 26, no earlier than week 20 measurement.
3. Each participant will be categorized as either a success or a failure according to whether the week 26 serum albumin values as calculated in Step 2 are above the LLN.

The additional secondary endpoint will be analyzed in the ITT set with participants grouped according to the treatment assigned at randomization.

A logistic model will be used to analyze the additional secondary endpoint. The model will include treatment group as the independent variable and adjusted for baseline albumin value, disease type (C3G vs IC-MPGN), and stratification factors. Further details on the presentation of the results are described in Section 6.1. Strategies for addressing ICEs are the same as the ones described in Section 6.5.1.1.

For participants with serum albumin levels below the LLN at baseline, the numbers and proportion of participants with normalization of serum albumin levels at week 26 will be tabulated by treatment group. The observed values for serum albumin will be summarized by treatment group and visit.

All serum albumin data will be listed for the ITT set.

### 6.6.3. The Proportion of Participants With Serum C3 Levels Above the LLN

For participants with serum C3 levels below the LLN at baseline, the additional secondary efficacy endpoint of the proportion of participants with serum C3 levels above the LLN at week 26 will be examined with the following null ($H_{9,0}$) and alternative ($H_{9,1}$) hypotheses:

$H_{9,0}$: There is no difference in proportion of participants with serum C3 levels above the LLN at week 26 between the pegcetacoplan and placebo treatment groups.

$H_{9,1}$: There is a difference in proportion of participants with serum C3 levels above the LLN at week 26 between the pegcetacoplan and placebo treatment groups.

The serum C3 levels status at week 26 will be calculated using the following steps:

1. Baseline serum C3 value will be calculated as the average of up to 2 serum C3 measurements preceding and including day 1. Participants will only be included in the denominator of this calculation if the baseline C3 value is below the LLN.
2. Week 26 serum C3 value will be calculated as the average of up to 2 serum C3 measurements preceding and including week 26, no earlier than week 20 measurement.
3. Each participant will be categorized as either a success or a failure based on whether the week 26 serum C3 value as calculated in Step 2 is above the lower limit of normal.

The additional secondary endpoint will be analyzed in the ITT set with participants grouped according to the treatment assigned at randomization.

A logistic model will be used to analyze the additional secondary endpoint. The model will include treatment group as the independent variable and adjusted for baseline serum C3 level, disease type (C3G vs IC-MPGN), and stratification factors. Further details on the presentation of

the results are described in Section 6.1. Strategies for addressing ICEs are the same as the ones described in Section 6.5.1.1.

For participants with low serum C3 levels at baseline, the numbers and proportion of participants with serum C3 levels above the LLN at week 26 will be tabulated by treatment group. The observed values for serum C3 will be summarized by treatment group and visit.

All serum C3 level data will be listed for the ITT set.

### 6.6.4. Change From Baseline in FACIT-Fatigue Scale Score

The additional secondary efficacy endpoint is the FACIT-Fatigue score at week 26. It will be examined with the following null ($H_{10,0}$) and alternative ($H_{10,1}$) hypotheses:

$H_{10,0}$: There is no difference in FACIT-Fatigue score between the pegcetacoplan and placebo treatment groups.

$H_{10,1}$: There is a difference in FACIT-Fatigue score between the pegcetacoplan and placebo treatment groups.

The additional secondary endpoint will be analyzed in the ITT set with participants grouped according to the treatment assigned at randomization.

An ANCOVA model will be used to analyze the additional secondary endpoint. The model will include treatment as a fixed effect, adjusted for baseline FACIT-Fatigue score, disease type (C3G vs IC-MPGN), and stratification factors. Further details on the presentation of the results are described in Section 6.1. Strategies for addressing ICEs are the same as the ones described in Section 6.4.1.

The observed values for FACIT-Fatigue score will be summarized by treatment group and visit. Summaries will present the descriptive statistics for baseline, absolute values, change and percentage change from baseline data by visit.

All FACIT-Fatigue score data will be listed for the ITT set.

### 6.6.5. Change From Baseline in KDQOL Score

The additional secondary efficacy endpoint is the KDQOL score at week 26. It will be examined with the following null ($H_{11,0}$) and alternative ($H_{11,1}$) hypotheses:

$H_{11,0}$: There is no difference in KDQOL score between the pegcetacoplan and placebo treatment groups.

$H_{11,1}$: There is a difference in KDQOL score between the pegcetacoplan and placebo treatment groups.

The additional secondary endpoint will be analyzed in the ITT set with participants grouped according to the treatment assigned at randomization.

The KDQOL score is constructed as the KDQOL-36 Summary Score (KSS) by averaging the 24 items from Burden of Kidney Disease, Symptoms and Problems of Kidney Disease, and Effects of Kidney Disease on 0-100 scale (Peipert et al. 2019).

An ANCOVA model will be used to analyze the additional secondary endpoint. The model will include treatment as a fixed effect, adjusted for baseline KDQOL score, disease type (C3G vs IC-MPGN), and stratification factors. Further details on the presentation of the results are described in Section 6.1. Strategies for addressing ICEs are the same as the ones described in Section 6.4.1.

The observed values for KDQOL score will be summarized by treatment group and visit. Summaries will present the descriptive statistics for baseline, absolute values, change and percentage change from baseline data by visit.

All KDQOL score data will be listed for the ITT set.

## 6.7. Analyses of Exploratory Efficacy Endpoints

All primary, key secondary, and additional secondary endpoints will be evaluated at week 52 as exploratory efficacy endpoints. In addition, the following exploratory endpoints will also be evaluated. Note that this SAP focuses on the analysis of the randomized controlled period (RCP) data only. The analysis for interim data post-week 26 at the time of the datacut will be detailed in an SAP addendum.

### 6.7.1. The Change From Baseline in uPCR Using the 24-Hour Urine Collections

The observed values for uPCR measured by 24-hour urine collections will be summarized by treatment group and visit using ITT Set. Summaries will present the descriptive statistics for baseline, absolute values, change and percentage change from baseline data by visit.

An MMRM model will be used to analyze the exploratory efficacy endpoint up to week 24. The model will include fixed categorical effects for treatment group, visit, disease type (C3G vs IC-MPGN), stratification factors, and the visit-by-treatment group interactions, as well as the continuous, fixed covariate of baseline 24-hour uPCR values. Further details on the presentation of the results are described in Section 6.1. No multiple imputations will be applied. ICE strategies will be simplified where missing data after the ICEs will be imputed implicitly within the MMRM in a hypothetical strategy under the assumption of MAR.

All 24-hour uPCR data will be listed for the ITT set.

### 6.7.2. The Annual Rate of Change From up to 3 Years Prior to Screening in eGFR

Based on the protocol, retrospective eGFR data will be collected for up to 3 years prior to study entry. For each participant, the annual rate of change (or slope) will be calculated using linear regression methods to obtain the historic slope, whereas the annualized slope during the RCP will be obtained as described in Section 6.5.3.3.2. The resulting differences between the historic slope vs RCP slope will be summarized by treatment group and visit using the mITT set.

In situations where for a certain historical collection, the eGFR value was not collected but the components for calculating it (such as serum creatinine, age, height, etc.) were collected, then the eGFR value will be calculated based on the formula specified in protocol.

All retrospective eGFR data will be listed for the ITT set.

### 6.7.3. The Proportion of Participants With Reductions From Baseline in Proteinuria of at Least 30%

The proportion of participants with reductions from baseline in proteinuria (measured by triplicate FMU uPCR) of at least 30% at week 26 will be summarized using ITT set. The baseline and week 26 uPCR calculation will be handled the same way as in Section 6.5.1.

A logistic model will be used to analyze the exploratory efficacy endpoint. The model will include treatment group as the independent variable and adjusted for baseline log-transformed uPCR values, disease type (C3G vs IC-MPGN), and stratification factors. Further details on the presentation of the results are described in Section 6.1. ICE strategies will be the same as those specified in Section 6.5.1.1.

A data listing for each participant's reduction status at week 26 will be provided using ITT set.

### 6.7.4. The Proportion of Participants With Normalization of Proteinuria

The proportion of participants with normalization of proteinuria (measured by triplicate FMU uPCR) at week 26 will be summarized using ITT set. The baseline and week 26 uPCR calculation will be handled the same way as in Section 6.5.1. Normalization of proteinuria is defined as test result values within the normal reference range.

A logistic model will be used to analyze the exploratory efficacy endpoint. The model will include treatment group as the independent variable and adjusted for baseline log-transformed uPCR values, disease type (C3G vs IC-MPGN), and stratification factors. Further details on the presentation of the results are described in Section 6.1. ICE strategies will be the same as those specified in Section 6.5.1.1.

A data listing for each participant's normalization status at week 26 be provided using ITT set.

### 6.7.5. The Proportion of Participants With Normalization of Hematuria

Based on protocol schedule of assessments, hematuria (measured by urine hemoglobin) collection is only scheduled during screening period. Therefore, this planned analysis is not applicable.

### 6.7.6. Mean Time to 50% Reduction in uPCR With a Stable or Improved eGFR

Time to 50% reduction in uPCR with a stable or improved eGFR (a.k.a., achieving the composite renal endpoint as defined in Section 6.5.1) will be summarized using ITT set with the Kaplan-Meier method (Kaplan and Meier 1958), using plots and presentation of median (and 95% CIs) for time-to-event.

A data listing for each participant's time to achieve 50% reduction in uPCR with a stable or improved eGFR will be provided using ITT set.

### 6.7.7. Time to Normalization for Selected Parameters for Participants in Whom the Parameter is Abnormal at Baseline

For parameters serum C3, uPCR (measured by triplicate FMU), serum albumin, and blood pressure (both SBP and DBP), time to normalization (in days) will be summarized using ITT set

with the Kaplan-Meier method, using plots and presentation of median (and 95% CIs) for time-to-event. The analyses will be based on participants whose test result being abnormal at baseline.

A data listing for each participant's time to normalization for these parameters will be provided using ITT set.

### 6.7.8. The Change From Baseline in Glomerular Macrophage Count

The observed values for glomerular macrophage count, determined by CD68 staining, will be summarized by treatment group and visit using ITT set. Summaries will present the descriptive statistics for baseline, absolute values, change and percentage change from baseline data by visit.

An ANCOVA model will be used to analyze the exploratory endpoint at week 26. The model will include treatment as a fixed effect, adjusted for baseline glomerular macrophage count, disease type (C3G vs IC-MPGN), and stratification factors. Further details on the presentation of the results are described in Section 6.1. No multiple imputations will be applied. ICE strategies will be simplified where participants with missing data at week 26 (whether due to ICE or not) will be excluded from the modeling.

All glomerular macrophage count results will be listed for the ITT set.

### 6.7.9. The Change From Baseline in PGIC Score

The observed values for PGIC score will be summarized by treatment group and visit using ITT set. Summaries will present the descriptive statistics for baseline, absolute values, change and percentage change from baseline data by visit.

An ANCOVA model will be used to analyze the exploratory endpoint at week 26. The model will include treatment as a fixed effect, adjusted for baseline PGIC score, disease type (C3G vs IC-MPGN), and stratification factors. Further details on the presentation of the results are described in Section 6.1. No multiple imputations will be applied. ICE strategies will be simplified where participants with missing data at week 26 (whether due to ICE or not) will be excluded from the modeling.

All PGIC score results will be listed for the ITT set.

### 6.7.10. The Change From Baseline in EQ-5D-5L Score

The observed values for EQ-5D-5L score will be summarized by treatment group and visit using ITT set. Summaries will present the descriptive statistics for baseline, absolute values, change and percentage change from baseline data by visit.

An ANCOVA model will be used to analyze the exploratory endpoint. The model will include treatment as a fixed effect, adjusted for baseline EQ-5D-5L score, disease type (C3G vs IC-MPGN), and stratification factors. Further details on the presentation of the results are described in Section 6.1. No multiple imputations will be applied. ICE strategies will be simplified where participants with missing data at week 26 (whether due to ICE or not) will be excluded from the modeling.

All EQ-5D-5L score results will be listed for the ITT set.

### 6.7.11. The Change From Baseline in WPAI Score

The observed values for WPAI score will be summarized by treatment group and visit using ITT set. Summaries will present the descriptive statistics for baseline, absolute values, change and percentage change from baseline data by visit.

An ANCOVA model will be used to analyze the exploratory endpoint. The model will include treatment as a fixed effect, adjusted for baseline WPAI score, disease type (C3G vs IC-MPGN), and stratification factors. Further details on the presentation of the results are described in Section 6.1. No multiple imputations will be applied. ICE strategies will be simplified where participants with missing data at week 26 (whether due to ICE or not) will be excluded from the modeling.

All WPAI score results will be listed for the ITT set.

### 6.7.12. Change in Drusen From Baseline

Based on protocol schedule of assessments, ophthalmologic evaluations are only scheduled at screening and week 42 hence the endpoint will not be part of this RCP analysis.

### 6.7.13. Correlation Analysis on Reduction in uPCR and Change in eGFR

An exploratory correlation analysis exploring the relationship between the reduction in uPCR and change in eGFR will be considered at week 26, with correlation coefficients provided. The analysis may be repeated based on alternative populations (such as ITT, mITT, or subgroups based on baseline level) or uPCR reduction metrics (such as change from baseline, percentage change from baseline, or absolute levels) if needed.

# 7. SAFETY ANALYSIS

The safety analysis will be performed using the Safety Set. Safety variables include AEs, clinical laboratory data, vital signs, and ECGs. For each safety variable, the last value collected before the first dose of investigational product will be used as baseline for all analyses of that safety variable.

All safety analyses will be conducted according to the treatment the participant actually received.

## 7.1. Adverse Events

Treatment-emergent adverse events (TEAEs) are defined as those AEs that develop or worsen after the first dose of double-blinded investigational product and up to 56 days beyond the last dose of investigational product. Analysis of AEs (as discussed below) will be limited to TEAEs, but data listings will include all AEs regardless of their timing.

All reported AEs will be coded using the current version of MedDRA and summarized by System Organ Class (SOC) and Preferred Term (PT). All summaries by SOC and PT will be ordered by decreasing frequency of participants within each SOC and then similarly by decreasing frequency of participants within each PT based on the pegcetacoplan group. In the case of equal frequency of number of participants in SOCs or PTs, summaries of TEAEs will be sorted alphabetically. Rules for handling missing severity, relationship to study drug, relationship to infusion procedure, outcome, and missing dates of AEs are described in Section 13.

If a PT was reported more than once for the same participant, that participant would only be counted once in the incidence for that PT. For participants experiencing the same PT at multiple severities, the occurrence of the AEs with the greatest severity will be used in the analysis of incidence by severity. For participants experiencing the same PT at multiple relationship levels, the occurrence of the AEs with the strongest relationship to study drug will be used in the analysis of incidence by relationship to study drug.

The AE summaries will be presented by treatment group. In addition, the incidence of death, stratified by transplant history will be summarized.

In participants who are posttransplant, the number and incidence of rejection episodes at week 26 will be tabulated by treatment group. Additionally, if the number of participants is sufficient, the results for treatment groups will be compared using Fisher's exact test (or a stratified Cochran-Mantel-Haenszel $\chi$-square test as appropriate).

In participants who are posttransplant, the number and incidence of graft loss at week 26 will be tabulated by treatment group. Additionally, if the number of participants is sufficient, the results for treatment groups will be compared using Fisher's exact test (or a stratified Cochran-Mantel-Haenszel $\chi$-square test as appropriate).

An overall summary for TEAEs including number of participants who experience a TEAE, number of total TEAEs and number of unique TEAEs will be provided:

- TEAE
- Treatment-related TEAE
- Infusion-related TEAEs

- Serious TEAE
- Maximum severity of TEAEs
- Infusion site reaction
- TEAE leading to treatment withdrawn
- TEAE leading to dose interruption
- TEAE leading to study discontinuation
- TEAE leading to death
- Rejection episodes (for posttransplant only)
- Graft loss (for posttransplant only)

The following summaries will be provided:

- TEAEs by SOC and PT
- TEAEs by PT in decreasing order of frequency
- Treatment-related TEAEs by SOC and PT
- Infusion-related TEAEs by SOC and PT
- Serious TEAEs by SOC and PT
- TEAE by SOC, PT and maximum severity
- TEAE leading to treatment discontinuation by SOC and PT
- TEAE leading to dose interruption by SOC and PT
- TEAE leading to study discontinuation by SOC and PT
- TEAE leading to death by SOC and PT

The following listings will be provided:

- All AEs
- Serious adverse events (SAEs)
- AEs leading to study drug discontinuation
- Death
- Treatment-related adverse events
- Infusion-related adverse events

## 7.2. Clinical Laboratory Data

All laboratory results will be standardized by converting values in original units to values in standard units and classified as normal, abnormal low, or abnormal high on normal ranges supplied by the local laboratories and upon employing standardization.

When a certified local laboratory is used instead of a central laboratory due to COVID-19 pandemic, the units and normal ranges might be different. Therefore, all local laboratory values will be standardized to a central laboratory value using the following formula:

$$X_S = L_S + \frac{U_S - L_S}{U_X - L_X} \times (X - L_X)$$

Where,
$X$: local lab value
$X_S$: standardized value of X
$L_S$: Low normal range for central lab
$U_S$: High normal range for central lab
$L_X$: Low normal range for local lab
$U_X$: High normal range for local lab

Specifications will be developed to detail all the steps necessary for developing the standardized laboratory values and to address issues like missing normal ranges or negative standardized laboratory values.

Observed and change from baseline of clinical laboratory data (hematology, serum chemistry, coagulation and continuous urinalysis parameters) will be summarized at each analysis visit by treatment group and overall. Categorical urinalysis data will also be tabulated at each analysis visit when applicable.

In addition, liver abnormalities will be summarized at each analysis visit including following variables by treatment group and overall:

- ALT or AST ≥ 3×ULN
- ALT or AST ≥ 3×ULN and (TBL >2×ULN or INR >1.5)
- ALT or AST ≥ 5×ULN
- ALT or AST >8×ULN

Clinical laboratory test values are potentially clinically significant (PCS) if they meet either the low or high PCS criteria listed in the table below:

**Table 2: Criteria for Potentially Clinically Significant Laboratory Tests**

| Parameter | Criteria |
|---|---|
| **Hematology** | |
| WBC (total) (x10^9/L) | < 3.0<br>> 16 |
| Lymphocyte (x10^9/L) | < 0.5<br>< 0.8<br>> 12 |

**Table 2: Criteria for Potentially Clinically Significant Laboratory Tests**

| **Parameter** | **Criteria** |
|---|---|
| Neutrophils (x10^9/L) | < 1.0<br>< 1.5<br>> 12 |
| RBC (x10^12/L) | < 3.3<br>> 6.8 |
| Hemoglobin (g/dL) | < 10 |
| Platelet count (x10^9/L) | < 100<br>> 600 |
| **Serum Chemistry** | |
| ALT | > 1.5xULN<br>> 3.0xULN |
| AST | > 1.5xULN<br>> 3.0xULN |
| ALP | > 1.5xULN<br>> 3.0xULN |
| Total Serum Bilirubin | > 1.5xULN |
| GGT | > 3.0xULN |
| ALT or AST > 3xULN and concurrent elevated total bilirubin defined as | > 2.0xULN |

For the following serum chemistry parameters: creatinine; LDH; creatine kinase (CK); uric acid; glucose; sodium; potassium; and chloride, abnormal values will be used as potentially clinically significant.

Similarly, for urinalysis parameters, protein; glucose; blood; nitrite; and leukocyte esterase, abnormal values will be used as potentially clinically significant.

The number and percentage of participants with post-baseline PCS laboratory values will be tabulated by treatment group and overall. A listing of participant data with at least one post-baseline PCS value will be provided.

All laboratory values will be listed for each participant.

## 7.3. Vital Signs

Observed and change from baseline in vital signs (pulse rate, respiratory rate, systolic and diastolic blood pressure, temperature, and weight) will be summarized by treatment group and overall at each analysis visit.

Additionally, the number and percentage of participants with post-baseline potentially clinically significant (PCS) vital sign values will be tabulated. Vital signs are potentially clinically significant (PCS) if they meet the PCS criteria listed in the table below:

**Table 3: Criteria for Potentially Clinically Significant Vital Signs**

| VS parameter | Criteria |
|---|---|
| HR | ≥100 BPM<br><40 BPM |
| SBP | ≥130 mm Hg<br>≥160 mm Hg<br>≥ 180 mm Hg<br>≥20 mm Hg increase from baseline<br><90 mm Hg<br>≥20 mm Hg decrease from baseline |
| DBP | ≥90 mm Hg<br>≥15 mm Hg increase from baseline<br><40 mm Hg<br>≥15 mm Hg decrease from baseline |
| Temp | ≥ 38°C |

All vital sign values and change from baseline values will be listed. In the listing, potentially clinically significant vital sign values will be flagged.

## 7.4. Electrocardiograms (ECGs)

Observed and change from baseline in ECGs (heart rate, PR interval, QRS duration, QT interval, and QTc interval which is corrected by Fridericia's method) will be summarized by treatment group and overall at each analysis visit.

Additionally, the number and percentage of participants with post-baseline potentially clinically significant (PCS) ECG values will be tabulated. ECGs are potentially clinically significant (PCS) if they meet the PCS criteria listed in the table below:

**Table 4: Criteria for Potentially Clinically Significant ECGs**

| ECG parameter | Criteria |
|---|---|
| HR | < 40 bpm<br>> 100 bpm |
| PR | > 200 msec |
| QRS | > 120 msec |
| QTcF | > 450 msec<br>> 480 msec<br>> 500 msec |
| QTcF increase from baseline | > 30 msec<br>> 60 msec |

All ECG values and change from baseline values will be listed. In the listing, potentially clinically significant ECG values will be flagged.

## 7.5. Other Safety Data

Physical examinations will be described in a data listing.

# 8. PHARMACOKINETIC ANALYSIS

All summaries and analyses of the pharmacokinetic data will be based on the PK set as defined in Section 4.6.

## 8.1. Drug Concentration

Individual pegcetacoplan concentrations, actual sampling times and deviations from nominal sampling times will be presented in a data listing for all participants included in the PK set. Placebo samples will also be included in the data listing if they are analyzed but will not be summarized.

Pegcetacoplan concentrations will be summarized by treatment group at each scheduled time point using descriptive statistics (including mean, SD, coefficient of variation (CV), median, min, max, geometric mean/%CV). The number of participants with a BLQ concentration at each scheduled time point will also be tabulated. The handling of BLQ concentrations in the summary tables is described in Section 8.2. Missing values will be omitted from the calculation of descriptive statistics.

Linear and semilogarithmic individual concentration-time profiles will be generated using actual sampling times. Linear and semilogarithmic mean (± SD/SE) and median concentration-time profiles will be generated using nominal sampling times. The number of participants contributing to each mean or median value at a visit will be presented above the x-axis.

## 8.2. Handling BLQ Values

### 8.2.1. Handling of BLQ Concentrations in Summary Tables

BLQ concentrations prior to first dosing (day 1): Pre-treatment pegcetacoplan concentrations reported as below the limit of quantification (BLQ) will be taken as zero for the computation of descriptive statistics, except geometric mean. Geometric mean cannot be calculated and will be reported as “N/A” or “-”.

BLQ concentrations occurring at any time after first dosing: Pegcetacoplan concentrations reported as below the limit of quantification (BLQ) will be taken as half the lower limit of quantification (LLOQ/2).

### 8.2.2. Handling of BLQ Concentrations in Figures

BLQ concentrations prior to first dosing (day 1): Pre-treatment pegcetacoplan concentrations reported as below the limit of quantification (BLQ) will be taken as zero for linear plots, and equal to half the lower limit of quantification (LLOQ/2) for semilogarithmic plots.

BLQ concentrations occurring at any time after first dosing: Pegcetacoplan concentrations reported as BLQ will be taken as half the lower limit of quantification (LLOQ/2) for both linear and semilogarithmic plots.

# 9. PHARMACODYNAMIC ANALYSIS

## 9.1. Pharmacodynamic Data

All summaries and analyses of the pharmacodynamic data will be based on the PD set as defined in Section 4.7.

Observed values, changes from baseline and percentage changes from baseline in C3, AH50, CH50, C3b/iC3b, sC5b9, C4, and C3a will be summarized at each protocol specified time point using descriptive statistics.

Individual observed values and individual changes from baseline will be presented graphically. Actual sampling times will be used for the graphical presentation of individual data. The mean (± SE) of the observed values, mean changes from baseline, and mean percentage changes from baseline will also be presented graphically. Nominal sampling times will be used for the mean plots.

PD parameters will be listed together with changes from baseline and percentage changes from baseline by treatment group.

In addition to the analyses outlined above, all PK and complement biomarker concentration data may be used to develop the population PK and exposure-response models in conjunction with other clinical study data. The methods and procedures will be described in a separate Analysis Plan if needed. The results from population modeling will be reported separately.

# 10. IMMUNOGENICITY

Immunogenicity data will be listed and summarized using safety set separately for anti-pegcetacoplan peptide antibody and anti-PEG antibody results. A sample and participant level summary table will be presented as described below.

**Sample Level Summary**

The number of evaluable (ADA-positive, ADA-negative, and ADA-inconclusive) and unevaluable samples will be summarized by treatment group and overall. The number and percentage of each ADA sample classification will be summarized by treatment group and overall in the evaluable samples. In addition, the number and percentage of baseline and post-dose ADA positive samples along with the titer range for each will also be summarized by treatment group and overall in samples that are ADA positive.

ADA samples will be classified as follows:

- ADA-Positive Sample – when the sample is positive in the confirmatory assay
- ADA-Negative Sample – when the sample is negative in the screening assay or the confirmatory assay, and drug is at a level that does not interfere with the ADA method
- ADA-Inconclusive Sample – when the sample is negative in the screening assay or the confirmatory assay, and drug is at a level that interferes with the ADA method, then the sample is considered inconclusive
- Unevaluable Sample – when a sample could not be tested for ADA status due to inadequate sample volume, mishandling, or errors in sample collection, processing, storage, etc.

**Participant Level Summary**

The number and percentage of participants with pre-existing ADAs will be summarized by treatment group and overall in the participants with a baseline sample result. In addition, the number and percentage of participants with an ADA positive response, ADA negative response, and ADA inconclusive response as well as the type and kinetics of the positive ADA response will be summarized by treatment group and overall in the evaluable participants. The participant summary table will include a summary by treatment group and overall for the ADA population parameters.

Pre-existing ADA will be defined as follows: any participant with an ADA positive baseline sample from the total participants with a baseline sample result.

Evaluable participants will be defined as follows: a participant with at least one sample taken with a reportable result after first dosing during the treatment or follow-up period.

Evaluable participants will be classified as follows for ADA response:

- ADA-Positive Participant – An evaluable participant with at least one pre-dose sample and one treatment-emergent or treatment-boosted ADA-positive sample at any time after dosing

- ADA-Negative Participant – An evaluable participant without a treatment-emergent or treatment-boosted ADA-positive sample during the treatment or follow-up period
- ADA-Inconclusive Participant – An evaluable participant who cannot be classified as either ADA-positive or ADA-negative (eg, assay drug tolerance issues, post-dose positive without a baseline sample, positive baseline and positive post-dose sample without a titer value, etc.)

ADA positive responses will be classified as follows:

- Treatment-Boosted ADA Response – An evaluable participant with a baseline ADA positive sample and a post-dose ADA positive sample that is ≥ 4x the baseline titer (eg, baseline titer of 10 vs. post-dose titer of 40)
- Treatment-Emergent ADA Response – An evaluable participant with a baseline ADA negative sample and an ADA positive sample after treatment, ADA developed *de novo*

The kinetics of ADA positive responses will be classified as follows:

- Transient ADA Response
  - Treatment-emergent positive participants are classified as having a transient response if they have only a single ADA positive sample (that was not the last assessment), or more than 1 ADA positive sample where the first and last ADA positive samples are separated by a period of less than 112 days (16 weeks), irrespective of any negative and positive samples in between.
  - Treatment-boosted ADA positive participants are classified as having a transient response if they have only a single ADA boosted sample (that was not the last assessment), or more than 1 positive boosted sample where the first and last ADA positive samples are separated by a period of less than 112 days (16 weeks), irrespective of any negative and positive samples in between.
- Persistent ADA Response
  - Treatment-emergent positive participants are classified as having a persistent response if they have more than 1 positive ADA sample ≥ 112 days (16 weeks) apart, or a positive ADA sample at the last timepoint with no further results available
  - Treatment-boosted ADA positive participants were classified as having a persistent response if they have more than 1 positive boosted sample ≥ 112 days (16 weeks) apart, or a positive boosted sample at the last timepoint with no further results available
- Unclassified Response
  - Any ADA positive participant that cannot be defined as having a transient or persistent ADA response

ADA population parameters are derived as follows:

- ADA Prevalence – The proportion of all ADA positive participants, including pre-existing ADA, computed as a percentage of the total number of evaluable and unevaluable participants
- ADA Incidence – The sum of all ADA positive treatment-emergent and treatment-boosted participants computed as a percentage of the total number of evaluable participants

  Note: This population parameter is the same as the percentage of participants with an ADA positive response.

# 11. INTERIM ANALYSIS

No formal interim analysis is planned for this study.

# 12. DATA MONITORING COMMITTEE

A Data Monitoring Committee (DMC) will review cumulative safety/tolerability data (eg, physical examinations, ECGs, vital signs, clinical laboratory tests, and AEs) and efficacy data. The DMC will have the responsibility to conduct a thorough safety assessment at regular pre-defined intervals during the treatment period of the study.

DMC meetings will be held according to the schedule in the DMC charter. An ad hoc DMC data review may be recommended by the DMC or requested by the sponsor at any time during the study.

The remit, roles, and responsibilities of the DMC will be specified in a separate DMC charter. There will be a separate DMC SAP covering the presentation and analyses for the DMC.

# 13. DATA HANDLING CONVENTIONS

## 13.1. General Data Reporting Conventions

All statistical analyses will be performed using SAS® (Version 9.4 or higher, SAS Institute Inc., Cary, NC, USA).

Categorical variables will be tabulated as number of participants and percentage of total number of participants in the given analysis set as noted for each category. Descriptive statistics will be used to summarize continuous variables including number of participants, mean, standard deviation (SD), median, Q1, Q3, minimum and maximum. Mean, Q1, Q3, and median will be reported to 1 more decimal place than the raw data, while the SD will be reported to 2 more decimal places than the raw data. Minimum and maximum will be reported the same as the original data.

Participant specific listings will be provided by treatment group, participant ID, study period and visit, if applicable.

## 13.2. Definition of Study Days

Unless otherwise noted, study days of an evaluation are defined as number of days relative to the first dose date of study drug, which is designated as day 1, and the preceding day is day −1, the day before that is day −2, etc.

- For assessments on/after study day 1, study days are calculated as:
  - (date of assessment– date of study day 1 + 1)
- For assessments before study day 1, study days are calculated as:
  - (date of assessment – date of study day 1)

## 13.3. Definition of Baseline, RCP, and OLP

For all evaluations unless otherwise noted, baseline is defined as the most recent non-missing measurement prior to the first administration of study drug. Baseline can be the same date as first dose, given the measurement is expected prior to first dose when only date information is available.

In by-visit summary tables, the baseline will be summarized using all available data, but also for each visit using only the baseline data from participants with available data at the visit; hence the mean change from baseline will equal the mean visit value – mean baseline value.

Throughout this document ‘change from baseline’ refers to the actual change from baseline (ie, visit value – baseline value).

The date when a participant receives first administration of OLP drug will be defined as the start date of the participant’s OLP, and that date minus one day will be the end date of the participant’s RCP.

## 13.4. Definition of Analysis Visit Windows

Unless otherwise specified, the actual scheduled nominal post-baseline visit will be used for all summaries across time. Post-baseline unscheduled visits and early termination visits will be mapped to a scheduled visit and will be used in the analysis only if the nominal scheduled visit result is missing. Table 5 presents the analysis visit window mapping for unscheduled and early term visits for assessments done mostly every 4 weeks (central lab, vital sign, PK). For other assessments that do not follow Table 5 schedule (such as 24-hour urine, triplicate FMU uPCR, ECG, PD, ADA, etc.), the analysis visit window mapping will be adjusted accordingly. In the case that multiple unscheduled or early termination visits are in the same analysis window, the one closest to the target date will be used. In the event that the windowed visit is mapped to an illogical sequence of visits when considering nearby scheduled visits (ie, windowed visit is higher than the subsequent visit or lower than the preceding visit), the windowed visit will be set to the logical scheduled visit.

**Table 5: Analysis Visit Windows for Unscheduled and Early Termination Visits for Assessments Done Mostly Every 4 Weeks**

| Study period | Analysis visit | Target day | Analysis window | Interval |
|---|---|---|---|---|
| Screening | Week -10 to -4 | -49 | -77 - < -21 | 56 |
| | Week -2 | -14 | -21 – ≤ -1 | 21 |
| Randomized controlled period | Day 1 | 1 | 1 | 1 |
| | Week 4 | 28 | 2 – < 42 | 40 |
| | Week 8 | 56 | 42 – < 70 | 28 |
| | Week 12 | 84 | 70 – < 98 | 28 |
| | Week 16 | 112 | 98 – < 126 | 28 |
| | Week 20 | 140 | 126 – < 154 | 28 |
| | Week 24 | 168 | 154 – < 175 | 21 |
| | Week 26 | 182 | 175 – < OLP start date | NA |
| Open-label period | Week 28 | 196 | OLP start date – <210 | NA |
| | Week 32 | 224 | 210 – < 238 | 28 |
| | Week 36 | 252 | 238 – < 273 | 35 |
| | Week 42 | 294 | 273 – < 315 | 42 |
| | Week 48 | 336 | 315 – < 350 | 35 |
| | Week 52 | 364 | 350 – < 371 | 21 |
| Follow-up period (for participants not entering long term extension) | Week 54 | 378 | 371 – < 385 | 14 |
| | Week 56 | 392 | 385 – < 406 | 21 |
| | Week 60 | 420 | 406 – < 434 | 28 |

## 13.5. Repeated or Unscheduled Assessments of Safety Parameters

For safety parameters, if a participant has repeated assessments before the start of investigational product, then the results from the final assessment made prior to the start of investigational product will be used as baseline. If end of study assessments are repeated or unscheduled, the last post-baseline assessment will be used as the end of study assessment for generating descriptive statistics. However, all post-baseline assessments will be used for Potentially Clinically Significant (PCS) value determination and all assessments will be presented in the data listings.

## 13.6. Handling of Missing, Unused, and Spurious Data

Missing data is addressed in the relevant sections elsewhere. Spurious data will be discussed with data management and others as appropriate to address queries to sites or vendors. In general, all data will be used as reported (and after query resolutions); any decisions to remove data would be made and documented prior to unblinding.

### 13.6.1. Missing Date of Investigational Product

When the date of the last dose of investigational product is missing for a participant in the Safety Set, all efforts should be made to obtain the date from the investigator. If it is still missing after all efforts, then the last visit date will be used in the calculation of treatment duration.

### 13.6.2. Missing Date Information for Prior or Concomitant Medications

For prior or concomitant medications (and/or therapies/procedures), including rescue medications, incomplete (ie, partially missing) start date and/or stop date will be imputed. When the start date and the stop date are both incomplete for a participant, the start date will be imputed first.

#### 13.6.2.1. Incomplete Start Date

The following rules will be applied to impute the missing numerical fields. If the stop date is complete and the imputed start date is after the stop date, then the start date will be imputed using the stop date.

##### 13.6.2.1.1. Missing Day, Month, and Year

In this case, no start date will be imputed. However, the medication will be assumed to be a prior medication. If the stop date is missing or if the stop date is on or after the date of the first dose of investigational product, the medication will also be considered a concomitant medication.

##### 13.6.2.1.2. Missing Day and Month

- If the year of the incomplete start date is the same as the year of the date of the first dose of investigational product, then the day and month of the date of the first dose of investigational product will be assigned to the missing fields.
- If the year of the incomplete start date is before the year of the date of the first dose of investigational product, then December 31 will be assigned to the missing fields.

- If the year of the incomplete start date is after the year of the date of the first dose of investigational product, then 01 January will be assigned to the missing fields.

#### 13.6.2.1.3. Missing Month Only

- The day will be treated as missing and both month and day will be replaced according to the above procedure.

#### 13.6.2.1.4. Missing Day Only

- If the month and year of the incomplete start date are the same as the month and year of the date of the first dose of investigational product, then the day of the date of the first dose of investigational product will be assigned to the missing day
- If either the year is before the year of the date of the first dose of investigational product or if both years are the same but the month is before the month of the date of the first dose of investigational product, then the last day of the month will be assigned to the missing day
- If either the year is after the year of the date of the first dose of investigational product or if both years are the same but the month is after the month of the date of the first dose of investigational product, then the first day of the month will be assigned to the missing day.

### 13.6.2.2. Incomplete Stop Date

The following rules will be applied to impute the missing numerical fields. If the date of the last dose of investigational product is missing, then replace it with the last visit date. If the imputed stop date is before the start date (imputed or non-imputed start date), then the imputed stop date will be equal to the start date.

#### 13.6.2.2.1. Missing Day and Month

- If the year of the incomplete stop date is the same as the year as of the date of the last dose of investigational product, then the day and month of the date of the last dose of investigational product will be assigned to the missing fields
- If the year of the incomplete stop date is before the year of the date of the last dose of investigational product, then 31 December will be assigned to the missing fields
- If the year of the incomplete stop date is after the year of the date of the last dose of investigational product, then 01 January will be assigned to the missing fields.

#### 13.6.2.2.2. Missing Month Only

- The day will be treated as missing and both month and day will be replaced according to the above procedure.

#### 13.6.2.2.3. Missing Day Only

- If the month and year of the incomplete stop date are the same as the month and year of the date of the last dose of investigational product, then the day of the date of the last dose of investigational product will be assigned to the missing day
- If either the year is before the year of the date of the last dose of investigational product or if both years are the same but the month is before the month of the date of the last dose of investigational product, then the last day of the month will be assigned to the missing day
- If either the year is after the year of the last dose of investigational product or if both years are the same but the month is after the month of the date of the last dose of investigational product, then the first day of the month will be assigned to the missing day.

### 13.6.3. Missing Date Information for Adverse Events

For AEs with partial start dates, non-missing date parts will be used to determine if the AE is treatment-emergent or not. If a determination cannot be made using the non-missing date parts as to when the AE occurred relative to study drug administration, eg, AE start year and month are the same as the year and month of the first dose of investigational product, then the AE will be classified as treatment-emergent.

To facilitate categorization of AEs as treatment emergent, imputation of dates can be used. For AEs, the default is to only impute incomplete (ie, partially missing) start dates. Incomplete stop dates may also be imputed when calculation of the duration of an AE is required per the protocol. If imputation of an incomplete stop date is required, and both the start date and the stop date are incomplete for a participant, impute the start date first.

- Rules to impute incomplete start date are the same as stated in Section 13.6.2.1.
- Rules to impute incomplete stop date are the same as stated in Section 13.6.2.2.

### 13.6.4. Missing Severity Assessment for Adverse Events

- If severity is missing for an AE starting prior to the date of study day 1, then a severity of "Mild" will be assigned.
- If the severity is missing for an AE starting on or after the date of study day 1, then a severity of "Severe" will be assigned.

The imputed values for severity assessment will be used for incidence summaries, while the actual values will be presented in data listings.

### 13.6.5. Missing Relationship to Investigational Product for Adverse Events

If the relationship to investigational product is missing for an AE starting on or after the date of study day 1, a causality of "related" will be assigned. The imputed values for relationship to double-blind investigational product will be used for incidence summaries, while the actual values will be presented in data listings.

#### 13.6.6. Character Values of Clinical Laboratory Variables

If the reported value of a clinical laboratory variable cannot be used in a statistical analysis due to, for example, that a character string is reported for a numerical variable. The appropriately determined coded value will be used in the statistical analysis. If the laboratory results are collected as < or > a numeric value, 0.0000000001 will be subtracted or added, respectively to the value. However, the actual values as reported in the database will be presented in data listings.

# 14. ANALYSIS SOFTWARE

Statistical analyses will be performed using Version 9.4 (or newer) of SAS on a suitably qualified environment.

# 15. CHANGES TO ANALYSIS SPECIFIED IN PROTOCOL

Not applicable.

# 16. REFERENCES

Diggle PJ, Liang K-Y, Zeger SL. *Analysis of Longitudinal Data*. Oxford: Clarendon Press, 1994.

Kaplan EL, Meier P. Nonparametric estimation from incomplete observations. *Journal of the American Statistical Association*. 1958;53(282):457-81.

Li KH. Imputation Using Markov Chains. *Journal of Statistical Computation and Simulation*.1988;30:57-79.

Liu C. Bartlett's Decomposition of the Posterior Distribution of the Covariance for Normal Monotone Ignorable Missing Data. *Journal of Multivariate Analysis*.1993; 46:198-206.

O'Kelly M, Ratitch B (editors). Analyses under missing-not-at-random assumptions. *Clinical Trials with Missing Data: A Guide for Practitioners*. Wiley, 2014.

Peipert JD, Nair D, Klicko K, Schatell DR, Hays RD. Kidney Disease Quality of Life 36-Item Short Form Survey (KDQOL-36) normative values for the United States dialysis population and new single summary score. *Journal of the American Society of Nephrology*. 2019; 30(4): 654-663.

# 17. APPENDICES

# APPENDIX 1. SCHEDULE OF ASSESSMENTS

| **Study period** | **Screening period[a]** | | **Randomized controlled period** | | | | | | | | **Open-label period** | | | | | | **Follow-up period** | | |
|---|---|---|---|---|---|---|---|---|---|---|---|---|---|---|---|---|---|---|---|
| **Study week** | **–10 to –4** | **–2** | **1** | **4** | **8** | **12** | **16** | **20** | **24** | **26** | **28** | **32** | **36** | **42** | **48** | **52** | **54** | **56** | **60 Exit** |
| **Study day** | **–70** | **–14** | **1** | **28** | **56** | **84** | **112** | **140** | **168** | **182** | **196** | **224** | **252** | **294** | **336** | **364** | **378** | **392** | **420** |
| **Study visit** | **1** | **2** | **3** | **4** | **5** | **6** | **7** | **8** | **9** | **10** | **11** | **12** | **13** | **14** | **15** | **16** | **17** | **18** | **19** |
| **Visit window (± days)** | **N/A[b]** | | **0** | **3** | **7** | **7** | **7** | **7** | **3** | **3** | **3** | **7** | **7** | **7** | **7** | **7** | **3** | **3** | **7** |
| **Assessments** | | | | | | | | | | | | | | | | | | | |
| Informed consent | X | | | | | | | | | | | | | | | | | | |
| Demographics | X | | | | | | | | | | | | | | | | | | |
| Medical history | X | | | | | | | | | | | | | | | | | | |
| Post-transplant immunosuppression plan documentation | | X | | | | | | | | | | | | | | | | | |
| Inclusion/exclusion | X | X | X | | | | | | | | | | | | | | | | |
| Vaccination[d] | | X[b] | | | | X | | | | | | | | | | | | | |
| Adverse events | X | X | X | X | X | X | X | X | X | X | X | X | X | X | X | X | X | X | X |
| Concomitant medications | X | X | X | X | X | X | X | X | X | X | X | X | X | X | X | X | X | X | X |
| Physical examination (full)[e] | X | | X | | | | | | | X[e] | | | | | | X | | | |
| Physical examination (brief)[e] | | X | | X | X | X | X | X | X | | X[e] | X | X | X | X | | X | X | X |
| 12-lead ECG | X | | X | | X | | X | | X | X | X | | X | | X | X | X | | X |
| Chest radiography | | X[b] | | | | | | | | X | | | | | | X | | | |
| Renal biopsy[f] | X[b] | | | | | | | | | X[g] | | | | | | Xh | | | |
| Randomization | | | X | | | | | | | | | | | | | | | | |
| Study drug administration[i] | | | X | X | X | X | X | X | X | X | X | X | X | X | X | X | | | |
| Infusion site/pump safety assessment[j] | | | X | X | X | X | X | X | X | X | X | X | X | X | X | X | X | | |
| Vital sign measurements[k] | X | X | X | X | X | X | X | X | X | X | X | X | X | X | X | X | X | X | X |
| HRQoLl | | X | | | | | | | | X | | | | | | X | | | |

| **Study period** | **Screening period[a]** | | **Randomized controlled period** | | | | | | | | **Open-label period** | | | | | | **Follow-up period** | | |
|---|---|---|---|---|---|---|---|---|---|---|---|---|---|---|---|---|---|---|---|
| **Study week** | **–10 to –4** | **–2** | **1** | **4** | **8** | **12** | **16** | **20** | **24** | **26** | **28** | **32** | **36** | **42** | **48** | **52** | **54** | **56** | **60 Exit** |
| **Study day** | **–70** | **–14** | **1** | **28** | **56** | **84** | **112** | **140** | **168** | **182** | **196** | **224** | **252** | **294** | **336** | **364** | **378** | **392** | **420** |
| **Study visit** | **1** | **2** | **3** | **4** | **5** | **6** | **7** | **8** | **9** | **10** | **11** | **12** | **13** | **14** | **15** | **16** | **17** | **18** | **19** |
| **Visit window (± days)** | **N/A[b]** | | **0** | **3** | **7** | **7** | **7** | **7** | **3** | **3** | **3** | **7** | **7** | **7** | **7** | **7** | **3** | **3** | **7** |
| **Urine** | | | | | | | | | | | | | | | | | | | |
| 24-hour urine collection[m] | X | | | | | X | | | X | | | | X | | X | | | | X |
| Triplicate FMU uPCR[n] | X | X | X[o] | X | X | X | X | X | X | X | X | X | X | X | X | X | X | X | X |
| In-clinic (random) uPCR | X | X | X | X | X | X | X | X | X | X | X | X | X | X | X | X | X | X | X |
| Urinalysis (dipstick & microscopic)[p] | X | | | | | | | | | | | | | | | | | | |
| Urine pregnancy test | | X | X | X | X | X | X | X | X | X | X | X | X | X | X | X | X | X | X |
| **Blood** | | | | | | | | | | | | | | | | | | | |
| Hematology[p] & chemistry[p] | X | X | X | X | X | X | X | X | X | X | X | X | X | X | X | X | X | X | X |
| PK sample collection | | | X | X | X | X | X | X | X | X | X | X | X | X | X | X | X | X | X |
| ADA assaysq | | | X | X | | | X | | X | | | | X | | | X | X | | X |
| Serum complement profilep | X | X | X | X | X | X | | X | X | X | X | X | X | | X | X | X | X | X |
| Plasma complement profile[p] | X | | X | | X | | X | | X | X | X | | X | | X | X | X | | X |
| eGFRr | X | X | X | X | X | X | X | X | X | X | X | X | X | X | X | X | X | X | X |
| Pregnancy (β-HCG) | X | | | | | | | | | | | | | | | | | | |
| Screening assayss | X | | | | | | | | | | | | | | | | | | |
| **Optional assessments** | | | | | | | | | | | | | | | | | | | |
| Ophthalmologic evaluations[t] | | Xt | | | | | | | | | | | | Xt | | | | | |
| Measured GFR[u] | | | Xu | | | | | | | X[u] | | | | Xu | | | | | |

Abbreviations: Ab = antibodies; ADA = antidrug antibodies; AE = adverse events; AH50 = 50% alternative hemolytic complement pathway activity; ANA = antinuclear antibodies; ANCA = antineutrophil cytoplasmic antibodies; β-HCG = beta human chorionic gonadotropin; CH50 = 50% classical hemolytic complement pathway activity;

ECG = electrocardiogram; eGFR = estimated glomerular filtration rate; EQ-5D-5L = 5-Level EuroQol-5 Dimension; FACIT-Fatigue = Functional Assessment of Chronic Illness Therapy-Fatigue Scale; FMU = first-morning spot urine; FSH = follicle-stimulating hormone; GFR = glomerular filtration rate; HIV = human immunodeficiency virus; HRQoL = health-related quality of life; KDQOL = Kidney Disease Quality of Life; N/A = not applicable; PGIC = Patient Global Impression of Change; PK = pharmacokinetic; SC = subcutaneous; SD-OCT = spectral domain optical coherence tomography; SPEP = serum protein electrophoresis; uPCR = urine protein-to-creatinine ratio; WPAI = Work Productivity and Activity Impairment.

**NOTE:** When multiple assessments/procedures occur at the same visit, order should be: vital signs, ECGs, blood collection/sampling, study drug dosing.

[a] All visit 2 assessments need not occur in a single visit and visit 2 can be split into multiple visits (eg, visit 2a, visit 2b).

[b] Vaccinations, baseline renal biopsy, and screening chest radiography can occur any time during screening after confirmation of eligibility based on visit 1 data.

[c] For transplant participants only. Must be documented prior to assignment to study treatment.

[d] Vaccine series should be initiated at least 14 days prior to randomization; additional vaccines may be required at visit 6. Please see Protocol Section 8.2 for more details on vaccination requirements. Vaccination serum samples will be collected on the day of vaccination before receiving vaccinations on that visit. These samples will be analyzed to evaluate response to vaccinations in the event that the participant has a positive infection for *Streptococcus pneumoniae*, *Neisseria meningitidis*, or *Haemophilus influenzae*.

[e] A full physical examination is required at visits 1, 3 (week 1), 10 (week 26), and 16 (week 52). A full physical examination should also be conducted on the first day of open-label dosing, if that is not at the week 26 visit. Brief physical examinations, including weight (kg) and assessment of edema, will be conducted at all other visits noted. A symptom-driven physical examination may be performed at any time, at the investigator's discretion. Body height (cm) will be measured only during screening for adults but will be measured at screening and every 12 weeks throughout the study for adolescents; weight (kg) will be measured throughout study, during brief and full physical examinations for all participants. Both body weight and height will be assessed without shoes on; height will be measured using a calibrated stadiometer. Edema should be assessed at every visit.

[f] Renal biopsies will not be required for participants younger than 18 years provided that they have adequate previous renal biopsies to establish the diagnosis as per the central pathology laboratory.

[g] The week 26 renal biopsy need not occur on the same day as all other assessments for that visit. However, the week 26 triplicate FMU uPCR collections must occur before the renal biopsy. In addition, the week 26 biopsy must occur before the first visit of the open-label period at week 28. Participants younger than 18 years are not required to provide renal biopsies and may advance to the open-label period upon completion of all assessments for weeks 24 through 26 other than the renal biopsy.

[h] The week 52 renal biopsy is optional for all participants. If performed it should be after collection of the week 48 24-hour urine and FMU samples, and not more than 8 weeks after the week 52 visit. In the event that a participant has a renal biopsy as part of their clinical management within this window, it may serve as the week 52 biopsy provided that it includes the required components for this study.

[i] Study drug will be self-administered by the participant or administered by their caregiver, after receiving appropriate training and sign-off by a research nurse (or other qualified personnel) in their first treatment week, as described in Protocol Section 9.5. Once qualified, the participant or caregiver should administer study drug at site visits (as done at home) on those days when a clinic visit occurs on a dosing day. If a home nurse is administering study drug on nonvisit days, the site staff may administer study drug on days of site visits.

[j] Between site visits, participants will be instructed to report any infusion site reactions to the study staff. Pump use safety will be reviewed by licensed health care professionals (eg, investigator or nurse) for each study drug administration at clinic visits and during at-home qualification.

[k] Vital signs should be measured a maximum of 2 hours before study drug infusion. On day 1 and on the first day of open-label dosing (week 26 or week 28, depending on whether a week 26 renal biopsy is required and when it is performed), vital signs should also be measured approximately 30 minutes to 1 hour after the first infusion of study drug dosing, timed from the completion of the study drug administration. Blood pressure and heart rate should be evaluated after the participant has been resting in a seated position for at least 5 minutes, except when they are supine or semireclined because of study procedures and/or AEs, or if deemed necessary by the investigator.

[l] FACIT-Fatigue, EQ-5D-5L, KDQOL, PGIC, and WPAI.

[m] The screening 24-hour urine collection may be done any time between screening visit 1 and screening visit 2. After week 1, collections should be within ±1 week of the visit (except for the week 24 collection, which cannot be earlier than week 24). Courier arrangements can be made to pick up the collection container from the participant, or the participant may return the container directly to the site.

n. Triplicate FMU samples will be collected by the participant at home on 3 consecutive days throughout the duration of the study. These should be the first urinary output of the day. An additional triplicate FMU uPCR sample will be collected at week 25. Samples should be collected within ±1 week of the clinic visit (except for weeks 24 through 26, when sample collection should be within ±3 days of the clinic visit). Courier arrangements can be made to pick up the collection containers from the participants, or the participant may return the containers directly to the site. At every visit, enough uPCR collection containers should be dispensed to the participant to enable all at-home uPCR collections until the next clinic visit.

o. The day 1 triplicate FMU uPCR samples should be collected before the first dose of study drug (eg, day –2, day –1, and before dosing on day 1).

p. Serum complement profile includes AH50, CH50, and C3NeF; C3NeF will only be assayed in samples collected at the baseline (screening) visits. Plasma complement profile includes C3a, C3b/iC3b, C5a, and sC5b-9. See laboratory assessments (Protocol Table 4) for more details.

q. The day 1 samples should be collected before dosing with study drug. Participants who discontinue dosing will have ADA samples collected at 2 and 8 weeks after the last treatment. Participants who have a treatment-emergent or treatment-boosted ADA response at any time will have ADA samples collected approximately every 6 months until the antibody levels revert to baseline.

r. eGFR will be calculated using the Chronic Kidney Disease–Epidemiology Collaboration creatinine equation for adults or the Bedside Schwartz equation for adolescents. For each participant, eGFR will be calculated using the same formula for the duration of the study; the choice of formula will be determined by the participant's age at study entry.

s. Serum FSH (to be measured in female participants only), hepatitis B panel, hepatitis C panel, HIV antibodies, SPEP (adult participants only), ANA, and ANCA (see Protocol Table 4 and laboratory manual for more details).

t. Ophthalmologic evaluations are optional and will be performed at selected sites. If evaluations are performed, each participant should have a baseline ophthalmologic evaluation, including a basic ophthalmologic examination, SD-OCT, and color fundus photography, at an approved ophthalmologic clinical site at any time during the screening period. For participants who have drusen before pegcetacoplan administration, a follow-up ophthalmologic evaluation, including an SD-OCT and color fundus photography, should occur at a convenient time between weeks 44 and 52.

u. Measured GFR is an optional assessment that, if performed, should occur at 3 time points: once on day 1 or within 10 weeks before day 1, again at week 26, and a third time between week 44 and week 52, inclusive. Measured GFR should only be done at sites where it is routinely performed, as per the site's standard protocol.

# APPENDIX 2. SELECT SAMPLE SAS® CODE

To be added later.

STATISTICAL ANALYSIS PLAN (FINAL)
**Statistical Analysis Plan: 2.0**
**Protocol Number: APL2-C3G-310**

# STATISTICAL ANALYSIS PLAN

**Pegcetacoplan (APL-2)**
**PHASE 3**

# A Phase 3, Randomized, Placebo-Controlled, Double-Blinded, Multicenter Study to Evaluate the Efficacy and Safety of Pegcetacoplan in Patients with C3 Glomerulopathy or Immune-Complex Membranoproliferative Glomerulonephritis

**PROTOCOL IDENTIFIER: VALIANT**

| | |
|---|---|
| **Study Sponsor(s)**: | Apellis Pharmaceuticals, Inc<br>100 5th Ave, Waltham, MA 02451 |

| | |
|---|---|
| Author: | |
| Protocol: | Amendment 4 dated 25 April 2024 |
| SAP Version #: | 2.0 |
| SAP Date: | 27 June 2024 |
| Status: | Final |

Individuals printing or viewing Apellis procedural document from a source other than the Electronic Document Management System are responsible for ensuring that they are printing or viewing the current version. This document may not be distributed outside of Apellis without approval from Global Quality.

# REVISION HISTORY

| Version | Issue date | Summary of changes |
|---|---|---|
| 1.0 | 07 September 2023 | Original |
| 2.0 | 27 June 2024 | • Revised the order of key secondary efficacy endpoints; change from baseline in eGFR is now the last key secondary endpoint.<br>• Removed exploratory endpoint evaluating normalization of hematuria.<br>• Removed exploratory endpoint of evaluation of changes in drusen at 26 weeks.<br>• Updated strategies for addressing ICEs.<br>• Removed mITT set from statistical analysis sets.<br>• Provided further details on the analysis of C3c staining endpoint.<br>• Provided further details on the analysis of time-to-event endpoints.<br>• Provided further details on the analyses of FACIT-Fatigue score, EQ-5D-5L score, and WPAI score endpoints.<br>• Provided further details on immunogenicity analysis.<br>• Provided further details on the analysis of baseline disease characteristics.<br>• Updated strategies for addressing missing data in PP analysis.<br>• Clarified more details on the covariance structures for the MMRM models.<br>• Provided further details on the analysis window mapping for roll-over participants.<br>• For endpoints of C3c histologic activity score, FACIT-Fatigue score, and KDQOL score, clarified in the null/alternative hypothesis that the change from baseline is being modeled instead of the value at week 26.<br>• Clarified for the key secondary endpoints of activity score and C3c staining reduction, modeling will only be based on adult participants.<br>• Updated analysis for the exploratory endpoint of annual rate of change from up to 3 years prior to screening in eGFR.<br>• Updated analysis for the exploratory endpoint of glomerular macrophage count.<br>• Updated analysis for the exploratory endpoint of PGIC score.<br>• Provided more details on the activity score endpoint regarding the primary, secondary, and adjudication reports.<br>• Updated to assign different randomization seeds for different endpoints.<br>• Updated safety analysis to add summary tables for AESIs and exposure-adjusted AEs.<br>• Added new subgroup “baseline immunosuppressants use” in all subgroup analyses, the new subgroup has also been added in primary analysis modeling<br>• Added clarification languages. |

DocuSign Envelope ID: F3CECE8F-19C9-4A91-B302-44798097142C



# APPROVAL SIGNATURES

**Date (dd-Mmm-yyyy)**

**Apellis Pharmaceuticals, Inc.**

**Date (dd-Mmm-yyyy)**

**Apellis Pharmaceuticals, Inc.**

# TABLE OF CONTENTS

REVISION HISTORY ....2
APPROVAL SIGNATURES ....3
TABLE OF CONTENTS ....4
LIST OF TABLES ....8
LIST OF FIGURES ....8
LIST OF ABBREVIATIONS ....9
1. INTRODUCTION ....12
2. OBJECTIVES AND ENDPOINTS ....13
2.1. Objectives ....13
2.1.1. Primary Objective ....13
2.1.2. Secondary Objectives ....13
2.1.3. Exploratory Objectives ....13
2.2. Endpoints ....13
2.2.1. Primary Efficacy Endpoint ....13
2.2.2. Key Secondary Efficacy Endpoints ....13
2.2.3. Additional Secondary Efficacy Endpoints ....14
2.2.4. Exploratory Efficacy Endpoints ....14
2.2.5. Safety Endpoints ....15
2.2.6. Pharmacokinetic Endpoint ....15
2.2.7. Pharmacodynamics Endpoints ....15
2.2.8. Immunogenicity Endpoint ....15
3. STUDY DESIGN ....16
3.1. General Description ....16
3.2. Randomization ....17
3.3. Blinding ....17
3.4. Sample Size and Power Considerations ....18
3.5. Analysis Timing and Unblinding ....18
4. STATISTICAL ANALYSIS SETS ....19
4.1. Screened Set ....19
4.2. Intent-to-Treat Set ....19
4.3. Safety Set ....19

4.4. Per-protocol Set ....................19
4.5. Pharmacokinetic Set ....................19
4.6. Pharmacodynamic Set ....................19
5. STUDY PARTICIPANTS ....................20
5.1. Disposition of Participants ....................20
5.2. Demographic and Other Baseline Characteristics ....................20
5.3. Medical History ....................21
5.4. Prior and Concomitant Medications ....................21
5.5. Prior and Concomitant Procedures ....................22
5.6. Exposure to Investigational Product ....................22
5.7. Measurements of Treatment Compliance ....................22
5.8. Protocol Deviations ....................22
6. EFFICACY ANALYSES ....................23
6.1. Analysis Models ....................23
6.2. Multiplicity Adjustment ....................24
6.3. Estimands ....................24
6.4. Analyses of Primary Efficacy Endpoint ....................27
6.4.1. Main Analysis of Primary Efficacy Endpoint ....................27
6.4.2. Sensitivity Analyses of Primary Efficacy Endpoint ....................29
6.4.3. Supplemental Analyses of Primary Efficacy Endpoint ....................30
6.4.3.1. Per Protocol Set ....................30
6.4.3.2. Slope Analysis ....................30
6.4.4. Subgroup Analyses of Primary Efficacy Endpoint ....................30
6.5. Analyses of Key Secondary Efficacy Endpoints ....................31
6.5.1. The Proportion of Participants Who Meet the Criteria for Achieving a Composite Renal Endpoint ....................31
6.5.1.1. Main Analysis ....................32
6.5.1.2. Supplemental Analyses ....................32
6.5.1.3. Subgroup Analyses ....................32
6.5.2. The Proportion of Participants With a Reduction of at least 50% from Baseline in uPCR ....................32
6.5.2.1. Main Analysis ....................33
6.5.2.2. Supplemental Analyses ....................33

6.5.2.3. Subgroup Analyses ....................33
6.5.3. Change From Baseline in the Activity Score of the C3G Histologic Index Score ....................33
6.5.3.1. Main Analysis ....................34
6.5.3.2. Sensitivity Analyses ....................34
6.5.3.3. Supplemental Analyses ....................34
6.5.3.4. Subgroup Analyses ....................34
6.5.4. The Proportion of Participants Showing Decreases in C3c Staining From Baseline ....................34
6.5.4.1. Main Analysis ....................35
6.5.4.2. Supplemental Analyses ....................35
6.5.4.3. Subgroup Analyses ....................35
6.5.5. Change From Baseline in eGFR ....................35
6.5.5.1. Main Analysis ....................36
6.5.5.2. Sensitivity Analyses ....................36
6.5.5.3. Supplemental Analyses ....................36
6.5.5.4. Subgroup Analyses ....................36
6.6. Analyses of Additional Secondary Efficacy Endpoints ....................37
6.6.1. The Proportion of Participants Achieving Proteinuria <1 g/day ....................37
6.6.2. The Proportion of Participants With Normalization of Serum Albumin Levels ....................37
6.6.3. The Proportion of Participants With Serum C3 Levels Above the LLN ....................38
6.6.4. Change From Baseline in FACIT-Fatigue Scale Score ....................39
6.6.5. Change From Baseline in KDQOL Score ....................39
6.7. Analyses of Exploratory Efficacy Endpoints ....................40
6.7.1. The Change From Baseline in uPCR Using the 24-Hour Urine Collections ....................40
6.7.2. The Annual Rate of Change From up to 3 Years Prior to Screening in eGFR ....................41
6.7.3. The Proportion of Participants With Reductions From Baseline in Proteinuria of at least 30% ....................41
6.7.4. The Proportion of Participants With Normalization of Proteinuria ....................41
6.7.5. Time to 50% Reduction in uPCR With a Stable or Improved eGFR ....................42
6.7.6. Time to Normalization for Selected Parameters for Participants in Whom the Parameter is Abnormal at Baseline ....................42

6.7.7. The Change From Baseline in Glomerular Macrophage Count ....42
6.7.8. The Change From Baseline in PGIC Score ....42
6.7.9. The Change From Baseline in EQ-5D-5L Score ....43
6.7.10. The Change From Baseline in WPAI Score ....43
6.7.11. Correlation Analysis on Reduction in uPCR and Change in eGFR ....43
7. SAFETY ANALYSIS ....44
7.1. Adverse Events ....44
7.2. Clinical Laboratory Data ....46
7.3. Vital Signs ....48
7.4. Electrocardiogram (ECG) ....49
7.5. Other Safety Data ....50
8. PHARMACOKINETIC ANALYSIS ....51
8.1. Drug Concentration ....51
8.2. Handling BLQ Values ....51
8.2.1. Handling of BLQ Concentrations in Summary Tables ....51
8.2.2. Handling of BLQ Concentrations in Figures ....51
9. PHARMACODYNAMIC ANALYSIS ....52
9.1. Pharmacodynamic Data ....52
10. IMMUNOGENICITY ....53
11. INTERIM ANALYSIS ....56
12. DATA MONITORING COMMITTEE ....57
13. DATA HANDLING CONVENTIONS ....58
13.1. General Data Reporting Conventions ....58
13.2. Definition of Study Days ....58
13.3. Definition of Baseline, RCP, and OLP ....58
13.4. Definition of Analysis Visit Windows ....59
13.5. Repeated or Unscheduled Assessments of Safety Parameters ....60
13.6. Handling of Missing, Unused, and Spurious Data ....60
13.6.1. Missing Date of Investigational Product ....60
13.6.2. Missing Date Information for Prior or Concomitant Medications (Therapies/Procedures) ....60
13.6.2.1. Incomplete Start Date ....60
13.6.2.2. Incomplete Stop Date ....61

13.6.3. Missing Date Information for Adverse Events ....62
13.6.4. Missing Severity Assessment for Adverse Events ....62
13.6.5. Missing Relationship to Investigational Product for Adverse Events ....63
13.6.6. Character Values of Clinical Laboratory Variables ....63
14. ANALYSIS SOFTWARE ....64
15. CHANGES TO ANALYSIS SPECIFIED IN PROTOCOL ....65
16. REFERENCES ....66
17. APPENDICES ....67
17.1. Schedule of Assessments ....68
17.2. Sample SAS code ....71
17.2.1. Mixed Effect Model for Repeated Measure ....71
17.2.2. Multiple Imputation ....73
17.2.3. Rate of Change Models ....79
17.2.4. Logistic Model ....82
17.2.5. ANCOVA Model ....84
17.2.6. Time-to-Event Analysis ....84

# LIST OF TABLES

Table 1: Estimands and Attributes for Primary, Key Secondary, and Additional Secondary Endpoints ....25
Table 2: Criteria for Potentially Clinically Significant Laboratory Tests ....47
Table 3: Criteria for Potentially Clinically Significant Vital Signs ....49
Table 4: Criteria for Potentially Clinically Significant ECGs ....49
Table 5: Analysis Visit Windows for Unscheduled and Early Termination Visits for Assessments Done Mostly Every 4 Weeks ....59

# LIST OF FIGURES

Figure 1: Study Design ....17

# LIST OF ABBREVIATIONS

| Term | Definition |
|---|---|
| ADA | anti-drug antibodies |
| AE | adverse event |
| AESI | Adverse event of special interest |
| AH50 | 50% alternative hemolytic complement pathway activity |
| ALP | alkaline phosphatase |
| ALT | alanine aminotransferase |
| ANCOVA | analysis of covariance |
| AR | autoregressive |
| ARH | heterogeneous autoregressive |
| AST | aspartate aminotransferase |
| ATC | anatomical therapeutic class |
| BLQ | below limit of quantification |
| BMI | body mass index |
| C3G | C3 glomerulopathy |
| C3GN | C3 glomerulonephritis |
| CH50 | 50% classical hemolytic complement pathway activity |
| CI | confidence interval |
| CKD-EPI | Chronic Kidney Disease–Epidemiology Collaboration |
| COVID-19 | coronavirus disease 2019 |
| CRO | contract research organization |
| CS | compound symmetry |
| CV | coefficient of variation |
| DBP | diastolic blood pressure |
| DDD | dense deposit disease |
| DMC | data monitoring committee |
| ECG | electrocardiogram |
| eGFR | estimated glomerular filtration rate |
| EQ-5D-5L | 5-Level EuroQol-5 Dimension |
| FACIT | Functional Assessment of Chronic Illness Therapy |
| FMU | first-morning spot urine |
| GGT | gamma-glutamyltransferase |

| Term | Definition |
|---|---|
| IC-MPGN | immune-complex membranoproliferative glomerulonephritis |
| ICE | intercurrent event |
| INR | international normalized ratio |
| ITT | intention-to-treat |
| KDQOL | kidney disease quality of life |
| LLN | lower limit of normal |
| LLOQ | lower limit of quantification |
| LS | least square |
| MAR | missing at random |
| MedDRA | Medical Dictionary for Regulatory Activities |
| MI | multiple imputation |
| MMRM | mixed-effect model for repeated measures |
| MNAR | missing not at random |
| OLP | open-label period |
| PCS | potentially clinically significant |
| PD | pharmacodynamic |
| PEG | polyethylene glycol |
| PGIC | patient global impression of change |
| PK | pharmacokinetic |
| PP | per-protocol |
| PT | Preferred Term |
| QTcF | QT interval corrected for heart rate using Fridericia's formula |
| RBC | red blood cell |
| RCP | randomized controlled period |
| SAE | serious adverse event |
| SAP | statistical analysis plan |
| SBP | systolic blood pressure |
| SC | subcutaneous |
| SD | standard deviation |
| SE | standard error |
| SOC | system organ class |
| TBL | total bilirubin |

| Term | Definition |
|---|---|
| TEAE | treatment-emergent adverse event |
| ULN | upper limit of normal |
| uPCR | urine protein-to-creatinine ratio |
| WBC | white blood cell |
| WHO | World Health Organization |
| WPAI | work productivity and activity impairment |

# 1. INTRODUCTION

This statistical analysis plan (SAP) provides a technical and detailed elaboration of the statistical analyses of efficacy, safety, and pharmacokinetic/pharmacodynamic/immunogenicity data as described in the study protocol Amendment 4 dated 25 April 2024. Specifications for tables, figures, and listings are contained in a separate document.

The current SAP describes analysis of the randomized controlled period (RCP, up to week 26). Analysis on the interim data post-week 26 at the time of the datacut, referred to as the open-label period (OLP) reporting, will be provided in an SAP addendum.

# 2. OBJECTIVES AND ENDPOINTS

## 2.1. Objectives

### 2.1.1. Primary Objective

The primary objective of this study is to assess the efficacy of twice-weekly subcutaneous (SC) doses of pegcetacoplan compared with that of placebo in patients with primary C3 glomerulopathy (C3G) or immune-complex membranoproliferative glomerulonephritis (IC-MPGN) on the basis of a reduction in proteinuria.

### 2.1.2. Secondary Objectives

The secondary objectives of the study are as follows:

- To assess the effect of pegcetacoplan on estimated glomerular filtration rate (eGFR)
- To assess the effect of pegcetacoplan on additional C3G/IC-MPGN disease-related parameters
- To evaluate the safety of pegcetacoplan over 52 weeks of treatment

### 2.1.3. Exploratory Objectives

The exploratory objectives are to characterize the additional clinical, laboratory, and histologic findings of C3G/IC-MPGN in response to pegcetacoplan.

## 2.2. Endpoints

### 2.2.1. Primary Efficacy Endpoint

The primary efficacy endpoint is the log-transformed ratio of urine protein-to-creatinine ratio (uPCR) at week 26 compared to baseline.

### 2.2.2. Key Secondary Efficacy Endpoints

The key secondary efficacy endpoints are (to be evaluated at week 26):

- The proportion of participants who meet the criteria for achieving a composite renal endpoint (a stable or improved eGFR compared to the baseline visit (≤15% reduction in eGFR), and a ≥50% reduction in uPCR compared to the baseline visit.)
- The proportion of participants with a reduction of at least 50% from baseline in uPCR
- For participants with evaluable renal biopsies, the change from baseline in the activity score of the C3G histologic index score
- The proportion of participants with evaluable renal biopsies showing decreases in C3c staining on renal biopsy from baseline
- Change from baseline in eGFR

### 2.2.3. Additional Secondary Efficacy Endpoints

The additional secondary endpoints are (to be evaluated at week 26):

- The proportion of participants achieving proteinuria <1 g/day
- For participants with serum albumin levels below the lower limit of normal (LLN) at baseline, the proportion of participants with normalization of serum albumin levels
- For participants with serum C3 levels below the LLN at baseline, the proportion of participants with serum C3 levels above the LLN
- The change from baseline in Functional Assessment of Chronic Illness Therapy (FACIT)–Fatigue Scale score
- The change from baseline in Kidney Disease Quality of Life (KDQOL) score

### 2.2.4. Exploratory Efficacy Endpoints

The primary, key secondary, and additional secondary efficacy endpoints will also be evaluated at week 52 as exploratory efficacy endpoints.

In addition, the following exploratory efficacy endpoints will be evaluated:

- The change from baseline in uPCR using the 24-hour urine collections at week 24 and week 48
- The annual rate of change from up to 3 years prior to screening in eGFR at week 26 and week 52
- The proportion of participants with reductions from baseline in proteinuria of at least 30% at week 26 and week 52
- The proportion of participants with normalization of proteinuria at week 26 and week 52
- The time to 50% reduction in uPCR with a stable or improved eGFR
- The time to normalization for the following parameters for participants in whom the parameter is abnormal at baseline:
  - serum C3
  - uPCR
  - serum albumin
  - blood pressure
- The change from baseline in glomerular macrophage count, as determined by CD68 staining at week 26 and week 52
- The change from baseline in Patient Global Impression of Change (PGIC) score at week 26 and week 52
- The change from baseline in 5-Level EuroQol-5 Dimension (EQ-5D-5L) score at week 26 and week 52

- The change from baseline in Work Productivity and Activity Impairment (WPAI) score at week 26 and week 52
- Change in drusen from baseline at week 52:
  - change in maximum drusen size
  - change in number of intermediate or large drusen

### 2.2.5. Safety Endpoints

The safety endpoints are as follows:

- The incidence and severity of treatment-emergent adverse events (TEAEs)
- The change from baseline in:
  - vital signs measurements
  - clinical laboratory tests
  - Electrocardiogram (ECG) results
- The number and incidence of rejection episodes (posttransplant participants only)
- The number and incidence of graft loss (posttransplant participants only)
- The incidence of death, stratified by transplant history

### 2.2.6. Pharmacokinetic Endpoint

The pharmacokinetic (PK) endpoint is pegcetacoplan serum concentrations over time.

### 2.2.7. Pharmacodynamics Endpoints

The pharmacodynamic (PD) endpoints are:

- Changes from baseline in complement levels at week 26 and week 52:
  - CH50 (50% classical hemolytic complement pathway activity)
  - AH50 (50% alternative hemolytic complement pathway activity)
  - sC5b-9

Additional complement components may be measured, as noted in Protocol Table 4 and Protocol Section 11.1.11, and evaluated as exploratory endpoints.

### 2.2.8. Immunogenicity Endpoint

The immunogenicity endpoint is:

- The incidence of antidrug antibodies (ie, antibodies against the peptide and polyethylene glycol [PEG] domains of pegcetacoplan)

# 3. STUDY DESIGN

## 3.1. General Description

This phase 3 randomized, placebo-controlled, double-blinded, multicenter clinical study is designed to evaluate the safety and efficacy of twice-weekly SC infusions of pegcetacoplan in patients with primary C3G or IC-MPGN. There will be approximately 80 to 100 participants enrolled in this study, at least 78 of whom will have native kidney disease and up to 22 of whom may have posttransplant recurrence of C3G or IC-MPGN. At least 63 participants with C3G in native kidney will be enrolled. The enrollment of participants with C3G or IC-MPGN will be monitored to ensure balance between the groups. At least 10 adolescent participants (aged 12-17 years) will be enrolled; adolescent participants may be either patients with native kidney disease or posttransplant disease recurrence. Participants initially screened as adolescents will follow adolescent procedures and requirements through the duration of their participation in study, even if they pass their 18th birthday while enrolled in the study.

The planned length of participation in the study for each participant is a maximum of approximately 70 weeks. This study will consist of 4 parts:

- Part 1: 10-week screening period
- Part 2: 26-week randomized controlled period (RCP)
- Part 3: 26-week open-label period
- Part 4: 8-week follow-up period (only for participants who do not roll into a long-term extension study)

Informed consent (and assent, when applicable) will be obtained prior to any study-related procedures being conducted. The screening period will start once informed consent has been obtained.

See Figure 1 for the study schematic, and Section 17.1 for the schedule of activities.

**Figure 1: Study Design**

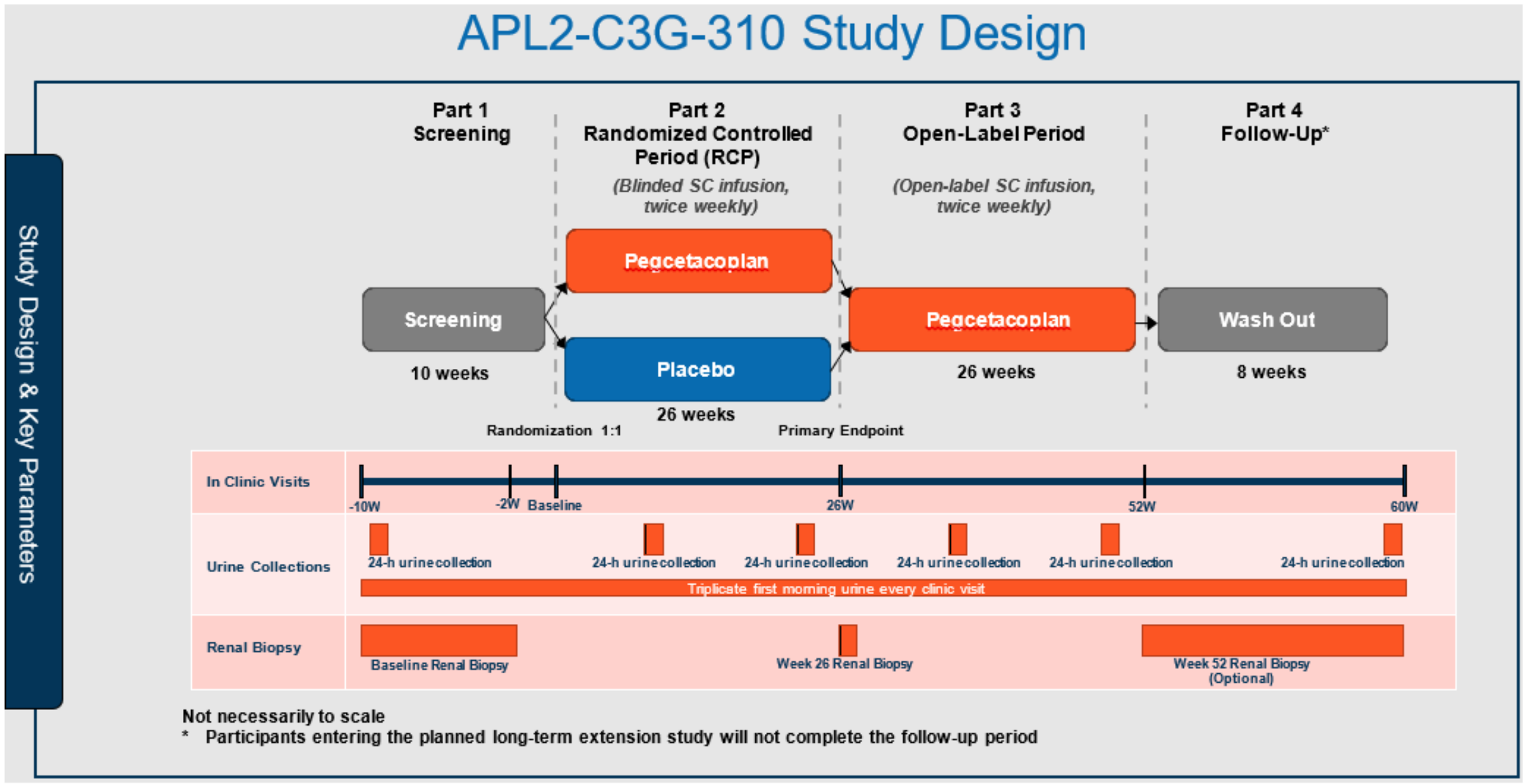


Abbreviations: SC = subcutaneous; uPCR = urine protein-to-creatinine ratio; W = week.

## 3.2. Randomization

Participants will be randomized to receive pegcetacoplan or placebo in a ratio of 1:1 via stratified permuted block randomization. The randomization will be performed centrally. To achieve balance between the arms, 2 stratification factors will be applied to the randomization. The first stratification factor examines participants with posttransplant recurrence versus nontransplant participants; at least 78 participants with C3G or IC-MPGN in a native kidney will be enrolled. This is followed by the second stratification factor, which examines participants with baseline renal biopsies (either collected during screening or a historic biopsy collected within 28 weeks prior to randomization) versus participants without baseline renal biopsies.

## 3.3. Blinding

This is a double-blinded study during the randomized controlled period. The open-label period is not blinded.

Designated blinded study staff (eg, research coordinators, nurses, technicians administering questionnaires, participants, central pathologists, assigned evaluating physicians, and the sponsor) will be blinded to treatment assignment. Access to unblinded study treatment information will be strictly limited as mandated in the study's Blinding Plan; any individuals who are unblinded are not allowed to discuss treatment and/or participant outcome with blinded study staff, including the evaluating physician. The principal investigator must be blinded to participants' treatment assignment. Although treatment in the open-label period is not blinded, investigators, study site personnel, and participants will remain blinded to the RCP treatment assignment until the blind has been broken.

## 3.4. Sample Size and Power Considerations

Approximately 80 to 100 participants, including patients with native kidney disease or post-transplant, will be randomized 1:1 to pegcetacoplan or placebo with 40 to 50 participants per arm.

Based on preliminary data from Study APL2-201, a reduction of 60% in uPCR in the pegcetacoplan group at week 26 is assumed vs a reduction of 20% in uPCR in the placebo arm, which corresponds to a mean log ratio to baseline of -0.92 vs -0.22 respectively, and a standard deviation of 0.88 (on log-scale). Based on this assumption, a sample size of 70 participants in total provides at least 90% power at 1-sided significance level of 0.025. Considering a 10% attrition to account for potential missing assessments and the impact of coronavirus disease 2019 (COVID-19), it is expected that at least 78 participants with native kidney disease should be enrolled.

A minimum of 63 participants with C3G in native kidneys will be enrolled, which is approximately 80% of the enrolled participants with native kidney disease.

## 3.5. Analysis Timing and Unblinding

The analysis of data from the RCP of the study will be performed when all participants have completed the RCP or discontinued early and all corresponding data have been entered into the database, reviewed, cleaned, and finalized, and the week 26 Analysis database locked. At that time, the sponsor analysis team will be unblinded to the treatment code, and the primary analysis will be performed, which will include all efficacy and safety analyses for the RCP.

# 4. STATISTICAL ANALYSIS SETS

## 4.1. Screened Set

The screened set includes all participants who provide written informed consent. This set will be used only for the purpose of describing participant disposition.

## 4.2. Intent-to-Treat Set

The intent-to-treat (ITT) set includes all participants who are randomized. Participants will be analyzed in the treatment arm assigned at randomization.

## 4.3. Safety Set

The safety set includes all participants who receive at least one dose of pegcetacoplan or placebo. Participants will be analyzed according to the actual treatment received. This population will be used for all safety analyses.

## 4.4. Per-protocol Set

The per-protocol (PP) set includes all participants in the ITT set who have not violated any inclusion or exclusion criteria and/or deviated from the protocol in a way that could influence their efficacy assessment. Decisions concerning the exclusion of participants from the PP set will be made and documented prior to week 26 Analysis database lock.

The review and classification of protocol deviations are described in the study's Protocol Deviation Handling Plan, where the criteria for major and minor deviations are also defined. Deviations that affect exclusion from the PP set are a subset of protocol deviations; major protocol deviations do not necessarily result in exclusion of the participant from the PP set.

## 4.5. Pharmacokinetic Set

The pharmacokinetic (PK) set includes all participants in the safety set who have at least one quantifiable post-dose concentration of pegcetacoplan (even with values below the limit of quantification [BLQ]).

## 4.6. Pharmacodynamic Set

The pharmacodynamic (PD) set includes all participants in the safety set who have at least one quantifiable post-dose PD endpoint (eg, C3, CH50, or AH50) evaluated.

# 5. STUDY PARTICIPANTS

## 5.1. Disposition of Participants

For summary of analysis sets, the number and percentage of participants screened, who failed screening, and who were included in each of the analysis sets as specified in Section 4 will be summarized and listed using the Screened Set.

For summary of disposition, the number and percentage of participants who were treated, who were ongoing with study treatment, who completed study treatment, who discontinued study treatment with a primary reason for discontinuation, who were still in the study, who completed the study, who withdrew from the study with a primary reason for withdrawal will be summarized and listed using the ITT set.

Participant disposition by region will also be provided for the ITT set.

## 5.2. Demographic and Other Baseline Characteristics

The following baseline demographic characteristics will be summarized and listed using the ITT and Safety sets (if these two sets are different, otherwise will only generate for ITT): age at screening, age group (≤ 65 years vs > 65 years, alternative grouping based on age distribution may be considered if necessary), sex, ethnicity, race, weight, height, body mass index (BMI), and blood pressure.

The following baseline disease characteristics will be summarized and listed using the ITT and Safety sets (if these two sets are different, otherwise will only generate for ITT): underlying disease based on screening biopsy (C3G including C3GN and DDD, or IC-MPGN), indication per disease-specific medical history form, potential risk factors of C3G/IC-MPGN, disease manifestations, drusen, prior kidney transplant (Y/N), time since last kidney transplant, total number of kidney transplants, prior dialysis (Y/N), baseline 24h uPCR, baseline uPCR (triplicate first-morning spot urine), baseline eGFR, annualized historical eGFR slope, baseline creatinine, baseline serum albumin, baseline serum C3, time since diagnosis of C3G/IC-MPGN, time since most recent post-transplant recurrence, and time from last kidney transplant to the most recent post-transplant recurrence.

Time since diagnosis of C3G/IC-MPGN will be calculated as:

Time since diagnosis of C3G/IC-MPGN (years) = (day 1 date – start date of indication)/365.25. Note: for native kidney participants, the source of "start date of indication" comes from the case report form's question "Start date of indication" from the disease specific medical history form. For post-transplant participant, the source of "start date of indication" comes from the derived variable "date of most recent post-transplant recurrence", which is obtained through programmatic search from medical history.

Time since last kidney transplant will be calculated as:

Time since last kidney transplant (years) = (day 1 date – date of last kidney transplant)/365.25.

Time since most recent post-transplant recurrence will be calculated as:

Time since most recent post-transplant recurrence (years) = (day 1 date – date of most recent post-transplant recurrence)/365.25.

Time from last kidney transplant to the most recent post-transplant recurrence will be calculated as:

Time from last kidney transplant to the most recent post-transplant recurrence (years) = (date of most recent post-transplant recurrence – date of last kidney transplant)/365.25.

Note in the above time since calculations, day 1 date will be replaced with randomization date if participants are not dosed.

The following baseline biopsy characteristics will be summarized and listed using the ITT and Safety sets (if these two sets are different, otherwise will only generate for ITT): C3 staining, number of glomeruli, light microscopic patterns, glomerular crescents, global sclerosis, interstitial fibrosis, tubular atrophy, deposits by electron microscopy (absent/present, characters of deposits, locations of deposits, degree of foot process effacement), total activity score, total chronicity score, and Banff score (post-transplant only).

For baseline demographic characteristics and baseline disease characteristics, additional subgroup tables will be provided separately for adults vs adolescents.

For baseline demographic characteristics, baseline disease characteristics and baseline biopsy characteristics, additional subgroup tables will be provided separately for C3G vs IC-MPGN.

## 5.3. Medical History

Medical history will be coded using the latest Medical Dictionary for Regulatory Activities (MedDRA) coding dictionary. For participants in the Safety Set, medical history will be summarized by System Organ Class (SOC) and Preferred Term (PT) and listed. Each participant will be counted only once in each SOC or SOC/PT summary.

In the summary table, medical history will be presented by decreasing frequency of participants overall within each SOC and then similarly by decreasing frequency of participants overall within each PT. In the cases of SOCs or PTs with equal frequencies, medical history will be sorted alphabetically.

Vaccination will be summarized for the Safety Set and listed. Kidney transplant history will be listed for the Safety Set.

## 5.4. Prior and Concomitant Medications

Prior and concomitant medications will be coded using the latest World Health Organization (WHO) Drug Dictionary version available. Summaries of prior and concomitant medications will be presented by Anatomical Therapeutic Class (ATC) level 2 (therapeutic main group) and preferred term with numbers and percentages by treatment groups and overall for participants in the Safety Set. A participant who takes more than one medication will be counted only once if these medications belong to the same ATC level 2 classification.

In the summary tables, prior medications and concomitant medications will be presented by decreasing frequency of participants overall within each ATC level 2 class and then similarly by decreasing frequency of participants overall within each preferred term. In cases of ATC level 2 classes or preferred terms with equal frequencies, medications will be sorted alphabetically.

Prior medications are defined as those medications that started prior to the first administration of study drug. Concomitant medications are defined as those medications taken on/after the date of first administration of study drug. Medications started before the first dose of study drug and continuing after will be considered as both prior and concomitant medications. A data listing of prior and concomitant medications will be provided for the Safety Set.

## 5.5. Prior and Concomitant Procedures

Prior and concomitant procedures will be coded using the latest MedDRA version available. Summaries of prior and concomitant procedures will be presented by SOC and PT with numbers and percentages by treatment groups and overall for participants in the Safety Set. Prior procedures are defined as those that started prior to the first administration of study drug. Concomitant procedures are defined as those that started on/after the date of first administration of study drug. Procedures that started before the first dose of study drug and continuing after will be considered as both prior and concomitant procedures. A data listing of all procedures will be provided for the Safety Set.

## 5.6. Exposure to Investigational Product

The following parameters will be summarized using the Safety Set and listed:

- Total dose administered (mg)
- Duration of treatment (days), defined as (last infusion date – first infusion date + 1)
- Number and percentage of participants who received at least one infusion
- Number and percentage of participants with any infusions missed
- Number and percentage of participants with one or more incomplete infusions
- Number and percentage of participants with any infusions interrupted

## 5.7. Measurements of Treatment Compliance

Percent compliance will be summarized using the Safety Set and listed. Compliance is calculated as follows: Compliance (%) = total number of study infusions taken / total number of expected infusions, multiplied by 100.

The number and percentage of participants who had a percentage of drug compliance range by increment of 10% (<80%, ≥ 80% - < 90%, ≥ 90 - ≤100%, and >100%) will also be summarized.

## 5.8. Protocol Deviations

All protocol deviations will be reviewed and documented before database lock. Protocol deviations are being captured in accordance with the protocol deviation management plan. They may also be identified through programmable checks of the data.

The CRO/Apellis will classify major and minor protocol deviations per the agreed Protocol Deviation Handling Plan. The Apellis study team will review the protocol deviations and their classification throughout the study and before database lock.

The number and percentage of participants with protocol deviations will be summarized by importance of deviation for ITT Set and listed.

# 6. EFFICACY ANALYSES

Efficacy analysis including primary, key secondary, additional secondary, and exploratory analysis will be performed primarily using the ITT set, with participants grouped according to the treatment assigned at randomization. Available data from all randomized participants regardless of adherence to the protocol will be included in the efficacy analysis, this includes data from participants who discontinued study drug early but continued with study assessments. All efficacy data will be listed for the ITT set.

All statistical tests will be performed at 2-sided 5% level of significance and all confidence intervals will be two-sided 95% confidence intervals.

Unless otherwise noted, the following randomization stratification factors will be adjusted for in analysis of efficacy endpoints in the overall population:

- Participants with posttransplant recurrence versus nontransplant participants
- Participants with baseline renal biopsies versus participants without baseline renal biopsies

If participants are found to have had the incorrect stratum assigned at randomization, they will be analyzed according to the randomization stratum.

For potential treatment imbalance, will check imbalance between treatment and placebo. In case there are major imbalances on important prognostic factors other than those already included in the analysis model, additional sensitivity analyses may be performed to adjust any or all of these factors if appropriate. Data will be verified for outliers. Sensitivity analyses may be performed using more robust approaches or excluding outliers.

## 6.1. Analysis Models

In general, data will be analyzed using the approaches described below.

*<u>Mixed-effect model for repeated measures (MMRM) for continuous outcomes</u>*

Longitudinal assessments for changes from baseline in continuous outcomes will be analyzed using a mixed-effect model for repeated measures (MMRM). The model will include fixed categorical effects for treatment group, visit, disease type (C3G vs. IC-MPGN), baseline immunosuppressants use (yes vs. no, to add this effect for primary endpoint analysis only), stratification factors, and the visit-by-treatment group interaction, as well as the continuous, fixed covariate of the baseline value of the endpoint. The least square (LS) means with standard errors (SEs) and 95% confidence intervals (CIs) of the change from baseline will be presented by treatment group and visit; between-treatment differences and 95% CIs and p-values will be presented by visit. LS means (±SE) will be plotted over time by treatment group.

A common unstructured covariance matrix will be used. If the model fails to converge, the following covariance structures will be fit in this order until convergence is met: (*i*) heterogenous autoregressive ARH(1), (*ii*) autoregressive AR(1), (*iii*) compound symmetry CS. The sandwich estimator (Diggle et al. 1994) will be used to estimate the standard errors of the fixed effects parameters.

*Logistic regression model for binary outcomes*

Binary outcomes will be analyzed using a logistic regression model. The model will include treatment group as independent variable and adjusted for baseline value of the variable(s) used to define the endpoint, disease type, and stratification factors. Point estimates for proportions and difference in proportions will be presented. The p-value and odds ratio of being a responder for the pegcetacoplan group to being a responder for the placebo group with associated 95% CI will be provided. In situations where event rates are low leading to convergence issue for the logistic regression model, alternative methods such as removal of covariates may be considered when appropriate.

*Analysis of covariance (ANCOVA)*

Analysis of covariance (ANCOVA) will be used to analyze C3G histologic index activity score, FACIT-Fatigue score, and KDQOL score with treatment as a fixed effect, adjusted for baseline score of the endpoint, disease type, and stratification factors. LS means will be presented for each treatment group, along with the between-treatment difference and 95% CI.

*Mixed effects model for slope analysis of continuous outcomes*

For slope analysis of continuous outcomes, a mixed effects model using the baseline and all postbaseline assessments will be used and will include treatment group, disease type, baseline immunosuppressants use (to add this effect for primary endpoint analysis only), and stratification factors as fixed effects, time (study week, continuous assuming linearity), and the time-by-treatment interaction. A common unstructured covariance matrix will be used. If the model fails to converge, the following covariance structures will be fit in this order until convergence is met: (*i*) heterogenous autoregressive ARH(1), (*ii*) autoregressive AR(1), (*iii*) compound symmetry CS. The sandwich estimator (Diggle et al. 1994) will be used to estimate the standard errors of the fixed effects parameters.

## 6.2. Multiplicity Adjustment

The primary endpoint of the study will be tested at the 2-sided 0.05 level, and if the null hypothesis for the primary endpoint is rejected, the secondary endpoints will be tested. The key secondary and additional secondary endpoints will be tested sequentially in the order in which they are presented in Section 2.2.2 and Section 2.2.3; the testing will stop once a null hypothesis is not rejected. This fixed-sequence testing procedure will ensure that trial-wise error rate is controlled to be 0.05.

## 6.3. Estimands

The primary objective of this study is to assess the efficacy of twice-weekly SC doses of pegcetacoplan compared with that of placebo in patients with primary C3G or IC-MPGN on the basis of a reduction in proteinuria.

The estimands and their attributes for the primary and all comparative key secondary and additional secondary endpoints are shown in Table 1 below. This includes strategies for addressing the following intercurrent events (ICEs):

- ProhibiRescue = use of prohibited concomitant medication specified in Protocol Section 8.3.3, or use of rescue therapies defined in Protocol Section 8.3.2
- RenalReplace = start renal replacement therapy (dialysis and/or renal transplant)
- DisctTrt = permanent discontinuation of study treatment

**Table 1: Estimands and Attributes for Primary, Key Secondary, and Additional Secondary Endpoints**

For all estimands:

A. **Population**: participants with C3G or IC-MPGN defined through the study inclusion/exclusion criteria in the ITT Set

B. **Treatment regimens of interest:**
   - Twice-weekly SC doses of pegcetacoplan for 26 weeks of treatment
   - Twice-weekly SC doses of placebo for 26 weeks of treatment

| C: Variable (or endpoint) | D: Strategies for addressing ICEs (event†: strategy‡) | E: Population-level summary |
|---|---|---|
| **Primary Estimand** | | |
| Log-transformed ratio of uPCR at week 26 compared to baseline | ProhibiRescue: hypothetical strategy<br>RenalReplace: hypothetical strategy<br>DisctTrt: treatment policy strategy | Difference in mean change of log-transformed uPCR from baseline to week 26 (measured by equal-weighted average over weeks 24, 25, and 26) between the pegcetacoplan group and the placebo group. |
| **Key Secondary Estimands (for comparative endpoints)** | | |
| The proportion of participants who meet the criteria for achieving a composite renal endpoint at week 26 | ProhibiRescue, RenalReplace, DisctTrt: composite strategy | Odds ratio of achieving a composite renal endpoint for the pegcetacoplan group to achieving a composite renal endpoint for the placebo group at week 26. |
| The proportion of participants with a reduction of at least 50% from baseline in uPCR at week 26 | ProhibiRescue, RenalReplace, DisctTrt: composite strategy | Odds ratio of achieving a reduction of at least 50% from baseline in uPCR for the pegcetacoplan group to achieving a reduction of at least 50% from baseline in uPCR for the placebo group at week 26. |
| For participants with evaluable renal biopsies, the change from baseline in the activity score of the C3G histologic index score at week 26 | ProhibiRescue: hypothetical strategy<br>RenalReplace: hypothetical strategy<br>DisctTrt: treatment policy strategy | Difference in mean change from baseline to week 26 in activity score between the pegcetacoplan group and the placebo group. |

**Table 1: Estimands and Attributes for Primary, Key Secondary, and Additional Secondary Endpoints**

| | | |
|---|---|---|
| The proportion of participants with evaluable renal biopsies showing decreases in C3c staining on renal biopsy from baseline at week 26 | ProhibiRescue, RenalReplace, DisctTrt: composite strategy | Odds ratio of showing decreases in C3c staining for the pegcetacoplan group to showing decreases in C3c staining for the placebo group at week 26. |
| Change from baseline in eGFR at week 26 | ProhibiRescue: hypothetical strategy<br>RenalReplace: hypothetical strategy<br>DisctTrt: treatment policy strategy | Difference in mean change of eGFR from baseline to week 26 between the pegcetacoplan group and the placebo group. |
| **Additional Secondary Estimands (for comparative endpoints)** | | |
| The proportion of participants achieving proteinuria <1 g/day at week 24 | ProhibiRescue, RenalReplace, DisctTrt: composite strategy | Odds ratio of achieving proteinuria <1 g/day for the pegcetacoplan group to achieving proteinuria <1 g/day for the placebo group at week 24. |
| For participants with serum albumin levels below LLN at baseline, the proportion of participants with normalization of serum albumin levels at week 26 | ProhibiRescue, RenalReplace, DisctTrt: composite strategy | Odds ratio of achieving normalization of serum albumin for the pegcetacoplan group to achieving normalization of serum albumin for the placebo group at week 26. |
| For participants with serum C3 levels below the LLN at baseline, the proportion of participants with serum C3 levels above the LLN at week 26 | ProhibiRescue, RenalReplace, DisctTrt: composite strategy | Odds ratio of achieving serum C3 levels above the LLN for the pegcetacoplan group to achieving serum C3 levels above the LLN for the placebo group at week 26. |
| The change from baseline in FACIT–Fatigue Scale score | ProhibiRescue: hypothetical strategy<br>RenalReplace: hypothetical strategy<br>DisctTrt: treatment policy strategy | Difference in mean change from baseline to week 26 in FACIT-Fatigue Scale score between the pegcetacoplan group and the placebo group. |
| The change from baseline in KDQOL score | ProhibiRescue: hypothetical strategy<br>RenalReplace: hypothetical strategy<br>DisctTrt: treatment policy strategy | Difference in mean change from baseline in KDQOL score between the pegcetacoplan group and the placebo group. |

**Table 1: Estimands and Attributes for Primary, Key Secondary, and Additional Secondary Endpoints**

| |
|---|
| †ICE definitions:<br>• ProhibiRescue = use of prohibited concomitant medication specified in Protocol Section 8.3.3, or use of rescue therapies defined in Protocol Section 8.3.2<br>• RenalReplace = start renal replacement therapy (dialysis and/or renal transplant)<br>• DisctTrt = permanent discontinuation of study treatment<br><br>‡Strategies**:**<br>• Composite strategy: the endpoint status at or after the initiation of the ICEs will be regarded as non-responder.<br>• Hypothetical strategy: (for ICEs due to ProhibiRescue) all measurements after the ICEs will be set to missing. Missing data resulting from the ICEs will be imputed using copy reference imputation. (for ICEs due to RenalReplace) all measurements after the ICEs will be set to missing. Missing data resulting from the ICEs will be imputed based on the worst change of all participants across visits plus a random error.<br>• Treatment policy strategy: all measurements after the ICEs will be used as is. Missing data resulting from the ICEs will be imputed using copy reference imputation. |

## 6.4. Analyses of Primary Efficacy Endpoint

The primary efficacy endpoint is the log-transformed ratio of uPCR at week 26 compared to baseline. The null ($H_{1,0}$) and alternative ($H_{1,1}$) hypotheses for the primary efficacy analysis are:

$H_{1,0}$: There is no difference in log-transformed ratio of uPCR at week 26 compared to baseline between the pegcetacoplan and placebo treatment groups.

$H_{1,1}$: There is a difference in log-transformed ratio of uPCR at week 26 compared to baseline between the pegcetacoplan and placebo treatment groups.

uPCR values are expected to be highly skewed so that the values will be natural log-transformed in analysis and presentation. For the primary efficacy endpoint, sensitivity analyses (Section 6.4.2), supplemental analyses (Section 6.4.3), and subgroup analyses (Section 6.4.4) will be performed.

### 6.4.1. Main Analysis of Primary Efficacy Endpoint

The primary endpoint will be analyzed in the ITT set with participants grouped according to the treatment assigned at randomization.

Baseline uPCR value will be calculated as the average of the uPCR measurements from at least 6 of the 9 first-morning spot urine (FMU) samples collected between the start of screening and day 1, inclusive. The uPCR values used to calculate baseline should include those from the samples collected on day −2, day −1, and before dosing on day 1. In situations where less than 6 samples or more than 9 samples were collected, the average of all collected samples will be used for baseline derivation. For scheduled visits where 3 collections are expected for calculating averages, if there are missing collections, the average will be based on those available collections; if there are more than 3 collections available (e.g., additional unscheduled collections), the selection of 3 collections to be used for average calculation will follow data handling conventions as specified in Section 13.4.

An MMRM model will be used to analyze the primary endpoint. The model will include fixed categorical effects for treatment group, visit, disease type (C3G vs. IC-MPGN), baseline immunosuppressants use (yes vs. no), stratification factors, and the visit-by-treatment group interactions, as well as the continuous, fixed covariate of baseline log-transformed uPCR. As the primary estimate, the difference between treatment groups using a composite contrast of equal-weighted average over weeks 24, 25, and 26 will be estimated (refer to sample SAS code section for more details) with its 95% CI and corresponding p-value. Further details on the presentation of the results are described in Section 6.1above. In addition, the geometric means and geometric mean ratios relative to baseline will also be presented after converting the log-transformed values back to the original unit.

ICEs strategy and handling are listed in Table 1. Imputation for the non-monotone missing pattern (ie, arbitrary missing pattern) will be performed prior to the multiple imputation for the monotone missing pattern (ie, where a missing uPCR measurement at a visit for a participant implies that uPCR measurements at all subsequent visits for that participant are missing).

For the non-monotone missing pattern, missing value(s) between two visits with uPCR measurements will be imputed using the MCMC method based on the MAR assumption, which was first proposed by Li (1988) and Liu (1993) described the algorithm. Multiple imputation will then be carried out for monotone missing pattern. For ICEs due to renal replacement therapy (dialysis and/or renal transplant), missing data will be imputed based on the worst change of all participants across visits plus a random error. For other ICEs, missing data will be imputed based on copy reference imputation:

- Participants with monotone missing data in the placebo arm will have missing data imputed based on the observed values in the placebo arm.
- For the active treatment arm, participants with monotone missing data due to prohibited medication, rescue therapies, or treatment discontinuation will have missing data imputed based on the placebo arm.
- For the active treatment arm, participants with monotone missing data that are not due to prohibited medication, rescue therapies, or treatment discontinuation will have missing data imputed based on the observed values in the active treatment arm.

The number and percent of participants without monotone missing data (separated further by non-monotone missing vs. complete data), the number of participants with monotone missing data and a breakdown of reasons will be reported.

The imputation method will be implemented in SAS using the three standard steps to generate inference from imputed data: imputation step, analysis step, and pooling step. The randomization seed to be used is 230815.

- The missing data are filled in 100 times to generate 100 complete datasets.
- The 100 complete datasets are analyzed by using the MMRM approach described above.
- The results from the 100 complete datasets are combined for inference using Rubin’s rule.

The observed values for uPCR will be summarized by treatment group and visit. Summaries will present the descriptive statistics for baseline, absolute values, change and percentage change from baseline data by visit.

The mean change from baseline in uPCR (±SE) will be plotted over time by treatment group.

All uPCR data will be listed for the ITT set.

### 6.4.2. Sensitivity Analyses of Primary Efficacy Endpoint

Sensitivity analyses will be performed to evaluate the robustness of the primary analysis results. Analyses will be performed for the overall population based on the outcome of the primary analyses including all participants in the ITT set.

The following sensitivity analysis will be performed, as appropriate, using the same statistical approach as the one used in the primary analysis:

- Imputation based on MAR: Participants with missing data, regardless of the reason being the ICEs or not, will be handled implicitly within the MMRM analysis under the assumption of missing at random (MAR).
- Tipping point analysis: Participants with a monotone missing data pattern due to the ICEs will have missing data after the last assessment explicitly imputed by multiple imputation using a tipping point analysis method assuming missing not at random (MNAR).

The tipping point analysis method (a delta-adjusted stress testing approach) will be implemented for the active treatment arm under the MNAR assumption by searching for a tipping point that reverses the conclusion regarding positive treatment effect (O'Kelly and Ratitch 2014).
The following strategy will be used:

- For the control arm, participants will have missing data imputed based on the observed values in the control arm.
- For the active treatment arm, participants with monotone missing data that are not due to the ICEs will have missing data imputed based on the observed values in the active treatment arm.
- For the active treatment arm, participants with monotone missing data due to the ICEs will have missing data imputed based on the available values (observed values and values imputed for non-monotone missing data) in the active treatment arm with a shift parameter added to the imputed values. Multiple imputation will be implemented with the shift parameter allocated to the missing data point(s) proportionally across timepoints. (eg, for a shift parameter of 0.26 at week 26, incremental shifts of 0.04 would apply to week 4, …, and week 24, respectively, then a shift of 0.01 would apply to week 25 and week 26, respectively. 0.04 × 6 visits+ 0.01 × 2 visits= 0.26) The range of the shift parameters will be from 0.13 to 0.65 by increments of 0.13 for week 26. A tipping point may not exist within reasonable clinical assumptions.

To generate inference from imputed data, the same three standard steps as described in the main analysis will be implemented.

### 6.4.3. Supplemental Analyses of Primary Efficacy Endpoint

#### 6.4.3.1. Per Protocol Set

The main analysis described in Section 6.4.1 will be repeated using the PP set to investigate the impact of changing the population in the estimand. Missing data (regardless of the reason being the ICEs or not) will be handled implicitly within the MMRM analysis under the assumption of missing at random (MAR).

#### 6.4.3.2. Slope Analysis

The mean rate of change (ie, slope) in observed log-transformed uPCR values will be compared between treatment groups using the linear mixed effects model for slope analysis of continuous outcomes described above in Section 6.1. The mean rate of change (slope), standard errors, and confidence interval will be estimated for the baseline to week 26 period for each treatment group. In addition, the estimated difference in slopes between the treatment groups along with the 95% CIs and p-value will be reported. Strategies for addressing ICEs are the same as the ones described in Section 6.4.1.

### 6.4.4. Subgroup Analyses of Primary Efficacy Endpoint

Subgroup analyses will be performed to evaluate the consistency of the primary analysis results across subgroups defined by demographic and baseline characteristics. Analyses will be performed for the primary efficacy endpoint for each of the following subgroups (as appropriate per actual subgroup sample size, levels with low sample size may be pooled to allow for an analysis to be conducted):

- Age group: adolescent, adult
- Sex: male, female
- Race: Caucasian, non-Caucasian
- Geographic region: United States, Rest of World
- Disease type: C3G, IC-MPGN
- Transplant history: nontransplant, posttransplant
- Baseline FMU uPCR: <3000 mg/g, ≥3000 mg/g
- Baseline eGFR: <60 mL/min/1.73 $m^2$, ≥60 mL/min/1.73 $m^2$
- Baseline C3 level: normal, below the LLN
- Baseline immunosuppressants use: yes, no (based on ATC level 2 of “IMMUNOSUPPRESSANTS” and/or “CORTICOSTEROIDS FOR SYSTEMIC USE”)

The estimated treatment effects and corresponding 95% CIs and p-values from the models will be displayed graphically for each level of the subgroups specified via forest plots.

## 6.5. Analyses of Key Secondary Efficacy Endpoints

The following subsections describe the analyses of the key secondary efficacy endpoints of the study. All analyses of the key secondary efficacy endpoints will be done primarily using the ITT set.

### 6.5.1. The Proportion of Participants Who Meet the Criteria for Achieving a Composite Renal Endpoint

The key secondary efficacy endpoint of the proportion of participants who meet the criteria for achieving a composite renal endpoint at week 26 will be examined with the following null ($H_{2,0}$) and alternative ($H_{2,1}$) hypotheses:

$H_{2,0}$: There is no difference in proportion of participants who meet the criteria for achieving a composite renal endpoint at week 26 between the pegcetacoplan and placebo treatment groups.

$H_{2,1}$: There is a difference in proportion of participants who meet the criteria for achieving a composite renal endpoint at week 26 between the pegcetacoplan and placebo treatment groups.

A participant meets the requirements of the composite renal endpoint if they satisfy:

(1) A stable or improved eGFR compared to baseline (≤15% reduction in eGFR), and
(2) A ≥50% reduction in uPCR compared to baseline.

For requirement (1), the eGFR status at week 26 will be calculated using the following steps:

1. Baseline eGFR value will be calculated using the last non-missing assessment prior to first dose.
2. Week 26 eGFR value will be calculated based on the week 26 assessment result.
3. Each participant will be categorized as either a "stable or improved" or not "stable or improved" according to whether the change from baseline in eGFR calculated from steps 1 and 2 is a reduction of no more than 15%.

For requirement (2), the uPCR response status at week 26 will be calculated using the following steps:

1. Baseline uPCR value will be calculated as the average of the uPCR measurements from at least 6 of the 9 FMU samples collected between the start of screening and day 1, inclusive.
2. Week 26 uPCR value will be calculated as the average of the uPCR measurements from at least 6 of the 9 FMU samples collected in week 24, week 25, and week 26.
3. Each participant will be categorized as either a success or a failure according to whether the change from baseline in uPCR calculated from steps 1 and 2 is a reduction of at least 50%.

#### 6.5.1.1. Main Analysis

The key secondary endpoint will be analyzed in the ITT set with participants grouped according to the treatment assigned at randomization.

A logistic model will be used to analyze the key secondary endpoint. The model will include treatment group as the independent variable and adjusted for baseline eGFR values, baseline log-transformed uPCR values, disease type (C3G vs IC-MPGN), and stratification factors. Further details on the presentation of the results are described in Section 6.1.

A composite strategy will be used for addressing the ICEs listed in Table 1. In the composite strategy, the composite renal endpoint status at or after the occurrence of any of these ICEs will be regarded as non-responder. Additionally, participants with missing eGFR and/or uPCR values at week 26 for reasons other than those listed as ICEs will be regarded as non-responder.

The numbers and proportion of participants who meet the criteria for achieving a composite renal endpoint at week 26 will be tabulated by treatment group, including detailed breakdown on each of the two requirements.

All composite renal endpoint achievement data will be listed for the ITT set.

#### 6.5.1.2. Supplemental Analyses

##### 6.5.1.2.1. Per Protocol Set

The main analysis described in Section 6.5.1.1 will be repeated using the PP set to investigate the impact of changing the population in the estimand.

#### 6.5.1.3. Subgroup Analyses

Subgroup analyses will be performed for this key secondary endpoint to evaluate the consistency of the results across the subgroups specified in Section 6.4.4 above.

### 6.5.2. The Proportion of Participants With a Reduction of at least 50% from Baseline in uPCR

The key secondary efficacy endpoint of the proportion of participants with a reduction of at least 50% from baseline in uPCR at week 26 will be examined with the following null ($H_{3,0}$) and alternative ($H_{3,1}$) hypotheses:

$H_{3,0}$: There is no difference in proportion of participants who have a reduction of at least 50% from baseline in uPCR at week 26 between the pegcetacoplan and placebo treatment groups.

$H_{3,1}$: There is a difference in proportion of participants who have a reduction of at least 50% from baseline in uPCR at week 26 between the pegcetacoplan and placebo treatment groups.

The uPCR response status at week 26 will be calculated using the following steps:

1. Baseline uPCR value will be calculated as the average of the uPCR measurements from at least 6 of the 9 FMU samples collected between the start of screening and day 1, inclusive.
2. Week 26 uPCR value will be calculated as the average of the uPCR measurements from at least 6 of the 9 FMU samples collected in week 24, week 25, and week 26.
3. Each participant will be categorized as either a success or a failure according to whether the change from baseline in uPCR calculated from steps 1 and 2 is a reduction of at least 50%.

#### 6.5.2.1. Main Analysis

The key secondary endpoint will be analyzed in the ITT set with participants grouped according to the treatment assigned at randomization.

A logistic model will be used to analyze the key secondary endpoint. The model will include treatment group as the independent variable and adjusted for baseline log-transformed uPCR values, disease type (C3G vs IC-MPGN), and stratification factors. Further details on the presentation of the results are described in Section 6.1 above. Strategies for addressing ICEs are the same as the ones described in Section 6.5.1.1.

The numbers and proportion of participants with a reduction of at least 50% from baseline at week 26 will be tabulated by treatment group.

All the uPCR response status data will be listed for the ITT set.

#### 6.5.2.2. Supplemental Analyses

##### 6.5.2.2.1. Per Protocol Set

The main analysis described in Section 6.5.2.1 will be repeated using the PP set to investigate the impact of changing the population in the estimand.

#### 6.5.2.3. Subgroup Analyses

Subgroup analyses will be performed for this key secondary endpoint to evaluate the consistency of the results across the subgroups specified in Section 6.4.4 above.

### 6.5.3. Change From Baseline in the Activity Score of the C3G Histologic Index Score

For participants with evaluable renal biopsies, the key secondary efficacy endpoint is the change from baseline in the activity score of the C3G histologic index score at week 26. It will be examined with the following null ($H_{4,0}$) and alternative ($H_{4,1}$) hypotheses:

$H_{4,0}$: There is no difference in mean change from baseline to week 26 in activity score between the pegcetacoplan and placebo treatment groups.

$H_{4,1}$: There is a difference in mean change from baseline to week 26 in activity score between the pegcetacoplan and placebo treatment groups.

#### 6.5.3.1. Main Analysis

The key secondary endpoint will be analyzed in the ITT set with participants grouped according to the treatment assigned at randomization.

An ANCOVA model will be used to analyze the key secondary endpoint. The model will include treatment as a fixed effect, adjusted for baseline C3G histologic index activity score, disease type (C3G vs IC-MPGN), and stratification factors. Further details on the presentation of the results are described in Section 6.1. Strategies for addressing ICEs are the same as the ones described in Section 6.4.1. The randomization seed to be used is 240417. Note that because adolescents are not required to provide week 26 biopsies, this endpoint will be analyzed based on adult participants only.

The observed values for activity score will be summarized by treatment group and visit. Summaries will present the descriptive statistics for baseline, absolute values, change and percentage change from baseline data by visit. In situations where an adjudication is needed (the difference of total activity score >3.5 between the primary pathologist and the secondary pathologist), the final score based on adjudication will be used for analysis; if an adjudication is not needed (the difference of total activity score <=3.5 between the primary pathologist and the secondary pathologist), the average of the two pathologists' scores will be used for analysis.

All activity score data will be listed for the ITT set.

#### 6.5.3.2. Sensitivity Analyses

The tipping point analysis method described in Section 6.4.2 will be repeated for the key secondary endpoint. The range of the shift parameters will be from 1.3 to 6.5 by increments of 1.3.

#### 6.5.3.3. Supplemental Analyses

##### 6.5.3.3.1. Per Protocol Set

The main analysis described in Section 6.5.3.1 will be repeated using the PP set to investigate the impact of changing the population in the estimand.

#### 6.5.3.4. Subgroup Analyses

Subgroup analyses will be performed for this key secondary endpoint to evaluate the consistency of the results across the subgroups specified in Section 6.4.4 above.

### 6.5.4. The Proportion of Participants Showing Decreases in C3c Staining From Baseline

For participants with evaluable renal biopsies, the key secondary efficacy endpoint of the proportion of participants showing decreases in C3c staining (defined as decrease of at least 2 orders of magnitude of intensity) from baseline at week 26 will be examined with the following null ($H_{5,0}$) and alternative ($H_{5,1}$) hypotheses:

$H_{5,0}$: There is no difference in proportion of participants who show decreases in C3c staining from baseline at week 26 between the pegcetacoplan and placebo treatment groups.

$H_{5,1}$: There is a difference in proportion of participants who show decreases in C3c staining from baseline at week 26 between the pegcetacoplan and placebo treatment groups.

#### 6.5.4.1. Main Analysis

The key secondary endpoint will be analyzed in the ITT set with participants grouped according to the treatment assigned at randomization.

A logistic model will be used to analyze the key secondary endpoint. The model will include treatment group as the independent variable and adjusted for baseline C3c staining, disease type (C3G vs IC-MPGN), and stratification factors. Further details on the presentation of the results are described in Section 6.1. Strategies for addressing ICEs are the same as the ones described in Section 6.5.1.1. Since adolescents are not required to provide week 26 biopsies, this endpoint will be analyzed based on adult participants only.

The numbers and proportion of participants who meet the criteria of showing decreases in C3c staining at week 26 will be tabulated by treatment group. A shift table of changes in C3c staining from baseline to week 26 will also be tabulated.

All C3c staining data will be listed for the ITT set.

#### 6.5.4.2. Supplemental Analyses

##### 6.5.4.2.1. Per Protocol Set

The main analysis described in Section 6.5.4.1 will be repeated using the PP set to investigate the impact of changing the population in the estimand.

#### 6.5.4.3. Subgroup Analyses

Subgroup analyses will be performed for this key secondary endpoint to evaluate the consistency of the results across the subgroups specified in Section 6.4.4.

### 6.5.5. Change From Baseline in eGFR

The key secondary efficacy endpoint of change from baseline in eGFR at week 26 will be examined with the following null ($H_{6,0}$) and alternative ($H_{6,1}$) hypotheses:

$H_{6,0}$: There is no difference in mean change from baseline to week 26 in eGFR between the pegcetacoplan and placebo treatment groups.

$H_{6,1}$: There is a difference in mean change from baseline to week 26 in eGFR between the pegcetacoplan and placebo treatment groups.

#### 6.5.5.1. Main Analysis

The key secondary endpoint will be analyzed in the ITT set with participants grouped according to the treatment assigned at randomization.

A MMRM model will be used to analyze the key secondary endpoint. The model will include fixed categorical effects for treatment group, visit, disease type (C3G vs IC-MPGN), stratification factors, and the visit-by-treatment group interactions, as well as the continuous, fixed covariate of baseline eGFR values. Further details on the presentation of the results are described in Section 6.1. Strategies for addressing ICEs are the same as the ones described in Section 6.4.1. The randomization seed to be used is 210809.

The observed values for eGFR will be summarized by treatment group and visit. Summaries will present the descriptive statistics for baseline, absolute values, change and percentage change from baseline data by visit.

The mean change from baseline in eGFR (±SE) will be plotted over time by treatment group.

All eGFR data will be listed for the ITT set.

#### 6.5.5.2. Sensitivity Analyses

The two sensitivity analyses method described in Section 6.4.2 will be repeated for the key secondary endpoint. For tipping point analysis, the range of the shift parameters will be from −1.3 to −6.5 by increments of −1.3.

#### 6.5.5.3. Supplemental Analyses

##### 6.5.5.3.1. Per Protocol Set

The main analysis described in Section 6.5.5.1 will be repeated using the PP set to investigate the impact of changing the population in the estimand. Missing data (regardless of the reason being the ICEs or not) will be handled implicitly within the MMRM analysis under the assumption of missing at random (MAR).

##### 6.5.5.3.2. Slope Analysis

The mean rate of change (ie, slope) in observed eGFR values will be compared between treatment groups using the linear mixed effects model for slope analysis of continuous outcomes described above in Section 6.1. The mean rate of change (slope), standard errors, and confidence interval will be estimated for the baseline to week 26 period for each treatment group.
In addition, the estimated difference in slopes between the treatment groups along with the 95% CIs and p-value will be reported.

#### 6.5.5.4. Subgroup Analyses

Subgroup analyses will be performed for this key secondary endpoint to evaluate the consistency of the results across the subgroups specified in Section 6.4.4.

## 6.6. Analyses of Additional Secondary Efficacy Endpoints

The following subsections describe the analyses of the additional secondary efficacy endpoints of the study. All analyses of the additional secondary efficacy endpoints will be done on the ITT set.

### 6.6.1. The Proportion of Participants Achieving Proteinuria <1 g/day

The additional secondary efficacy endpoint of the proportion of participants achieving proteinuria <1 g/day (assessed by 24-hour urine protein) at week 24 will be examined with the following null ($H_{7,0}$) and alternative ($H_{7,1}$) hypotheses:

$H_{7,0}$: There is no difference in proportion of participants who achieve proteinuria <1 g/day at week 24 between the pegcetacoplan and placebo treatment groups.

$H_{7,1}$: There is a difference in proportion of participants who achieve proteinuria <1 g/day at week 24 between the pegcetacoplan and placebo treatment groups.

The additional secondary endpoint will be analyzed in the ITT set with participants grouped according to the treatment assigned at randomization.

A logistic model will be used to analyze the additional secondary endpoint. The model will include treatment group as the independent variable and adjusted for baseline proteinuria values, disease type (C3G vs IC-MPGN), and stratification factors. Further details on the presentation of the results are described in Section 6.1. Strategies for addressing ICEs are the same as the ones described in Section 6.5.1.1.

The numbers and proportion of participants achieving proteinuria <1 g/day at week 24 will be tabulated by treatment group.

All 24-hour urine protein data will be listed for the ITT set.

### 6.6.2. The Proportion of Participants With Normalization of Serum Albumin Levels

For participants with serum albumin levels below LLN at baseline, the additional secondary efficacy endpoint of the proportion of participants with normalization of serum albumin levels at week 26 will be examined with the following null ($H_{8,0}$) and alternative ($H_{8,1}$) hypotheses:

$H_{8,0}$: There is no difference in proportion of participants who achieve normalization of serum albumin levels at week 26 between the pegcetacoplan and placebo treatment groups.

$H_{8,1}$: There is a difference in proportion of participants who achieve normalization of serum albumin levels at week 26 between the pegcetacoplan and placebo treatment groups.

The serum albumin levels status at week 26 will be calculated using the following steps:

1. Baseline serum albumin value will be calculated as the average of up to 2 serum albumin measurements preceding and including day 1. Participants will be included in the denominator of this calculation only if the baseline albumin value is below the LLN.
2. Week 26 serum albumin values will be calculated as the average of up to 2 serum albumin measurements preceding and including week 26, no earlier than week 20 measurement.

3. Each participant will be categorized as either a success or a failure according to whether the week 26 serum albumin values as calculated in Step 2. Values greater than or equal to the LLN will be categorized as a success; values less than the LLN will be categorized as a failure.

The additional secondary endpoint will be analyzed in the ITT set with participants grouped according to the treatment assigned at randomization.

A logistic model will be used to analyze the additional secondary endpoint. The model will include treatment group as the independent variable and adjusted for baseline albumin value, disease type (C3G vs IC-MPGN), and stratification factors. Further details on the presentation of the results are described in Section 6.1. Strategies for addressing ICEs are the same as the ones described in Section 6.5.1.1.

For participants with serum albumin levels below the LLN at baseline, the numbers and proportion of participants with normalization of serum albumin levels at week 26 will be tabulated by treatment group. The observed values for serum albumin will be summarized by treatment group and visit.

All serum albumin data will be listed for the ITT set.

### 6.6.3. The Proportion of Participants With Serum C3 Levels Above the LLN

For participants with serum C3 levels below the LLN at baseline, the additional secondary efficacy endpoint of the proportion of participants with serum C3 levels above the LLN at week 26 will be examined with the following null ($H_{9,0}$) and alternative ($H_{9,1}$) hypotheses:

$H_{9,0}$: There is no difference in proportion of participants with serum C3 levels above the LLN at week 26 between the pegcetacoplan and placebo treatment groups.

$H_{9,1}$: There is a difference in proportion of participants with serum C3 levels above the LLN at week 26 between the pegcetacoplan and placebo treatment groups.

The serum C3 levels status at week 26 will be calculated using the following steps:

1. Baseline serum C3 value will be calculated as the average of up to 2 serum C3 measurements preceding and including day 1. Participants will only be included in the denominator of this calculation if the baseline C3 value is below the LLN.
2. Week 26 serum C3 value will be calculated as the average of up to 2 serum C3 measurements preceding and including week 26, no earlier than week 20 measurement.
3. Each participant will be categorized as either a success or a failure based on whether the week 26 serum C3 value as calculated in Step 2. Values greater than or equal to the LLN will be categorized as a success; values less than the LLN will be categorized as a failure.

The additional secondary endpoint will be analyzed in the ITT set with participants grouped according to the treatment assigned at randomization.

A logistic model will be used to analyze the additional secondary endpoint. The model will include treatment group as the independent variable and adjusted for baseline serum C3 level, disease type (C3G vs IC-MPGN), and stratification factors.

Further details on the presentation of the results are described in Section 6.1. Strategies for addressing ICEs are the same as the ones described in Section 6.5.1.1.

For participants with low serum C3 levels at baseline, the numbers and proportion of participants with serum C3 levels above the LLN at week 26 will be tabulated by treatment group. The observed values for serum C3 will be summarized by treatment group and visit.

All serum C3 level data will be listed for the ITT set.

### 6.6.4. Change From Baseline in FACIT-Fatigue Scale Score

The additional secondary efficacy endpoint is the FACIT-Fatigue score at week 26. It will be examined with the following null ($H_{10,0}$) and alternative ($H_{10,1}$) hypotheses:

$H_{10,0}$: There is no difference in mean change from baseline to week 26 in FACIT-Fatigue score between the pegcetacoplan and placebo treatment groups.

$H_{10,1}$: There is a difference in mean change from baseline to week 26 in FACIT-Fatigue score between the pegcetacoplan and placebo treatment groups.

The additional secondary endpoint will be analyzed in the ITT set with participants grouped according to the treatment assigned at randomization.

The FACIT-Fatigue score is constructed as the total score of the 13 questions. Each question has 5 possible responses as "Not at all" (0), "A little bit" (1), "Somewhat" (2), "Quite a bit" (3) and "Very much" (4). Before calculating the total score, some responses are reversed to ensure that the higher score corresponds to a higher quality of life.

An ANCOVA model will be used to analyze the additional secondary endpoint. The model will include treatment as a fixed effect, adjusted for baseline FACIT-Fatigue score, disease type (C3G vs IC-MPGN), the stratification factor of transplant status, and age group (adults vs. adolescents). Further details on the presentation of the results are described in Section 6.1. Strategies for addressing ICEs are the same as the ones described in Section 6.4.1. The randomization seed to be used is 191224.

The observed values for FACIT-Fatigue score will be summarized by treatment group and visit. Summaries will present the descriptive statistics for baseline, absolute values, change and percentage change from baseline data by visit. Note that the FACIT-Fatigue questionnaires have two versions, one for adults and one for adolescents. The summaries will be pooled together regardless of the different versions. Additional analysis on modeling the adults and adolescents separately will also be performed.

All FACIT-Fatigue score data will be listed for the ITT set.

### 6.6.5. Change From Baseline in KDQOL Score

The additional secondary efficacy endpoint is the KDQOL score at week 26. It will be examined with the following null ($H_{11,0}$) and alternative ($H_{11,1}$) hypotheses:

$H_{11,0}$: There is no difference in mean change from baseline to week 26 in KDQOL score between the pegcetacoplan and placebo treatment groups.

$H_{11,1}$: There is a difference in mean change from baseline to week 26 in KDQOL score between the pegcetacoplan and placebo treatment groups.

The additional secondary endpoint will be analyzed in the ITT set with participants grouped according to the treatment assigned at randomization.

The KDQOL score is constructed as the KDQOL-36 Summary Score (KSS) by averaging the 24 items from Burden of Kidney Disease, Symptoms and Problems of Kidney Disease, and Effects of Kidney Disease on 0-100 scale (Peipert et al. 2019).

An ANCOVA model will be used to analyze the additional secondary endpoint. The model will include treatment as a fixed effect, adjusted for baseline KDQOL score, disease type (C3G vs IC-MPGN), and stratification factors. Further details on the presentation of the results are described in Section 6.1. Strategies for addressing ICEs are the same as the ones described in Section 6.4.1. The randomization seed to be used is 180511.

The observed values for KDQOL score will be summarized by treatment group and visit. Summaries will present the descriptive statistics for baseline, absolute values, change and percentage change from baseline data by visit.

All KDQOL score data will be listed for the ITT set.

## 6.7. Analyses of Exploratory Efficacy Endpoints

All primary, key secondary, and additional secondary endpoints will be evaluated at week 52 as exploratory efficacy endpoints. In addition, the following exploratory endpoints will also be evaluated. Note that this SAP focuses on the analysis of the randomized controlled period (RCP) data only. The analysis for interim data post-week 26 at the time of the datacut will be detailed in an SAP addendum.

### 6.7.1. The Change From Baseline in uPCR Using the 24-Hour Urine Collections

The observed values for uPCR measured by 24-hour urine collections will be summarized by treatment group and visit using ITT Set. Summaries will present the descriptive statistics for baseline, absolute values, change and percentage change from baseline data by visit.

An MMRM model will be used to analyze the exploratory efficacy endpoint up to week 24. The model will include fixed categorical effects for treatment group, visit, disease type (C3G vs IC-MPGN), stratification factors, and the visit-by-treatment group interactions, as well as the continuous, fixed covariate of baseline 24-hour uPCR values. Further details on the presentation of the results are described in Section 6.1. No multiple imputations will be applied. ICE strategies will be simplified where missing data after the ICEs will be imputed implicitly within the MMRM in a hypothetical strategy under the assumption of MAR.

All 24-hour uPCR data will be listed for the ITT set.

### 6.7.2. The Annual Rate of Change From up to 3 Years Prior to Screening in eGFR

A generalized linear mixed model will be used to analyze the exploratory efficacy endpoint from up to 3 years prior to screening to the end of the RCP period. The model will include fixed effect for disease type, stratification factors, a common intercept, a pre-treatment (either pegcetacoplan or placebo) slope, and a change in the slope by arm after treatment. Random effects include the individual intercept and slope, and the random errors are i.i.d. normally distributed. The eGFR slope prior to treatment, the slope after pegcetacoplan treatment and the difference comparing to pre-treatment slope, the slope after placebo treatment and the difference comparing to pre-treatment slope, as well as the difference between post-pegcetacoplan slope vs. post-placebo slope, will be summarized along with their 95% CIs and p-values using the ITT set.

In situations where for a certain historical collection, the eGFR value was not collected but the components for calculating it (such as serum creatinine, age, height, etc.) were collected, then the eGFR value will be calculated based on the formula (CKD-EPI creatinine equation for adults or the Bedside Schwartz equation for adolescents) specified in protocol.

### 6.7.3. The Proportion of Participants With Reductions From Baseline in Proteinuria of at least 30%

The proportion of participants with reductions from baseline in proteinuria (measured by triplicate FMU uPCR) of at least 30% at week 26 will be summarized using ITT set. The baseline and week 26 uPCR calculation will be handled the same way as in Section 6.5.1.

A logistic model will be used to analyze the exploratory efficacy endpoint. The model will include treatment group as the independent variable and adjusted for baseline log-transformed uPCR values, disease type (C3G vs IC-MPGN), and stratification factors. Further details on the presentation of the results are described in Section 6.1. ICE strategies will be the same as those specified in Section 6.5.1.1.

A data listing for each participant's reduction status at week 26 will be provided using ITT set.

### 6.7.4. The Proportion of Participants With Normalization of Proteinuria

The proportion of participants with normalization of proteinuria (measured by triplicate FMU uPCR) at week 26 will be summarized using ITT set. The baseline and week 26 uPCR calculation will be handled the same way as in Section 6.5.1. Normalization of proteinuria is defined as test result values within the normal reference range.

A logistic model will be used to analyze the exploratory efficacy endpoint. The model will include treatment group as the independent variable and adjusted for baseline log-transformed uPCR values, disease type (C3G vs IC-MPGN), and stratification factors. Further details on the presentation of the results are described in Section 6.1. ICE strategies will be the same as those specified in Section 6.5.1.1.

A data listing for each participant's normalization status at week 26 be provided using ITT set.

### 6.7.5. Time to 50% Reduction in uPCR With a Stable or Improved eGFR

Time to 50% reduction in uPCR with a stable or improved eGFR (a.k.a., achieving the composite renal endpoint as defined in Section 6.5.1) will be summarized using ITT set with the Kaplan-Meier method (Kaplan and Meier 1958), using plots and presentation of median (and 95% CIs) for time-to-event. Participants who did not have the event at or prior to Week 26 in study will be censored at the minimum of the last date of contact or their treatment discontinuation date, or their Week 26 visit or first dose date+182 if Week 26 is missing. Participants who were randomized but did not receive any treatment will be censored at Day 1. Time to vent (or censor) will be calculated as date of event (or censor date) – Day 1 date +1. Event rates at week 12 and week 26 will also be presented in the summary tables. Comparisons between treatment groups will be done using Cox proportional hazard models (Cox 1976) stratified by disease type (C3G vs IC-MPGN) and stratification factors, and adjusted for baseline eGFR values and baseline log-transformed uPCR values. The hazard ratio, 95% CI, and p-value will be presented for the pegcetacoplan group over placebo group.

A data listing for each participant's time to achieve 50% reduction in uPCR with a stable or improved eGFR will be provided using ITT set.

### 6.7.6. Time to Normalization for Selected Parameters for Participants in Whom the Parameter is Abnormal at Baseline

For parameters serum C3, uPCR (measured by triplicate FMU), serum albumin, and blood pressure (both SBP and DBP), time to normalization (in weeks) will be summarized using ITT set with the Kaplan-Meier method, using plots and presentation of median (and 95% CIs) for time-to-event. Participants who did not have the event at or prior to Week 26 in study will be censored at the minimum of the last date of contact or their treatment discontinuation date, or their Week 26 visit or first dose date+182 if Week 26 is missing. Participants who were randomized but did not receive any treatment will be censored at Day 1. Time to vent (or censor) will be calculated as date of event (or censor date) – Day 1 date +1. Event rates at week 12 and week 26, as well as p-values from the log-rank test (stratified by disease type and stratification factors) will also be presented in the summary tables. The analyses will be based on participants whose test result being abnormal at baseline.

A data listing for each participant's time to normalization for these parameters will be provided using ITT set.

### 6.7.7. The Change From Baseline in Glomerular Macrophage Count

The observed values for glomerular macrophage count, determined by CD68 staining, will be summarized by treatment group and visit using ITT set. The categories for glomerular macrophage count are: 0 = None, 1 = Mild (<25% glomeruli), 2 = Moderate (26 to 50 % glomeruli), 3 = Severe (>50 – 75 % glomeruli), and 4 = Extensive (>75%).

All glomerular macrophage count results will be listed for the ITT set.

### 6.7.8. The Change From Baseline in PGIC Score

A shift table of changes in PGIC score from baseline to week 26 will be tabulated by treatment group.

All PGIC score results will be listed for the ITT set.

#### 6.7.9. The Change From Baseline in EQ-5D-5L Score

The observed values for EQ-5D-5L score (based on visual analog scale) will be summarized by treatment group and visit using ITT set. Summaries will present the descriptive statistics for baseline, absolute values, change and percentage change from baseline data by visit.

An ANCOVA model will be used to analyze the exploratory endpoint. The model will include treatment as a fixed effect, adjusted for baseline EQ-5D-5L score, disease type (C3G vs IC-MPGN), and stratification factors. Further details on the presentation of the results are described in Section 6.1. No multiple imputations will be applied. ICE strategies will be simplified where participants with missing data at week 26 (whether due to ICE or not) will be excluded from the modeling. Alternative approaches on the construction of EQ-5D-5L score may be explored as post-hoc analysis if necessary.

All EQ-5D-5L score results will be listed for the ITT set.

#### 6.7.10. The Change From Baseline in WPAI Score

The observed values for WPAI score will be summarized by treatment group and visit using ITT set. Summaries will present the descriptive statistics for baseline, absolute values, change and percentage change from baseline data by visit.

The WPAI score is constructed based on the responses to the 6 questions: Q1=currently employed, Q2=hours missed due to specified problem, Q3=hours missed other reasons, Q4=hours actually worked, Q5=degree problem affected productivity while working, Q6=degree problem affected regular activities. Each response will be multiplied by 100 to express in percentages. And the WPAI score is constructed as Q2/(Q2+Q4)+[(1-(Q2/(Q2+Q4)))x(Q5/10)], which indicates percent overall work impairment due to problem.

An ANCOVA model will be used to analyze the exploratory endpoint. The model will include treatment as a fixed effect, adjusted for baseline WPAI score, disease type (C3G vs IC-MPGN), and stratification factors. Further details on the presentation of the results are described in Section 6.1. No multiple imputations will be applied. ICE strategies will be simplified where participants with missing data at week 26 (whether due to ICE or not) will be excluded from the modeling.

All WPAI score results will be listed for the ITT set.

#### 6.7.11. Correlation Analysis on Reduction in uPCR and Change in eGFR

An exploratory correlation analysis exploring the relationship between the reduction in uPCR and change in eGFR will be considered at week 26, with correlation coefficients provided. The analysis may be repeated based on alternative populations (such as ITT or subgroups based on baseline level) or uPCR reduction metrics (such as change from baseline, percentage change from baseline, or absolute levels) if needed.

# 7. SAFETY ANALYSIS

The safety analysis will be performed using the Safety Set. Safety variables include AEs, clinical laboratory data, vital signs, and ECGs. For each safety variable, the last value collected before the first dose of the investigational product will be used as a baseline for all analyses of that safety variable.

All safety analyses will be conducted according to the treatment the participant actually received.

## 7.1. Adverse Events

Treatment-emergent adverse events (TEAEs) are defined as those AEs that develop or worsen after the first dose of double-blinded investigational product and up to 56 days beyond the last dose of investigational product. Analysis of AEs (as discussed below) will be limited to TEAEs, but data listings will include all AEs regardless of their timing.

All reported AEs will be coded using the current version of MedDRA and summarized by System Organ Class (SOC) and Preferred Term (PT). All summaries by SOC and PT will be ordered by decreasing frequency of participants within each SOC and then similarly by decreasing frequency of participants within each PT based on the pegcetacoplan group. In the case of equal frequency of number of participants in SOCs or PTs, summaries of TEAEs will be sorted alphabetically. Rules for handling missing severity, relationship to study drug, relationship to infusion procedure, outcome, and missing dates of AEs are described in Section 13.

If a PT was reported more than once for the same participant, that participant would only be counted once in the incidence for that PT. For participants experiencing the same PT at multiple severities, the occurrence of the AEs with the greatest severity will be used in the analysis of incidence by severity. For participants experiencing the same PT at multiple relationship levels, the occurrence of the AEs with the strongest relationship to study drug will be used in the analysis of incidence by relationship to study drug.

The AE summaries will be presented by treatment group. In addition, the incidence of death, stratified by transplant history will be summarized.

In post-transplant participants, the number and incidence of rejection episodes at week 26 will be tabulated by treatment group. Additionally, if the number of participants is sufficient, the results for treatment groups will be compared using Fisher's exact test (or a stratified Cochran-Mantel-Haenszel $\chi$-square test as appropriate).

In participants who are posttransplant, the number and incidence of graft loss at week 26 will be tabulated by treatment group. Additionally, if the number of participants is sufficient, the results for treatment groups will be compared using Fisher's exact test (or a stratified Cochran-Mantel-Haenszel $\chi$-square test as appropriate).

An overall summary for TEAEs, including number of participants who experience a TEAE, number of total TEAEs, and number of unique TEAEs, will be provided:

- TEAE
- Treatment-related TEAE
- Infusion-related TEAEs
- Serious TEAE
- Maximum severity of TEAEs
- Infusion site reaction
- TEAE leading to treatment withdrawn
- TEAE leading to dose interruption
- TEAE leading to study discontinuation
- TEAE leading to death
- Rejection episodes (for posttransplant only, based on PTs of “KIDNEY TRANSPLANT REJECTION”, “RENAL AND PANCREAS TRANSPLANT REJECTION”, “MULTIPLE ORGAN TRANSPLANT REJECTION”, “SOLID ORGAN TRANSPLANT REJECTION”)
- Graft loss (for posttransplant only, based on PTs of “TRANSPLANT FAILURE”, “ENGRAFT FAILURE”, “RENAL TRANSPLANT FAILURE”, “REMOVAL OF RENAL TRANSPLANT”, “GRAFT LOSS”)

The following summaries will be provided:

- TEAEs by SOC and PT
- TEAEs by PT in decreasing order of frequency
- Treatment-related TEAEs by SOC and PT
- Infusion-related TEAEs by SOC and PT
- Serious TEAEs by SOC and PT
- TEAE by SOC, PT and maximum severity
- TEAE leading to treatment discontinuation by SOC and PT
- TEAE leading to dose interruption by SOC and PT
- TEAE leading to study discontinuation by SOC and PT
- TEAE leading to death by SOC and PT
- Treatment-emergent adverse events of special interest (AESIs) by SOC and PT
- Serious treatment-emergent AESIs by SOC and PT
- Exposure-adjusted incidence TEAEs by SOC and PT
- Exposure-adjusted incidence serious TEAEs by SOC and PT

Exposure-adjusted incidence rate of an AE is defined as the number of events divided by the total duration of exposure of the participants at risk during treatment, which is computed as following:

Exposure-adjusted incidence rate = n*100/EY,

where n is the number of participants impacted; the exposure year (EY) = (sum of time to onset of the event)/365.25, where time to onset of the event = date of onset of event – Day 1 date +1. For participants without the event, time to onset is censored as follows: time to onset of the event = (last dose date + 56 days) – Day 1 date +1. For participants who experienced the same event on multiple occasions: time to onset of the event = date of onset at the first occasion – Day 1 date +1.

The following listings will be provided:

- All AEs
- Serious adverse events (SAEs)
- AEs leading to study drug discontinuation
- Death
- Treatment-related adverse events
- Infusion-related adverse events

## 7.2. Clinical Laboratory Data

All laboratory results will be standardized by converting values in original units to values in standard units and classified as normal, abnormal low, or abnormal high on normal ranges supplied by the local laboratories and upon employing standardization.

When a certified local laboratory is used instead of a central laboratory due to COVID-19 pandemic, the units and normal ranges might be different. Therefore, all local laboratory values will be standardized to a central laboratory value using the following formula:

$$X_S = L_S + \frac{U_S - L_S}{U_X - L_X} \times (X - L_X)$$

Where,

$X$: local lab value

$X_S$: standardized value of X

$L_S$: Low normal range for central lab

$U_S$: High normal range for central lab

$L_X$: Low normal range for local lab

$U_X$: High normal range for local lab

Specifications will be developed to detail all the steps necessary for developing the standardized laboratory values and to address issues like missing normal ranges or negative standardized laboratory values.

Observed and change from baseline of clinical laboratory data (hematology, serum chemistry, coagulation and continuous urine chemistry parameters) will be summarized at each analysis visit by treatment group and overall. Categorical urine chemistry data will also be tabulated at each analysis visit when applicable.

In addition, liver abnormalities will be summarized at each analysis visit including following variables by treatment group and overall:

- ALT or AST ≥ 3×ULN
- ALT or AST ≥ 3×ULN and (TBL >2×ULN or INR >1.5)
- ALT or AST ≥ 5×ULN
- ALT or AST >8×ULN

Clinical laboratory test values are potentially clinically significant (PCS) if they meet either the low or high PCS criteria listed in the table below:

**Table 2: Criteria for Potentially Clinically Significant Laboratory Tests**

| Parameter | Criteria |
|---|---|
| **Hematology** | |
| WBC (total) (x10^9/L) | < 3.0<br>> 16 |
| Lymphocyte (x10^9/L) | < 0.5<br>< 0.8<br>> 12 |
| Neutrophils (x10^9/L) | < 1.0<br>< 1.5<br>> 12 |
| RBC (x10^12/L) | < 3.3<br>> 6.8 |
| Hemoglobin (g/dL) | < 10 |
| Platelet count (x10^9/L) | < 100<br>> 600 |

**Table 2: Criteria for Potentially Clinically Significant Laboratory Tests**

| Parameter | Criteria |
|---|---|
| **Serum Chemistry** | |
| ALT | > 1.5xULN<br>> 3.0xULN |
| AST | > 1.5xULN<br>> 3.0xULN |
| ALP | > 1.5xULN<br>> 3.0xULN |
| Total Serum Bilirubin | > 1.5xULN |
| GGT | > 3.0xULN |
| ALT or AST > 3xULN and concurrent elevated total bilirubin defined as | > 2.0xULN |

For the following serum chemistry parameters: creatinine; LDH; creatine kinase (CK); uric acid; glucose; sodium; potassium; and chloride, abnormal values will be used as potentially clinically significant.

The number and percentage of participants with post-baseline PCS laboratory values will be tabulated by treatment group and overall. A listing of participant data with at least one post-baseline PCS value will be provided.

All laboratory values will be listed for each participant.

## 7.3. Vital Signs

Observed and change from baseline in vital signs (pulse rate, respiratory rate, systolic and diastolic blood pressure, temperature, and weight) will be summarized by treatment group and overall at each analysis visit.

Additionally, the number and percentage of participants with post-baseline potentially clinically significant (PCS) vital sign values will be tabulated. Vital signs are potentially clinically significant (PCS) if they meet the PCS criteria listed in the table below:

**Table 3: Criteria for Potentially Clinically Significant Vital Signs**

| VS parameter | Criteria |
|---|---|
| HR | ≥100 BPM<br><40 BPM |
| SBP | ≥130 mm Hg<br>≥160 mm Hg<br>≥180 mm Hg<br>>20 mm Hg increase from baseline<br><90 mm Hg<br>≥20 mm Hg decrease from baseline |
| DBP | ≥90 mm Hg<br>≥15 mm Hg increase from baseline<br><40 mm Hg<br>≥15 mm Hg decrease from baseline |
| Temp | ≥38°C |

All vital sign values and change from baseline values will be listed. In the listing, potentially clinically significant vital sign values will be flagged.

## 7.4. Electrocardiogram (ECG)

Observed and change from baseline in ECGs (heart rate, PR interval, QRS duration, QT interval, and QTc interval which is corrected by Fridericia's method) will be summarized by treatment group and overall at each analysis visit.

Additionally, the number and percentage of participants with post-baseline potentially clinically significant (PCS) ECG values will be tabulated. ECGs are potentially clinically significant (PCS) if they meet the PCS criteria listed in the table below:

**Table 4: Criteria for Potentially Clinically Significant ECGs**

| ECG parameter | Criteria |
|---|---|
| HR | <40 bpm<br>>100 bpm |
| PR | >200 msec |
| QRS | >120 msec |
| QTcF | >450 msec<br>>480 msec<br>>500 msec |
| QTcF increase from baseline | >30 msec<br>>60 msec |

All ECG values and change from baseline values will be listed. In the listing, potentially clinically significant ECG values will be flagged.

## 7.5. Other Safety Data

Physical examinations will be described in a data listing.

# 8. PHARMACOKINETIC ANALYSIS

All summaries and analyses of the pharmacokinetic data will be based on the PK set as defined in Section 4.5.

## 8.1. Drug Concentration

Individual pegcetacoplan concentrations, actual sampling times and deviations from nominal sampling times will be presented in a data listing for all participants included in the PK set. Placebo samples will also be included in the data listing if they are analyzed but will not be summarized.

Pegcetacoplan concentrations will be summarized by treatment group at each scheduled time point using descriptive statistics (including mean, SD, coefficient of variation (CV), median, min, max, geometric mean/%CV). The number of participants with a BLQ concentration at each scheduled time point will also be tabulated. The handling of BLQ concentrations in the summary tables is described in Section 8.2. Missing values will be omitted from the calculation of descriptive statistics.

Linear and semilogarithmic individual concentration-time profiles will be generated using actual sampling times. Linear and semilogarithmic mean (±SE) and median concentration-time profiles will be generated using nominal sampling times. The number of participants contributing to each mean or median value at a visit will be presented above the x-axis.

## 8.2. Handling BLQ Values

### 8.2.1. Handling of BLQ Concentrations in Summary Tables

BLQ concentrations prior to first dosing (day 1): Pre-treatment pegcetacoplan concentrations reported as below the limit of quantification (BLQ) will be taken as zero for the computation of descriptive statistics, except geometric mean. Geometric mean cannot be calculated and will be reported as “N/A” or “-”.

BLQ concentrations occurring at any time after first dosing: Pegcetacoplan concentrations reported as below the limit of quantification (BLQ) will be taken as half the lower limit of quantification (LLOQ/2).

### 8.2.2. Handling of BLQ Concentrations in Figures

BLQ concentrations prior to first dosing (day 1): Pre-treatment pegcetacoplan concentrations reported as below the limit of quantification (BLQ) will be taken as zero for linear plots, and equal to half the lower limit of quantification (LLOQ/2) for semilogarithmic plots.

BLQ concentrations occurring at any time after first dosing: Pegcetacoplan concentrations reported as BLQ will be taken as half the lower limit of quantification (LLOQ/2) for both linear and semilogarithmic plots.

# 9. PHARMACODYNAMIC ANALYSIS

## 9.1. Pharmacodynamic Data

All summaries and analyses of the pharmacodynamic data will be based on the PD set as defined in Section 4.6.

Observed values, changes from baseline, and percentage changes from baseline in C3, AH50, CH50, C3b/iC3b, sC5b9, C4, and C3a will be summarized at each protocol-specified time point using descriptive statistics.

Individual observed values and individual changes from baseline will be presented graphically. Actual sampling times will be used for the graphical presentation of individual data. The mean (± SE) of the observed values, mean changes from baseline, and mean percentage changes from baseline will also be presented graphically. Nominal sampling times will be used for the mean plots.

PD parameters will be listed together with changes from baseline and percentage changes from baseline by treatment group.

In addition to the analyses outlined above, all PK and complement biomarker concentration data may be used to develop the population PK and exposure-response models in conjunction with other clinical study data. The methods and procedures will be described in a separate Analysis Plan if needed. The results from population modeling will be reported separately.

# 10. IMMUNOGENICITY

Immunogenicity data will be listed and summarized using safety set separately for anti-pegcetacoplan peptide antibody and anti-PEG antibody results. A sample and participant level summary table will be presented as described below.

**Sample Level Summary**

The number of evaluable (ADA-positive, ADA-negative, and ADA-inconclusive) and unevaluable samples will be summarized by treatment group and overall. The number and percentage of each ADA sample classification will be summarized by treatment group and overall in the evaluable samples. In addition, the number and percentage of baseline and post-dose ADA positive samples along with the titer range for each will also be summarized by treatment group and overall in samples that are ADA positive.

ADA samples will be classified as follows:

- ADA-Positive Sample – when the sample is positive in the confirmatory assay
- ADA-Negative Sample – when the sample is negative in the screening assay or the confirmatory assay, and drug is at a level that does not interfere with the ADA method.
- ADA-Inconclusive Sample – when the sample is negative in the screening assay or the confirmatory assay, and drug is at a level that interferes with the ADA method, then the sample is considered inconclusive. Note that the drug is considered at a level that interferes with the ADA method if the corresponding visit's PK concentration is greater than or equal to: (a)1000 ug/mL for anti-pegcetacoplan peptide antibody; or (b) 5000 ug/mL for anti-PEG antibody.
- Unevaluable Sample – when a sample could not be tested for ADA status due to inadequate sample volume, mishandling, or errors in sample collection, processing, storage, etc.

**Participant Level Summary**

The number and percentage of participants with pre-existing ADAs will be summarized by treatment group and overall in the participants with a baseline sample result. In addition, the number and percentage of participants with an ADA positive response, ADA negative response, and ADA inconclusive response, as well as the type and kinetics of the positive ADA response, will be summarized by treatment group and overall in the evaluable participants. The participant summary table will include a summary by treatment group and overall for the ADA population parameters. For participants with an ADA positive response, association with changes in PK, efficacy, and safety may be explored.

Pre-existing ADA will be defined as follows: any participant with an ADA positive baseline sample from the total participants with a baseline sample result.

Evaluable participants will be defined as follows: a participant with at least one sample taken with a reportable result after first dosing during the treatment or follow-up period.

Evaluable participants will be classified as follows for ADA response:

- ADA-Positive Participant – An evaluable participant with at least one pre-dose sample and one treatment-emergent or treatment-boosted ADA-positive sample at any time after dosing
- ADA-Negative Participant – An evaluable participant without a treatment-emergent or treatment-boosted ADA-positive sample during the treatment or follow-up period
- ADA-Inconclusive Participant – An evaluable participant who cannot be classified as either ADA-positive or ADA-negative (eg, assay drug tolerance issues, post-dose positive without a baseline sample, positive baseline and positive post-dose sample without a titer value, etc.)

ADA positive responses will be classified as follows:

- Treatment-Boosted ADA Response – An evaluable participant with a baseline ADA positive sample and a post-dose ADA positive sample that is ≥ 4x the baseline titer (eg, baseline titer of 10 vs. post-dose titer of 40)
- Treatment-Emergent ADA Response – An evaluable participant with a baseline ADA negative sample and an ADA positive sample after treatment, ADA developed *de novo*

The kinetics of ADA positive responses will be classified as follows:

- Transient ADA Response
  - Treatment-emergent positive participants are classified as having a transient response if they have only a single ADA positive sample (that was not the last assessment), or more than 1 ADA positive sample where the first and last ADA positive samples are separated by a period of less than 112 days (16 weeks), irrespective of any negative and positive samples in between.
  - Treatment-boosted ADA positive participants are classified as having a transient response if they have only a single ADA boosted sample (that was not the last assessment), or more than 1 positive boosted sample where the first and last ADA positive samples are separated by a period of less than 112 days (16 weeks), irrespective of any negative and positive samples in between.
- Persistent ADA Response
  - Treatment-emergent positive participants are classified as having a persistent response if they have more than 1 positive ADA sample ≥ 112 days (16 weeks) apart, or a positive ADA sample at the last timepoint with no further results available
  - Treatment-boosted ADA positive participants were classified as having a persistent response if they have more than 1 positive boosted sample ≥ 112 days (16 weeks) apart, or a positive boosted sample at the last timepoint with no further results available
- Unclassified Response
  - Any ADA positive participant that cannot be defined as having a transient or persistent ADA response

ADA population parameters are derived as follows:

- ADA Prevalence – The proportion of all ADA positive participants, including pre-existing ADA, computed as a percentage of the total number of evaluable and unevaluable participants
- ADA Incidence – The sum of all ADA positive treatment-emergent and treatment-boosted participants computed as a percentage of the total number of evaluable participants

  Note: This population parameter is the same as the percentage of participants with an ADA positive response.

# 11. INTERIM ANALYSIS

No formal interim analysis is planned for this study.

# 12. DATA MONITORING COMMITTEE

A Data Monitoring Committee (DMC) will review cumulative safety/tolerability data (eg, physical examinations, ECGs, vital signs, clinical laboratory tests, and AEs) and efficacy data. The DMC will have the responsibility to conduct a thorough safety assessment at regular pre-defined intervals during the treatment period of the study.

DMC meetings will be held according to the schedule in the DMC charter. An ad hoc DMC data review may be recommended by the DMC or requested by the sponsor at any time during the study.

The remit, roles, and responsibilities of the DMC will be specified in a separate DMC charter. There will be a separate DMC SAP covering the presentation and analyses for the DMC.

# 13. DATA HANDLING CONVENTIONS

## 13.1. General Data Reporting Conventions

All statistical analyses will be performed using SAS® (Version 9.4 or higher, SAS Institute Inc., Cary, NC, USA).

Categorical variables will be tabulated as number of participants and percentage of total number of participants in the given analysis set as noted for each category. Descriptive statistics will be used to summarize continuous variables including number of participants, mean, standard deviation (SD), median, Q1, Q3, minimum and maximum. Mean, Q1, Q3, and median will be reported to 1 more decimal place than the raw data, while the SD will be reported to 2 more decimal places than the raw data. Minimum and maximum will be reported the same as the original data.

Participant specific listings will be provided by treatment group, participant ID, study period and visit, if applicable.

## 13.2. Definition of Study Days

Unless otherwise noted, study days of an evaluation are defined as number of days relative to the first dose date of study drug, which is designated as day 1, and the preceding day is day −1, the day before that is day −2, etc.

- For assessments on/after study day 1, study days are calculated as:
  - (date of assessment– date of study day 1 + 1)
- For assessments before study day 1, study days are calculated as:
  - (date of assessment – date of study day 1)

## 13.3. Definition of Baseline, RCP, and OLP

For all evaluations unless otherwise noted, baseline is defined as the most recent non-missing measurement prior to the first administration of study drug. Baseline can be the same date as first dose, given the measurement is expected prior to first dose when only date information is available.

In by-visit summary tables, the baseline will be summarized using all available data, but also for each visit using only the baseline data from participants with available data at the visit; hence the mean change from baseline will equal the mean visit value – mean baseline value.

Throughout this document ‘change from baseline’ refers to the actual change from baseline (ie, visit value – baseline value).

The date when a participant receives first administration of OLP drug will be defined as the start date of the participant’s OLP, and that date minus one day will be the end date of the participant’s RCP.

## 13.4. Definition of Analysis Visit Windows

Unless otherwise specified, the actual scheduled nominal post-baseline visit will be used for all summaries across time. Post-baseline unscheduled visits, follow-up visits, and early termination visits will be mapped to a scheduled visit and will be used in the analysis only if the nominal scheduled visit result is missing. Table 5 presents the analysis visit window mapping for unscheduled and early term visits for assessments done mostly every 4 weeks (central lab, vital sign, PK). For other assessments that do not follow Table 5 schedule (such as 24-hour urine, triplicate FMU uPCR, ECG, PD, ADA, etc.), the analysis visit window mapping will be adjusted accordingly. In the case that multiple unscheduled or early termination visits are in the same analysis window, the one closest to the target date will be used. In the event that the windowed visit is mapped to an illogical sequence of visits when considering nearby scheduled visits (ie, windowed visit is higher than the subsequent visit or lower than the preceding visit), the windowed visit will be set to the logical scheduled visit.

**Table 5: Analysis Visit Windows for Unscheduled and Early Termination Visits for Assessments Done Mostly Every 4 Weeks**

| Study period | Analysis visit | Target day | Analysis window | Interval |
|---|---|---|---|---|
| Screening | Week -10 to -4 | -49 | -77 - < -21 | 56 |
| | Week -2 | -14 | -21 – ≤ -1 | 21 |
| Randomized controlled period | Day 1 | 1 | 1 | 1 |
| | Week 4 | 28 | 2 – < 42 | 40 |
| | Week 8 | 56 | 42 – < 70 | 28 |
| | Week 12 | 84 | 70 – < 98 | 28 |
| | Week 16 | 112 | 98 – < 126 | 28 |
| | Week 20 | 140 | 126 – < 154 | 28 |
| | Week 24 | 168 | 154 – < 175 | 21 |
| | Week 26 | 182 | 175 – < OLP start date | NA |
| Open-label period | Week 28 | 196 | OLP start date – <210 | NA |
| | Week 32 | 224 | 210 – < 238 | 28 |
| | Week 36 | 252 | 238 – < 273 | 35 |
| | Week 42 | 294 | 273 – < 315 | 42 |
| | Week 48 | 336 | 315 – < 350 | 35 |
| | Week 52 (for roll-over participants) | 364 | 350 – < 434 | 84 |
| | Week 52 (for non-roll-over participants) | 364 | 350 – < 371 | 21 |

**Table 5: Analysis Visit Windows for Unscheduled and Early Termination Visits for Assessments Done Mostly Every 4 Weeks**

| Study period | Analysis visit | Target day | Analysis window | Interval |
|---|---|---|---|---|
| Follow-up period (for participants not entering long term extension) | Week 54 | 378 | 371 – < 385 | 14 |
| | Week 56 | 392 | 385 – < 406 | 21 |
| | Week 60 | 420 | 406 – < 434 | 28 |

## 13.5. Repeated or Unscheduled Assessments of Safety Parameters

For safety parameters, if a participant has repeated assessments before the start of investigational product, then the results from the final assessment made prior to the start of investigational product will be used as baseline. If end of study assessments are repeated or unscheduled, the last post-baseline assessment will be used as the end of study assessment for generating descriptive statistics. However, all post-baseline assessments will be used for Potentially Clinically Significant (PCS) value determination and all assessments will be presented in the data listings.

## 13.6. Handling of Missing, Unused, and Spurious Data

Missing data is addressed in the relevant sections elsewhere. Spurious data will be discussed with data management and others as appropriate to address queries to sites or vendors.
In general, all data will be used as reported (and after query resolutions); any decisions to remove data would be made and documented prior to unblinding.

### 13.6.1. Missing Date of Investigational Product

When the date of the last dose of investigational product is missing for a participant in the Safety Set, all efforts should be made to obtain the date from the investigator. If it is still missing after all efforts, then the last visit date will be used in the calculation of treatment duration.

### 13.6.2. Missing Date Information for Prior or Concomitant Medications (Therapies/Procedures)

For prior or concomitant medications (and/or therapies/procedures), including rescue medications, incomplete (ie, partially missing) start date and/or stop date will be imputed. When the start date and the stop date are both incomplete for a participant, the start date will be imputed first.

#### 13.6.2.1. Incomplete Start Date

The following rules will be applied to impute the missing numerical fields. If the stop date is complete and the imputed start date is after the stop date, then the start date will be imputed using the stop date.

##### 13.6.2.1.1. Missing Day, Month, and Year

In this case, no start date will be imputed. However, the medication will be assumed to be a prior medication. If the stop date is missing or if the stop date is on or after the date of the first dose of investigational product, the medication will also be considered a concomitant medication.

##### 13.6.2.1.2. Missing Day and Month

- If the year of the incomplete start date is the same as the year of the date of the first dose of investigational product, then the day and month of the date of the first dose of investigational product will be assigned to the missing fields.
- If the year of the incomplete start date is before the year of the date of the first dose of investigational product, then December 31 will be assigned to the missing fields.
- If the year of the incomplete start date is after the year of the date of the first dose of investigational product, then 01 January will be assigned to the missing fields.

##### 13.6.2.1.3. Missing Month Only

- The day will be treated as missing and both month and day will be replaced according to the above procedure.

##### 13.6.2.1.4. Missing Day Only

- If the month and year of the incomplete start date are the same as the month and year of the date of the first dose of investigational product, then the day of the date of the first dose of investigational product will be assigned to the missing day
- If either the year is before the year of the date of the first dose of investigational product or if both years are the same but the month is before the month of the date of the first dose of investigational product, then the last day of the month will be assigned to the missing day
- If either the year is after the year of the date of the first dose of investigational product or if both years are the same but the month is after the month of the date of the first dose of investigational product, then the first day of the month will be assigned to the missing day.

#### 13.6.2.2. Incomplete Stop Date

The following rules will be applied to impute the missing numerical fields. If the date of the last dose of investigational product is missing, then replace it with the last visit date. If the imputed stop date is before the start date (imputed or non-imputed start date), then the imputed stop date will be equal to the start date.

##### 13.6.2.2.1. Missing Day and Month

- If the year of the incomplete stop date is the same as the year as of the date of the last dose of investigational product, then the day and month of the date of the last dose of investigational product will be assigned to the missing fields

- If the year of the incomplete stop date is before the year of the date of the last dose of investigational product, then 31 December will be assigned to the missing fields
- If the year of the incomplete stop date is after the year of the date of the last dose of investigational product, then 01 January will be assigned to the missing fields.

##### 13.6.2.2.2. Missing Month Only

- The day will be treated as missing and both month and day will be replaced according to the above procedure.

##### 13.6.2.2.3. Missing Day Only

- If the month and year of the incomplete stop date are the same as the month and year of the date of the last dose of investigational product, then the day of the date of the last dose of investigational product will be assigned to the missing day
- If either the year is before the year of the date of the last dose of investigational product or if both years are the same but the month is before the month of the date of the last dose of investigational product, then the last day of the month will be assigned to the missing day
- If either the year is after the year of the last dose of investigational product or if both years are the same but the month is after the month of the date of the last dose of investigational product, then the first day of the month will be assigned to the missing day.

### 13.6.3. Missing Date Information for Adverse Events

For AEs with partial start dates, non-missing date parts will be used to determine if the AE is treatment-emergent or not. If a determination cannot be made using the non-missing date parts as to when the AE occurred relative to study drug administration, eg, AE start year and month are the same as the year and month of the first dose of investigational product, then the AE will be classified as treatment-emergent.

To facilitate categorization of AEs as treatment emergent, imputation of dates can be used. For AEs, the default is to only impute incomplete (ie, partially missing) start dates. Incomplete stop dates may also be imputed when calculation of the duration of an AE is required per the protocol. If imputation of an incomplete stop date is required, and both the start date and the stop date are incomplete for a participant, impute the start date first.

- Rules to impute incomplete start date are the same as stated in Section 13.6.2.1.
- Rules to impute incomplete stop date are the same as stated in Section 13.6.2.2.

### 13.6.4. Missing Severity Assessment for Adverse Events

- If severity is missing for an AE starting prior to the date of study day 1, then a severity of “Mild” will be assigned.
- If the severity is missing for an AE starting on or after the date of study day 1, then a severity of “Severe” will be assigned.

The imputed values for severity assessment will be used for incidence summaries, while the actual values will be presented in data listings.

### 13.6.5. Missing Relationship to Investigational Product for Adverse Events

If the relationship to investigational product is missing for an AE starting on or after the date of study day 1, a causality of "related" will be assigned. The imputed values for relationship to double-blind investigational product will be used for incidence summaries, while the actual values will be presented in data listings.

### 13.6.6. Character Values of Clinical Laboratory Variables

If the reported value of a clinical laboratory variable cannot be used in a statistical analysis due to, for example, that a character string is reported for a numerical variable. The appropriately determined coded value will be used in the statistical analysis. If the laboratory results are collected as < or > a numeric value, 0.0000000001 will be subtracted or added, respectively to the value. However, the actual values as reported in the database will be presented in data listings.

# 14. ANALYSIS SOFTWARE

Statistical analyses will be performed using Version 9.4 (or newer) of SAS on a suitably qualified environment.

# 15. CHANGES TO ANALYSIS SPECIFIED IN PROTOCOL

Not applicable.

# 16. REFERENCES

Cox DR. Regression models and life-tables. *J R Stat Soc B*, 1972;34(2):187-220.

Diggle PJ, Liang K-Y, Zeger SL. *Analysis of Longitudinal Data*. Oxford: Clarendon Press, 1994.

Kaplan EL, Meier P. Nonparametric estimation from incomplete observations. *J Am Stat Assoc*. 1958;53(282):457-81.

Li KH. Imputation using Markov chains. *J Stat Comput Simul.*1988;30:57-79.

Liu C. Bartlett's decomposition of the posterior distribution of the covariance for normal monotone ignorable missing data. *J Multivar Anal.*1993; 46:198-206.

O'Kelly M, Ratitch B (editors). Analyses under missing-not-at-random assumptions. *Clinical Trials with Missing Data: A Guide for Practitioners*. Wiley, 2014.

Peipert JD, Nair D, Klicko K, Schatell DR, Hays RD. Kidney Disease Quality of Life 36-Item Short Form Survey (KDQOL-36) normative values for the United States dialysis population and new single summary score. *J Am Soc Nephrol*. 2019;30(4):654-663. doi:10.1681/ASN.2018100994

# 17. APPENDICES

## 17.1. Schedule of Assessments

| **Study period** | **Screening period[a]** | | **Randomized controlled period** | | | | | | | | **Open-label period** | | | | | | **Follow-up period** | | |
|---|---|---|---|---|---|---|---|---|---|---|---|---|---|---|---|---|---|---|---|
| **Study week** | **–10 to –4** | **–2** | **1** | **4** | **8** | **12** | **16** | **20** | **24** | **26** | **28** | **32** | **36** | **42** | **48** | **52** | **54** | **56** | **60 Exit** |
| **Study day** | **–70** | **–14** | **1** | **28** | **56** | **84** | **112** | **140** | **168** | **182** | **196** | **224** | **252** | **294** | **336** | **364** | **378** | **392** | **420** |
| **Study visit** | **1** | **2** | **3** | **4** | **5** | **6** | **7** | **8** | **9** | **10** | **11** | **12** | **13** | **14** | **15** | **16** | **17** | **18** | **19** |
| **Visit window (± days)** | **N/A[b]** | | **0** | **3** | **7** | **7** | **7** | **7** | **3** | **3** | **3** | **7** | **7** | **7** | **7** | **7** | **3** | **3** | **7** |
| **Assessments** | | | | | | | | | | | | | | | | | | | |
| Informed consent | X | | | | | | | | | | | | | | | | | | |
| Demographics | X | | | | | | | | | | | | | | | | | | |
| Medical history | X | | | | | | | | | | | | | | | | | | |
| Post-transplant immunosuppression plan documentation | | X | | | | | | | | | | | | | | | | | |
| Inclusion/exclusion | X | X | X | | | | | | | | | | | | | | | | |
| Vaccination[d] | | X[b] | | | | X | | | | | | | | | | | | | |
| Adverse events | X | X | X | X | X | X | X | X | X | X | X | X | X | X | X | X | X | X | X |
| Concomitant medications | X | X | X | X | X | X | X | X | X | X | X | X | X | X | X | X | X | X | X |
| Physical examination (full)[e] | X | | X | | | | | | | X[e] | | | | | | X | | | |
| Physical examination (brief)[e] | | X | | X | X | X | X | X | X | | X[e] | X | X | X | X | | X | X | X |
| 12-lead ECG | X | | X | | X | | X | | X | X | X | | X | | X | X | X | | X |
| Chest radiography | | X[b] | | | | | | | | X | | | | | | X | | | |
| Renal biopsy[f] | X[b] | | | | | | | | | X[g] | | | | | | Xh | | | |
| Randomization | | | X | | | | | | | | | | | | | | | | |
| Study drug administration[i] | | | X | X | X | X | X | X | X | X | X | X | X | X | X | X | | | |
| Infusion site/pump safety assessment[j] | | | X | X | X | X | X | X | X | X | X | X | X | X | X | X | X | | |
| Vital sign measurements[k] | X | X | X | X | X | X | X | X | X | X | X | X | X | X | X | X | X | X | X |
| HRQoLl | | X | | | | | | | | X | | | | | | X | | | |

| Study period | Screening period[a] | | Randomized controlled period | | | | | | | | Open-label period | | | | | | Follow-up period | | |
|---|---|---|---|---|---|---|---|---|---|---|---|---|---|---|---|---|---|---|---|
| **Study week** | **–10 to –4** | **–2** | **1** | **4** | **8** | **12** | **16** | **20** | **24** | **26** | **28** | **32** | **36** | **42** | **48** | **52** | **54** | **56** | **60 Exit** |
| **Study day** | **–70** | **–14** | **1** | **28** | **56** | **84** | **112** | **140** | **168** | **182** | **196** | **224** | **252** | **294** | **336** | **364** | **378** | **392** | **420** |
| **Study visit** | **1** | **2** | **3** | **4** | **5** | **6** | **7** | **8** | **9** | **10** | **11** | **12** | **13** | **14** | **15** | **16** | **17** | **18** | **19** |
| **Visit window (± days)** | **N/A[b]** | | **0** | **3** | **7** | **7** | **7** | **7** | **3** | **3** | **3** | **7** | **7** | **7** | **7** | **7** | **3** | **3** | **7** |
| **<u>Urine</u>** | | | | | | | | | | | | | | | | | | | |
| 24-hour urine collection[m] | X | | | | | X | | | X | | | | X | | X | | | | X |
| Triplicate FMU uPCR[n] | X | X | X[o] | X | X | X | X | X | X | X | X | X | X | X | X | X | X | X | X |
| In-clinic (random) uPCR | X | X | X | X | X | X | X | X | X | X | X | X | X | X | X | X | X | X | X |
| Urinalysis (dipstick & microscopic)[p] | X | | | | | | | | | | | | | | | | | | |
| Urine pregnancy test | | X | X | X | X | X | X | X | X | X | X | X | X | X | X | X | X | X | X |
| **<u>Blood</u>** | | | | | | | | | | | | | | | | | | | |
| Hematology[p] & chemistry[p] | X | X | X | X | X | X | X | X | X | X | X | X | X | X | X | X | X | X | X |
| PK sample collection | | | X | X | X | X | X | X | X | X | X | X | X | X | X | X | X | X | X |
| ADA assaysq | | | X | X | | | X | | X | | | | X | | | X | X | | X |
| Serum complement profilep | X | X | X | X | X | X | | X | X | X | X | X | X | | X | X | X | X | X |
| Plasma complement profile[p] | X | | X | | X | | X | | X | X | X | | X | | X | X | X | | X |
| eGFRr | X | X | X | X | X | X | X | X | X | X | X | X | X | X | X | X | X | X | X |
| Pregnancy (β-HCG) | X | | | | | | | | | | | | | | | | | | |
| Screening assayss | X | | | | | | | | | | | | | | | | | | |
| **<u>Optional assessments</u>** | | | | | | | | | | | | | | | | | | | |
| Ophthalmologic evaluations[t] | | Xt | | | | | | | | | | | | Xt | | | | | |
| Measured GFR[u] | | | Xu | | | | | | | X[u] | | | | Xu | | | | | |

Abbreviations: Ab = antibodies; ADA = antidrug antibodies; AE = adverse events; AH50 = 50% alternative hemolytic complement pathway activity; ANA = antinuclear antibodies; ANCA = antineutrophil cytoplasmic antibodies; β HCG = beta human chorionic gonadotropin; CH50 = 50% classical hemolytic complement pathway activity; ECG = electrocardiogram; eGFR = estimated glomerular filtration rate; EQ-5D-5L = 5-Level EuroQol-5 Dimension; FACIT-Fatigue = Functional Assessment of Chronic Illness Therapy-Fatigue Scale; FMU = first-morning spot urine; FSH = follicle-stimulating hormone; GFR = glomerular filtration rate; HIV = human immunodeficiency virus; HRQoL = health-related quality of life; KDQOL = Kidney Disease Quality of Life; N/A = not applicable; PGIC = Patient Global Impression of Change; PK = pharmacokinetic; SC = subcutaneous; SD OCT = spectral domain optical coherence tomography; SPEP = serum protein electrophoresis; uPCR = urine protein-to-creatinine ratio; WPAI = Work Productivity and Activity Impairment.

<u>NOTE</u>: When multiple assessments/procedures occur at the same visit, order should be: vital signs, ECGs, blood collection/sampling, study drug dosing.

a. All visit 2 assessments need not occur in a single visit and visit 2 can be split into multiple visits (eg, visit 2a, visit 2b).

b. Vaccinations, baseline renal biopsy, and screening chest radiography can occur any time during screening after confirmation of eligibility based on visit 1 data.

c. For transplant participants only. Must be documented prior to assignment to study treatment.

d. Vaccine series should be initiated at least 14 days prior to randomization; additional vaccines may be required at visit 6. Please see Protocol Section 8.2 for more details on vaccination requirements. Vaccination serum samples will be collected on the day of vaccination before receiving vaccinations on that visit. These samples will be analyzed to evaluate response to vaccinations in the event that the participant has a positive infection for Streptococcus pneumoniae, Neisseria meningitidis, or Haemophilus influenzae.

e. A full physical examination is required at visits 1, 3 (week 1), 10 (week 26), and 16 (week 52). A full physical examination should also be conducted on the first day of open-label dosing, if that is not at the week 26 visit. Brief physical examinations, including weight (kg) and assessment of edema, will be conducted at all other visits noted. A symptom-driven physical examination may be performed at any time, at the investigator's discretion. Body height (cm) will be measured only during screening for adults but will be measured at screening and every 12 weeks throughout the study for adolescents; weight (kg) will be measured throughout study, during brief and full physical examinations for all participants. Both body weight and height will be assessed without shoes on; height will be measured using a calibrated stadiometer. Edema should be assessed at every visit.

f. Renal biopsies will not be required for participants younger than 18 years provided that they have adequate previous renal biopsies to establish the diagnosis as per the central pathology laboratory.

g. The week 26 renal biopsy need not occur on the same day as all other assessments for that visit. However, the week 26 triplicate FMU uPCR collections must occur before the renal biopsy. In addition, the week 26 biopsy must occur before the first visit of the open-label period at week 28. Participants younger than 18 years are not required to provide renal biopsies and may advance to the open-label period upon completion of all assessments for weeks 24 through 26 other than the renal biopsy.

h. The week 52 renal biopsy is optional for all participants. If performed it should be after collection of the week 48 24-hour urine and FMU samples, and not more than 8 weeks after the week 52 visit. In the event that a participant has a renal biopsy as part of their clinical management within this window, it may serve as the week 52 biopsy provided that it includes the required components for this study.

i. Study drug will be self-administered by the participant or administered by their caregiver, after receiving appropriate training and sign-off by a research nurse (or other qualified personnel) in their first treatment week, as described in Protocol Section 9.5. Once qualified, the participant or caregiver should administer study drug at site visits (as done at home) on those days when a clinic visit occurs on a dosing day. If a home nurse is administering study drug on nonvisit days, the site staff may administer study drug on days of site visits.

j. Between site visits, participants will be instructed to report any infusion site reactions to the study staff. Pump use safety will be reviewed by licensed health care professionals (eg, investigator or nurse) for each study drug administration at clinic visits and during at-home qualification.

k. Vital signs should be measured a maximum of 2 hours before study drug infusion. On day 1 and on the first day of open-label dosing (week 26 or week 28, depending on whether a week 26 renal biopsy is required and when it is performed), vital signs should also be measured approximately 30 minutes to 1 hour after the first infusion of study drug dosing, timed from the completion of the study drug administration. Blood pressure and heart rate should be evaluated after the participant has been resting in a seated position for at least 5 minutes, except when they are supine or semireclined because of study procedures and/or AEs, or if deemed necessary by the investigator.

l. FACIT-Fatigue, EQ-5D-5L, KDQOL, PGIC, and WPAI.

m. The screening 24-hour urine collection may be done any time between screening visit 1 and screening visit 2. After week 1, collections should be within ±1 week of the visit (except for the week 24 collection, which cannot be earlier than week 24). Courier arrangements can be made to pick up the collection container from the participant, or the participant may return the container directly to the site.

n. Triplicate FMU samples will be collected by the participant at home on 3 consecutive days throughout the duration of the study. These should be the first urinary output of the day. An additional triplicate FMU uPCR sample will be collected at week 25. Samples should be collected within ±1 week of the clinic visit (except for weeks 24 through 26, when sample collection should be within ±3 days of the clinic visit). Courier arrangements can be made to pick up the collection containers from the participants, or the participant may return the containers directly to the site. At every visit, enough uPCR collection containers should be dispensed to the participant to enable all at-home uPCR collections until the next clinic visit.

o. The day 1 triplicate FMU uPCR samples should be collected before the first dose of study drug (eg, day –2, day –1, and before dosing on day 1).

p. Serum complement profile includes AH50, CH50, and C3NeF; C3NeF will only be assayed in samples collected at the baseline (screening) visits. Plasma complement profile includes C3a, C3b/iC3b, C5a, and sC5b 9. See laboratory assessments (Protocol Table 4) for more details.

q. The day 1 samples should be collected before dosing with study drug. Participants who discontinue dosing will have ADA samples collected at 2 and 8 weeks after the last treatment. Participants who have a treatment-emergent or treatment-boosted ADA response at any time will have ADA samples collected approximately every 6 months until the antibody levels revert to baseline.

r. eGFR will be calculated using the Chronic Kidney Disease–Epidemiology Collaboration creatinine equation for adults or the Bedside Schwartz equation for adolescents. For each participant, eGFR will be calculated using the same formula for the duration of the study; the choice of formula will be determined by the participant's age at study entry.

s. Serum FSH (to be measured in female participants only), hepatitis B panel, hepatitis C panel, HIV antibodies, SPEP (adult participants only), ANA, and ANCA (see Protocol Table 4 and laboratory manual for more details).

t. Ophthalmologic evaluations are optional and will be performed at selected sites. If evaluations are performed, each participant should have a baseline ophthalmologic evaluation, including a basic ophthalmologic examination, SD-OCT, and color fundus photography, at an approved ophthalmologic clinical site at any time during the screening period. For participants who have drusen before pegcetacoplan administration, a follow-up ophthalmologic evaluation, including an SD-OCT and color fundus photography, should occur at a convenient time between weeks 44 and 52.

u. Measured GFR is an optional assessment that, if performed, should occur at 3 time points: once on day 1 or within 10 weeks before day 1, again at week 26, and a third time between week 44 and week 52, inclusive. Measured GFR should only be done at sites where it is routinely performed, as per the site's standard protocol.

## 17.2. Sample SAS code

```
* The SAS codes in this section are shown as examples
* Some modifications (such as variables used in the model statement)
* may require further changes to reflect the methods specified in the
* SAP and analysis data structure
```

### 17.2.1. Mixed Effect Model for Repeated Measure

```
*----------------------------------------------------
* Read Analysis data
*----------------------------------------------------
* In this example, repeated measures are available at
* avisitn = 0 (baseline), 4 (Week 4), 8 (Week 8), 12 (Week 12), 16 (Week 16),
*   20 (Week 20), 24 (Week 24), 25 (Week 25), and 26 (Week 26)
*----------------------------------------------------;

data upcr;
 set adam.adeff;
 if paramcd = 'LNUPRCRF' and basetype = 'NORMAL' and
 avisitn in (0, 4, 8, 12, 16, 20, 24, 25, 26) and
 ANL01FL = 'Y';

 * avisitc will be used in PROC TRANSPOSE;
 if avisitn = 0 then avisitc = '00'; * Baseline;
 if avisitn = 4 then avisitc = '04'; * Week 4;
 if avisitn = 8 then avisitc = '08'; * Week 8;
 if avisitn = 12 then avisitc = '12'; * Week 12;
 if avisitn = 16 then avisitc = '16'; * Week 16;
 if avisitn = 20 then avisitc = '20'; * Week 20;
 if avisitn = 24 then avisitc = '24'; * Week 24;
 if avisitn = 25 then avisitc = '25'; * Week 25;
 if avisitn = 26 then avisitc = '26'; * Week 26;
run;

********************************;
*** ANALYSIS using MMRM assuming MAR (e.g., Section 6.4.2(1) )***;
********************************;

proc sql;
      select avg(base) into: abs
      from upcr_t; * upcr_t is the upcr dataset but in long format;
run;

%put abs=&abs;   * this will be used for the “estimate” statement, similarly
to obtain the empirical weight for each stratification factor and disease
type and baseline immune use, namely stra1_wt, stra1_wt_rev, stra2_wt,
stra2_wt_rev, distype_wt, distype_wt_rev, immunobl_wt, immunobl_wt_rev to be
used in next procedure;


proc mixed data=upcr method=reml covtest empirical;
  where avisitn NE 0; * exclude rows for baseline;
  * stra1 (stratification factor 1)
  * 0 = post-transplant recurrence
  * 1 = non-transplant
```

```
  * stra2 (stratification factor 2)
  * 0 = Without Baseline Renal Biopsy
  * 1 = With Baseline Renal Biopsy
  * distype (disease type)
  * 0 = IC-MPGN
  * 1 = C3G
  * immunobl (baseline immunosuppressants use)
  * 0 = No
  * 1 = Yes
  class trtpn avisitn usubjid stra1 stra2 distype;
  model chg = trtpn stra1 stra2 distype immunobl avisitn trtpn*avisitn
base/cl;
  repeated avisitn / subject=usubjid type=un r;
  lsmeans trtpn*avisitn/pdiff cl e om alpha=.05;
  ods output diffs= diff1 LSMeans= LSMean1;

estimate 'PEG Week 24-25-26 Avg LSMean' intercept 1 stra1 &stra1_wt_rev
&stra1_wt stra2 &stra2_wt_rev &stra2_wt distype &distype_wt_rev
&distype_wt immunobl &immunobl_wt_rev &immunobl_wt trtpn 1 0
BASE &abs
avisitn 0 0 0 0 0 0.333 0.333 0.334
trtpn*avisitn 0 0 0 0 0 0.333 0.333 0.334
               0 0 0 0 0 0 0 0 /cl ;
estimate 'PEG vs PBO Difference Week 24-25-26 Avg' trtpn 1 -1
trtpn*avisitn 0 0 0 0 0 0.333 0.333 0.334
               0 0 0 0 0 -0.333 -0.333 -0.334 /cl ;
ods output estimates = est;
run;


data lsmeans1;
 set lsmeans;
 geo_estimate = exp(estimate);     * = LS Geometric mean;
 geo_lower = exp(lower);           * = lower bound of the 95%CI for the
LS Geometric mean;
 geo_upper = exp(upper);           * = upper bound of the 95%CI for the
LS Geometric mean;
run;

data diffs1;
 set diffs;
 geo_estimate = exp(estimate);     * = LS Geometric mean ratio;
 geo_lower = exp(lower);           * = lower bound of the 95%CI for the
LS Geometric mean ratio;
 geo_upper = exp(upper);           * = upper bound of the 95%CI for the
LS Geometric mean ratio;
run;

data est1;
 set est;
 geo_estimate = exp(estimate);     * for composite results;
 geo_lower = exp(lower);
 geo_upper = exp(upper);
run;
```

### 17.2.2. Multiple Imputation

```
********************************;
*** MAIN ANALYSIS (e.g., Section 6.4.1 )***;
********************************;

********************************;
*** RESHAPE DATA FOR PROC MI ***;
********************************;

proc sort data= upcr;
 by usubjid stra1 stra2 distype immunobl trtpn base avisitc;
run;

proc transpose data= upcr out= upcr_t(drop= _NAME_ _LABEL_) prefix=V;
 by usubjid stra1 stra2 distype immunobl trtpn base;
 id avisitc;
 var aval;
run;

* Examine the missing patterns of the data;
proc mi data=upcr_t nimpute=0;
 var stra1 stra2 distype immunobl trtpn v00 - v26;
 ods output missPattern=pattern;
run;

*****************************************;
*** MCMC (impute nonmonotone missing) ***;
*****************************************;

* Below statements invoke MCMC procedure and specify IMPUTE=MONOTONE
 to turn the arbitrary missing patterns to monotone missing patterns
 under missing at random (MAR) assumption;

proc mi data=upcr_t out=upcr_t_mono seed= 230815 nimpute=100;
 mcmc chain=multiple impute= monotone displayinit
     initial=em(itprint);
 var stra1 stra2 distype immunobl trtpn v00 - v26;
run;

* Examine the missing patterns of the data;
proc mi data=upcr_t_mono nimpute=0;
 var stra1 stra2 distype immunobl trtpn v00 - v26;
 ods output missPattern=pattern;
run;

*******************************************************************;
*** Control-based pattern imputation(impute monotone missing) ***;
*******************************************************************;
data upcr_t_mono2;
      set upcr_t_mono;
      **Identify the subjects in the active groups we want to keep
  imputed as MAR;
      if ICEDT1>. or ICEDT2>. or ICEDT3>. then MAR = 'N';
      else MAR = 'Y';
      if trt01 = 2 then MAR = 'N';
```

```
      run;

**First impute general MAR based on regression method for all subjects to get
the true MAR;
proc mi data=upcr_t_mono2 out=upcr_step1 nimpute=1 seed=230815;
 by _Imputation_;
 class trtpn;
 monotone reg(v00 - v26/details);
 var stra1 stra2 distype immunobl trtpn v00 - v26;
run;

**Identify and keep the data from the subjects in the active group that
should remain as a MAR;
data upcr_step2;
      set upcr_step1;
      if Mar = "Y";
      run;

**Update the dataset and keep the MAR imputed data for the subjects in the
active group that should remain as a MAR, all others remain to be imputed
with control based imputation;
data upcr_t_mono3;
      update upcr_t_mono2 upcr_step2;
      by _Imputation_ subjid;
      run;

**Fill out the remaining missing where missing data should be imputed based
on the controls;
proc mi data=upcr_t_mono3 out=upcr_t_cbp nimpute=1 seed=230815;
 by _imputation_;
 class trtpn;
 var stra1 stra2 distype immunobl v00 - v26;
 monotone reg(/details);
 mnar model(v04 - v26 / modelobs= (trtpn='2'));
run;

**if there are ICEs of renal transplant/dialysis (ICEDT2>.) then need to
impute based on worst change;

** first, transpose the above upcr_t_cbp back to long format upcr_cbp(omit
the details), then, collect details on worst percentage across all visits
based on original data (e.g., name the variable as worstpchg), also, identify
for those subjects who had ICEDT2>., their first visit that should be imputed
based on worst percent change (e.g., name the variable as startavisit), plus,
for all 100 imputations, calculate the average log(uPCR) for each avisit
(e.g., name the variable as visit_avg), then;

data upcr_cbp_worst;
      merge upcr_cbp worstpchg startavisit visit_avg;
      by subjid;
      if avisitn>startavisit>. then aval = base*(1+ worstpchg/100) + (aval-
visit_avg);
      **Note, the "base*(1+ worstpchg/100)" part is worst change, the "(aval-
visit_avg)" is random error;
      run;

** lastly, transpose it back to the wide format;
```

```
proc transpose data= upcr_cbp_worst out= upcr_t_cbp_worst (drop= _NAME_
_LABEL_) prefix=V;
 by usubjid stra1 stra2 distype immunobl trtpn base;
 id avisitc;
 var aval;
run;



*******************************************************************;
*** Delta-Adjusted Pattern Imputation (tipping point analysis) ***;
*******************************************************************;
**First identify the subjects in the active treatment groups that we want to
apply the shift parameter if they have monotone missing data;
data upcr_t_mono2;
      set upcr_t_mono;

      **ICEDT1=. is just an example of a reason that we do not want to
  apply the shift parameter. The actual reasons are in the text;
      if ICEDT1=. then flag = 1;
      else flag = 0;

      if trtpn = 1 and flag = 0 then adjustthis = 1;
      else adjustthis = 0;
run;

proc mi data=upcr_t_mono2 out=upcr_t_tip nimpute=1 seed=230815;
 by _imputation_;
 class trtpn adjustthis;
 var stra1 stra2 distype immunobl trtpn v00 -- v12;
 monotone reg(/details);
 mnar adjust(v04 / shift=0.04 adjustobs=(adjustthis = '1' ))
  adjust(v08 / shift=0.04 adjustobs=(adjustthis = '1' ))
  adjust(v12 / shift=0.04 adjustobs=(adjustthis = '1' ))
  adjust(v16 / shift=0.04 adjustobs=(adjustthis = '1' ))
  adjust(v20 / shift=0.04 adjustobs=(adjustthis = '1' ))
  adjust(v24 / shift=0.04 adjustobs=(adjustthis = '1' ))
  adjust(v25 / shift=0.01 adjustobs=(adjustthis = '1' ))
  adjust(v26 / shift=0.01 adjustobs=(adjustthis = '1' ));
run;




*****************************************************;
*** SAS macro to run MIANALYZE on outputs from MMRM *;
*****************************************************;

%macro mi_results(in, lsm, dif); ** Note this macro is for individual visits
such as W4, W8,…,W24,W25,W26, see next macro for composite contrast of W24-
W25-W26 average;


proc transpose data= &in
    out= upcr_mi (rename= (_NAME_ = AVISITN COL1 = AVAL));
 by _imputation_ usubjid trtpn base stra1 stra2 distype immunobl;
```

```
 var V00 - V26;
run;

data upcr_mi;
 set upcr_mi;
 label AVISITN = 'AVISITN';
  chg = aval - base;
  * baseline record is not used in MMRM;
 if AVISITN = 'V00' then delete;
run;

proc sort; by _imputation_; run;

proc mixed data=upcr_mi method=reml covtest empirical;
 by _imputation_;
 class trtpn avisitn usubjid stra1 stra2 distype immunobl;
 model chg=trtpn stra1 stra2 distype immunobl avisitn trtpn*avisitn base /cl;
 repeated avisitn / subject=usubjid type=UN;
 lsmeans trtpn*avisitn / pdiff cl om alpha=.05;
 ods output diffs= diffs lsmeans= lsmeans;
run;

* In the final step, the analysis results obtained from PROC MIXED
  procedure are combined into a single estimation with standard error
  using PROC MIANALYZE;

proc sort data=lsmeans;
 by avisitn trtpn _imputation_;
run;
proc mianalyze parms=lsmeans;
 by avisitn trtpn;
 modeleffects trtpn*avisitn;
 ods output ParameterEstimates=&lsm;
run;

proc sort data=diffs (where= (_trtpn= 2 and (avisitn = _avisitn)));
 by avisitn trtpn _trtpn _imputation_;
run;

proc mianalyze parms=diffs;
 by avisitn trtpn _trtpn;
 modeleffects trtpn*avisitn;
 ods output ParameterEstimates=&dif;
run;

proc sql; *** LSMEANS using Multiple Imputation;
  select Parm as Effect, AVISITN, TRTPN, Estimate, StdErr, DF,
    tValue, Probt, LCLMean as L95, UCLMean as U95
 from &lsm;

  select Parm as Effect, AVISITN, TRTPN, _TRTPN, Estimate, StdErr, DF,
    tValue, Probt, LCLMean as L95, UCLMean as U95
 from &dif;
quit;
%mend;

%mi_results(in= upcr_t_cbp, lsm= lsm_cbp, dif=dif_cbp );
```

```
%mi_results(in= upcr_t_cbp_worst, lsm= lsm_cbp, dif=dif_cbp );


%macro mi_results(in, lsm, dif); ** Note this macro is for composite contrast
of W24-W25-W26 average;

proc sql;
      select avg(base) into: abs
      from &in
      group by _imputation_;
run;
%put abs=&abs;   * this will be used for the "estimate" statement, similarly
to obtain the empirical weight for each stratification factor and disease
type, namely stra1_wt, stra1_wt_rev, stra2_wt, stra2_wt_rev, distype_wt,
distype_wt_rev, immunobl_wt, immunobl_wt_rev to be used in next procedure;


proc transpose data= &in
    out= upcr_mi (rename= (_NAME_ = AVISITN COL1 = AVAL));
 by _imputation_ usubjid trtpn base stra1 stra2 distype immunobl;
 var V00 - V26;
run;

data upcr_mi;
 set upcr_mi;
 label AVISITN = 'AVISITN';
  chg = aval - base;
  * baseline record is not used in MMRM;
 if AVISITN = 'V00' then delete;
run;

proc sort; by _imputation_; run;

proc mixed data=upcr_mi method=reml covtest empirical;
 by _imputation_;
 class trtpn avisitn usubjid stra1 stra2 distype immunobl;
 model chg=trtpn stra1 stra2 distype immunobl avisitn trtpn*avisitn base /cl;
 repeated avisitn / subject=usubjid type=UN;
 lsmeans trtpn*avisitn / pdiff cl alpha=.05 om;
estimate 'PEG Week 24-25-26 Avg LSMean' intercept 1 stra1 &stra1_wt_rev
&stra1_wt stra2 &stra2_wt_rev &stra2_wt distype &distype_wt_rev &distype_wt
immunobl & immunobl_wt_rev & immunobl_wt trtpn 1 0
BASE &abs
avisitn 0 0 0 0 0 0.333 0.333 0.334
trtpn*avisitn 0 0 0 0 0 0.333 0.333 0.334
               0 0 0 0 0 0 0 0 /cl ;
estimate 'PEG vs PBO Difference Week 24-25-26 Avg' trtpn 1 -1
trtpn*avisitn 0 0 0 0 0 0.333 0.333 0.334
               0 0 0 0 0 -0.333 -0.333 -0.334 /cl ;
  ods output estimates = est;
run;

proc mianalyze data=est(where=(label='PEG Week 24-25-26 Avg LSMean'));
  modeleffects estimate;
  stderr stderr;
  ods output ParameterEstimates=&lsm;
run;
```

```
proc mianalyze data=est(where=(label='PEG vs PBO Difference Week 24-25-26
Avg'));
  modeleffects estimate;
  stderr stderr;
  ods output ParameterEstimates=&dif;
run;

proc sql; *** LSMEANS using Multiple Imputation;
  select Parm as Effect, Estimate, StdErr, DF,
       tValue, Probt, LCLMean as L95, UCLMean as U95
    from &lsm;

  select Parm as Effect,  Estimate, StdErr, DF,
       tValue, Probt, LCLMean as L95, UCLMean as U95
    from &dif;
quit;
%mend;

%mi_results(in= upcr_t_cbp, lsm= lsm_cbp, dif=dif_cbp );
%mi_results(in= upcr_t_cbp_worst, lsm= lsm_cbp, dif=dif_cbp );
```

### 17.2.3. Rate of Change Models

```
********************************;
*** Rate of Change analyses ***;

* In this example, repeated measures are available at
* avisitn = 0 (baseline), 4 (Week 4), 8 (Week 8), 12 (Week 12)
*   16 (Week 16), 20 (Week 20), 24 (Week 24), 25 (Week 25) and 26 (Week 26)
*
* AVAL is the actual average of triplicate FMU uPCR at the corresponding
visit.
* t is equivalent to AVISITN
*
* The data is read in from the control base imputation upcr_t_cbp, then
calling another macro to generate the "Week 0 to 26" slope for each arm as
well as the difference in slope between two arms
********************************;

********************************;
*This is the example if not based on multiple imputation
********************************;

proc mixed data=upcr method=reml covtest empirical;
      class subjid trtpn stra1 stra2 distype immunobl t;
      model aval = trtpn stra1 stra2 distype immunobl avisitn trtpn*avisitn
            /cl solution;
      repeated t /type=un sub=subjid r ;

      **Week 0 to 26;
  estimate 'PEG Week 0 to 26 slope' trtpn*avisitn 26 0   avisitn 26 /cl;
  estimate 'PBO Week 0 to 26 slope' trtpn*avisitn 0 26   avisitn 26 /cl;

  estimate 'Week 0 to Week 26 Difference in slope PEG - PBO'   trtpn*avisitn
26 -26 /cl;
run;


*****************************************************;
*** SAS macro to run MIANALYZE on outputs from multiple imputation dataset *;
*****************************************************;

%macro mi_results(in, lsm, dif);

proc transpose data= &in
    out= upcr_mi  (rename= (_NAME_ = AVISITN COL1 = AVAL));
 by _imputation_ usubjid trtpn base stra1 stra2 distype immunobl;
 var V00 - V26;
run;

data upcr_mi;
  set upcr_mi;
  label AVISITN = 'AVISITN';
 if avisitn = 'W00' then t = 0;
 else if avisitn = 'W04' then t = 4;
 else if avisitn = 'W08' then t = 8;
 else if avisitn = 'W12' then t = 12;
```

```
 else if avisitn = 'W16' then t = 16;
 else if avisitn = 'W20' then t = 20;
 else if avisitn = 'W24' then t = 24;
 else if avisitn = 'W25' then t = 25;
 else if avisitn = 'W26' then t = 26;
  drop avisitn base;
run;

data upcr_mi;
 set upcr_mi;
 avisitn = t;
run;

proc sort; by _imputation_; run;

proc mixed data=upcr_mi method=reml covtest empirical;
 by _imputation_;
 class trtpn usubjid stra1 stra2 distype immunobl t;
 model aval=trtpn stra1 stra2 distype immunobl avisitn trtpn*avisitn /cl;
 repeated t / subject=usubjid type=UN r;

  **Week 0 to 26;
  estimate 'PEG Week 0 to 26 slope' trtpn*avisitn 26 0   avisitn 26 /cl;
  estimate 'PBO Week 0 to 26 slope' trtpn *avisitn 0 26   avisitn 26 /cl;

  estimate 'Week 0 to 26 Difference in slope PEG - PBO'   trtpn *avisitn 26 -
26 /cl;

ods output estimates = est;
run;


proc mianalyze data=est(where=(label='PEG Week 0 to 26 slope')); * change
label for the other estimate;
  modeleffects estimate;
  stderr stderr;
  ods output ParameterEstimates=&lsm;
run;

proc mianalyze data=est(where=(label='Week 0 to 26 Difference in slope PEG -
PBO'));
  modeleffects estimate;
  stderr stderr;
  ods output ParameterEstimates=&dif;
run;

proc sql;
select Parm as Effect, Estimate, StdErr, DF,
       tValue, Probt, LCLMean as L95, UCLMean as U95
    from &lsm;

select Parm as Effect,  Estimate, StdErr, DF,
       tValue, Probt, LCLMean as L95, UCLMean as U95
    from &dif;
quit;
%mend;
```

```
%mi_results(in= upcr_t_cbp, lsm= lsm_cbp, dif=dif_cbp );
%mi_results(in= upcr_t_cbp_worst, lsm= lsm_cbp, dif=dif_cbp );
```

### 17.2.4. Logistic Model

```
*-----------------------------------------------------
* logistic regression, take example of FMU uPCR of at least 50% reduction
from baseline
*-----------------------------------------------------

* first select out the binary variable of the endpoint (avalc)
data upcr50;
 set ADAM.ADEFF;
 if paramcd = 'UPRTRD50';
 if ittfl='Y';
 if avisit = 'Week 26';
 if anl01fl='Y';
 keep subjid trt01p aval avalc param paramcd stragr1 stragr2p
disesbio;
run;


* then obtain the baseline variable (log-transformation) FMU uPCR value
data fmuupcr;
 set adam.adeff;
 if paramcd='LNUPRCRF';
 if ittfl='Y';
 if basetype='NORMAL';
 if avisit='Baseline';
 logbase = aval;
 keep subjid logbase;
run;

* then merge together to obtain all necessary variables
data upcr50;
 merge upcr50 fmuupcr;
 by subjid;
run;

proc sort data=upcr50; by trt01p;run;

** Descriptive summary of the data;
proc freq data=upcr50;
  by trt01p;
  tables avalc/nocol nopercent nocum;
run;


** logistic regression that produces Proportion (SE), CI, odd ratio
and CI, and p-value;
proc logistic data=upcr50;
 class trt01p(ref='Placebo') stragr1 stragr2p disesbio/param=glm;
 model avalc(event='Y') = trt01p logbase stragr1 stragr2p disesbio /
clodds=wald orpvalue;
 oddsratio trt01p;
```

```
 lsmeans trt01p / ilink e cl;
 store LogFit;
 ods output coef=Coeffs;
run;

** calling macro to produce difference in proportion and CI of it;
%NLMeans(instore=LogFit, coef=Coeffs, link=logit, title=Differences of
Proportion)
```

### 17.2.5. ANCOVA Model

```
*---------------------------------------------------
* Read Analysis data
*---------------------------------------------------
* Example used here is the key secondary endpoint of activity score of the
C3G Histologic Index at Week 26.
* Note the main analysis should be performed based on multiple imputation
dataset with ICE addressed (see sample code under 1.2.2), here is only an
illustration of the key code using data without the multiple imputation.
*---------------------------------------------------;

data activity;
 set ADAM.ADMI;
 if paramcd = 'C3HISACT';
 if ittfl='Y';
 if avisit = 'Week 26';
 if anl01fl='Y';
 keep usubjid trt01p chg base stragr1 stragr2p disesbio;
run;


proc mixed data=activity;
 by <subgroup>; *note: to add this line if for subgroup analysis;
 class trt01p disesbio stragr1 stragr2p;
 model chg = trt01p base disesbio stragr1 stragr2p;
 lsmeans trt01p /om cl pdiff;
run;
```

### 17.2.6. Time-to-Event Analysis

```
proc lifetest data=adtte;
 time aval*cnsr(1);
 strata stragr1 stragr2p disesbio / group=trt01p;
run;

proc phreg data=adtte;
 class trt01p/ param=reference ref=last;
 model aval*cnsr(1) = trt01p loguPCR_BL eGFR_BL/rl ties=efron;
 strata stragr1 stragr2p disesbio;
run;
```

**SUMMARY OF VALIANT STATISTICAL ANALYSIS PLAN CHANGES**

| **Statistical Analysis Plan v2 Amendments – Description of Change** |
|---|
| Revised the order of key secondary efficacy endpoints; change from baseline in eGFR is now the last key secondary endpoint. |
| Removed exploratory endpoint evaluating normalization of hematuria. |
| Removed exploratory endpoint of evaluation of changes in drusen at 26 weeks. |
| Updated strategies for addressing ICEs |
| Removed mITT set from statistical analysis sets. |
| Provided further details on the analysis of C3c staining endpoint. |
| Provided further details on the analysis of time-to-event endpoints. |
| Provided further details on the analyses of FACIT-Fatigue score, EQ5D-5L score, and WPAI score endpoints. |
| Provided further details on immunogenicity analysis. |
| Provided further details on the analysis of baseline disease characteristics. |
| Updated strategies for addressing missing data in PP analysis. |
| Clarified more details on the covariance structures for the MMRM models. |
| Provided further details on the analysis window mapping for roll-over participants. |
| For endpoints of C3c histologic activity score, FACIT-Fatigue score, and KDQOL score, clarified in the null/alternative hypothesis that the change from baseline is being modeled instead of the value at week 26. |
| Clarified for the key secondary endpoints of activity score and C3c staining reduction, modeling will only be based on adult participants. |
| Updated analysis for the exploratory endpoint of annual rate of change from up to 3 years prior to screening in eGFR. |
| Updated analysis for the exploratory endpoint of glomerular macrophage count. |
| Updated analysis for the exploratory endpoint of PGIC score. |
| Provided more details on the activity score endpoint regarding the primary, secondary, and adjudication reports. |
| Updated to assign different randomization seeds for different endpoints. |
| Updated safety analysis to add summary tables for AESIs and exposure-adjusted AEs. |
| Added new subgroup "baseline immunosuppressants use" in all subgroup analyses, the new subgroup has also been added in primary analysis modeling |
| Added clarification languages. |
